# Supplementary material for: Supramolecular catalysis with ethers enabled by dual chalcogen bonding activation
Source: Nat Commun. 2023 Oct 10;14:6347. doi: 10.1038/s41467-023-42129-1 (PMC10564790; doi:10.1038/s41467-023-42129-1)
Supplement: Supplementary file 1 — Supplementary Information [file 41467_2023_42129_MOESM1_ESM.pdf]

Supplementary Information

Supramolecular Catalysis with Ethers Enabled by Dual Chalcogen Bonding Activation

Zhiguo Zhao[1], Yuanling Pang[1], Ziqiang Zhao[1], Pan-Pan Zhou[2], Yao Wang*[1]

[1]School of Chemistry and Chemical Engineering, Key Laboratory of the Colloid and Interface Chemistry of the Ministry of Education, Shandong University, Jinan 250100, China

[2]College of Chemistry and Chemical Engineering, Key Laboratory of Special Function Materials and Structure Design of Ministry of Education, Lanzhou University, Lanzhou, 730000, China

*Corresponding Author: yaowang@sdu.edu.cn

Table of Contents:

1. General Information……S2

2. Preparation of Catalysts and Substrate……S2

3. Optimization of Reaction Condition……S10

4. Optimized Procedure……S11

5. Analytical Data……S11

6. Mechanistic Study……S19

7. DFT Calculations. ……S32

8. X-ray Crystallographic Data……S34

9. Copies of NMR Spectra……S39

10. Supplementary References……S102

1. General information

All the chemicals were either purchased from commercial suppliers or purified by standard procedures as specified in *Purification of Laboratory Chemicals*, 7th Ed (Armarego, W. L. F.; Chai, C. L. L. Butterworth Heinemann: 2013). All manipulations were carried out by using standard Schlenk techniques. All solvents were purified and degassed prior to use. Analytical thin-layer chromatography (TLC) was performed on silica gel plates and analyzed by UV light or by potassium permanganate stains followed by heating. Flash chromatography was carried out utilizing silica gel (200-300 mesh). ^{1}H NMR, ^{13}C NMR spectra were recorded in $CDCl_3$ or CD_2Cl_2 at room temperature on a Bruker AM-400 spectrometer (400 MHz ^{1}H, 100 MHz ^{13}C). The chemical shifts are reported in ppm relative to either the residual solvent peak (^{13}C) (δ = 77.00 ppm for $CDCl_3$; δ = 53.84 ppm for CD_2Cl_2), (^{1}H) (δ = 7.26 ppm for $CDCl_3$; δ = 5.32 ppm for CD_2Cl_2, δ = 0 ppm for TMS) as an internal standard or using PhSeSePh as a reference compound (^{77}Se) (δ = 461.58 ppm for PhSeSePh) as an external standard. Data for ^{1}H NMR are reported as follows: chemical shift (δ ppm), multiplicity (s = singlet, d = doublet, t = triplet, q = quartet, m = multiplet, dd = doublet doublet), coupling constant (Hz), integration. Data for ^{13}C NMR are reported as chemical shift. HRMS were performed on a Bruker Apex II mass instrument (ESI) and Agilent Technologies 7250 GCQTOF (EI).

2. Preparation of Catalysts and Substrate

(1) Catalysts **Ch1-15** as depicted below were evaluated in this work:

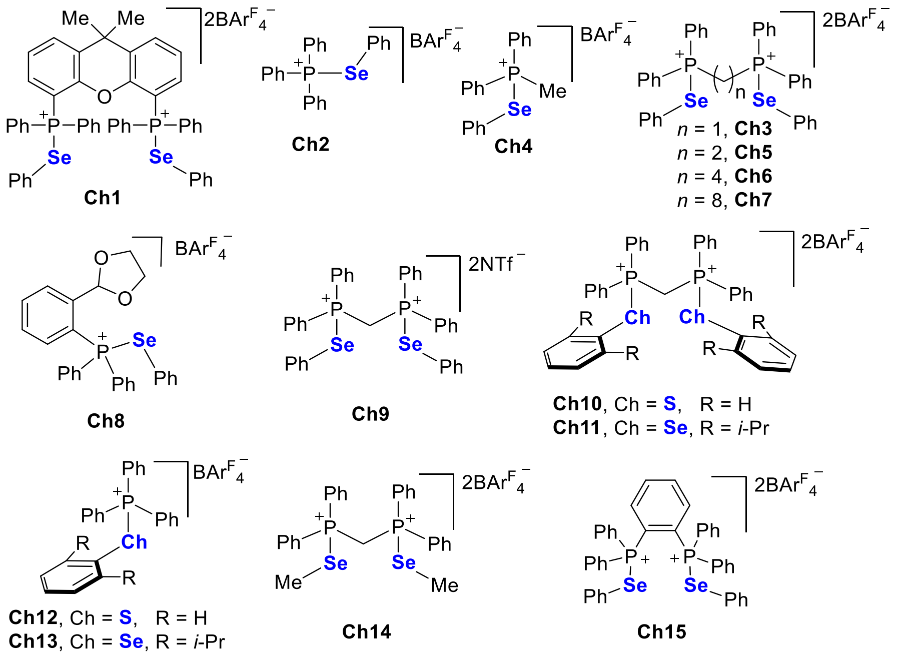

Supplementary Fig. 1 Catalysts Ch1-15.

Catalysts **Ch1**-**Ch5** and **Ch15** were prepared using the following procedure:

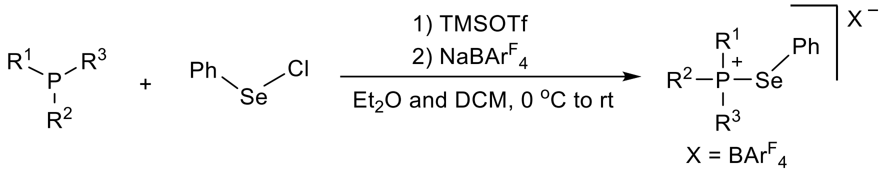

General procedure：To a red solution of PhSeCl (2.0 mmol for **Ch1**, **3**, **5**, **15**; 1.0 mmol for **Ch2** and **Ch4**) in dry Et_2O (6.0 mL) at 0 °C under argon was added TMSOTf (2.0 mmol for **Ch1**, **3**, **5**, **15**; 1.0 mmol for **C2** and **Ch4**). The reaction mixture was allowed to warm to room temperature and stirred for 40 minutes to give a dark orange solution. Then corresponding phosphine (1.0 mmol) in dry CH_2Cl_2 (4.0 mL) was added over 5 minutes at 0 °C. The reaction mixture was allowed to warm to room temperature and stand for 1 h. The white solid suspension was filtered and washed by anhydrous diethyl ether. Then tetrakis[3,5-bis(trifluoromethyl)phenyl]borate (2.0 mmol for **Ch1**, **3**, **5**, **15**; 1.0 mmol for **Ch2** and **Ch4**) was added to a solution of the above white solid (1.0 mmol) in dry CH_2Cl_2 (10.0 mL) under argon and the reaction mixture was stirred at room temperature for 24 h. Then the reaction mixture was filtered and the filtrate was concentrated to give a saturated solution under reduced pressure and then 10.0 mL *n*-hexane was slowly added. The two-phase solution was then placed at room temperature under argon and the desirable product precipitates out as a white solid. Then the precipitated white solid was collected by filtration and recrystallized twice from CH_2Cl_2 (or ether) and *n*-hexane to afford pure catalysts **Ch1**-**Ch5** and **Ch15**.

Catalysts **Ch1**-**Ch5** and **Ch15** are known compounds. The spectral data of these catalysts are in accordance with literature.[1-5]

Catalysts **Ch6**-**Ch13** were prepared using the following procedure:

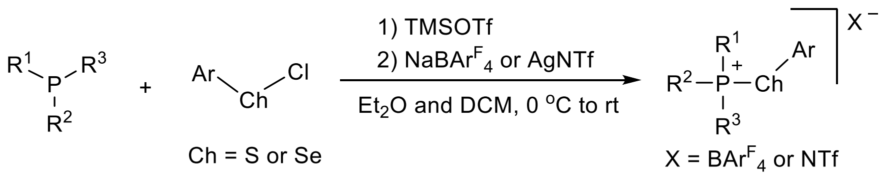

General procedure：To a red solution of PhSeCl or PhSCl (2.0 mmol, 1.0 mmol for **Ch8**, **Ch12** and **Ch13**) in dry Et_2O (6.0 mL) at 0 °C under argon was added TMSOTf (2.0 mmol, 1.0 mmol for **Ch8**, **Ch12** and **Ch13**). The reaction mixture was allowed to warm to room temperature and stirred for 40 minutes to give a dark orange solution. Then phosphine (1.0 mmol) in dry CH_2Cl_2 (4.0 mL) was added over 5 minutes at 0 °C. The reaction mixture was allowed to warm to room temperature and stand for 1 h. The white solid suspension was filtered and washed by anhydrous diethyl ether. Then tetrakis[3,5-bis(trifluoromethyl)phenyl]borate (2.0 mmol, 1.0 mmol for **Ch8**, **Ch12** and **Ch13**) or sliver bis(trifluoromethane sulfonimide) (2.0 mmol for **Ch9**) was added to a solution of the above white solid (1.0

mmol) in dry CH_2Cl_2 (10.0 mL) under argon and the reaction mixture was stirred at room temperature for 24 h. Then the reaction mixture filtered and the filtrate was concentrated to give a saturated solution under reduced pressure and then 10.0 mL *n*-hexane was slowly added. The two-phase solution was then placed at room temperature under argon and the desirable product precipitates out as a white solid. Then the precipitated white solid was collected by filtration and recrystallized twice from CH_2Cl_2 (or ether) and *n*-hexane to afford pure catalysts **Ch6**-**Ch13**.

Catalyst **Ch14** was prepared using the following procedure:[6]

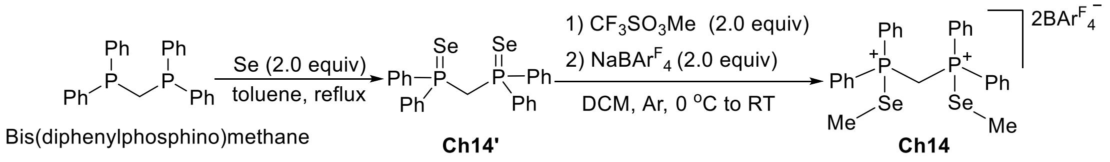

General procedure： To a solution of bis(diphenylphosphino)methane (768.8 mg, 2.0 mmol, 1.0 equiv) in dry toluene (5.0 mL) at room temperature under argon was added Se (315.8 mg, 4.0 mmol, 2.0 equiv). The reaction mixture was refluxed for 5 h, cooled, and evaporated. The obtained residue was purified by a silica gel chromatography column (CH_2Cl_2) to give **Ch14'** (1000.9 mg, 1.84 mmol, 92% yield). To a solution of **Ch14'** (543.9 mg, 1.0 mmol, 1.0 equiv) in dry CH_2Cl_2 (5.0 mL) under argon was added CF_3SO_3Me (226.0 μL, 2.0 mmol, 2.0 equiv) dropwise in ice-water bath. The reaction mixture was allowed to warm to room temperature and stand for 2 h. Then tetrakis[3,5-bis(trifluoromethyl)phenyl]borate (1772.4 mg, 2.0 mmol, 2.0 equiv) was added to the above solution and the reaction mixture was stirred at room temperature for 24 h. Then the reaction mixture filtered and the filtrate was concentrated to give a saturated solution under reduced pressure and then 10.0 mL *n*-hexane was slowly added. The two-phase solution was then placed at room temperature under argon and the desirable product precipitates out as a white solid. The precipitated white solid was collected by filtration and recrystallized twice from CH_2Cl_2 and *n*-hexane to afford pure catalyst **Ch14** (1679.1 mg, 0.73 mmol, 73% yield).

(2) Catalyst **XB** was prepared using the following procedure:

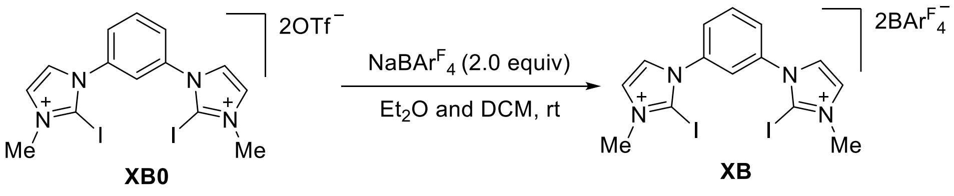

Catalyst **XB0** was prepared according to the literature procedure.[7] Then tetrakis[3,5-bis(trifluoromethyl)phenyl]borate (1772.4 mg, 2.0 mmol, 2.0 equiv) was added to a solution of **XB0** (789.8 mg, 1.0 mmol, 1.0 equiv) in dry CH_2Cl_2 (10.0 mL) under argon and the reaction mixture was stirred at room temperature for 24 h. Then the reaction mixture filtered and the filtrate was concentrated to give a saturated solution under reduced

pressure and then 10.0 mL *n*-hexane was slowly added. The two-phase solution was then placed at room temperature under argon and the desirable product precipitates out as a white solid. Then the precipitated white solid was collected by filtration and recrystallized twice from CH_2Cl_2 and *n*-hexane to afford pure catalyst **XB** (1929.7 mg, 0.87 mmol, 87% yield).

(3) Ethers **e4** and **e5** were prepared using the following procedure:

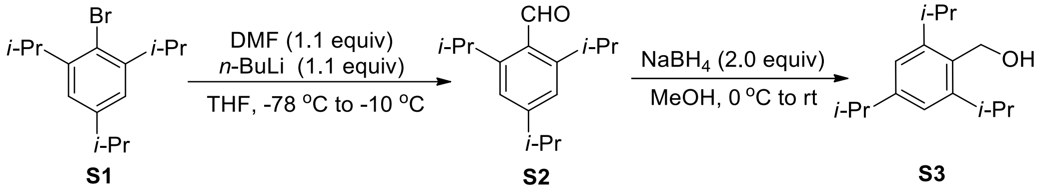

(2,4,6-triisopropylphenyl)methanol (**S3**): To a solutionof 2-bromo-1,3,5-triisopropylbenzene **S1** (2832.5 mg, 10.0 mmol, 1.0 equiv**)** in 20.0 mL of dry THF, kept under argon at -78°C, was added *n*-BuLi (4.4 mL, 2.5 M in hexane, 11.0 mmol, 1.1 equiv) over 20 minutes. The solution was left reacting for additional 20 min, then dry DMF (850.0 μL, 11.0 mmol, 1.1 equiv) was slowly dropped keeping the temperature below -78°C. After stirring for 15 min the mixture was allowed to warm to -10°C, then was quenched with water, extracted with ether and dried over anhydrous sodium sulphate. After removing the solvent at reduced pressure, the crude was purified by a silica gel chromatography column (petroleum ether/ethyl acetate, 10:1) to give **S2** (1741.4 mg, 0.75 mmol, 75% yield). To a solution of **S2** (1393.1 mg, 6.0 mmol, 1.0 equiv) in MeOH (10.0 mL) was added $NaBH_4$ (453.9 mg, 12.0 mmol, 2.0 equiv) in portions in ice-water bath. The mixture was stirred at ambient temperature over 8 h. To the mixture was quenched by diluted aqueous HCl (1.0 M). Organic compounds were extracted with CH_2Cl_2 (3 × 10.0 mL). Organic layer was combined, washed with brine, dried over Na_2SO_4 and the solvent was evaporated under reduced pressure. The obtained residue was purified by a silica gel chromatography column (petroleum ether/ethyl acetate, 3:1) to give **S3** (1278.7 mg, 5.46 mmol, 91% yield).

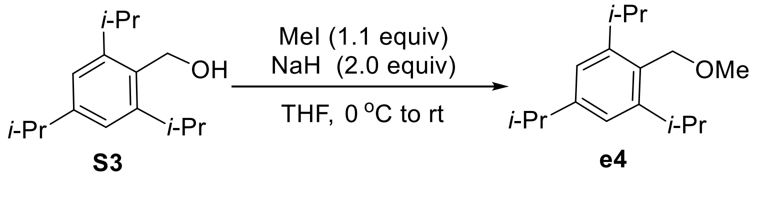

1,3,5-triisopropyl-2-(methoxymethyl)benzene (**e4**): To a 25 mL round bottom flask was added NaH (60% dispersion in mineral oil, pre-washed with dry hexane, 120.0 mg, 5.0 mmol, 2.0 equiv). The flask was evacuated and filled with argon (3 times). Anhydrous THF (10.0 mL) was added by syringe and the reaction mixture was cooled to 0 °C in an ice bath. **S3** (585.5 mg, 2.5 mmol, 1.0 equiv) in THF (5.0 mL) was added dropwise and the reaction mixture was allowed to warm to room temperature and stirred for 30 minutes. Following, iodomethane (170.0 μL, 2.75 mmol,

1.1 equiv) was added and the reaction was stirred at room temperature for 8 h. The reaction was quenched by the addition of saturated aqueous NH_4Cl (20.0 mL) and the phases were separated. The aqueous layer was extracted with diethyl ether (3 × 10.0 mL). The organic layer was combined, washed with brine, dried over Na_2SO_4 and the solvent was evaporated under reduced pressure. The obtained residue was purified by a silica gel chromatography column (petroleum ether/ethyl acetate, 100:1) to give **e4** (577.1 mg, 2.3 mmol, 93% yield).

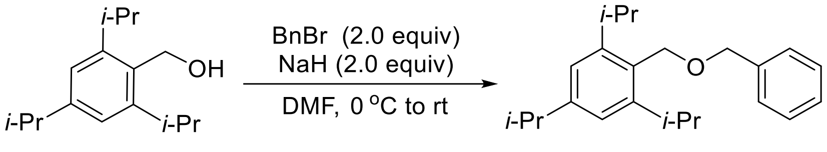

2-((benzyloxy)methyl)-1,3,5-triisopropylbenzene (**e5**): In a 25 mL round bottom flask, **S3** (351.3 mg, 1.5 mmol, 1.0 equiv) was dissolved in 6.0 mL of DMF under argon atmosphere. The solution was transferred via cannula to another flask containing DMF (5.0 mL) solution of sodium hydride (72.0 mg, 3.0 mmol, 2.0 equiv, 60% dispersion in mineral oil, pre-washed with dry hexane) under ice-cold condition and the solution was stirred for 30 minutes. To this solution, BnBr (356.0 μL, 3.0 mmol, 2.0 equiv) dissolved in 6.0 mL of DMF was slowly added under ice-cold condition and then the reaction mixture was warm to room temperature and stirred. Reaction progress was monitored by TLC. Upon completion, the reaction mixture was poured into ice-cold water and extracted with CH_2Cl_2 (3 × 10.0 mL). The combined organic layers were dried over anhydrous Na_2SO_4 and evaporated under reduced pressure. The obtained residue was purified by a silica gel chromatography column (petroleum ether/ethyl acetate, 100:1) to give **e5** (437.7 mg, 1.35 mmol, 90% yield).

Ch1 was prepared according to the general procedure. White solid (77% yield). ^{1}H NMR (400 MHz, CD_2Cl_2): δ 8.11 (d, *J* = 7.9 Hz, 2H), 7.85–7.70 (m, 16H), 7.67–7.54 (m, 12H), 7.53–7.27 (m, 20H), 7.17 (t, *J* = 7.9 Hz, 6H), 7.05–6.97 (m, 4H), 1.89 (s, 6H); ^{13}C NMR (100 MHz, CD_2Cl_2): δ 162.24 (q, *J* = 49.5 Hz), 152.89 (d, *J* = 2.7 Hz), 137.59 (d, *J* = 3.7 Hz), 137.16 (d, *J* = 7.9 Hz), 136.82 (d, *J* = 2.7 Hz), 136.69 (d, *J* = 3.3 Hz), 135.28 (bs), 134.39 (d, *J* = 10.6 Hz), 133.72 (d, *J* = 6.6 Hz), 132.75 (d, *J* = 3.6 Hz), 131.32 (d, *J* = 2.9 Hz), 131.12, 130.98, 129.38 (qq, *J* = 31.3, 3.0 Hz), 126.63 (d, *J* = 13.2 Hz), 125.05 (q, *J* = 270.9 Hz), 120.68 (d, *J* = 8.3 Hz), 119.59 (d, *J* = 78.4 Hz), 118.40–117.62 (m), 105.34 (d, *J* = 77.4 Hz), 35.24, 33.73; ^{31}P NMR (162 MHz, CD_2Cl_2): δ 30.95; ^{77}Se NMR (76 MHz, $CDCl_3$): δ 343.14 (d, $J_{Se\text{-}P}$ = 479.6 Hz); ^{19}F NMR (376 MHz, $CDCl_3$): δ -62.73; HRMS (ESI+) exact mass calculated for $[M]^{2+}$ ($C_{51}H_{42}OP_2Se_2$) requires m/z 446.0515, found m/z 446.0520.

Ch2 was prepared according to the general procedure. White solid (78% yield). ^{1}H NMR (400 MHz, CD_2Cl_2): δ 7.90–7.71 (m, 11H), 7.70–7.42 (m, 17H), 7.33–7.15 (m, 4H); ^{13}C NMR (100 MHz, CD_2Cl_2): δ 162.36 (q, *J* = 49.5

Hz), 138.23 (d, J = 3.3 Hz), 136.44 (d, J = 3.3 Hz), 135.37 (bs), 134.32 (d, J = 10.7 Hz), 132.57 (d, J = 3.4 Hz), 131.32 (d, J = 2.8 Hz), 130.97 (d, J = 13.5 Hz), 129.51 (qq, J = 31.1, 2.5 Hz), 125.13 (q, J = 270.8 Hz), 119.33 (d, J = 7.2 Hz), 118.78 (d, J = 77.5 Hz), 118.24–117.79 (m); ^{31}P NMR (162 MHz, CD_2Cl_2): δ 39.43; ^{77}Se NMR (76 MHz, CD_2Cl_2): δ 314.74 (d, $J_{Se\text{-}P}$ = 454.6 Hz); ^{19}F NMR (376 MHz, CD_2Cl_2): δ -62.72; HRMS (ESI+) exact mass calculated for $[M]^+$ ($C_{24}H_{20}PSe$) requires m/z 419.0462, found m/z 419.0458.

Ch3 was prepared according to the general procedure. White solid (67% yield). ^{1}H NMR (400 MHz, CD_2Cl_2): δ 7.89–7.69 (m, 20H), 7.68–7.52 (m, 16H), 7.51–7.40 (m, 8H), 7.34 (t, J = 7.7 Hz, 2H), 7.09 (t, J = 7.7 Hz, 4H), 7.02–6.89 (m, 4H), 4.59 (t, J = 13.7 Hz, 2H); ^{13}C NMR (100 MHz, CD_2Cl_2): δ 162.33 (q, J = 49.4 Hz), 138.52, 137.64, 135.37 (bs), 133.82, 133.73 (d, J = 5.6 Hz), 133.36, 132.09, 132.02, 131.95, 131.86, 129.46 (qq, J = 31.3, 2.8 Hz), 125.14 (q, J = 270.9 Hz), 118.66–117.60 (m), 114.88 (d, J = 76.7 Hz), 28.69 (t, J = 37.4 Hz); ^{31}P NMR (162 MHz, CD_2Cl_2): δ 30.27; ^{77}Se NMR (76 MHz, CD_2Cl_2): δ 312.59 (d, $J_{Se\text{-}P}$ = 486.9 Hz); ^{19}F NMR (376 MHz, CD_2Cl_2): δ -62.63; HRMS (ESI+) exact mass calculated for $[M]^{2+}$ ($C_{37}H_{32}P_2Se_2$) requires m/z 349.0149, found m/z 349.0153.

Ch4 was prepared according to the general procedure. White solid (87% yield). ^{1}H NMR (400 MHz, 298K, CD_2Cl_2) δ 7.83–7.74 (m, 10H), 7.68–7.56 (m, 12H), 7.52–7.43 (m, 1H), 7.40–7.22 (m, 4H), 2.52 (d, J = 13.3 Hz, 3H); ^{13}C NMR (100 MHz, 298K, CD_2Cl_2) δ 162.27 (q, J = 49.5 Hz), 137.83 (d, J = 3.4 Hz), 136.55 (d, J = 3.3 Hz), 135.30 (bs), 132.81 (d, J = 10.8 Hz), 131.46 (d, J = 2.8 Hz), 131.09 (d, J = 13.4 Hz), 129.41 (qq, J = 31.4, 2.9 Hz), 125.08 (q, J = 270.6 Hz), 119.59, 118.82, 118.42 (d, J = 7.1 Hz), 118.16–117.78 (m), 13.15 (d, J = 49.6 Hz); ^{31}P NMR (162 MHz, 298K, CD_2Cl_2) δ 33.07; ^{77}Se NMR (76 MHz, 298K, CD_2Cl_2) δ 307.84 (d, J Se-P = 442.8 Hz); ^{19}F NMR (376 MHz, 298K, CD_2Cl_2) δ -62.75; HRMS (ESI+) exact mass calculated for $[M]^+$ ($C_{19}H_{18}PSe$) requires m/z 357.0306, found m/z 357.0302.

Ch5 was prepared according to the general procedure. White solid (74% yield). ^{1}H NMR (400 MHz, CD_2Cl_2): δ 7.86–7.72 (m, 20H), 7.66–7.52 (m, 16H),7.47–7.31 (m, 10H), 7.14 (t, J = 7.9 Hz, 4H), 6.95 (d, J = 6.9 Hz, 4H), 2.80 (d, J = 3.6 Hz, 4H); ^{13}C NMR (100 MHz, CD_2Cl_2): δ 162.14 (q, J = 49.6 Hz), 137.93, 137.43, 135.18 (bs), 133.18 (d, J = 3.2 Hz), 133.09 (d, J = 5.3 Hz), 131.89, 131.78 (d, J = 6.7 Hz), 129.26 (qq, J = 31.4, 2.9 Hz), 124.96 (q, J = 270.7 Hz), 118.14–117.63 (m), 116.41, 115.16 (d, J = 74.8 Hz), 20.44 (d, J = 21.0 Hz); ^{31}P NMR (162 MHz, CD_2Cl_2): δ 41.90; ^{77}Se NMR (76 MHz, CD_2Cl_2): δ 275.18 (d, $J_{Se\text{-}P}$ = 469.8 Hz); ^{19}F NMR (376 MHz, CD_2Cl_2): δ -62.66; HRMS (ESI+) exact mass calculated for $[M]^{2+}$ ($C_{38}H_{34}P_2Se_2$) requires m/z 356.0228 found m/z 356.0232.

Ch6 was prepared according to the general procedure. White solid (78% yield). ^{1}H NMR (400 MHz, CD_2Cl_2): δ

7.92–7.67 (m, 20H), 7.66–7.30 (m, 26H), 7.25–7.00 (m, 8H), 2.83–2.62 (m, 4H), 1.80–1.55 (m, 4H); ^{13}C NMR (100 MHz, CD_2Cl_2): δ 162.19 (q, *J* = 49.5 Hz), 137.85, 136.15 (d, *J* = 3.1 Hz), 135.23 (bs), 133.66 (d, *J* = 10.6 Hz), 132.14, 131.14, 130.85 (d, *J* = 13.2 Hz), 129.32 (qq, *J* = 31.3, 2.8 Hz), 125.01 (q, *J* = 270.8 Hz), 118.12 (d, *J* = 74.0 Hz), 118.10–117.80 (m), 24.85 (d, *J* = 42.4 Hz), 23.38 (dd, *J* = 19.9, 3.6 Hz); ^{31}P NMR (162 MHz, CD_2Cl_2): δ 40.15; ^{77}Se NMR (76 MHz, CD_2Cl_2): δ 279.69 (d, $J_{Se\text{-}P}$ = 444.6 Hz); ^{19}F NMR (376 MHz, CD_2Cl_2): δ -62.75; HRMS (ESI+) exact mass calculated for $[M]^{2+}$ ($C_{40}H_{38}P_2Se_2$) requires m/z 370.0384, found m/z 370.0381.

Ch7 was prepared according to the general procedure. White solid (83% yield). ^{1}H NMR (400 MHz, CD_2Cl_2): δ 7.80–7.58 (m, 20H), 7.57–7.34 (m, 24H), 7.33–7.24 (m, 2H), 7.17–6.95 (m, 8H), 2.65–2.42 (m, 4H), 1.51–1.34 (m, 4H), 1.33–1.20 (m, 4H), 1.17–0.99 (m, 4H); ^{13}C NMR (100 MHz, CD_2Cl_2): δ 162.27 (q, *J* = 49.5 Hz), 137.78 (d, *J* = 3.3 Hz), 136.42 (d, *J* = 3.3 Hz), 135.30 (bs), 133.30 (d, *J* = 10.1 Hz), 132.42 (d, *J* = 3.3 Hz), 131.28 (d, *J* = 2.8 Hz), 130.96 (d, *J* = 13.0 Hz), 129.41 (qq, *J* = 31.3, 3.0 Hz), 125.07 (q, *J* = 270.7 Hz), 118.18–117.80 (m), 118.12 (d, *J* = 73.8 Hz), 30.68 (d, *J* = 16.9 Hz), 29.22, 25.96 (d, *J* = 41.2 Hz), 22.98 (d, *J* = 4.4 Hz); ^{31}P NMR (162 MHz, CD_2Cl_2): δ 40.97; ^{77}Se NMR (76 MHz, CD_2Cl_2): δ 278.80 (d, $J_{Se\text{-}P}$ = 441.6 Hz); ^{19}F NMR (376 MHz, CD_2Cl_2): δ -62.71; HRMS (ESI+) exact mass calculated for $[M]^{2+}$ ($C_{44}H_{46}P_2Se_2$) requires m/z 398.0725, found m/z 398.0727.

Ch8 was prepared according to the general procedure. White solid (66% yield). ^{1}H NMR (400 MHz, $CDCl_3$): δ 7.95–7.86 (m, 1H), 7.82–7.75 (m, 1H), 7.74–7.66 (m, 8H), 7.62–7.54 (m, 3H), 7.53–7.46 (m, 5H), 7.45–7.37 (m, 4H), 7.36–7.26 (m, 5H), 7.19–7.10 (m, 2H), 7.09–6.97 (m, 2H), 6.15 (s, 1H), 3.74–3.61 (m, 2H), 3.35–3.20 (m, 2H); ^{13}C NMR (100 MHz, $CDCl_3$): δ 161.70 (q, *J* =49.5 Hz), 144.49 (d, *J* = 7.9 Hz), 137.23 (d, *J* = 3.9 Hz), 135.80 (s), 135.73 (d, *J* = 7.9 Hz), 134.79 (bs), 133.17 (d, *J* = 10.3 Hz), 131.55 (d, *J* = 3.7 Hz), 130.12 (d, *J* = 3.1 Hz), 129.79 (d, *J* = 11.2 Hz), 129.61 (d, *J* = 3.8 Hz), 128.92 (qq, *J* = 31.3, 2.9 Hz), 124.52 (q, *J* = 270.9 Hz), 122.96 (d, *J* = 8.1 Hz), 120.52 (d, *J* = 78.4 Hz), 118.25–117.18 (m), 100.58 (d, *J* = 2.2 Hz), 64.72 (s); ^{31}P NMR (162 MHz, $CDCl_3$): δ 44.15; ^{77}Se NMR (76 MHz, $CDCl_3$): δ 363.80 (d, $J_{Se\text{-}P}$ = 487.9 Hz); ^{19}F NMR (376 MHz, $CDCl_3$): δ -62.36; HRMS (ESI+) exact mass calculated for $[M]^+$ ($C_{27}H_{24}O_2PSe$) requires m/z 491.0674, found m/z 491.0675.

Ch9 was prepared according to the general procedure. White solid (64% yield). ^{1}H NMR (400 MHz, CD_2Cl_2): δ 7.85–7.75 (m, 4H), 7.72–7.55 (m, 16H), 7.34–7.23 (m, 2H), 7.08–6.99 (m, 8H), 5.50 (t, *J* = 14.6 Hz, 2H); ^{13}C NMR (100 MHz, CD_2Cl_2): δ 137.94, 137.17, 134.43 (d, *J* = 11.5 Hz), 132.20, 131.26 (d, *J* = 14.1 Hz), 131.06, 120.23 (q, *J* = 319.4 Hz), 119.12, 26.86 (t, *J* = 37.7 Hz); ^{31}P NMR (162 MHz, CD_2Cl_2): δ 32.69; ^{77}Se NMR (76 MHz, CD_2Cl_2): δ 312.48 (d, $J_{Se\text{-}P}$ = 480.9 Hz); ^{19}F NMR (376 MHz, CD_2Cl_2): δ -79.21; HRMS (ESI+) exact mass calculated for $[M]^{2+}$ ($C_{37}H_{32}P_2Se_2$) requires m/z 349.0149, found m/z 349.0146.

Ch10 was prepared according to the general procedure. White solid (58% yield). ^{1}H NMR (400 MHz, CD_2Cl_2): δ 7.87–7.78 (m, 20H), 7.69–7.58 (m, 16H), 7.57–7.49 (m, 8H), 7.39 (t, J = 7.5 Hz, 2H), 7.16 (t, J = 7.9 Hz, 4H), 7.00 (d, J = 7.0 Hz, 4H), 4.61 (t, J = 13.8 Hz, 2H); ^{13}C NMR (100 MHz, CD_2Cl_2): δ 162.32 (q, J = 49.6 Hz), 138.66, 136.99, 135.35 (bs), 133.77 (d, J = 5.5 Hz), 133.68, 131.99, 131.92, 131.85, 131.81, 129.45 (qq, J = 31.3, 2.9 Hz), 125.14 (q, J = 270.8 Hz), 118.26–117.83 (m), 114.74 (d, J = 84.9 Hz), 27.85 (t, J = 43.8 Hz); ^{31}P NMR (162 MHz, CD_2Cl_2): δ 42.30; ^{19}F NMR (376 MHz, CD_2Cl_2): δ -62.68; HRMS (ESI+) exact mass calculated for $[M]^{2+}$ ($C_{37}H_{32}P_2S_2$) requires m/z 301.0705, found m/z 301.0709.

Ch11 was prepared according to the general procedure. White solid (61% yield). ^{1}H NMR (400 MHz, CD_2Cl_2): δ 7.81–7.70 (m, 20H), 7.61–7.48 (m, 16H), 7.42–7.26 (m, 10H), 6.98 (d, J = 7.7 Hz, 4H), 4.71 (t, J = 13.6 Hz, 2H), 2.97–2.76 (m, 4H), 0.96 (d, J = 6.8 Hz, 24H); ^{13}C NMR (100 MHz, CD_2Cl_2): δ 162.30 (q, J = 49.5 Hz), 155.37, 155.33, 136.24 (d, J = 3.5 Hz), 135.34 (bs), 133.57 (d, J = 2.1 Hz), 133.47 (d, J = 2.1 Hz), 133.23, 133.01, 131.02, 130.61, 130.48, 129.82, 129.74, 129.43 (qq, J = 31.2, 2.8 Hz), 125.85, 125.82, 125.12 (q, J = 270.6 Hz), 118.57 (dd, J = 73.7, 2.2 Hz), 118.15–117.83 (m), 35.76, 28.39 (dd, J = 43.2, 37.5 Hz); ^{31}P NMR (162 MHz, CD_2Cl_2): δ 27.63; ^{77}Se NMR (76 MHz, CD_2Cl_2): δ 152.4 (dd, $J_{Se\text{-}P}$ = 472.7, 55.0 Hz); ^{19}F NMR (376 MHz, CD_2Cl_2): δ -62.69; HRMS (ESI+) exact mass calculated for $[M]^{2+}$ ($C_{49}H_{56}P_2Se_2$) requires m/z 433.1088, found m/z 433.1081.

Ch12 was prepared according to the general procedure. White solid (76% yield). ^{1}H NMR (400 MHz, CD_2Cl_2): δ 7.87–7.76 (m, 11H), 7.69–7.51 (m, 16H), 7.50–7.44 (m, 1H), 7.33–7.10 (m, 4H); ^{13}C NMR (100 MHz, CD_2Cl_2): δ 162.29 (q, J = 49.4 Hz), 137.51 (d, J = 3.9 Hz), 136.67 (d, J = 3.2 Hz), 135.31 (bs), 134.35 (d, J = 10.7 Hz), 132.88 (d, J = 3.6 Hz), 131.23 (d, J = 3.0 Hz), 130.95 (d, J = 13.5 Hz), 129.44 (qq, J = 31.3, 2.9 Hz), 125.09 (q, J = 270.8 Hz), 120.08 (d, J = 7.0 Hz), 118.34 (d, J = 84.2 Hz), 118.18–117.80 (m); ^{31}P NMR (162 MHz, CD_2Cl_2): δ 46.05; ^{19}F NMR (376 MHz, CD_2Cl_2): δ -62.77; HRMS (ESI+) exact mass calculated for $[M]^+$ ($C_{24}H_{20}PS$) requires m/z 371.1018, found m/z 371.1021.

Ch13 was prepared according to the general procedure. White solid (75% yield). ^{1}H NMR (400 MHz, CD_2Cl_2): δ 7.72–7.61 (m, 11H), 7.52–7.41 (m, 10H), 7.39–7.22 (m, 7H), 7.05 (s, 1H), 7.03 (s, 1H), 3.18–2.96 (m, 2H), 0.73 (bs, 12H); ^{13}C NMR (100 MHz, CD_2Cl_2): δ 162.32 (q, J = 49.6 Hz), 155.90 (d, J = 3.3 Hz), 136.51 (d, J = 3.4 Hz), 135.35 (bs), 134.48 (d, J = 10.6 Hz), 133.56 (d, J = 3.3 Hz), 130.97 (d, J = 13.3 Hz), 129.46 (qq, J = 31.3, 2.9 Hz), 126.25 (d, J = 2.8 Hz), 125.13 (q, J = 270.7 Hz), 121.52 (d, J = 7.5 Hz), 118.97 (d, J = 76.6 Hz), 118.22–117.81 (m), 35.84 (s), 23.74 (bs); ^{31}P NMR (162 MHz, CD_2Cl_2): δ 32.99; ^{77}Se NMR (76 MHz, CD_2Cl_2): δ 171.17 (d, $J_{Se\text{-}P}$ = 481.5 Hz); ^{19}F NMR (376 MHz, CD_2Cl_2): δ -62.79; HRMS (ESI+) exact mass calculated for $[M]^+$ ($C_{30}H_{32}PSe$)

requires m/z 503.1401, found m/z 503.1397.

Ch14 was prepared according to the general procedure. White solid (73% yield). ^{1}H NMR (400 MHz, CD_2Cl_2): δ 7.93–7.85 (m, 4H), 7.80–7.67 (m, 24H), 7.66–7.52 (m, 16H), 4.48 (t, *J* = 13.6 Hz, 2H), 1.98 (s, 3H), 1.94 (s, 3H); ^{13}C NMR (100 MHz, CD_2Cl_2): δ 162.22 (q, *J* = 49.0 Hz), 138.93–138.48 (m), 135.26 (bs), 133.83–133.33 (m), 132.64–132.07 (m), 129.34 (qq, *J* = 32.0, 3.0 Hz), 125.04 (q, *J* = 271.0 Hz), 118.16–117.79 (m), 114.17 (d, *J* = 76.0 Hz), 29.45 (t, *J* = 41.0 Hz), 10.32; ^{31}P NMR (162 MHz, CD_2Cl_2): δ 31.06; ^{77}Se NMR (76 MHz, CD_2Cl_2): δ 74.88 (dd, *J* = 477.3, 12.9 Hz); ^{19}F NMR (376 MHz, CD_2Cl_2): δ -62.69; HRMS (ESI+) exact mass calculated for $[M]^{2+}$ ($C_{27}H_{28}P_2Se_2$) requires m/z 286.9993, found m/z 286.9997.

XB was prepared according to the general procedure. White solid (87% yield). ^{1}H NMR (400 MHz, CD_2Cl_2): δ 7.96–7.70 (m, 19H), 7.67–7.54 (m, 12H), 7.42 (t, *J* = 2.1 Hz), 3.94 (s, 6H); ^{13}C NMR (100 MHz, CD_2Cl_2): δ 162.23 (q, *J* = 49.5 Hz), 137.52 (s), 135.28 (bs), 134.35 (s), 130.92 (s), 129.37 (qq, *J* = 31.3, 2.6 Hz), 128.28 (s), 127.47 (s), 125.06 (q, *J* = 270.8 Hz), 124.54 (s), 118.25–117.79 (m), 97.77 (s), 41.21 (s); ^{19}F NMR (376 MHz, CD_2Cl_2): δ -62.47; HRMS (ESI+) exact mass calculated for $[M]^{2+}$ ($C_{14}H_{14}I_2N_4$) requires m/z 245.9645, found m/z 245.9640.

3. Optimization of reaction condition

Supplementary Table 1. Evaluation of catalysts.

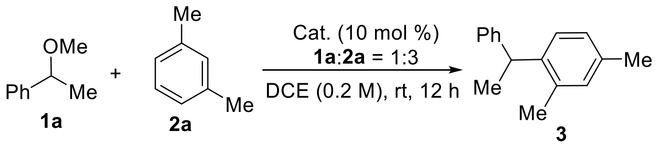

Entry	Cat.	Yield (%)
1	vacant	n.r.
2	**Ch1**	< 5%
3	**Ch2**	15%
4	**Ch3**	87%
5	**Ch5**	58%
6	**Ch6**	46%
7	**Ch7**	25%

Supplementary Table 2. Evaluation of solvent.

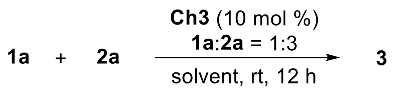

Entry	Solvent (0.2 M)	Yield (%)
1	DCM	75%
2	DCE	87%
3	CH_3CN	72%
4	toluene	< 5%

General procedure for optimization: To a reaction mixture of catalyst **Ch** (10 mol %) in a 10 mL-Schlenk tube was added freshly distilled dry solvent under argon atmosphere. Then **1a** (0.2 mmol) and **2a** (3.0 equiv) was added

to the above reaction mixture. The reaction was stirred at room temperature for the indicated reaction time. Then the solvent was removed under reduced pressure and the residue was purified by flash chromatography on silica gel to give the desired products.

4. Optimized Procedure

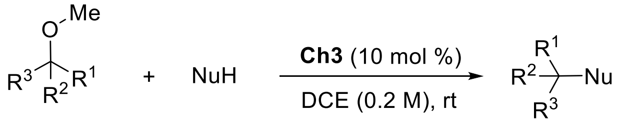

General Method: To a reaction mixture of catalyst **Ch3** (10 mol %) in a 10 mL-Schlenk tube was added DCE (1.0 mL) under argon atmosphere. Then ether **1** (0.2 mmol) and nucleophile (0.6 mmol, 3.0 equiv, without addition of nucleophile for **1u**, **1v**, **1w** and **1x**) was added to the above reaction mixture. The reaction was stirred at room temperature until the completion of the reaction as judged by TLC analysis. Then the solvent was removed under reduced pressure and the residue was purified by flash chromatography on silica gel to give the desired products.

5. Analytical Data

1,3,5-triisopropyl-2-(methoxymethyl)benzene (e4):

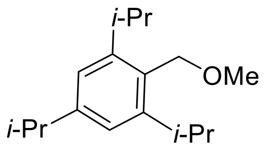

e4

Compound **e4** was synthesized according to the general method as a colorless oil in 93% yield by flash chromatography (petroleum ether/ethyl acetate, 100:1) on silica gel. **^{1}H NMR** (400 MHz, CD_2Cl_2): δ 7.09 (s, 2H), 4.52 (s, 2H), 3.45 (s, 3H), 3.41–3.29 (m, 2H), 3.01–2.85 (m, 1H), 1.31 (d, *J* = 7.0 Hz, 18H); **^{13}C NMR** (100 MHz, CD_2Cl_2): δ 149.34, 148.91, 130.10, 121.28, 67.28, 58.43, 34.93, 29.79, 24.70, 24.34; **HRMS** (EI) exact mass calculated for $[M]^+$ ($C_{17}H_{28}O$) requires m/z 248.2140, found m/z 248.2143.

2-((benzyloxy)methyl)-1,3,5-triisopropylbenzene (e5):

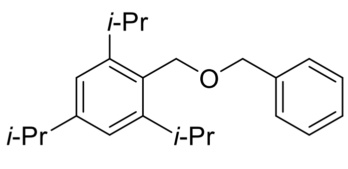

e5

Compound **e5** was synthesized according to the general method as a colorless oil in 90% yield by flash chromatography (petroleum ether/ethyl acetate, 100:1) on silica gel. **^{1}H NMR** (400 MHz, CD_2Cl_2): δ 7.54–7.34 (m, 5H), 7.15 (s, 2H), 4.71 (s, 2H), 4.69 (s, 2H), 3.45–3.31 (m, 2H), 3.05–2.92 (m, 2H), 1.40–1.30 (m, 18H); **^{13}C NMR** (100 MHz, CD_2Cl_2): δ 149.45, 149.03, 139.32, 129.99, 128.74, 128.37, 128.02, 121.36, 73.21, 65.05, 34.98, 29.80, 24.75, 24.40; **HRMS** (EI) exact mass calculated for $[M]^+$ ($C_{23}H_{32}O$) requires m/z 324.2453, found m/z 324.2450.

2,4-dimethyl-1-(1-phenylethyl)benzene (3):

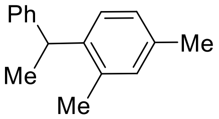

3

Compound **3** was synthesized according to the general method as a colorless oil using *m*-xylene as reactant (87% yield for **1a**; 73% yield for **1b**; 67% yield for **1c**) by flash chromatography (petroleum ether/ethyl acetate, 200:1) on silica gel. ^{1}**H NMR** (400 MHz, $CDCl_3$): δ 7.27–7.20 (m, 2H), 7.18–7.10 (m, 4H), 7.00 (d, *J* = 7.9 Hz, 1H), 6.95 (s, 1H), 4.27 (q, *J* = 7.2 Hz, 1H), 2.28 (s, 3H), 2.19 (s, 3H), 1.58 (d, *J* = 7.2 Hz, 3H); ^{13}**C NMR** (100 MHz, $CDCl_3$): δ 146.42, 140.94, 135.87, 135.46, 131.21, 128.26, 127.59, 126.60, 126.58, 125.71, 40.63, 22.15, 20.87 ,19.64; **HRMS** (EI) exact mass calculated for $[M]^+$ ($C_{16}H_{18}$) requires m/z 210.1409, found m/z 210.1403.

1-methoxy-4-(1-phenylethyl)benzene (5):

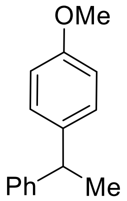

5

Compound **5** was synthesized according to the general method as a colorless oil using anisole as reactant (82% yield for **1a**; 88% yield for **1d**; 83% yield for **1e**; 81% yield for **1f**; 68% yield for **1g**) by flash chromatography (petroleum ether/ethyl acetate, 150:1) on silica gel. ^{1}**H NMR** (400 MHz, $CDCl_3$): δ 7.31–7.08 (m, 7H), 6.88–6.72 (m, 2H), 4.10 (q, *J* = 7.2 Hz, 1H), 3.76 (s, 3H), 1.61 (d, *J* = 7.2 Hz, 3H); ^{13}**C NMR** (100 MHz, $CDCl_3$): δ 157.79, 146.74, 138.52, 128.48, 128.31, 127.50, 125.90, 113.68, 55.20, 43.89, 22.03; **HRMS** (ESI+) exact mass calculated for $[M+H]^+$ ($C_{15}H_{17}O$) requires m/z 213.1274, found m/z 213.1272.

2-(4-methoxybenzyl)-1,3,5-trimethylbenzene (6):

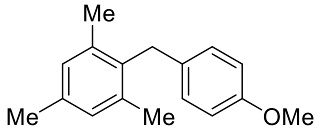

6

Compound **6** was synthesized according to the general method as a colorless oil in 77% yield by flash chromatography (petroleum ether/ethyl acetate, 150:1) on silica gel. ^{1}**H NMR** (400 MHz, $CDCl_3$): δ 6.96 (d, *J* = 8.6 Hz, 2H), 6.92 (s, 2H), 6.81 (d, *J* = 8.6 Hz, 2H), 3.99 (s, 2H), 3.79 (s, 3H), 2.33 (s, 3H) , 2.24 (s, 6H); ^{13}**C NMR** (100 MHz, $CDCl_3$): δ 157.62, 136.90, 135.52, 134.12, 132.02, 128.84, 128.68, 113.73, 55.18, 33.74, 20.87, 20.06; **HRMS** (ESI+) exact mass calculated for $[M+H]^+$ ($C_{17}H_{21}O$) requires m/z 241.1587, found m/z 241.1585.

1-butyl-4-(4-methoxybenzyl)benzene (7):

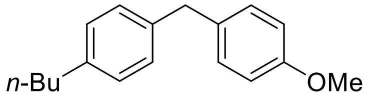

7

Compound **7** was synthesized according to the general method as a colorless oil in 74% yield by flash chromatography (petroleum ether/ethyl acetate, 150:1) on silica gel. ^{1}**H NMR** (400 MHz, $CDCl_3$): δ 7.16–7.06 (m, 6H), 6.85 (d, *J* = 8.7 Hz, 2H), 3.92 (s, 2H), 3.80 (s, 3H), 2.60 (t, *J* = 7.7 Hz, 2H), 1.66–1.56 (m, 2H), 1.41–1.33 (m, 2H), 0.95 (t, *J* = 7.3 Hz, 3H); ^{13}**C NMR** (100 MHz, $CDCl_3$): δ 157.87, 140.48, 138.70, 133.50, 129.80, 128.60, 128.43, 113.80,

55.20, 40.61, 35.21, 33.69, 22.37, 13.94; **HRMS** (ESI+) exact mass calculated for $[M+H]^+$ ($C_{18}H_{23}O$) requires m/z 255.1743, found m/z 255.1737.

1-(tert-butyl)-4-(4-methoxybenzyl)benzene (8):

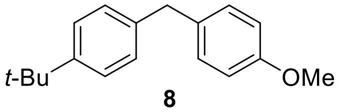

8

Compound **8** was synthesized according to the general method as a colorless oil in 64% yield by flash chromatography (petroleum ether/ethyl acetate, 150:1) on silica gel. **^{1}H NMR** (400 MHz, $CDCl_3$): δ 7.33 (d, *J* = 8.3 Hz, 2H), 7.21–7.07 (m, 4H), 6.86 (d, *J* = 8.6 Hz, 2H), 3.93 (s, 2H), 3.81 (s, 3H), 1.33 (s, 9H); **^{13}C NMR** (100 MHz, $CDCl_3$): δ 157.88, 148.70, 138.52, 133.40, 129.85, 128.35, 125.29, 113.81, 55.21, 40.48, 34.32, 31.38; **HRMS** (ESI+) exact mass calculated for $[M+H]^+$ ($C_{18}H_{23}O$) requires m/z 255.1743, found m/z 255.1743.

1-(4-(tert-butyl)benzyl)-2-methoxybenzene (8'):

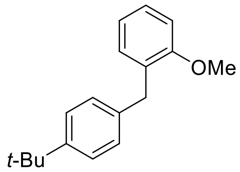

8'

Compound **8'** was synthesized according to the general method as a colorless oil in 14% yield by flash chromatography (petroleum ether/ethyl acetate, 150:1) on silica gel. **^{1}H NMR** (400 MHz, $CDCl_3$): δ 7.31–7.25 (m, 2H), 7.21–7.11 (m, 3H), 7.07 (d, *J* = 7.7 Hz, 1H), 6.91–6.81 (m, 2H), 3.94 (s, 2H), 3.82 (s, 3H), 1.29 (s, 9H); **^{13}C NMR** (100 MHz, $CDCl_3$): δ 157.29, 148.44, 137.87, 130.29, 129.81, 128.53, 127.27, 125.14, 120.42, 110.32, 55.32, 35.19, 34.31, 31.40; **HRMS** (ESI+) exact mass calculated for $[M+H]^+$ ($C_{18}H_{23}O$) requires m/z 255.1743, found m/z 255.1746.

1-(cyclohexyl(phenyl)methyl)-4-methoxybenzene (9):

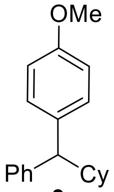

9

Compound **9** was synthesized according to the general method as a colorless oil in 73% yield by flash chromatography (petroleum ether/ethyl acetate, 150:1) on silica gel. **^{1}H NMR** (400 MHz, $CDCl_3$): δ 7.27–7.06 (m, 7H), 6.84–6.71 (m, 2H), 3.68 (s, 3H), 3.42 (d, *J* = 10.7 Hz, 1H), 2.14–1.96 (m, 1H), 1.73–1.51 (m, 4H), 1.33–1.02 (m, 2H), 0.93–0.73 (m, 2H); **^{13}C NMR** (100 MHz, $CDCl_3$): δ 157.67, 144.79, 136.60, 128.85, 128.30, 127.92, 125.73, 113.69, 58.59, 55.02, 41.32, 32.09, 32.03, 26.51, 26.32, 26.30; **HRMS** (ESI+) exact mass calculated for $[M+H]^+$ ($C_{20}H_{25}O$) requires m/z 281.1900, found m/z 281.1895.

1-(cyclopropyl(phenyl)methyl)-4-methoxybenzene (10):

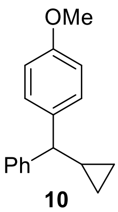

10

Compound **10** was synthesized according to the general method as a colorless oil in 68% yield by flash chromatography (petroleum ether/ethyl acetate, 150:1) on silica gel. **^{1}H NMR** (400 MHz, $CDCl_3$): δ 7.34–7.11 (m, 7H), 6.93–6.72 (m, 2H), 3.78 (s, 3H), 3.16 (d, *J* = 9.5 Hz, 1H), 1.40–1.29 (m, 1H), 0.69–0.59 (m, 2H), 0.34–0.22 (m, 2H); **^{13}C NMR** (100 MHz, $CDCl_3$): δ 157.85, 145.52,

137.38, 129.12, 128.17, 128.16, 125.99, 113.55, 55.18, 54.79, 16.88, 5.29, 5.24; **HRMS** (ESI+) exact mass calculated for $[M+H]^+$ ($C_{17}H_{19}O$) requires m/z 239.1430, found m/z 239.1426.

1-methoxy-4-(1-phenylbutyl)benzene (11):

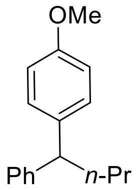

11

Compound **11** was synthesized according to the general method as a colorless oil in 75% yield by flash chromatography (petroleum ether/ethyl acetate, 150:1) on silica gel. **^{1}H NMR** (400 MHz, $CDCl_3$): δ 7.30–7.05 (m, 7H), 6.86–6.70 (m, 2H), 3.85 (t, J = 7.8 Hz, 1H), 3.71 (s, 3H), 2.05–1.88 (m, 2H), 1.32–1.19 (m, 2H), 0.90 (t, J = 7.1 Hz, 3H); **^{13}C NMR** (100 MHz, $CDCl_3$): δ 157.75, 145.69, 137.42, 128.68, 128.28, 127.71, 125.84, 113.67, 55.07, 50.13, 38.05, 21.12, 14.04; **HRMS** (ESI+) exact mass calculated for $[M+H]^+$ ($C_{17}H_{21}O$) requires m/z 241.1587, found m/z 241.1581.

((4-methoxyphenyl)methylene)dibenzene (12):

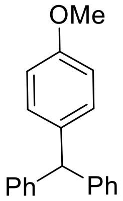

12

Compound **12** was synthesized according to the general method as a colorless oil in 77% yield by flash chromatography (petroleum ether/ethyl acetate, 150:1) on silica gel. **^{1}H NMR** (400 MHz, $CDCl_3$): δ 7.31–7.22 (m, 4H), 7.21–7.14 (m, 2H), 7.13–7.06 (m, 4H), 7.05–6.96 (m, 2H), 6.86–6.76 (m, 2H), 5.49 (s, 1H), 3.75 (s, 3H); **^{13}C NMR** (100 MHz, $CDCl_3$): δ 157.96, 144.20, 136.04, 130.33, 129.33, 128.24, 126.28, 113.61, 55.95, 55.15; **HRMS** (ESI+) exact mass calculated for $[M+H]^+$ ($C_{20}H_{19}O$) requires m/z 275.1430, found m/z 275.1427.

(1-(4-methoxyphenyl)ethane-1,2-diyl)dibenzene (13):

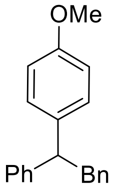

13

Compound **13** was synthesized according to the general method as a colorless oil in 68% yield by flash chromatography (petroleum ether/ethyl acetate, 200:1) on silica gel. **^{1}H NMR** (400 MHz, $CDCl_3$): δ 7.31–7.05 (m, 10H), 7.04–6.95 (m, 2H), 6.84–6.70 (m, 2H), 4.18 (t, J = 7.8 Hz, 1H), 3.73 (s, 3H) , 3.32 (d, J = 7.8 Hz, 2H); **^{13}C NMR** (100 MHz, $CDCl_3$): δ 157.84, 144.84, 140.33, 136.55, 129.05, 128.91, 128.28, 128.00, 127.00, 126.05, 125.80, 113.64, 55.13, 52.19, 42.25; **HRMS** (ESI+) exact mass calculated for $[M+H]^+$ ($C_{21}H_{21}O$) requires m/z 289.1587, found m/z 289.1587.

1-(4-methoxyphenyl)-2,3-dihydro-1H-indene (14):

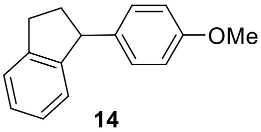

14

Compound **14** was synthesized according to the general method as a white solid in 64% yield by flash chromatography (petroleum ether/ethyl acetate, 150:1) on silica gel. **^{1}H NMR** (400 MHz, $CDCl_3$): δ 7.31–7.05 (m, 5H), 6.97–6.78 (m, 3H), 4.27 (t, J = 8.2 Hz, 1H), 3.77 (s, 3H), 3.07–2.85 (m, 2H), 2.60–2.48 (m, 2H), 2.07–1.92 (m, 1H); **^{13}C NMR** (100 MHz, $CDCl_3$): δ

158.06, 147.11, 144.16, 137.41, 128.97, 126.40, 126.27, 124.78, 124.26, 113.79, 55.16, 50.78, 36.69, 31.71; **HRMS** (APCI) exact mass calculated for $[M-H]^+$ ($C_{16}H_{15}O$) requires m/z 223.1118, found m/z 223.1115.

1-(4-methoxyphenyl)-1,2,3,4-tetrahydronaphthalene (15):

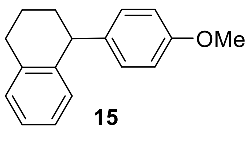

15

Compound **15** was synthesized according to the general method as a colorless oil in 62% yield by flash chromatography (petroleum ether/ethyl acetate, 150:1) on silica gel. ^{1}**H NMR** (400 MHz, $CDCl_3$): δ 7.15–6.96 (m, 5H), 6.87–6.76 (m, 3H), 4.06 (t, *J* = 6.7 Hz, 1H), 3.78 (s, 3H), 2.96–2.74 (m, 2H), 2.17–2.06 (m, 1H), 1.93–1.66 (m, 3H); ^{13}**C NMR** (100 MHz, $CDCl_3$): δ 157.77, 139.70, 139.63, 137.49, 130.09, 129.66, 128.90, 125.79, 125.57, 113.57, 51.19, 44.71, 33.30, 29.75, 20.92; **HRMS** (APCI) exact mass calculated for $[M-H]^+$ ($C_{17}H_{17}O$) requires m/z 237.1275, found m/z 237.1270.

(E)-1-methoxy-4-(4-phenylbut-3-en-2-yl)benzene (16):

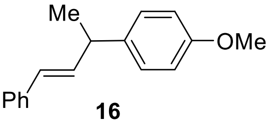

16

Compound **16** was synthesized according to the general method as a colorless oil in 68% yield by flash chromatography (petroleum ether/ethyl acetate, 100:1) on silica gel. ^{1}**H NMR** (400 MHz, $CDCl_3$): δ 7.37–7.31 (m, 2H), 7.30–7.24 (m, 2H), 7.21–7.13 (m, 3H), 6.90–6.80 (m, 2H), 6.41–6.31 (m, 2H), 3.78 (s, 3H), 3.64–3.54 (m, 1H), 1.43 (d, *J* = 7.0 Hz, 3H); ^{13}**C NMR** (100 MHz, $CDCl_3$): δ 157.99, 137.68, 137.60, 135.57, 128.45, 128.22, 128.19, 126.96, 126.10, 113.84, 55.24, 41.67, 21.29; **HRMS** (ESI+) exact mass calculated for $[M+H]^+$ ($C_{17}H_{19}O$) requires m/z 239.1430, found m/z 239.1433.

1-cinnamyl-4-methoxybenzene (17):

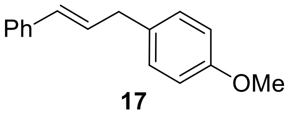

17

Compound **17** was synthesized according to the general method as a colorless oil in 69% yield by flash chromatography (petroleum ether/ethyl acetate, 150:1) on silica gel. ^{1}**H NMR** (400 MHz, $CDCl_3$): δ 7.38–7.30 (m, 2H),7.29–7.22 (m, 2H), 7.20–7.07 (m, 3H), 6.88–6.76 (m, 2H), 6.45–6.26 (m, 2H), 3.75 (s, 3H), 3.46 (d, *J* = 6.5 Hz, 2H); ^{13}**C NMR** (100 MHz, $CDCl_3$): δ 158.00, 137.45, 132.08, 130.66, 129.60, 129.54, 128.43, 126.99, 126.04, 113.83, 55.17, 38.39; **HRMS** (ESI+) exact mass calculated for $[M+H]^+$ ($C_{16}H_{17}O$) requires m/z 225.1274, found m/z 225.1275.

(*E*)-(((4-cyclohexylbut-3-en-2-yl)oxy)methyl)benzene (18):

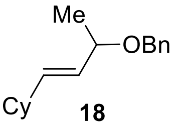

18

Compound **18** was synthesized according to the general method as a colorless oil in 74% yield by flash chromatography (petroleum ether/ethyl acetate, 100:1) on silica gel. ^{1}**H NMR** (400 MHz, $CDCl_3$): δ 7.40–7.28 (m, 4H), 7.27–7.21 (m, 1H), 5.55 (dd, *J* = 15.6, 6.6 Hz, 1H), 5.32 (ddd, *J*

= 15.6, 7.9, 1.3 Hz, 1H), 4.54 (d, *J* = 11.9 Hz, 1H), 4.35 (d, *J* = 11.9 Hz, 1H), 3.94–3.76 (m, 1H), 2.05–1.88 (m, 1H), 1.81–1.56 (m, 5H), 1.35–0.98 (m, 8H); **^{13}C NMR** (100 MHz, $CDCl_3$): δ 139.23, 139.01, 129.10, 128.25, 127.67, 127.25, 75.92, 69.45, 40.25, 32.93, 32.87, 26.14, 25.98, 21.82; **HRMS** (EI) exact mass calculated for $[M]^+$ ($C_{17}H_{24}O$) requires m/z 244.1827, found m/z 244.1822.

4-(1-phenylethyl)phenol (19):

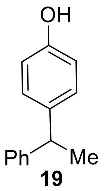

19

Compound **19** was synthesized according to the general method as a colorless oil in 67% yield by flash chromatography (petroleum ether/acetone, 25:1) on silica gel. **^{1}H NMR** (400 MHz, $CDCl_3$): δ 7.31–7.23 (m, 2H), 7.22–7.12 (m, 3H), 7.11–7.02 (m, 2H), 6.80–6.67 (m, 2H), 4.61 (s, 1H), 4.08 (q, *J* = 7.2 Hz, 1H), 1.59 (d, *J* = 7.2 Hz, 3H); **^{13}C NMR** (100 MHz, $CDCl_3$): δ 153.61, 146.69, 138.74, 128.71, 128.32, 127.50, 125.93, 115.10, 43.90, 22.03; **HRMS** (EI) exact mass calculated for $[M]^+$ ($C_{14}H_{14}O$) requires m/z 198.1045, found m/z 198.1039.

2-(1-phenylethyl)phenol (19'):

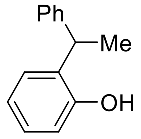

19'

Compound **19'** was synthesized according to the general method as a colorless oil in 16% yield by flash chromatography (petroleum ether/acetone, 25:1) on silica gel. **^{1}H NMR** (400 MHz, $CDCl_3$): δ 7.32–7.14 (m, 6H), 7.10 (t, *J* = 7.5 Hz, 1H), 6.93 (t, *J* = 7.5 Hz, 1H), 6.77 (d, *J* = 7.9 Hz, 1H), 4.66 (s, 1H), 4.36 (q, *J* = 7.2 Hz, 1H), 1.62 (d, *J* = 7.2 Hz, 3H); **^{13}C NMR** (100 MHz, $CDCl_3$): δ 153.20, 145.28, 131.90, 128.65, 127.90, 127.48, 126.41, 120.87, 115.92, 38.66, 20.98; **HRMS** (EI) exact mass calculated for $[M]^+$ ($C_{14}H_{14}O$) requires m/z 198.1045, found m/z 198.1041.

decyl(1-phenylethyl)sulfane (20):

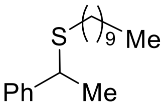

20

Compound **20** was synthesized according to the general method as a colorless oil in 56% yield by flash chromatography (petroleum ether/ethyl acetate, 150:1) on silica gel. **^{1}H NMR** (400 MHz, $CDCl_3$): δ 7.37–7.18 (m, 2H), 3.93 (q, *J* = 7.1 Hz, 1H), 2.40–2.19 (m, 2H), 1.56 (d, *J* = 7.1 Hz, 3H), 1.53–1.41 (m, 2H), 1.39–1.10 (m, 14H), 0.88 (t, *J* = 6.9 Hz, 3H); **^{13}C NMR** (100 MHz, $CDCl_3$): δ 144.22, 128.39, 127.21, 126.91, 44.02, 31.88, 31.28, 29.52, 29.46, 29.34, 29.29, 29.17, 28.91, 22.66, 22.60; **HRMS** (APCI) exact mass calculated for $[M+H]^+$ ($C_{18}H_{31}S$) requires m/z 279.2168, found m/z 279.2169.

2-(1-phenylethyl)furan (21):

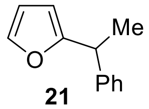

21

Compound **21** was synthesized according to the general method as a colorless oil in 56% yield by flash chromatography (petroleum ether/ethyl acetate, 200:1) on silica gel. **^{1}H NMR** (400

MHz, $CDCl_3$): δ 7.34─7.26 (m, 3H), 7.24─7.17 (m, 3H), 6.29 (dd, *J* = 1.8, 1.8 Hz, 1H), 6.05 (d, *J* = 3.2 Hz, 1H), 4.12 (q, *J* = 7.2 Hz, 1H), 1.59 (d, *J* = 7.2 Hz, 3H); **^{13}C NMR** (100 MHz, $CDCl_3$): δ 158.95, 144.14, 141.31, 128.46, 127.29, 126.49, 109.91, 104.89, 39.21, 20.50; **HRMS** (APCI) exact mass calculated for $[M+H]^+$ ($C_{12}H_{13}O$) requires m/z 173.0961, found m/z 173.0956.

3-(1-phenylethyl)pentane-2,4-dione (22):

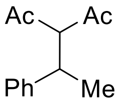

22

Compound **22** was synthesized according to the general method as a colorless oil in 79% yield by flash chromatography (petroleum ether/ethyl acetate, 20:1) on silica gel. **^{1}H NMR** (400 MHz, $CDCl_3$): δ 7.33─7.24 (m, 2H), 7.23─7.13 (m, 3H), 4.04 (d, *J* = 11.3 Hz, 1H), 3.67─3.52 (m, 1H), 2.27 (s, 3H), 1.84 (s, 3H), 1.21 (d, *J* = 6.8 Hz, 3H); **^{13}C NMR** (100 MHz, $CDCl_3$): δ 203.51, 203.46, 142.98, 128.79, 127.24, 126.96, 40.42, 29.79, 29.68, 20.84; **HRMS** (ESI+) exact mass calculated for $[M+Na]^+$ ($C_{13}H_{16}NaO_2$) requires m/z 227.1048, found m/z 227.1051.

(*E*)-pent-2-en-3-ylbenzene ((*E*)-23):

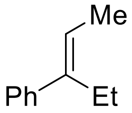

(*E*)-**23**

Compound (*E*)-**23** was synthesized according to the general method (without addition of nucleophile) as a colorless oil (76% yield, *E/Z* = 14.5:1) by flash chromatography (petroleum ether) on silica gel. **^{1}H NMR** (400 MHz, $CDCl_3$): δ 7.42─7.08 (m, 5H), 5.72 (q, *J* = 6.8 Hz, 1H), 2.51 (q, *J* = 7.5 Hz, 2H), 1.79 (d, *J* = 6.9 Hz, 3H), 0.98 (t, *J* = 7.5 Hz, 3H); **^{13}C NMR** (100 MHz, $CDCl_3$): δ 143.11, 142.33, 128.12, 126.35, 126.14, 122.03, 22.59, 13.92, 13.22; **HRMS** (EI) exact mass calculated for $[M]^+$ ($C_{11}H_{14}$) requires m/z 146.1096, found m/z 146.1090.

(*S*)-1,1-diphenyltetrahydro-1H,3H-pyrrolo[1,2-c]oxazol-3-one (24):

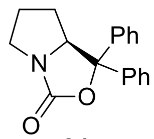

24

Compound **24** was synthesized according to the general method (without addition of nucleophile) as a white solid in 71% yield by flash chromatography (petroleum ether/ethyl acetate, 4:1) on silica gel. **^{1}H NMR** (400 MHz, $CDCl_3$): δ 7.57─7.46 (m, 2H), 7.43─7.21 (m, 8H), 4.54 (dd, *J* = 5.5, 5.5 Hz, 1H), 3.71 (td, *J* = 11.4, 8.1 Hz, 1H), 3.23 (ddd, *J* = 11.4, 9.4, 3.7 Hz, 1H), 2.02─1.64 (m, 3H), 1.20─1.02 (m, 1H); **^{13}C NMR** (100 MHz, $CDCl_3$): δ 160.36, 143.22, 140.19, 128.48, 128.23, 128.21, 127.61, 125.86, 125.38, 85.78, 69.14, 45.94, 28.91, 24.80; **HRMS** (APCI) exact mass calculated for $[M+H]^+$ ($C_{18}H_{18}NO_2$) requires m/z 280.1332, found m/z 280.1326.

1,3,3-trimethyl-1-(naphthalen-2-yl)-2,3-dihydro-1H-cyclopenta[a]naphthalene (25):

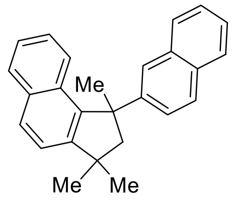

25

Compound **25** was synthesized according to the general method (without addition of nucleophile) as a colorless oil in 73% yield by flash chromatography (petroleum ether/ethyl acetate, 150:1) on silica gel. **^{1}H NMR** (400 MHz, $CDCl_3$): δ 7.93–7.69 (m, 5H), 7.64 (d, J = 8.6 Hz, 2H), 7.52–7.33 (m, 4H), 7.31–7.25 (m, 1H), 7.23–7.17 (m, 1H), 7.13–7.03 (m, 1H), 2.46 (d, J = 13.4 Hz, 2H), 2.35 (d, J = 13.4 Hz, 2H), 1.44 (s, 3H), 1.40 (s, 3H); **^{13}C NMR** (100 MHz, $CDCl_3$): δ 149.45, 148.62, 142.70, 133.90, 133.31, 131.70, 129.75, 128.83, 128.71, 128.11, 127.97, 127.45, 126.30, 125.85, 125.58, 125.30, 125.04, 124.49, 123.52, 121.30, 61.36, 52.15, 43.34, 31.40, 31.26, 28.08; **HRMS** (EI) exact mass calculated for $[M]^+$ ($C_{26}H_{24}$) requires m/z 336.1878, found m/z 336.1875.

2-(2,4-dimethylbenzyl)-1,3,5-triisopropylbenzene (26):

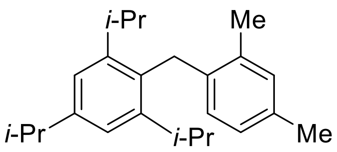

26

Compound **26** was synthesized according to the general method using **e5** and *m*-xylene as reactants in 56% yield by flash chromatography (petroleum ether) on silica gel. **^{1}H NMR** (400 MHz, $CDCl_3$): δ 7.05 (s, 2H), 7.00 (s, 1H), 6.80 (d, J = 7.7 Hz, 1H), 6.50 (d, J = 7.8 Hz, 1H), 3.90 (s, 2H), 2.99–2.80 (m, 3H), 2.39 (s, 3H), 2.26 (s, 3H), 1.29 (d, J = 7.0 Hz, 6H), 1.14 (d, J = 6.9 Hz, 12H); **^{13}C NMR** (100 MHz, $CDCl_3$): δ 147.45, 146.82, 136.34, 135.43, 134.93, 130.80, 130.45, 127.21, 126.43, 120.94, 34.17, 30.13, 29.56, 24.23, 24.13, 20.86, 19.73; **HRMS** (EI) exact mass calculated for $[M]^+$ ($C_{24}H_{34}$) requires m/z 322.2661, found m/z 322.2664.

6. Mechanistic study

(1) The interaction between **Ch1-7** and (methoxymethyl)benzene (**e1**)

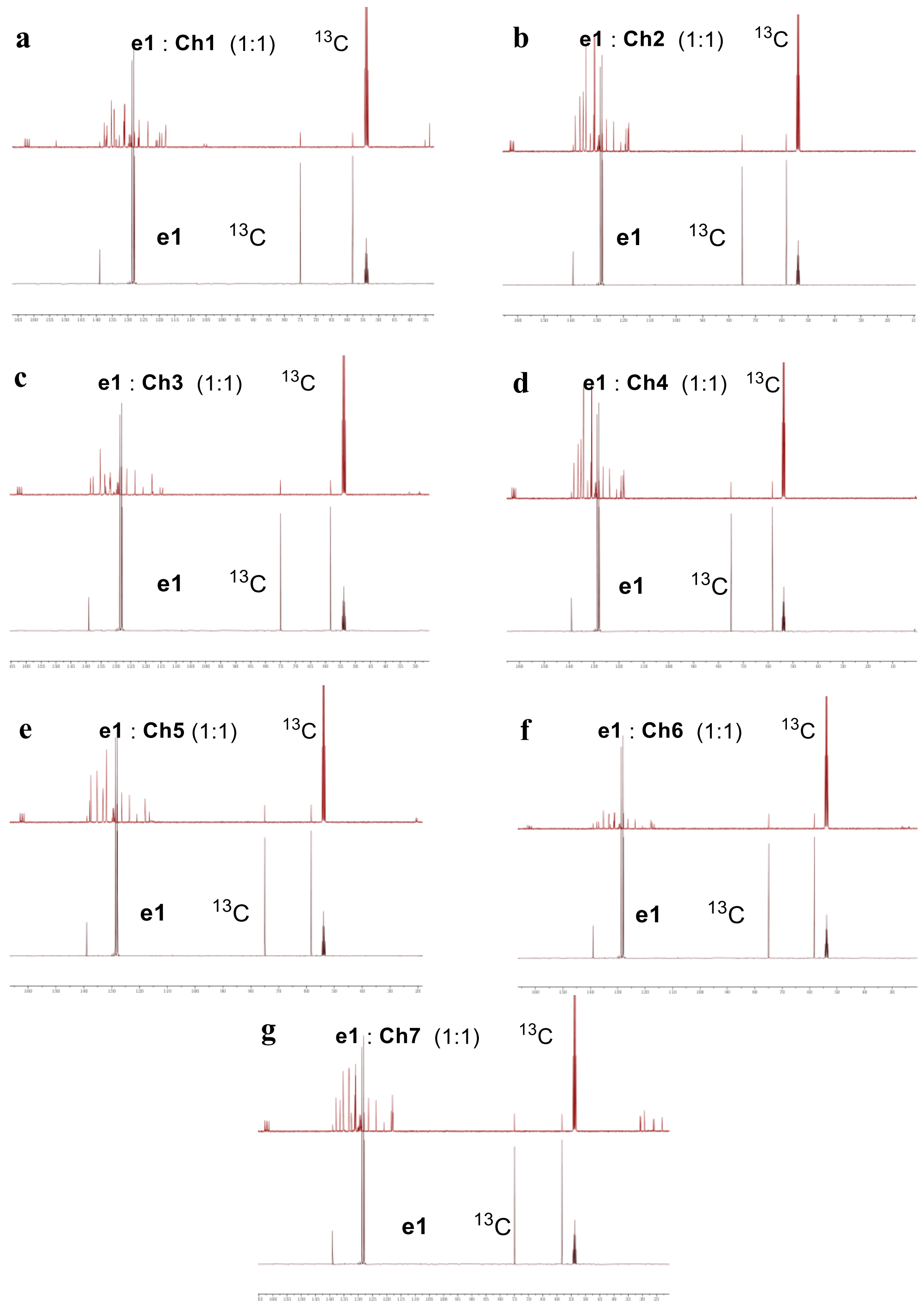

Supplementary Fig. 2 The interaction between Ch1-7 and e1 (^{13}C NMR in CD_2Cl_2, 100 MHz, 298K). a The ^{13}C NMR spectrum of **e1** upon addition of **Ch1**. **b** The ^{13}C NMR spectrum of **e1** upon addition of **Ch2**. **c** The ^{13}C NMR spectrum of **e1** upon addition of **Ch3**. **d** The ^{13}C NMR spectrum of **e1** upon addition of **Ch4**. **e** The ^{13}C NMR spectrum of **e1** upon addition of **Ch5**. **f** The ^{13}C NMR spectrum of **e1** upon addition of **Ch6**. **g** The ^{13}C NMR spectrum of **e1** upon addition of **Ch7**.

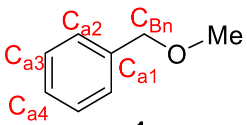

e1

To a mixture of catalyst **Ch** (0.05 mmol for bidentate **Ch1**, **Ch3**, **Ch5**, **Ch6** and **Ch7**; 0.1 mmol for monodentate **Ch2** and **Ch4**) and **e1** (0.05 mmol, 6.2 µL) in an NMR tube was added CD_2Cl_2 (0.5 mL) and then analysis of the reaction mixture by ^{13}C NMR experiments.

Supplementary Table 3. The shift of ^{13}C NMR spectrum of e1 upon addition of Ch1-7.

	C_{a1} (ppm)	C_{a2} (ppm)	C_{a3} (ppm)	C_{a4} (ppm)	C_{Bn} (ppm)
Only **e1**	139.03	128.06	128.70	127.91	74.95
**e1 : Ch1** (1:1)	139.04	128.06	128.69	127.90	74.96
**e1 : Ch2** (1:1)	139.02	128.08	128.70	127.92	74.97
**e1 : Ch3** (1:1)	138.55	128.19	128.78	128.12	75.07
**e1 : Ch4** (1:1)	138.99	128.09	128.71	127.93	74.97
**e1 : Ch5** (1:1)	138.94	128.09	128.70	127.94	74.96
**e1 : Ch6** (1:1)	138.98	128.05	128.67	127.89	74.94
**e1 : Ch7** (1:1)	139.00	128.08	128.70	127.92	74.97

Supplementary Table 4. The Δδ ^{13}C NMR spectrum of e1 upon addition of Ch1-7.

	Δδ C_{a1} (ppm)	Δδ C_{a2} (ppm)	Δδ C_{a3} (ppm)	Δδ C_{a4} (ppm)	Δδ C_{Bn} (ppm)
**e1 : Ch1** (1:1)	0.01	0	- 0.01	- 0.01	0.01
**e1 : Ch2** (1:1)	- 0.01	0.02	0	0.01	0.02
**e1 : Ch3** (1:1)	- 0.48	0.13	0.08	0.21	0.12
**e1 : Ch4** (1:1)	- 0.04	0.03	0.01	0.02	0.02
**e1 : Ch5** (1:1)	- 0.09	0.03	0	0.03	0.01
**e1 : Ch6** (1:1)	- 0.05	- 0.01	- 0.03	-0.02	- 0.01
**e1 : Ch7** (1:1)	- 0.03	0.02	0	0.01	0.02

(2) The interaction between **Ch3** and ethers (2-methoxyethyl)benzene (**e2**) or (3-methoxypropyl)benzene (**e3**)

To a mixture of catalyst **Ch3** (0.05 mmol) and **e2-e3** (0.05 mmol) in an NMR tube was added CD_2Cl_2 (0.5 mL) and then analysis of the reaction mixture by ^{13}C NMR experiments.

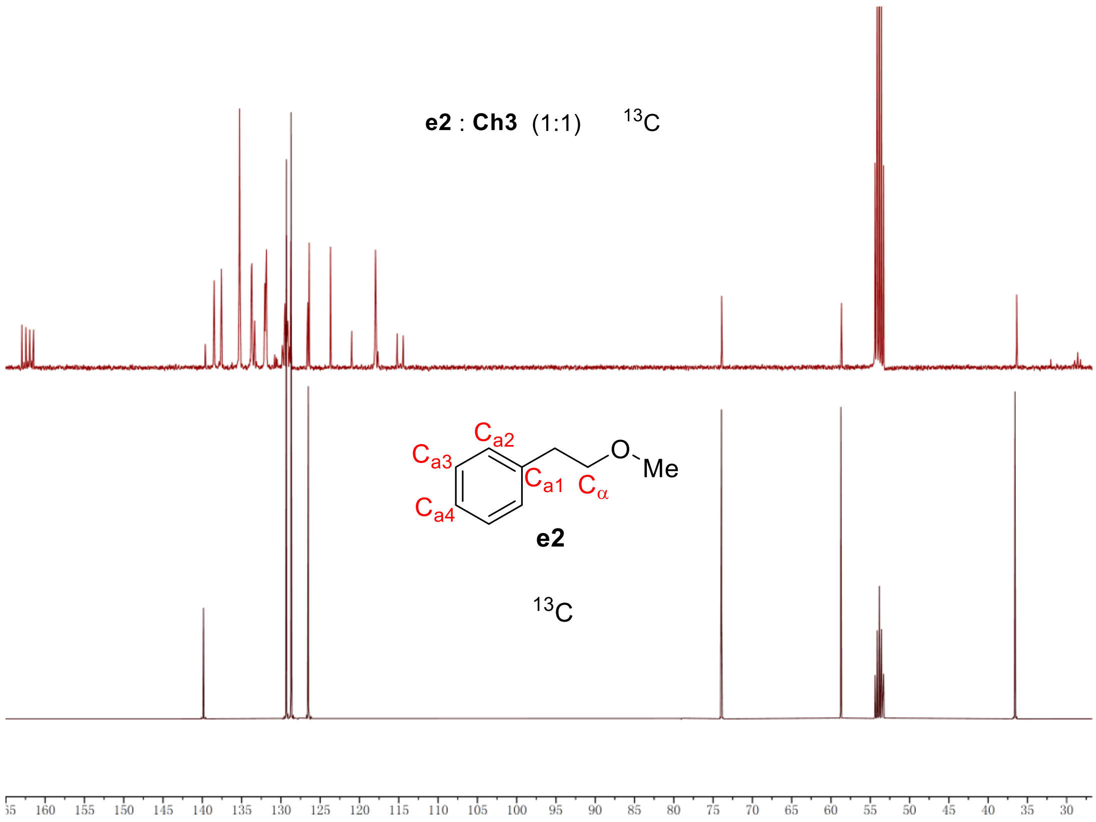

Supplementary Fig. 3 The interaction between Ch3 and e2 (^{13}C NMR in CD_2Cl_2, 100 MHz, 298K). The ^{13}C NMR spectrum of **e2** upon addition of **Ch3**.

Supplementary Table 5. The Δδ ^{13}C NMR spectrum of e2 upon addition of Ch3.

	C_{a1} (ppm)	C_{a2} (ppm)	C_{a3} (ppm)	C_{a4} (ppm)	C_{α} (ppm)
Only **e2**	139.85	128.70	129.30	126.51	73.93
**e2 : Ch3** (1:1)	139.62	128.74	129.27	126.59	73.91
△δ	- 0.23	0.04	- 0.03	0.08	- 0.02

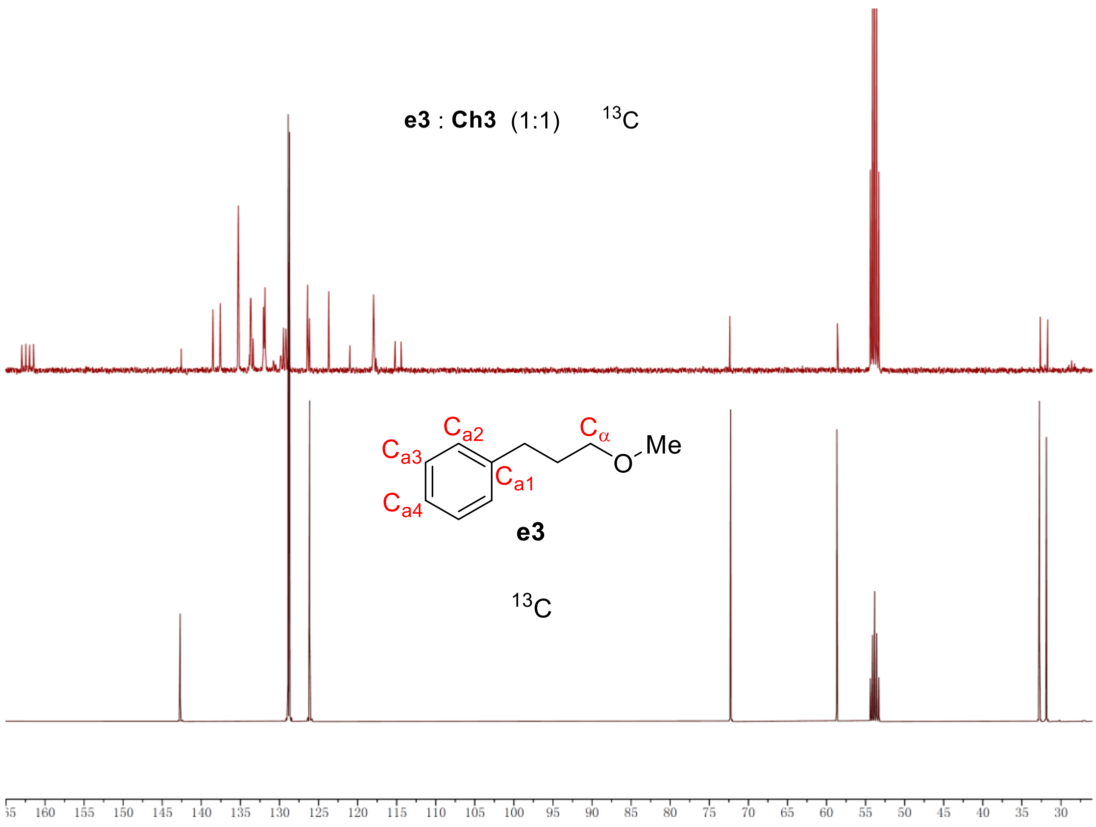

Supplementary Fig. 4 The interaction between Ch3 and e3 (^{13}C NMR in CD_2Cl_2, 100 MHz, 298K). The ^{13}C NMR spectrum of **e3** upon addition of **Ch3**.

Supplementary Table 6. The Δδ ^{13}C NMR spectrum of e3 upon addition of Ch3.

	**C$_{a1}$** (ppm)	**C$_{a2}$** (ppm)	**C$_{a3}$** (ppm)	**C$_{a4}$** (ppm)	**C$_{\alpha}$** (ppm)
Only **e3**	142.70	128.71	128.89	126.14	72.27
**e3 : Ch3** (1:1)	142.56	128.71	128.84	126.17	72.27
△δ	- 0.14	0	- 0.05	0.03	0.09

(3) The interaction between **Ch3** and ethers **e4-e6**

To a mixture of catalyst **Ch3** (0.05 mmol) and **e4-e6** (0.05 mmol) in an NMR tube was added CD_2Cl_2 (0.5 mL) and then analysis of the reaction mixture by ^{13}C NMR experiments.

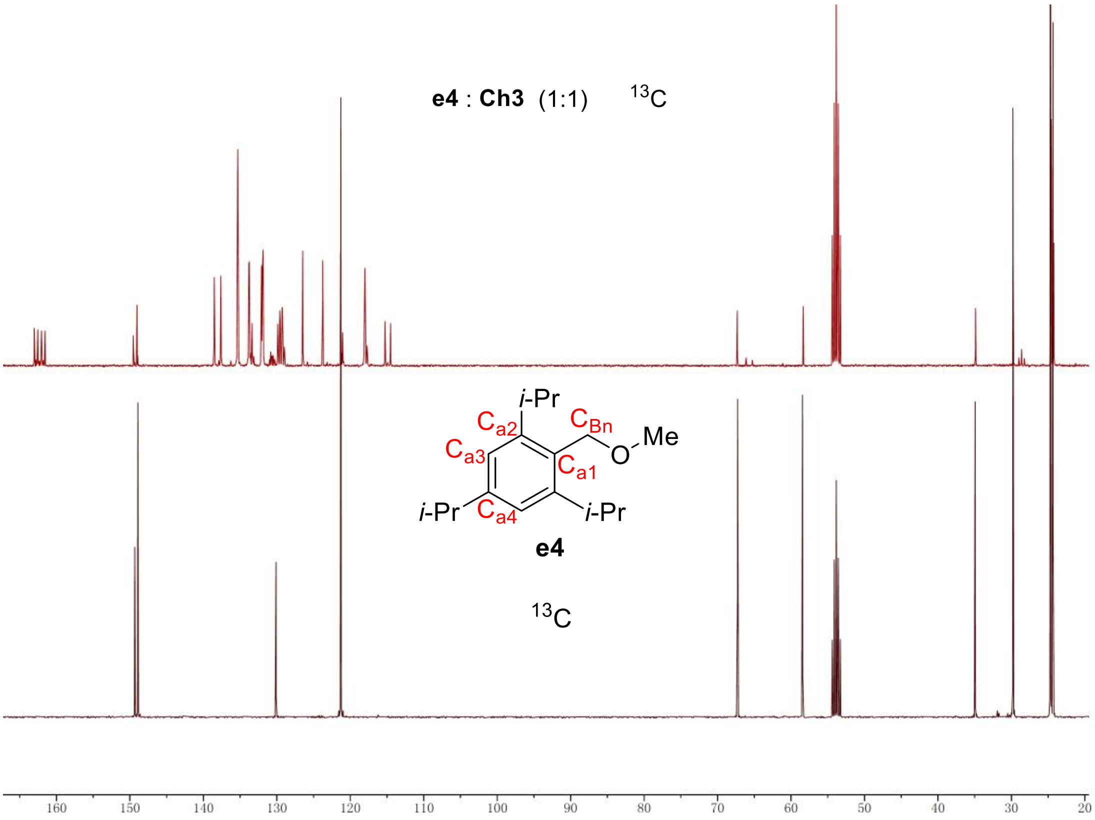

Supplementary Fig. 5 The interaction between Ch3 and e4 (^{13}C NMR in CD_2Cl_2, 100 MHz, 298K). The ^{13}C NMR spectrum of **e4** upon addition of **Ch3**.

Supplementary Table 7. The Δδ ^{13}C NMR spectrum of e4 upon addition of Ch3.

	**C_{a1}** (ppm)	**C_{a2}** (ppm)	**C_{a3}** (ppm)	**C_{a4}** (ppm)	**C_{Bn}** (ppm)
Only **e4**	130.10	148.91	121.28	149.34	67.28
**e4 : Ch3** (1:1)	129.86	149.03	121.31	149.63	67.31
△δ	- 0.24	0.12	0.03	0.19	0.03

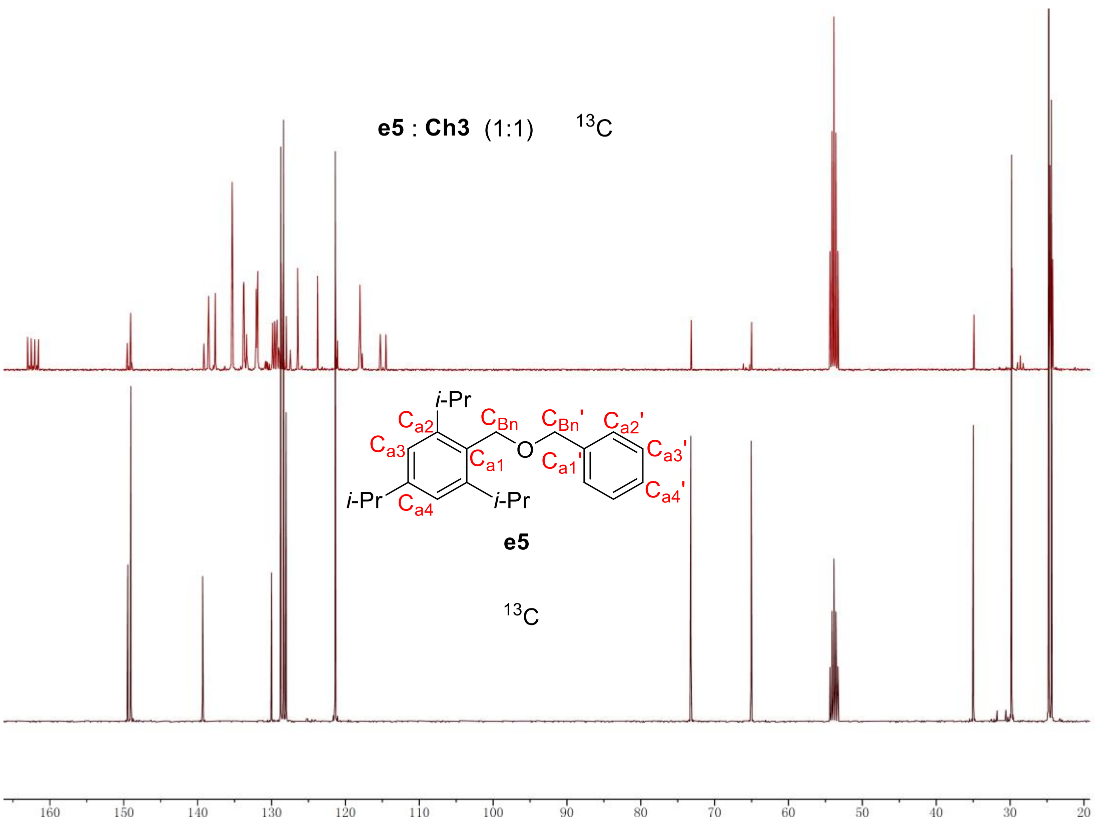

Supplementary Fig. 6 The interaction between Ch3 and e5 (^{13}C NMR in CD_2Cl_2, 100 MHz, 298K). The ^{13}C NMR spectrum of **e5** upon addition of **Ch3**.

Supplementary Table 8. The Δδ ^{13}C NMR spectrum of e5 upon addition of Ch3.

	**C_{a1}** (ppm)	**C_{a2}** (ppm)	**C_{a3}** (ppm)	**C_{a4}** (ppm)	**C_{Bn}** (ppm)
Only **e5**	128.02	149.03	121.36	149.45	65.05
**e5 : Ch3** (1:1)	127.99	149.06	121.33	149.51	64.99
△δ	- 0.03	0.03	- 0.03	0.06	- 0.06
	**C_{a1}'** (ppm)	**C_{a2}'** (ppm)	**C_{a3}'** (ppm)	**C_{a4}'** (ppm)	**C_{Bn}'** (ppm)
Only **e5**	139.32	128.37	128.74	129.99	73.21
**e5 : Ch3** (1:1)	139.16	128.37	128.69	129.84	73.16
△δ	- 0.16	0	- 0.05	- 0.15	- 0.05

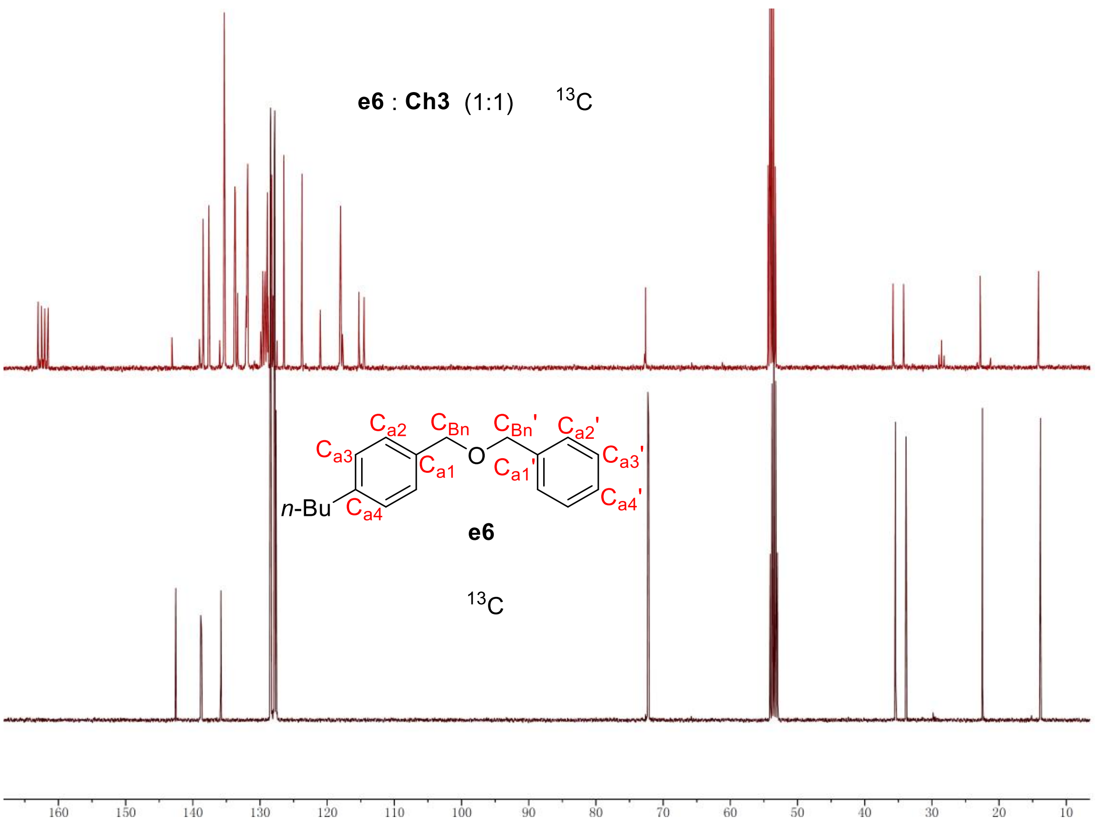

Supplementary Fig. 7 The interaction between Ch3 and e6 (^{13}C NMR in CD_2Cl_2, 100 MHz, 298K). The ^{13}C NMR spectrum of **e6** upon addition of **Ch3**.

Supplementary Table 9. The Δδ ^{13}C NMR spectrum of e6 upon addition of Ch3.

	**C$_{a1}$** (ppm)	**C$_{a2}$** (ppm)	**C$_{a3}$** (ppm)	**C$_{a4}$** (ppm)	**C$_{Bn}$** (ppm)
Only **e6**	136.16	128.11	128.73	142.87	72.50
**e6 : Ch3** (1:1)	135.99	128.25	128.82	143.11	72.61
△δ	- 0.17	0.14	0.09	0.24	0.11
	**C$_{a1}$’** (ppm)	**C$_{a2}$’** (ppm)	**C$_{a3}$’** (ppm)	**C$_{a4}$’** (ppm)	**C$_{Bn}$’** (ppm)
Only **e6**	139.16	128.23	128.80	127.90	72.56
**e6 : Ch3** (1:1)	139.01	128.37	128.91	128.05	72.69
△δ	- 0.15	0.14	0.11	0.15	0.13

(4) ^{77}Se NMR studies on the interactions between **Ch3** and **e1**

All experiments were conducted by mixing different ratios of the **Ch3** and **e1** at ambient temperature (298.5 K) in NMR tubes. Catalyst **Ch3** (1.0 equiv, 0.05 mmol) and the different equivalent of **e1** (0 equiv, 1.0 equiv, 2.0 equiv, 3.0 equiv, 6.0 equiv, 10.0 equiv, 15.0 equiv, 20.0 equiv) were dissolved in 0.5 mL CD_2Cl_2. Then the mixtures were analyzed by ^{77}Se NMR experiments.

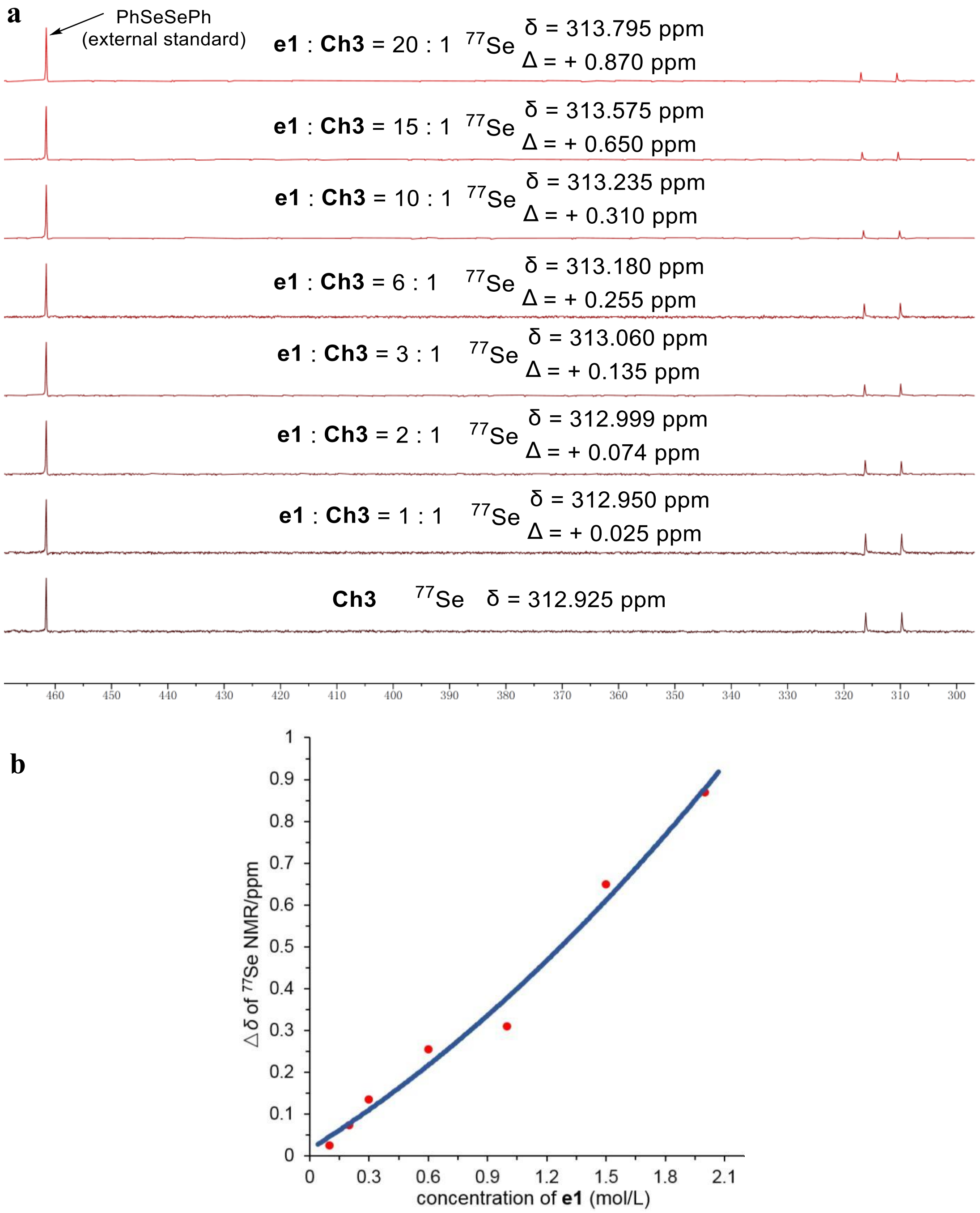

Supplementary Fig. 8 The interaction between Ch3 and e1 (^{77}Se NMR in CD_2Cl_2, 76 MHz, 298K). a The Δδ ^{77}Se NMR spectrum of **Ch3** upon addition of **e1**. **b** The relationship between the Δδ ^{77}Se NMR spectrum of **Ch3** and the concentration of **e1**.

Supplementary Table 10. The Δδ ^{77}Se NMR spectrum of Ch3 upon addition of e1.

**e1** (mol/L)	Shift of ^{77}Se (ppm)	△δ (ppm)
only **e1**	312.925	0
0.1 M	312.950	+ 0.025
0.2 M	312.999	+ 0.074
0.3 M	313.060	+ 0.135
0.6 M	313.180	+ 0.255
1.0 M	313.235	+ 0.310
1.5 M	313.575	+ 0.650
2.0 M	313.795	+ 0.870

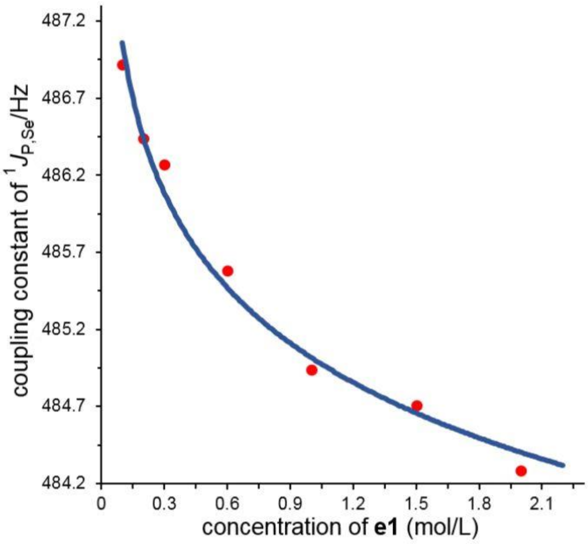

Supplementary Fig. 9 The interaction between Ch3 and e1 (^{77}Se NMR in CD_2Cl_2, 76 MHz, 298K). The relationship between the coupling constant of $^1J_{P,Se}$ of **Ch3** and the concentration of **e1**.

Supplementary Table 11. The $^1J_{p,Se}$ of ^{77}Se NMR spectrum of Ch3 upon addition of e1.

**e1** (mol/L)	$^1J_{p,Se}$ of ^{77}**Se** NMR spectrum (Hz)
only **e1**	486.491
0.1 M	486.917
0.2 M	486.438
0.3 M	486.271
0.6 M	485.579
1.0 M	484.941
1.5 M	484.705
2.0 M	484.287

(5) The interaction between **Ch3** and ether **1c**

To a mixture of catalyst **Ch3** (0.05 mmol) and **1c** (0.05 mmol) in an NMR tube was added CD_2Cl_2 (0.5 mL) and then analysis of the reaction mixture by ^{13}C NMR experiments.

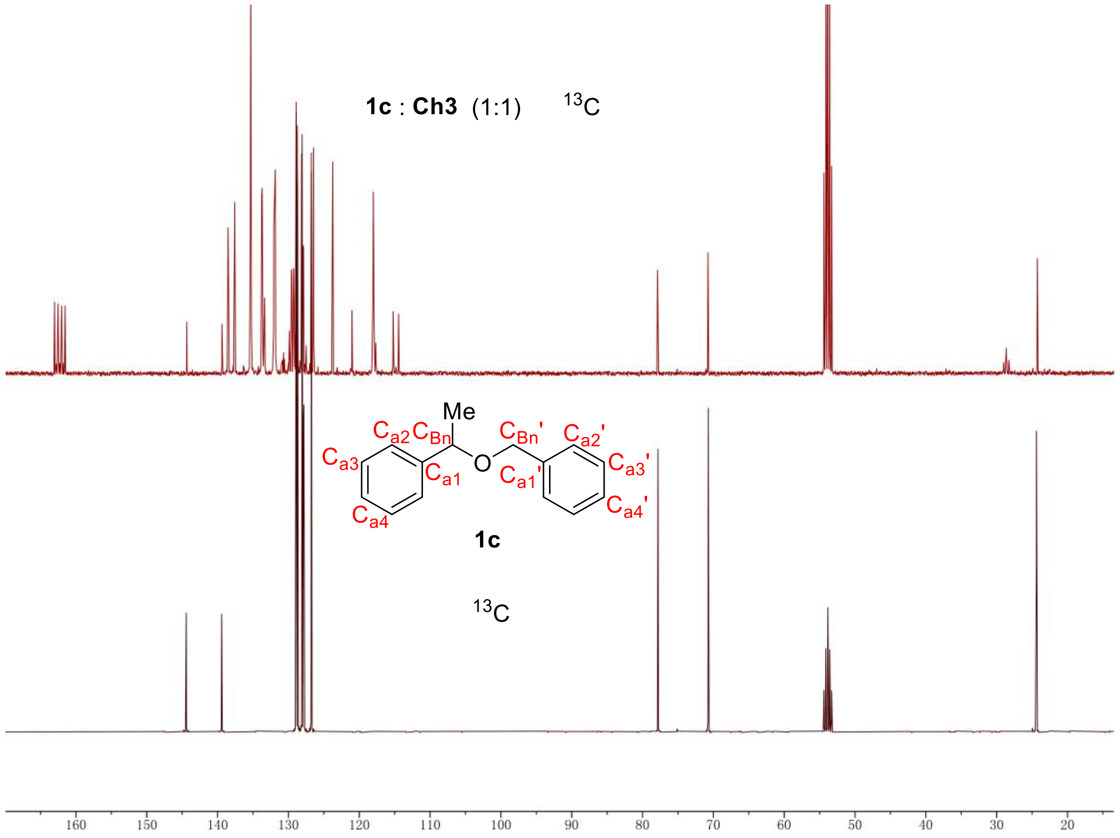

Supplementary Fig. 10 The interaction between Ch3 and 1c (^{77}Se NMR in CD_2Cl_2, 76 MHz, 298K). The ^{13}C NMR spectrum of **1c** upon addition of **Ch3**.

Supplementary Table 12. The Δδ ^{13}C NMR spectrum of 1c upon addition of Ch3.

	**C_{a1}** (ppm)	**C_{a1}'** (ppm)	**C_{a2-4}, C_{a2-4}'** (ppm)	**C_{Bn}** (ppm)	**C_{Bn}'** (ppm)
Only **1c**	144.42	139.42	128.89-126.75	77.81	70.72
**1c : Ch3** (1:1)	144.34	139.38	128.91-126.78	77.89	70.76
△δ	- 0.08	- 0.04	0.02-0.08	0.08	0.04

(6) Tracing the benchmark reaction process using ^{31}P NMR for **Ch3**

To a reaction mixture of catalyst **Ch3** (10 mol %) in a 10 mL-Schlenk tube was added CD_2Cl_2 (1.0 mL) under argon atmosphere. Then **1a** (0.2 mmol) and **2a** (3.0 equiv) was added to the above reaction mixture. The reaction mixture was stirred at room temperature. The reaction process was traced by ^{31}P NMR when the reaction was run for 12 h. NMR experiments reveal that only one ^{31}P signal (δ 30.21 ppm) assigned to catalyst **Ch3** was observed.

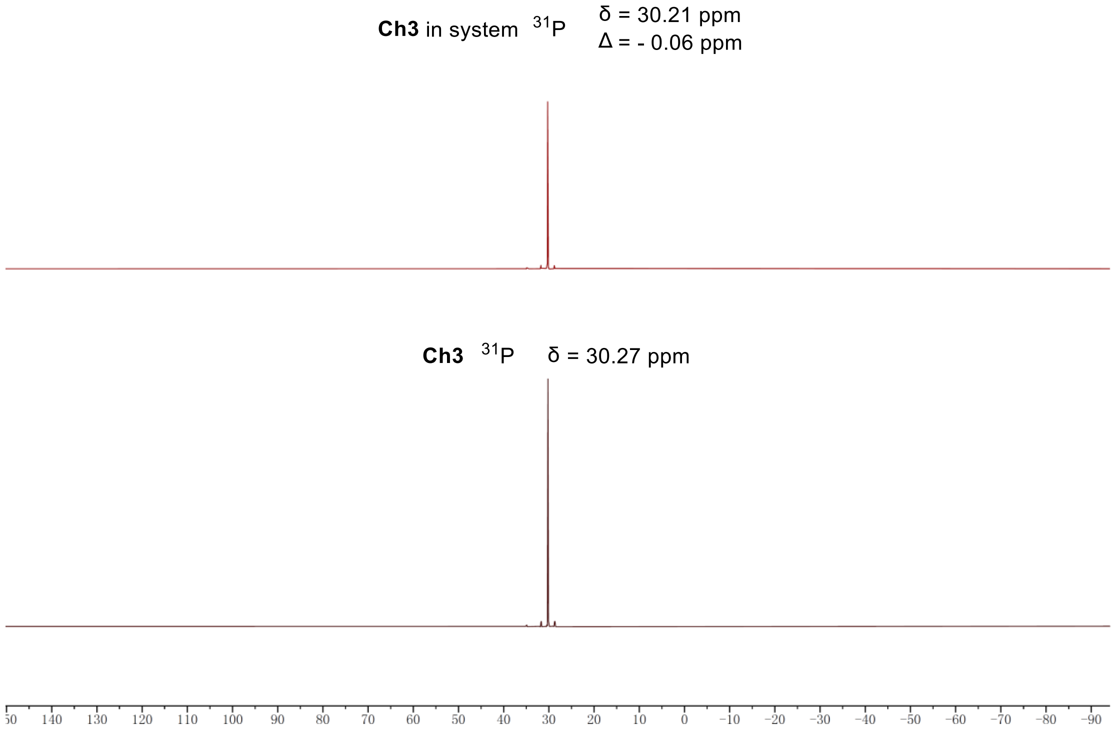

Supplementary Fig. 11 Tracing the reaction process using ^{31}P NMR for Ch3 (CD_2Cl_2, 162 MHz, 298K). The ^{31}P NMR spectrum of **Ch3** in reaction system at room temperature.

(7) The NMR studies of **Ch3** and **1x**

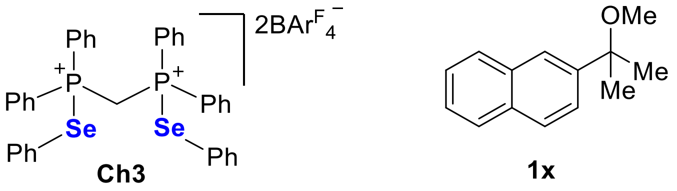

a) The ^{1}H NMR studies of **Ch3** and **1x**: To a mixture of catalyst **Ch3** (0.05 mmol) and **1x** (0.05 mmol) in an NMR tube was added CD_2Cl_2 (0.5 mL) and then analysis of the reaction mixture by ^{1}H NMR experiments.

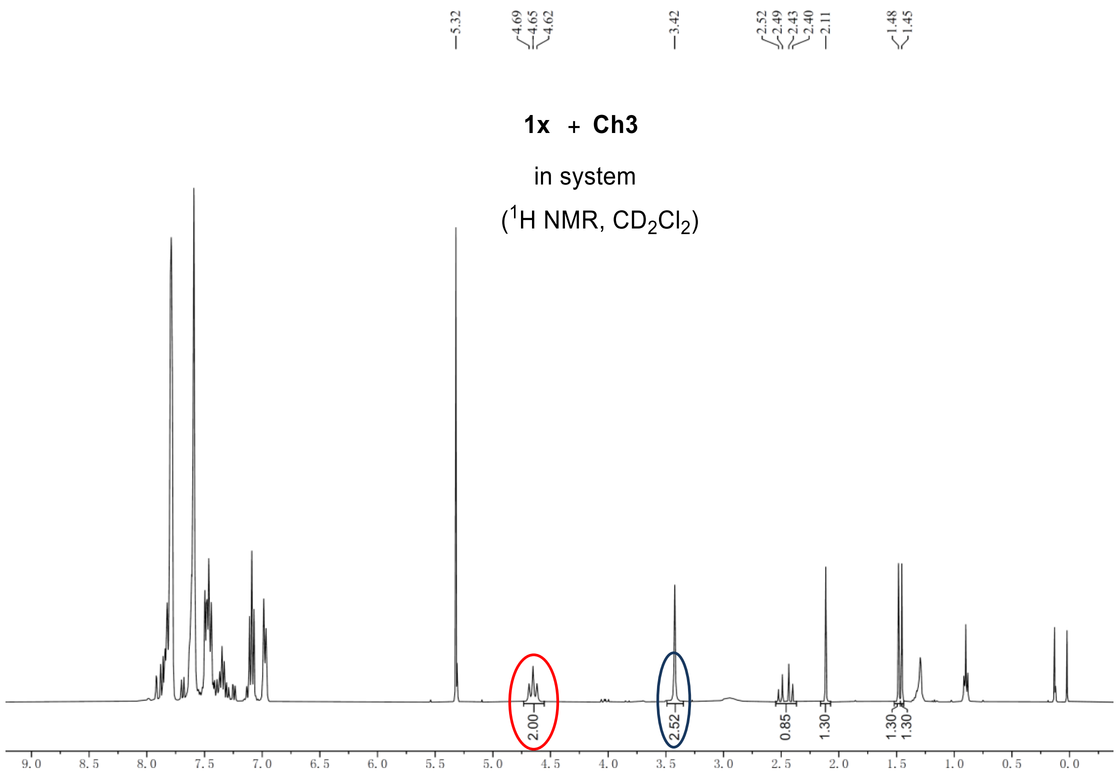

Supplementary Fig. 12 Tracing the reaction process using ^{1}H NMR for 1x (CD_2Cl_2, 400 MHz, 298K). The ^{1}H NMR spectrum of the reaction system at room temperature.

b) Tracing the reaction process using ^{31}P NMR: To a reaction mixture of catalyst **Ch3** (10 mol %) and **1x** (0.2 mmol) in a 10 mL-Schlenk tube was added CD_2Cl_2 (1.0 mL) under argon atmosphere. The above reaction mixture was stirred at room temperature. The reaction process was traced by ^{31}P NMR when the reaction was run for 2 h (**25**, 73% isolated yield). Only one ^{31}P signal (δ 30.22 ppm) assigned to catalyst **Ch3** was observed.

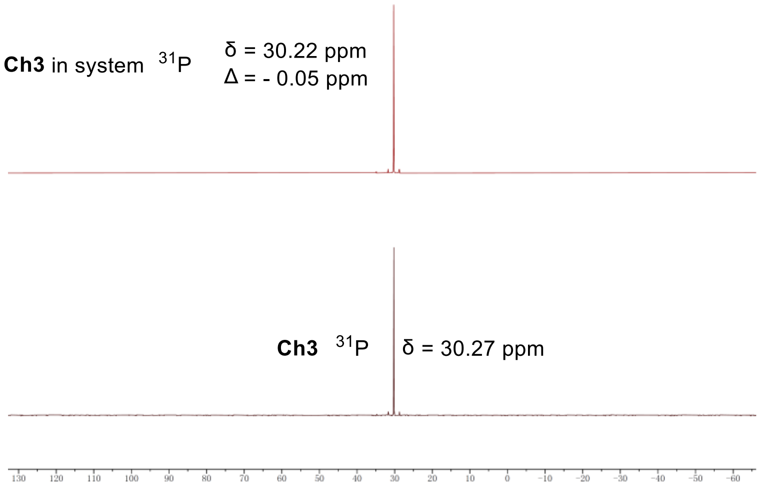

Supplementary Fig. 13 Tracing the reaction process using ^{31}P NMR for Ch3 (CD_2Cl_2, 162 MHz, 298K). The ^{31}P NMR spectrum of **Ch3** in reaction system at room temperature.

(8) Tracing the benchmark reaction process using ^{31}P NMR for **Ch14**

To a reaction mixture of catalyst **Ch14** (10 mol %) in a 10 mL-Schlenk tube was added CD_2Cl_2 (1.0 mL) under argon atmosphere. Then **1a** (0.2 mmol) and **2a** (3.0 equiv) was added to the above reaction mixture. The reaction mixture was stirred at room temperature. The reaction process was traced by ^{31}P NMR when the reaction was run for 12 h. NMR experiments reveal that only one ^{31}P signal (δ 31.11 ppm) assigned to catalyst **Ch14** was observed.

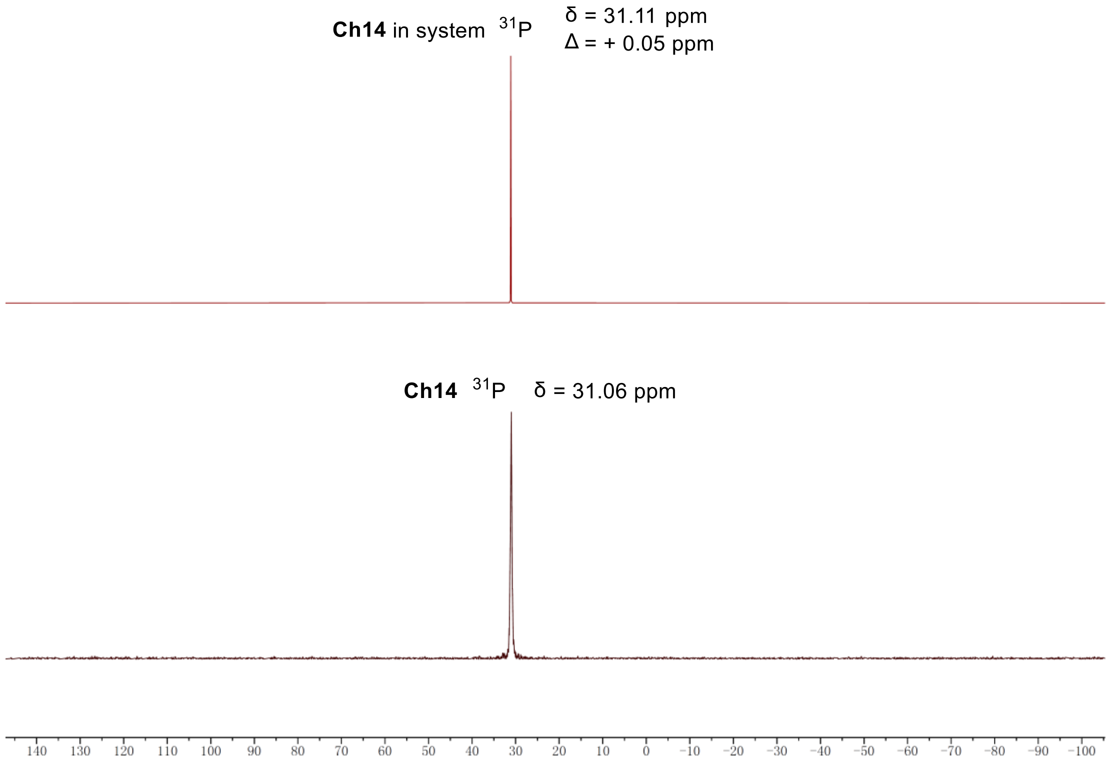

Supplementary Fig. 14 Tracing the reaction process using ^{31}P NMR for Ch14 (CD_2Cl_2, 162 MHz, 298K). The ^{31}P NMR spectrum of **Ch14** in reaction system at room temperature.

7. DFT Calculations

Computational details

The binding modes between **Ch3** and ether **e1** were further assessed via DFT calculations. The possible complexes between **Ch3** and ether **e1** (i.e., **COM1**, **COM3**) were constructed, and the geometries of **Ch3**, **e1** and the complexes were optimized at the M06-2X/6-311g(d,p) level of theory corrected with the Grimme's dispersion (D3) using the Gaussian 09 program,[8-10] and vibrational frequency calculations were also performed at this level. The integral equation formalism model (IEFPCM) in which dichloroethane was chosen as the solvent to simulate solvation effect.[11] The relative Gibbs free energy (i.e., ΔG) for the complex is calculated as the Gibbs free energy difference between the Gibbs free energy of the complex and the sum of Gibbs free energies of its components, while the interaction energy (i.e., ΔE) is calculated as the energy difference between the total energy of the complex and the sum of energies of its components. The molecular electrostatic potential (MEP) was used to analyze the molecular electronic property,[12-13] which can be helpful for understanding the role of noncovalent contacts in studied systems. To understand the interaction types in the complexes, the non-covalent interaction (NCI) analysis was also employed,[14-15] and the NCI plots were visualized using the Multiwfn software and Visual Molecular Dynamics (VMD) program.[16-17]

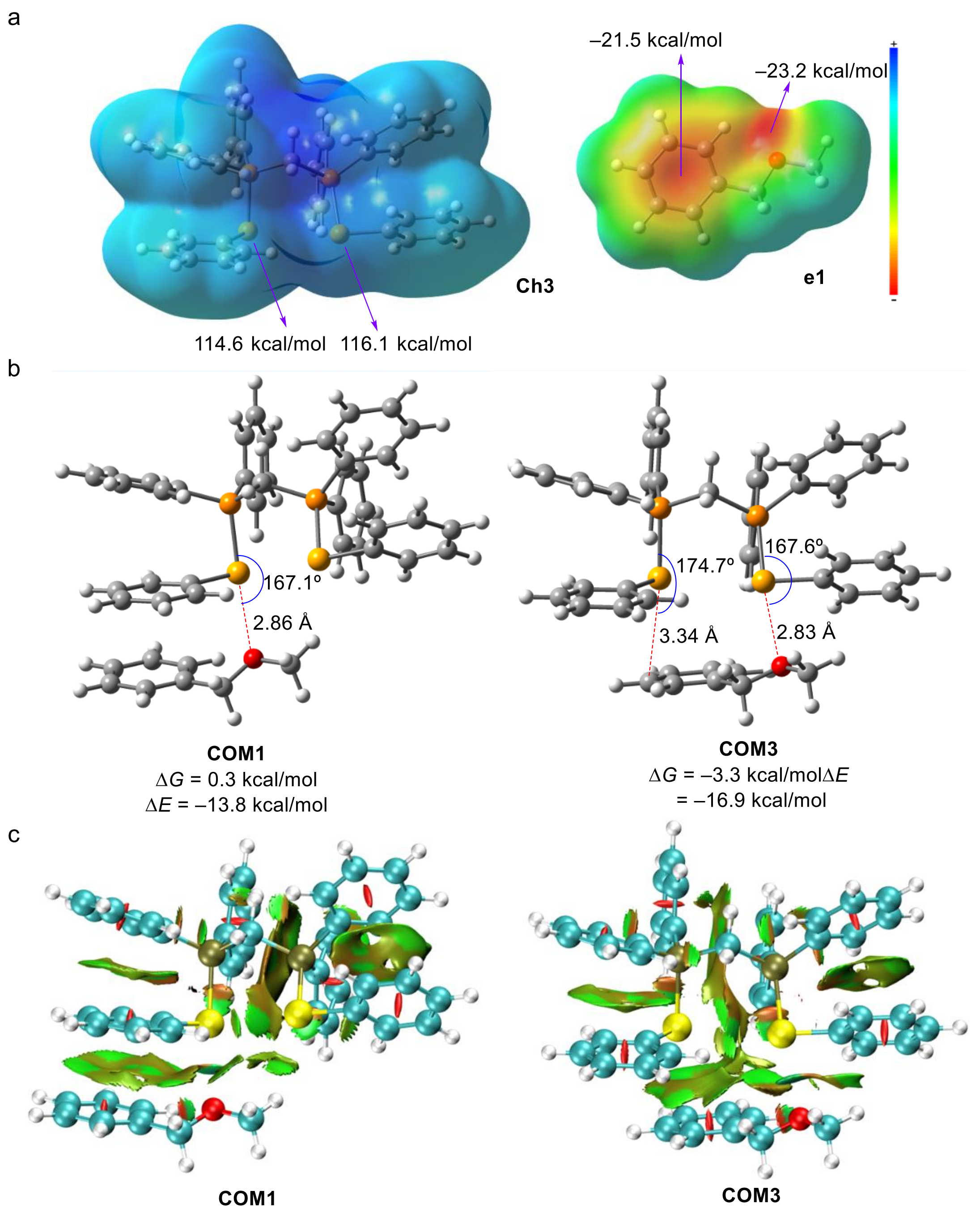

Supplementary Fig. 15 DFT calculations. a Electrostatic potential map. **b** Optimized complexes. **c** NCI analysis of complexes.

8. X-Ray Crystallographic Data

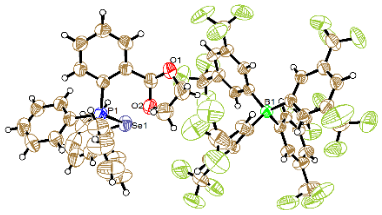

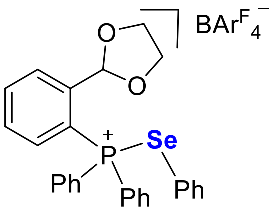

Ch8

Supplementary Fig. 16 X-ray crystallographic structure of Ch8; ellipsoid contour at the 50% probability level (CCDC 2203201).

Supplementary Table 13. Crystal data and structure refinement for Ch8.

Empirical formula	$C_{59}H_{36}BF_{24}O_2PSe$
Formula weight	1353.62
Temperature/K	173.0
Crystal system	monoclinic
Space group	C2/c
a/Å	18.4810(9)
b/Å	18.0085(9)
c/Å	36.1706(17)
α/°	90
β/°	98.758(2)
γ/°	90
Volume/Å^3	11897.8(10)
Z	8
ρ_{calc}g/cm^3	1.511
μ/mm^{-1}	2.172
F(000)	5408.0
Crystal size/mm^3	0.8 × 0.62 × 0.13
Radiation	CuKα (λ = 1.54184)
2Θ range for data collection/°	4.944 to 133.408
Index ranges	$-21 \leq h \leq 21$, $-21 \leq k \leq 20$, $-41 \leq l \leq 43$
Reflections collected	41756
Independent reflections	10379 [R_{int} = 0.0406, R_{sigma} = 0.0335]
Data/restraints/parameters	10379/141/874
Goodness-of-fit on F^2	1.066
Final R indexes [I>=2σ (I)]	R_1 = 0.0715, wR_2 = 0.2143
Final R indexes [all data]	R_1 = 0.0774, wR_2 = 0.2212
Largest diff. peak/hole / e Å^{-3}	1.56/-0.75

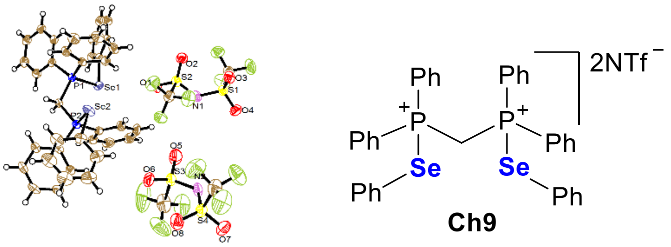

Supplementary Fig. 17 X-ray crystallographic structure of Ch9; ellipsoid contour at the 50% probability level (CCDC 2203203).

Supplementary Table 14. Crystal data and structure refinement for Ch9.

Empirical formula	$C_{41}H_{32}F_{12}N_2O_8P_2S_4Se_2$
Formula weight	1256.831
Temperature/K	173.00(10)
Crystal system	monoclinic
Space group	$P2_1/n$
a/Å	18.6106(3)
b/Å	12.52909(17)
c/Å	21.3369(3)
α/°	90
β/°	97.5901(12)
γ/°	90
Volume/Å^3	4931.62(12)
Z	4
ρ_{calc}g/cm^3	1.693
μ/mm^{-1}	4.956
F(000)	2514.2
Crystal size/mm^3	0.02 × 0.01 × 0.01
Radiation	Cu Kα (λ = 1.54184)
2Θ range for data collection/°	5.92 to 134.14
Index ranges	$-23 \le h \le 23$, $-15 \le k \le 13$, $-26 \le l \le 26$
Reflections collected	33356
Independent reflections	8702 [R_{int} = 0.0527, R_{sigma} = 0.0519]
Data/restraints/parameters	8702/36/640
Goodness-of-fit on F^2	1.025
Final R indexes [I>=2σ (I)]	R_1 = 0.0389, wR_2 = 0.0980
Final R indexes [all data]	R_1 = 0.0479, wR_2 = 0.1041
Largest diff. peak/hole / e Å^{-3}	0.98/-0.52

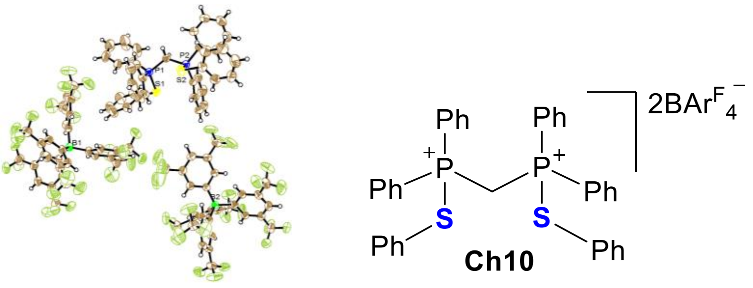

Supplementary Fig. 18 X-ray crystallographic structure of Ch10; ellipsoid contour at the 50% probability level (CCDC 2203204).

Supplementary Table 15. Crystal data and structure refinement for Ch10.

Empirical formula	$C_{101}H_{56}B_2F_{48}P_2S_2$
Formula weight	2329.13
Temperature/K	173.0
Crystal system	orthorhombic
Space group	Pbcn
a/Å	19.4058(7)
b/Å	25.7758(10)
c/Å	23.7889(9)
α/°	90
β/°	90
γ/°	90
Volume/Å^3	11899.2(8)
Z	4
ρ_{calc}g/cm^3	1.300
μ/mm^{-1}	1.708
F(000)	4664.0
Crystal size/mm^3	0.07 × 0.06 × 0.03
Radiation	CuKα (λ = 1.54184)
2Θ range for data collection/°	9.114 to 133.494
Index ranges	$-18 \leq h \leq 23$, $-30 \leq k \leq 28$, $-28 \leq l \leq 27$
Reflections collected	68318
Independent reflections	10514 [R_{int} = 0.0482, R_{sigma} = 0.0299]
Data/restraints/parameters	10514/630/835
Goodness-of-fit on F^2	1.068
Final R indexes [I>=2σ (I)]	R_1 = 0.0645, wR_2 = 0.1914
Final R indexes [all data]	R_1 = 0.0765, wR_2 = 0.2043
Largest diff. peak/hole / e Å^{-3}	0.85/-0.49

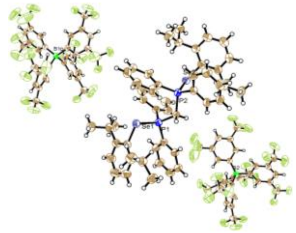

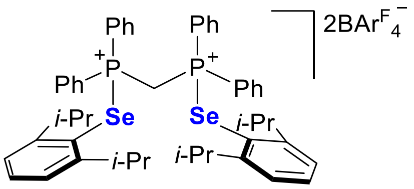

Ch11

Supplementary Fig. 19 X-ray crystallographic structure of Ch11; ellipsoid contour at the 50% probability level (CCDC 2203205).

Supplementary Table 16. Crystal data and structure refinement for Ch11.

Empirical formula	$C_{113}H_{80}B_2F_{48}P_2Se_2$
Formula weight	2591.25
Temperature/K	173.0
Crystal system	orthorhombic
Space group	Pbcn
a/Å	18.919(3)
b/Å	27.932(5)
c/Å	23.078(4)
α/°	90
β/°	90
γ/°	90
Volume/Å^3	12195(4)
Z	4
ρ_{calc}g/cm^3	1.411
μ/mm^{-1}	2.065
F(000)	5192.0
Crystal size/mm^3	0.16 × 0.14 × 0.12
Radiation	CuKα (λ = 1.54178)
2Θ range for data collection/°	6.328 to 133.104
Index ranges	$-22 \le h \le 22$, $-32 \le k \le 33$, $-27 \le l \le 25$
Reflections collected	93570
Independent reflections	10766 [R_{int} = 0.0701, R_{sigma} = 0.0351]
Data/restraints/parameters	10766/55/757
Goodness-of-fit on F^2	1.094
Final R indexes [I>=2σ (I)]	R_1 = 0.0526, wR_2 = 0.1463
Final R indexes [all data]	R_1 = 0.0651, wR_2 = 0.1564
Largest diff. peak/hole / e Å^{-3}	1.05/-0.69

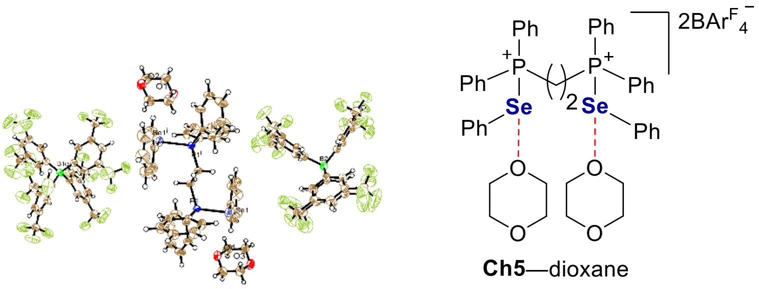

Ch5—dioxane

Supplementary Fig. 20 X-ray crystallographic structure of Ch5-dioxane; ellipsoid contour at the 50% probability level (CCDC 2203200).

Supplementary Table 17. Crystal data and structure refinement for Ch5-dioxane.

Empirical formula	$C_{110}H_{74}B_2F_{48}O_4P_2Se_2$
Formula weight	2613.17
Temperature/K	173(1)
Crystal system	triclinic
Space group	P-1
a/Å	12.6954(3)
b/Å	14.8783(4)
c/Å	16.9430(4)
α/°	71.430(2)
β/°	89.7350(19)
γ/°	66.845(2)
Volume/Å^3	2762.46(12)
Z	1
ρ_{calc}g/cm^3	1.571
μ/mm^{-1}	2.313
F(000)	1306.0
Crystal size/mm^3	0.06 × 0.06 × 0.05
Radiation	CuKα (λ = 1.54184)
2Θ range for data collection/°	6.882 to 134.15
Index ranges	-15 ≤ h ≤ 15, -13 ≤ k ≤ 17, -20 ≤ l ≤ 20
Reflections collected	28612
Independent reflections	9715 [R_{int} = 0.0333, R_{sigma} = 0.0324]
Data/restraints/parameters	9715/171/784
Goodness-of-fit on F^2	1.054
Final R indexes [I>=2σ (I)]	R_1 = 0.0612, wR_2 = 0.1656
Final R indexes [all data]	R_1 = 0.0656, wR_2 = 0.1690
Largest diff. peak/hole / e Å^{-3}	1.23/-1.14

9. Copies of NMR Spectra

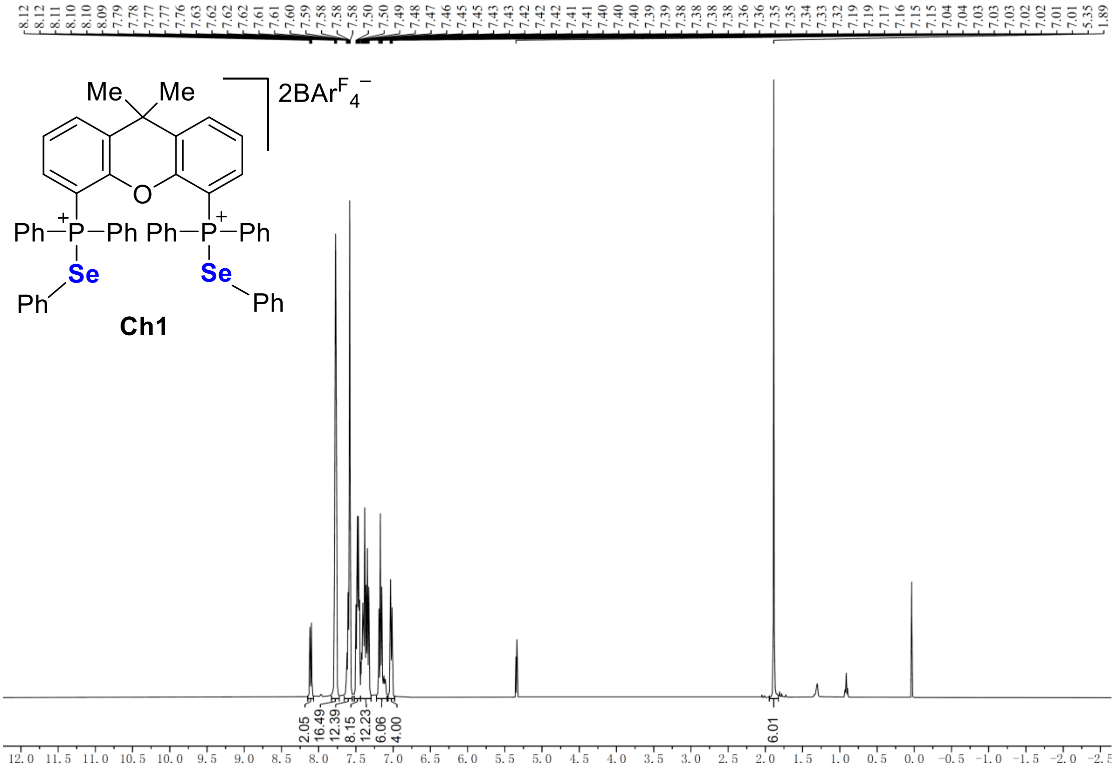

Supplementary Fig. 21 ^{1}H NMR spectrum of compound Ch1 (CD_2Cl_2, 400 MHz, 298K)

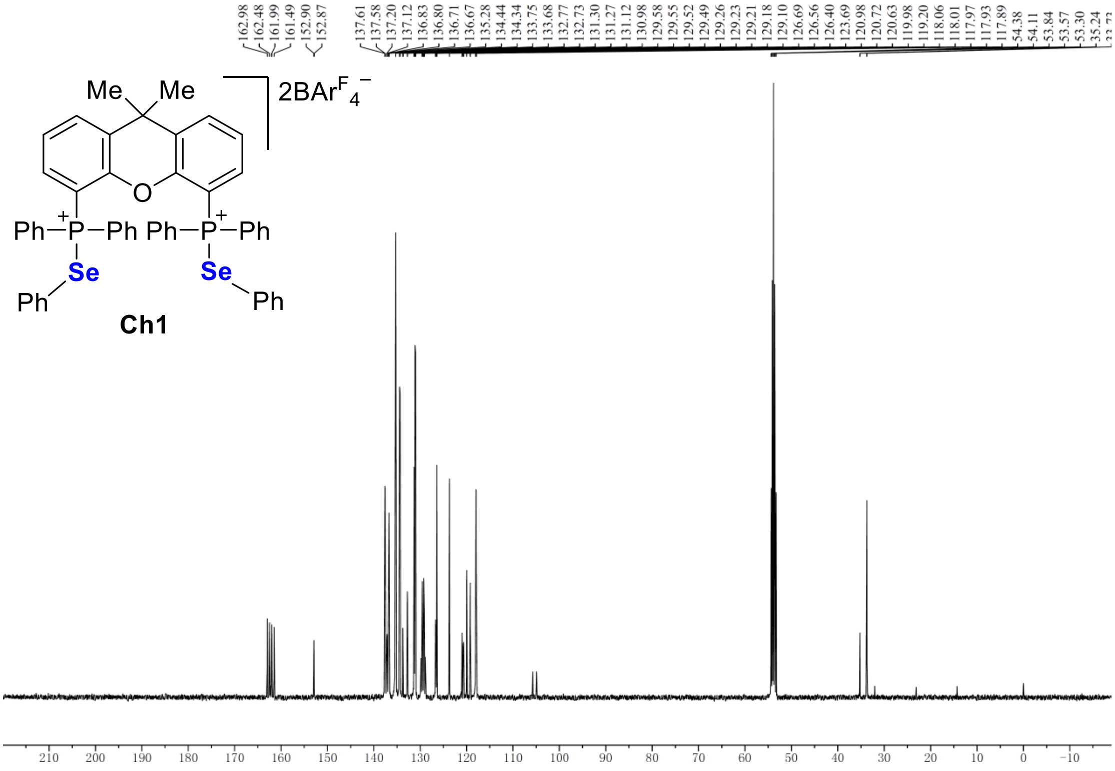

Supplementary Fig. 22 ^{13}C NMR spectrum of compound Ch1 (CD_2Cl_2, 100 MHz, 298K)

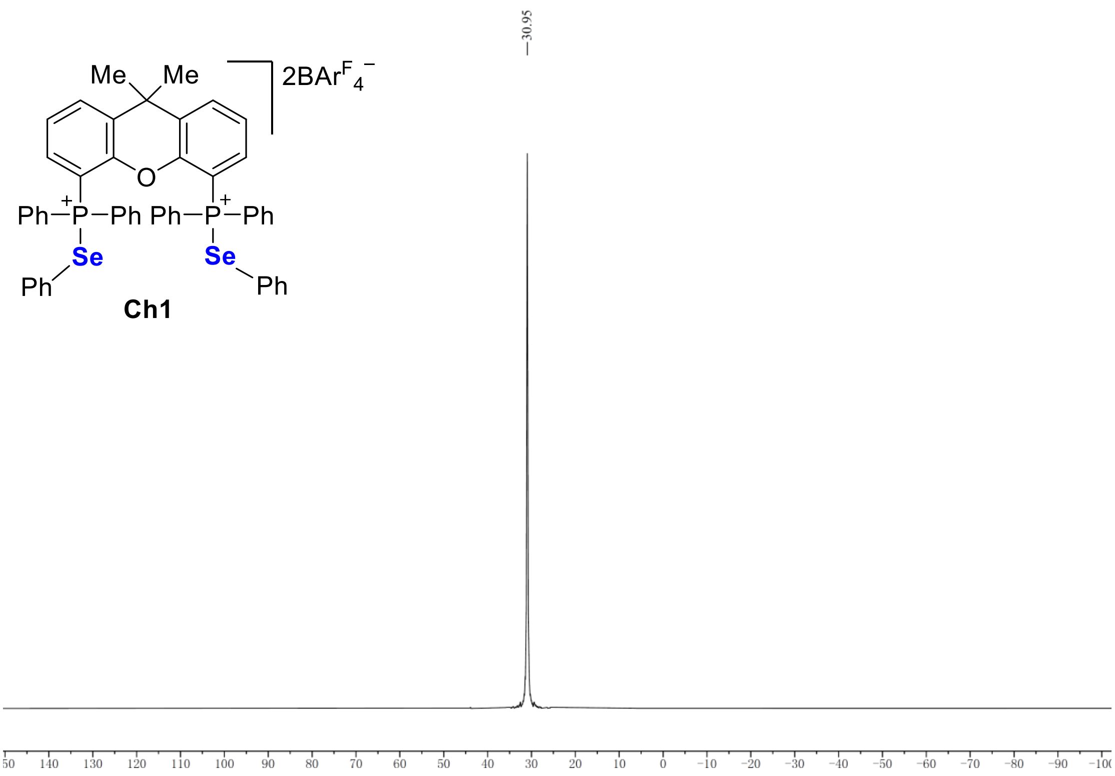

Supplementary Fig. 23 ^{31}P NMR spectrum of compound Ch1 (CD_2Cl_2, 162 MHz, 298K)

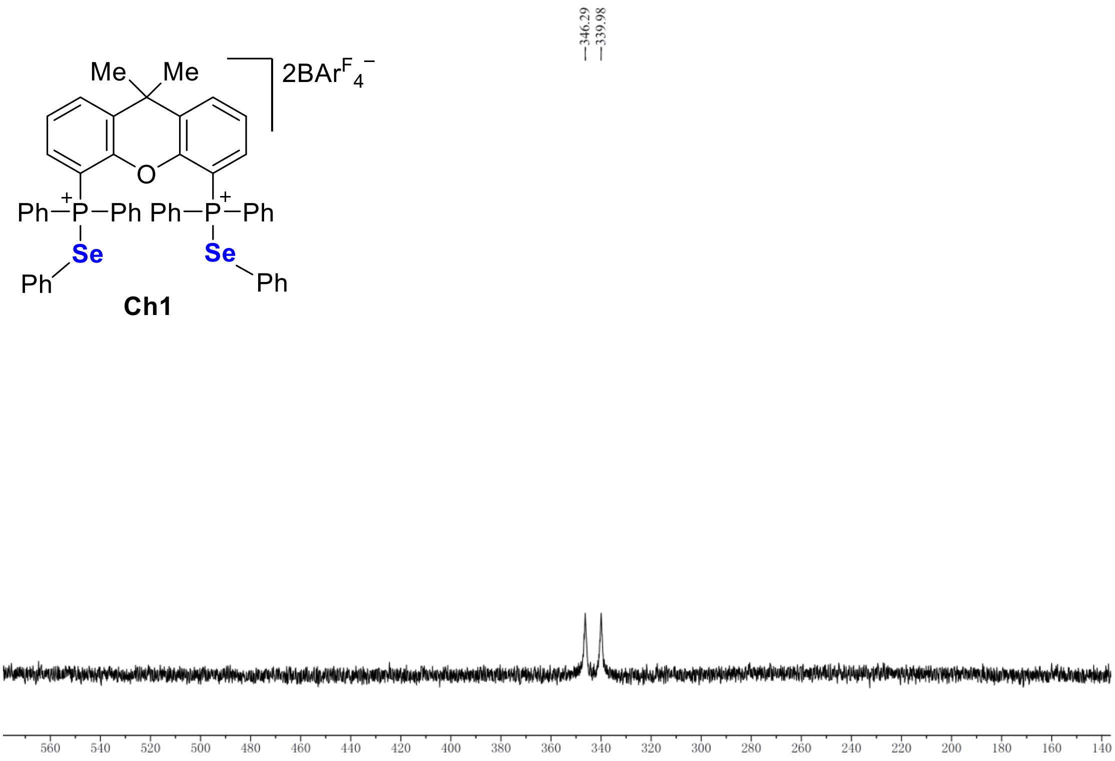

Supplementary Fig. 24 ^{77}Se NMR spectrum of compound Ch1 (CD_2Cl_2, 76 MHz, 298K)

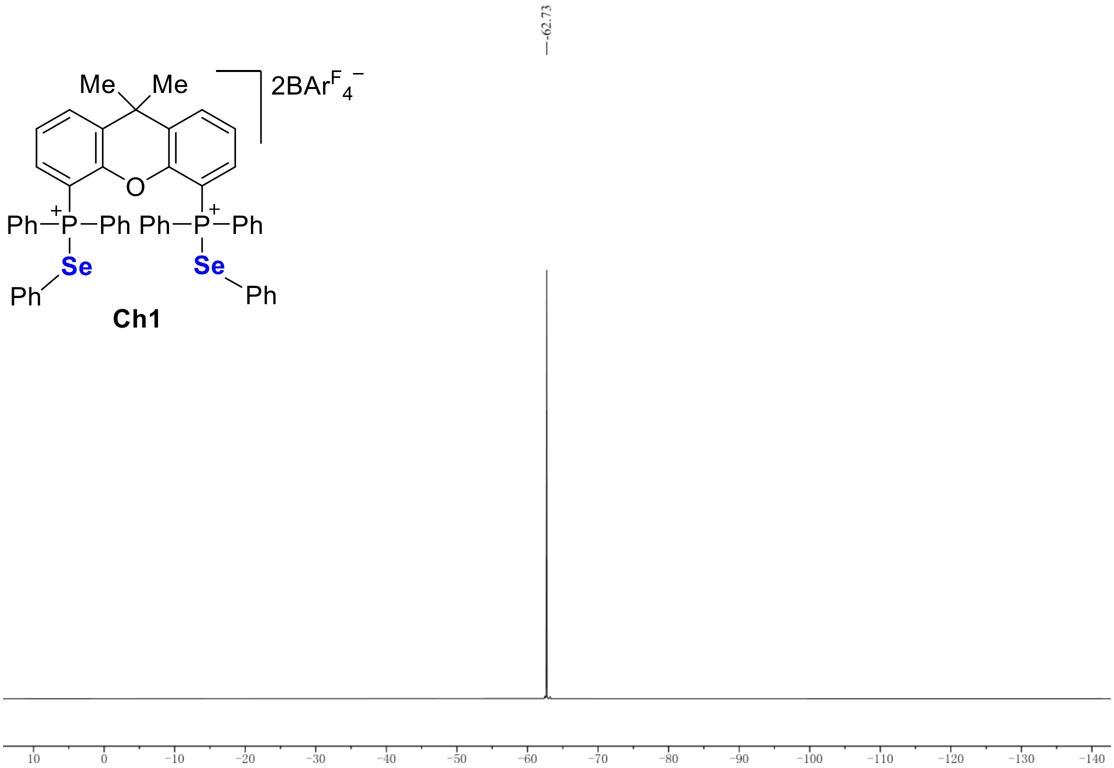

Supplementary Fig. 25 ^{19}F NMR spectrum of compound Ch1 (CD_2Cl_2, 376 MHz, 298K)

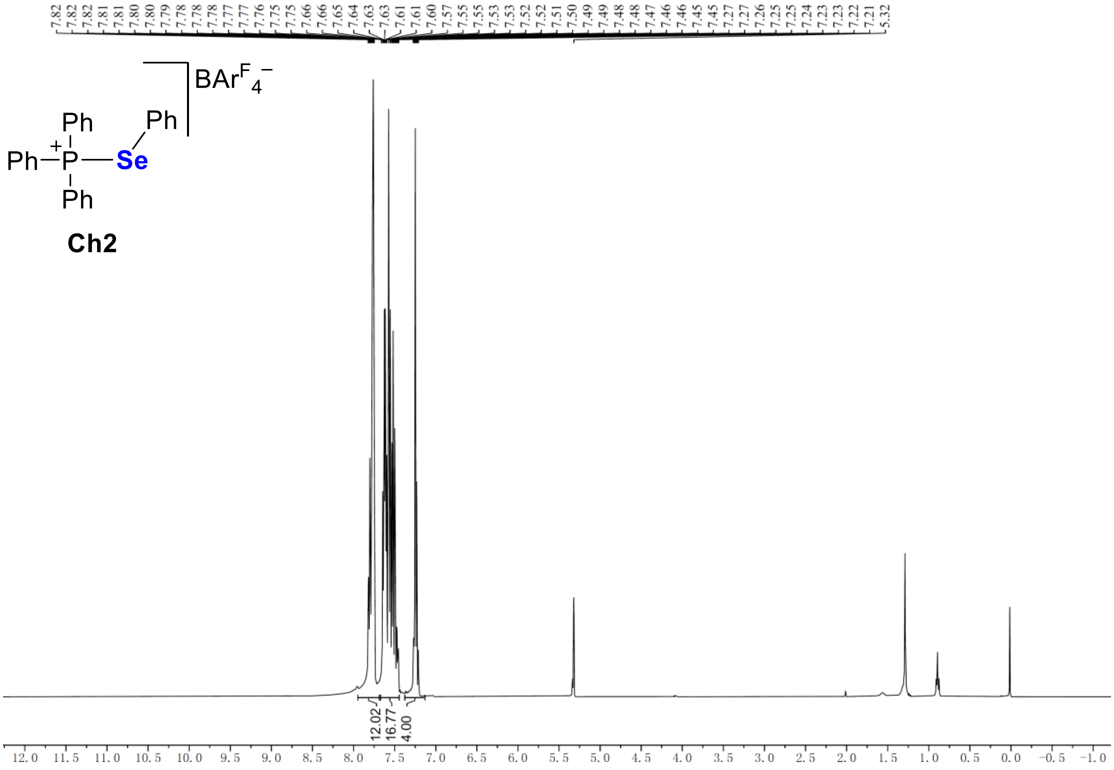

Supplementary Fig. 26 ^{1}H NMR spectrum of compound Ch2 (CD_2Cl_2, 400 MHz, 298K)

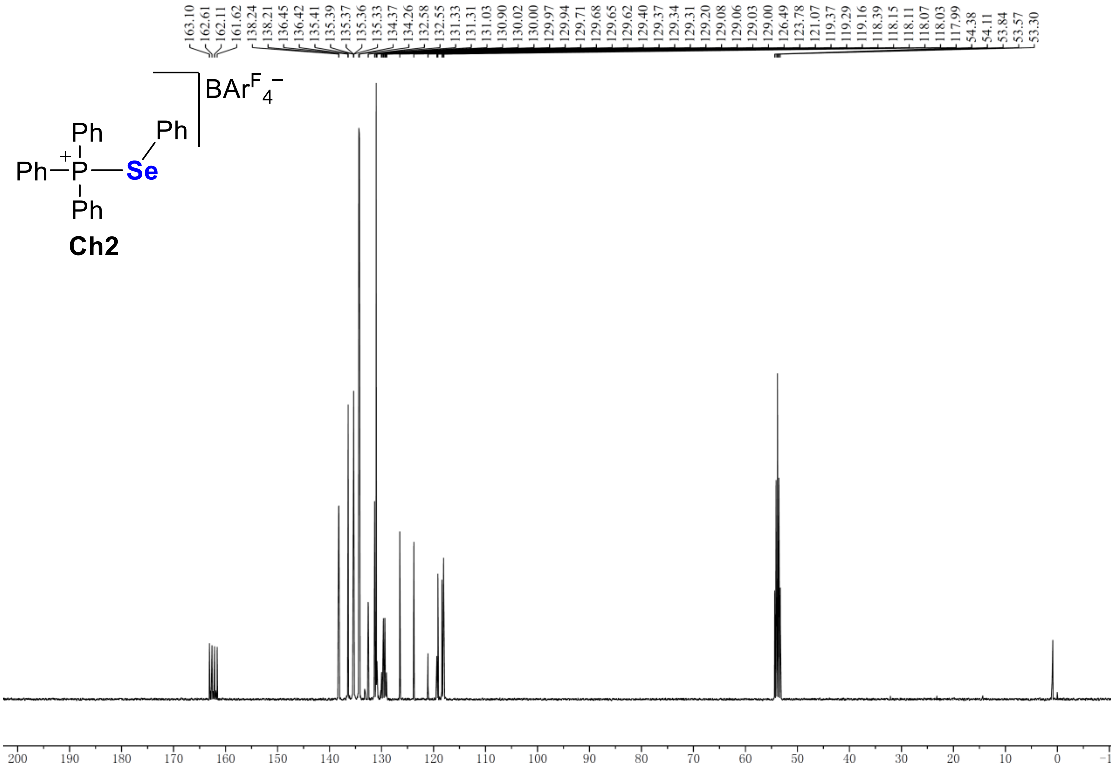

Supplementary Fig. 27 ^{13}**C NMR spectrum of compound Ch2 (CD$_2$Cl$_2$, 100 MHz, 298K)**

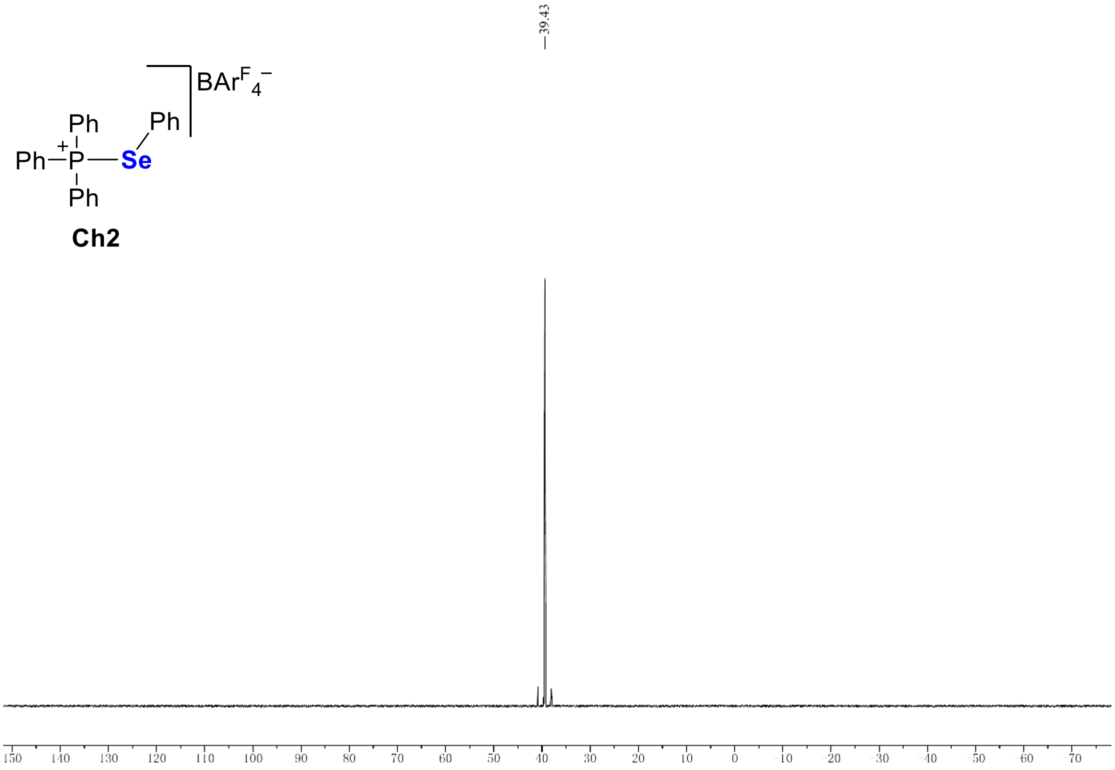

Supplementary Fig. 28 ^{31}**P NMR spectrum of compound Ch2 (CD$_2$Cl$_2$, 162 MHz, 298K)**

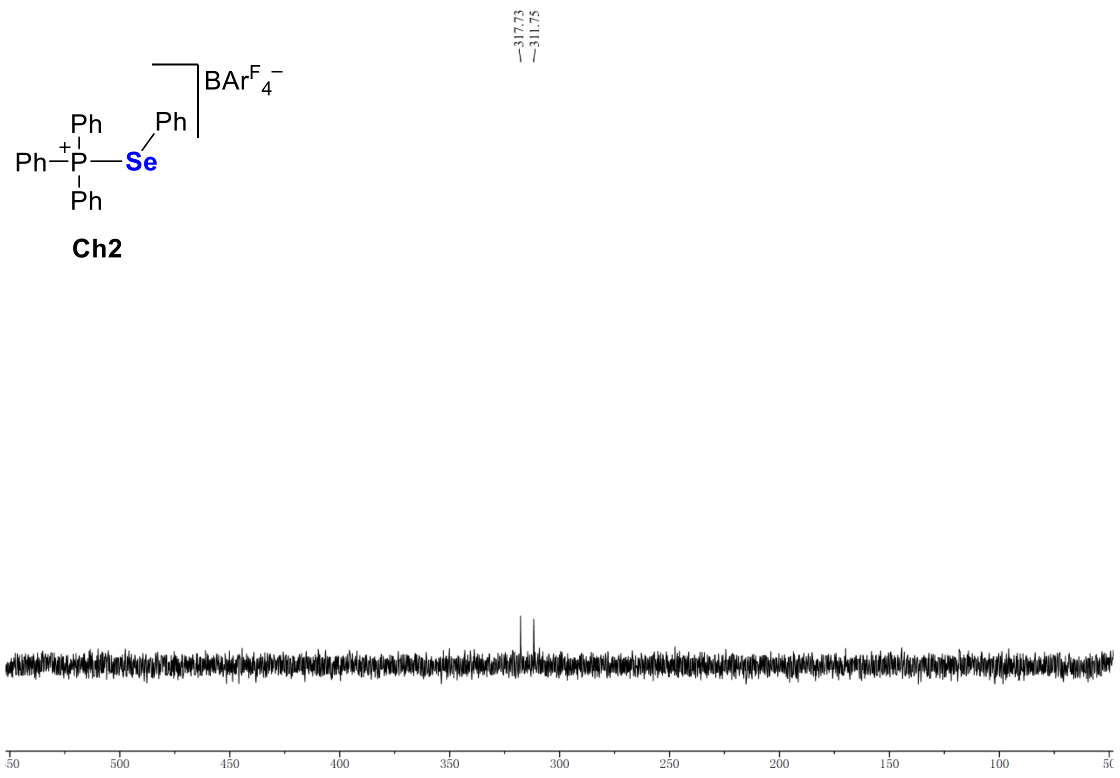

Supplementary Fig. 29 ^{77}**Se NMR spectrum of compound Ch2 (**CD_2Cl_2**, 76 MHz, 298K)**

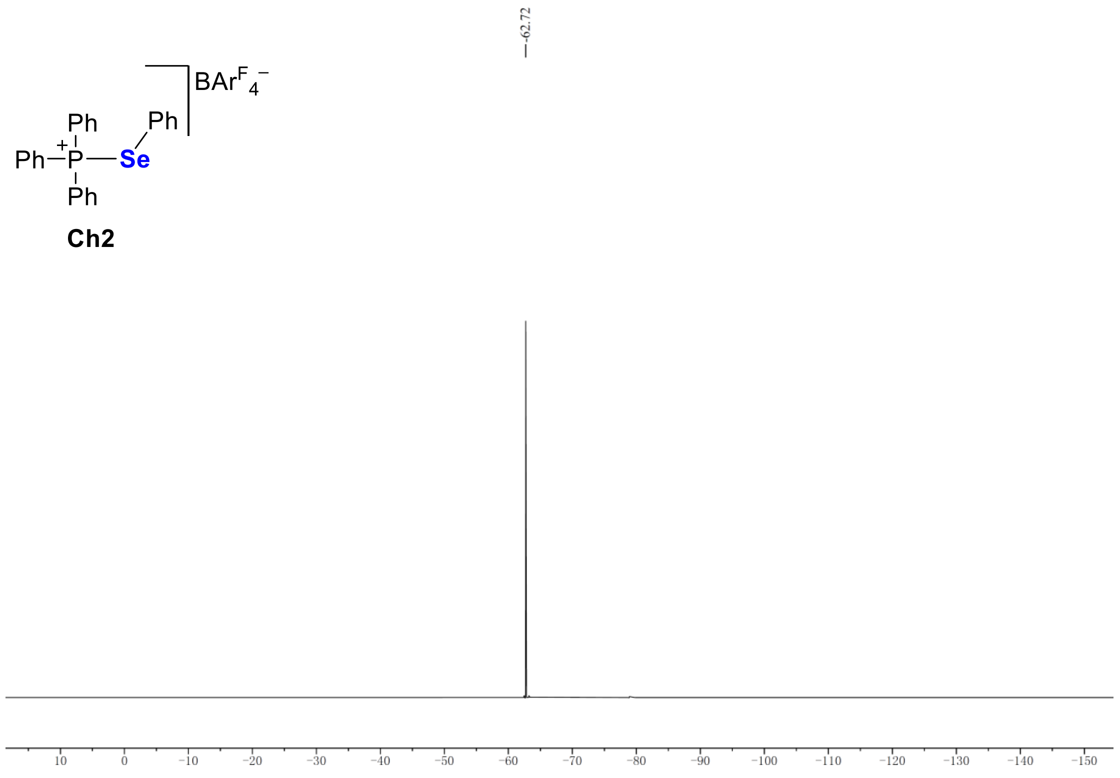

Supplementary Fig. 30 ^{19}**F NMR spectrum of compound Ch2 (**CD_2Cl_2**, 376 MHz, 298K)**

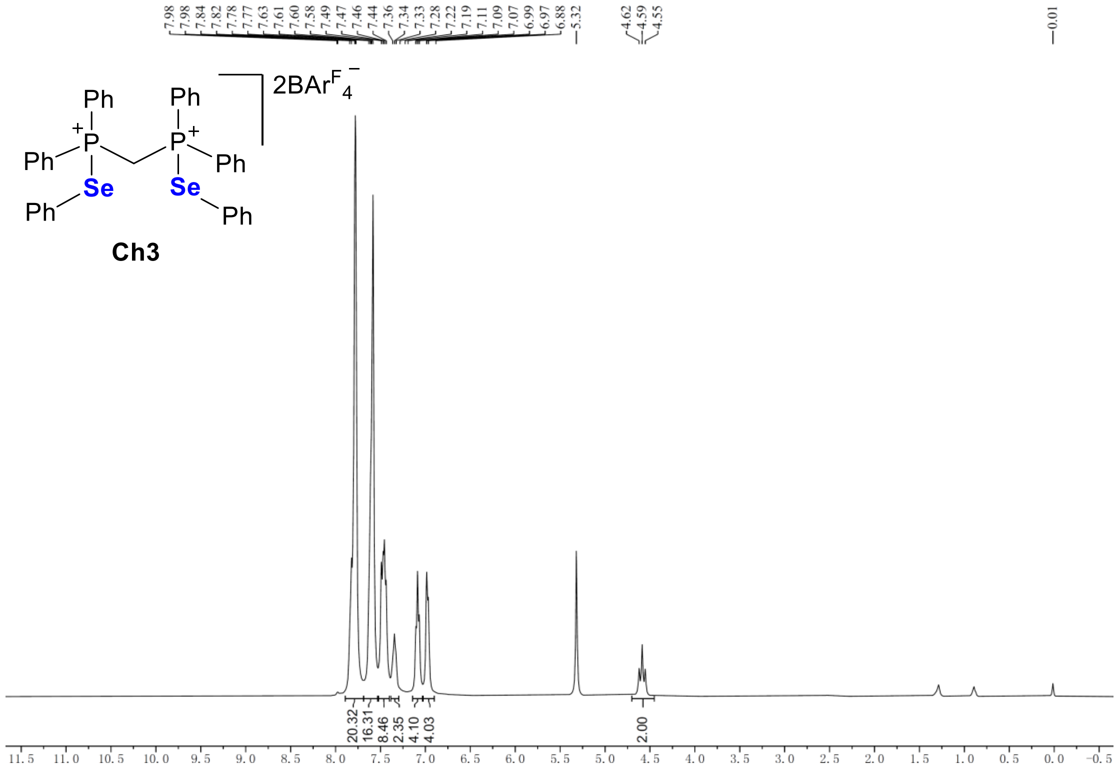

Supplementary Fig. 31 **^{1}H NMR spectrum of compound Ch3 (CD_2Cl_2, 400 MHz, 298K)**

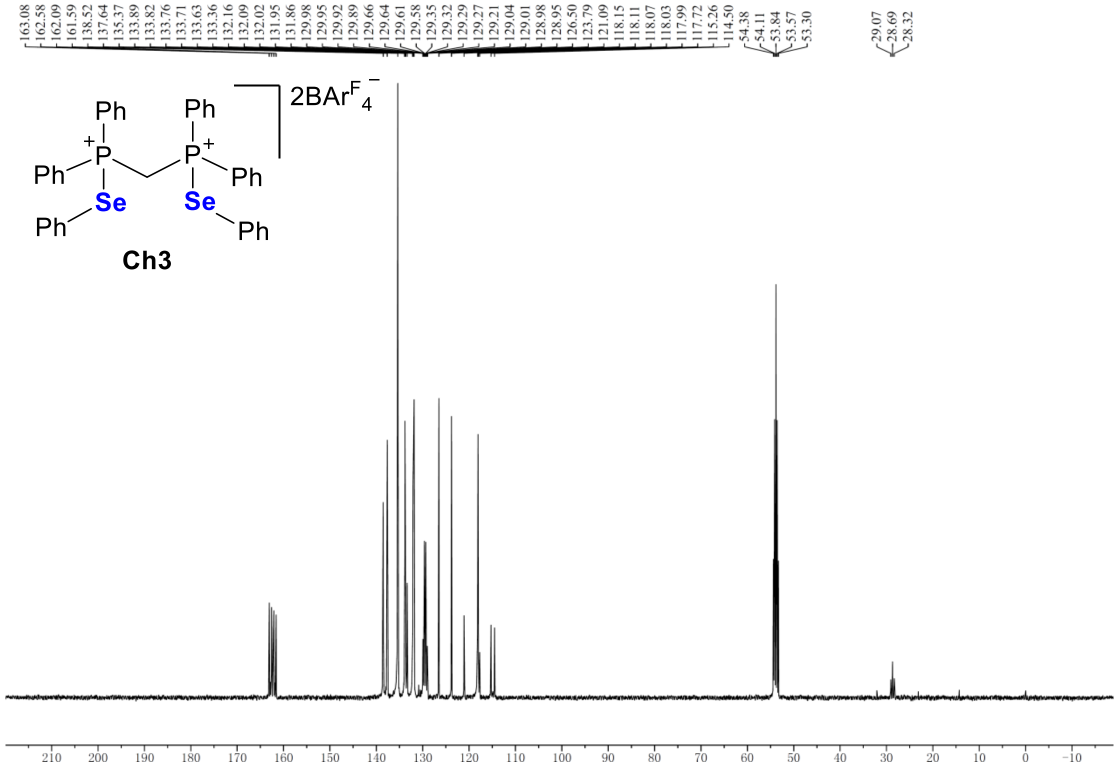

Supplementary Fig. 32 **^{13}C NMR spectrum of compound Ch3 (CD_2Cl_2, 100 MHz, 298K)**

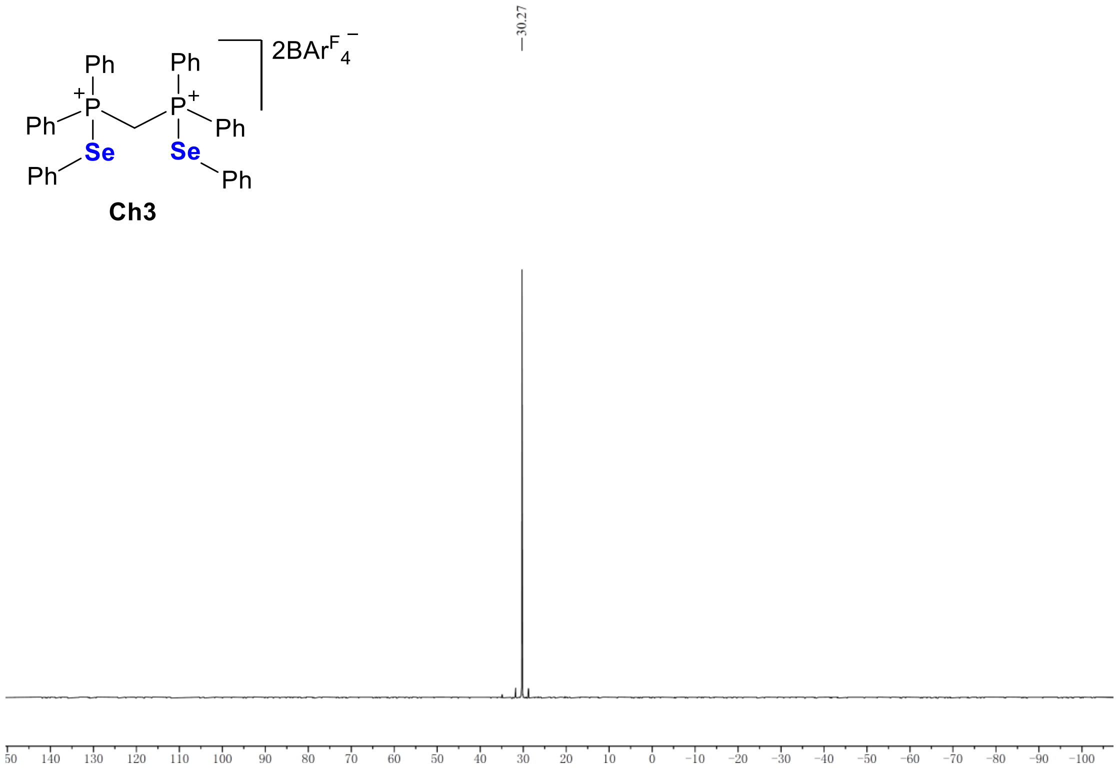

Supplementary Fig. 33 ^{31}**P NMR spectrum of compound Ch3 (CD_2Cl_2, 162 MHz, 298K)**

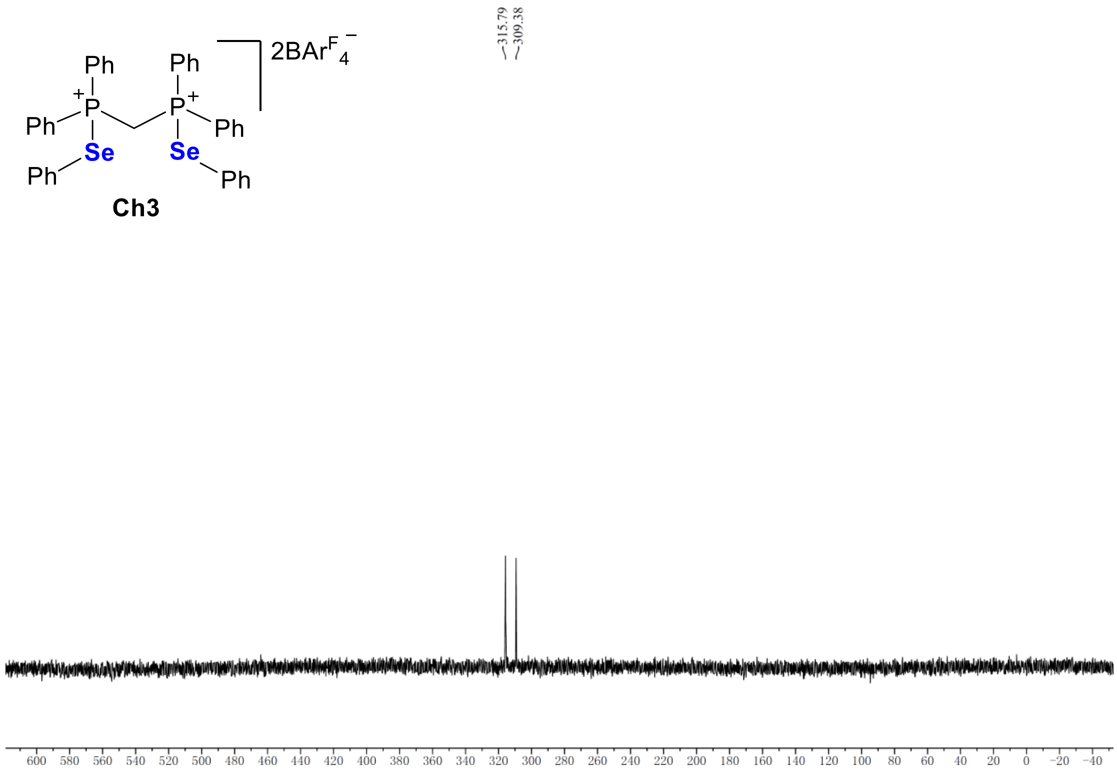

Supplementary Fig. 34 ^{77}**Se NMR spectrum of compound Ch3 (CD_2Cl_2, 76 MHz, 298K)**

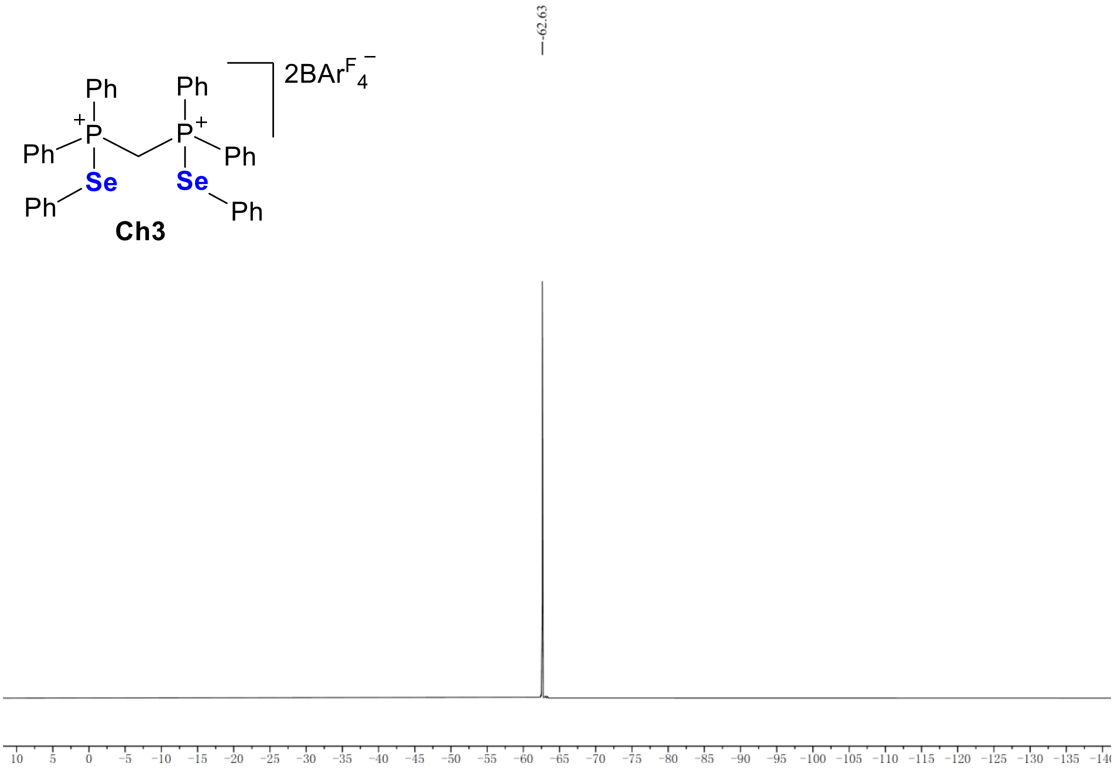

Supplementary Fig. 35 ^{19}F NMR spectrum of compound Ch3 (CD_2Cl_2, 376 MHz, 298K)

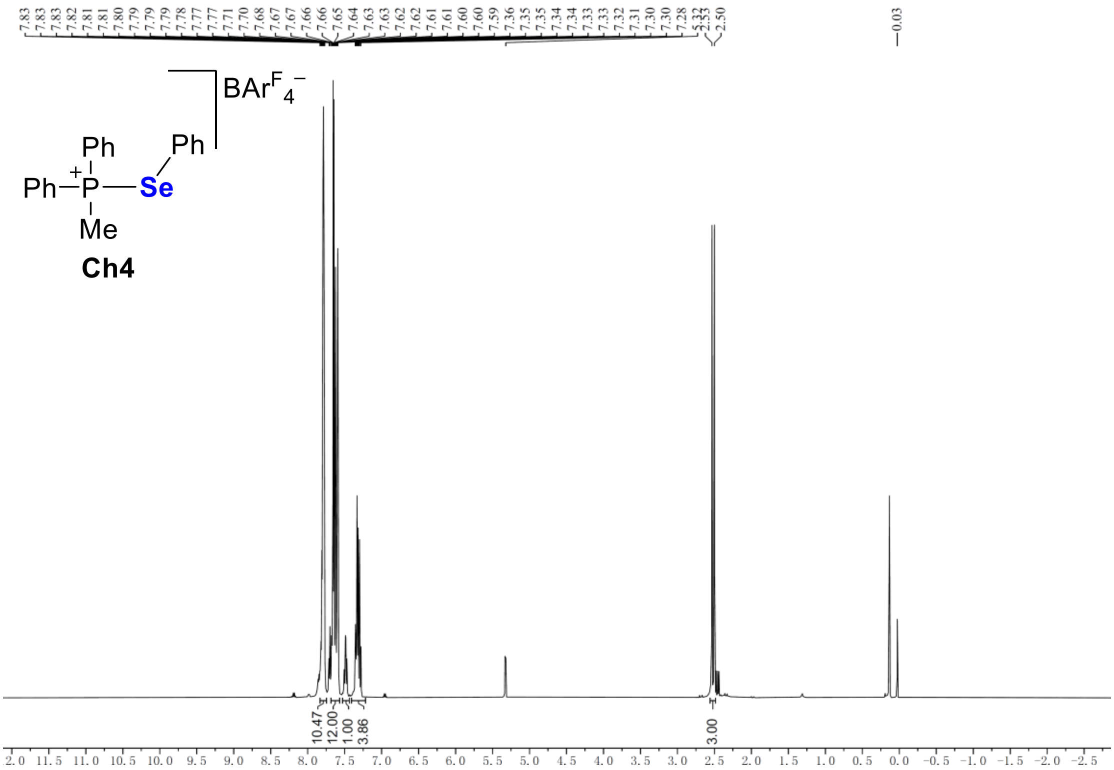

Supplementary Fig. 36 ^{1}H NMR spectrum of compound Ch4 (CD_2Cl_2, 400 MHz, 298K)

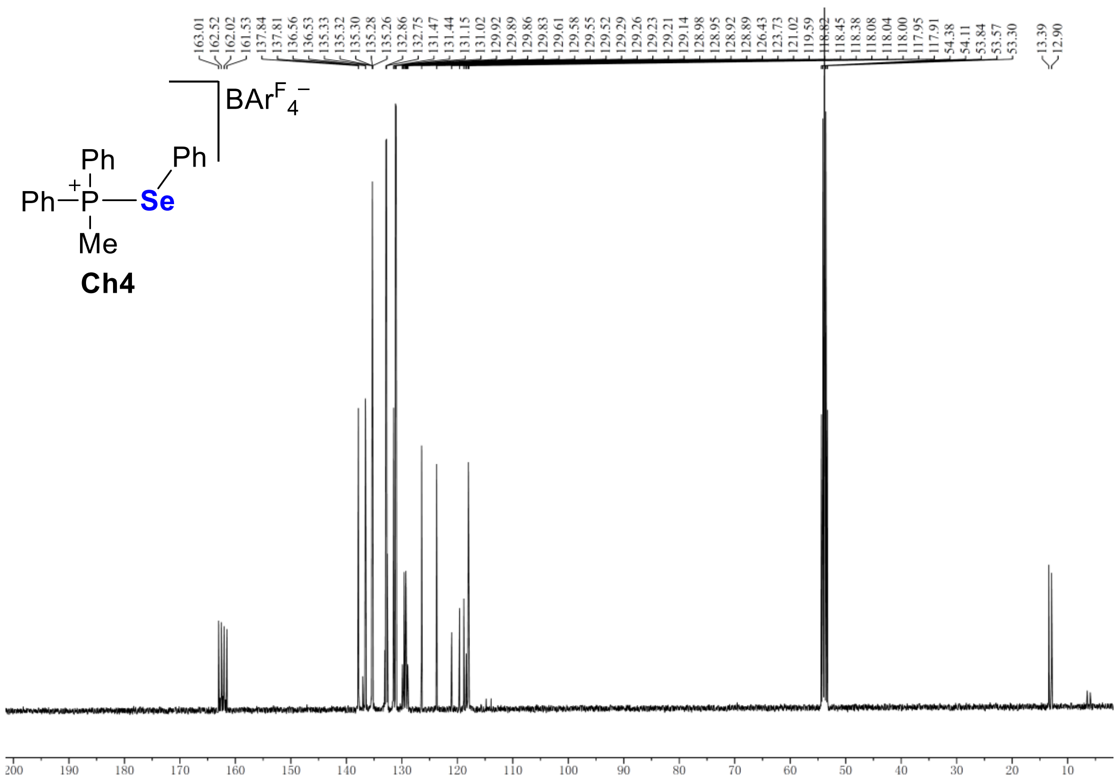

Supplementary Fig. 37 ^{13}**C NMR spectrum of compound Ch4 (CD**$_2$**Cl**$_2$**, 100 MHz, 298K)**

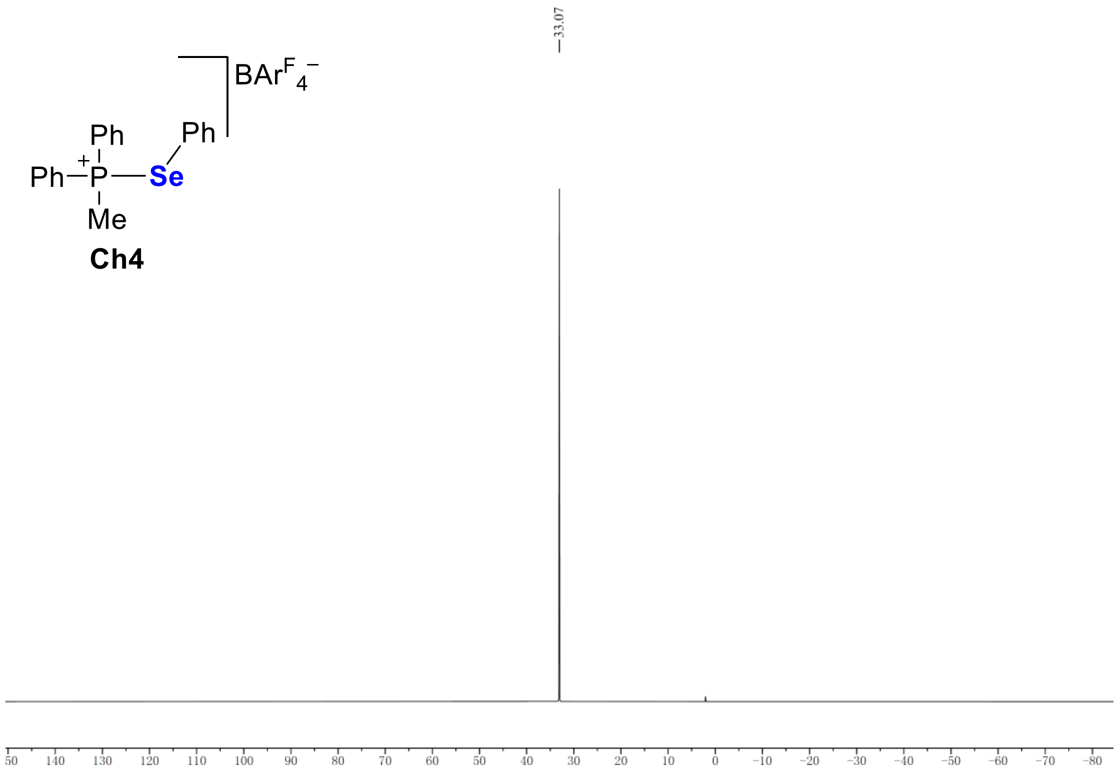

Supplementary Fig. 38 ^{31}**P NMR spectrum of compound Ch4 (CD**$_2$**Cl**$_2$**, 162 MHz, 298K)**

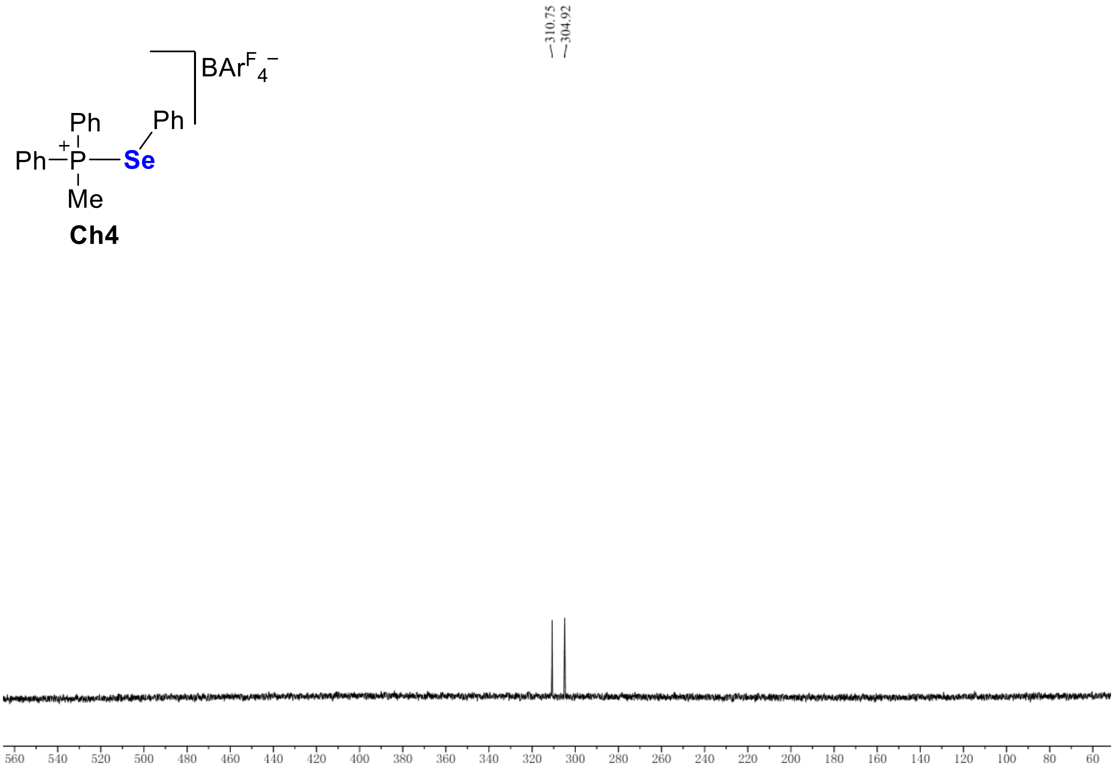

Supplementary Fig. 39 ^{77}Se NMR spectrum of compound Ch4 (CD_2Cl_2, 76 MHz, 298K)

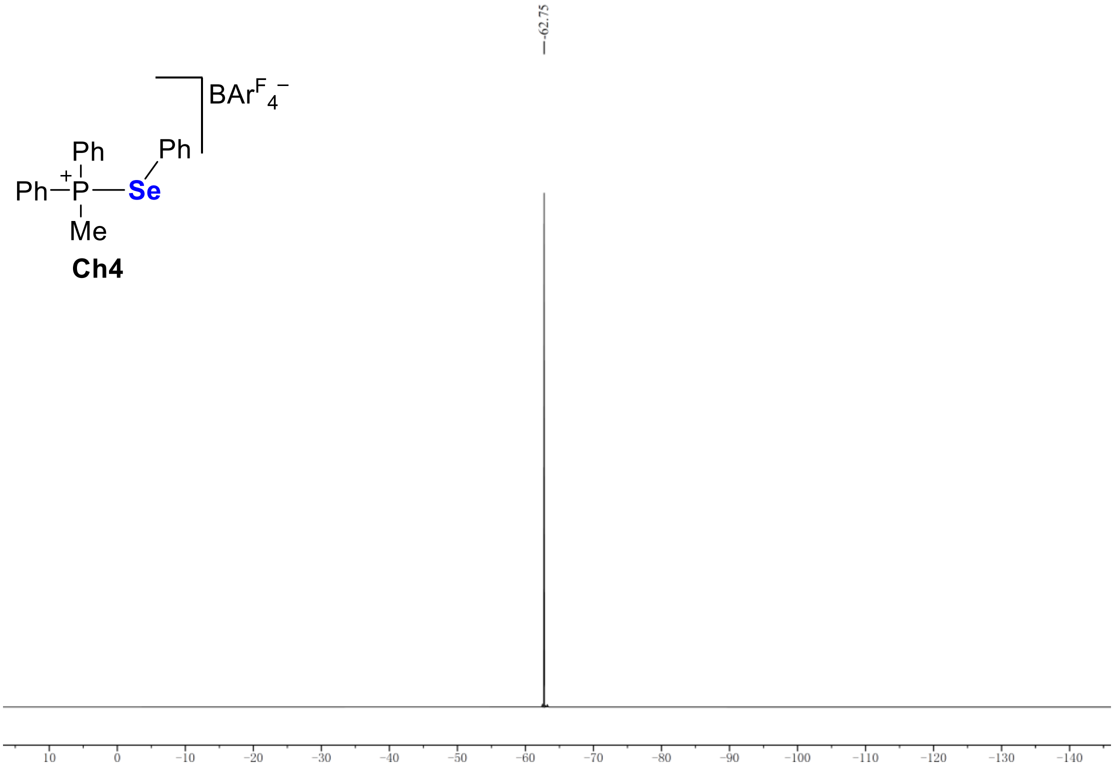

Supplementary Fig. 40 ^{19}F NMR spectrum of compound Ch4 (CD_2Cl_2, 376 MHz, 298K)

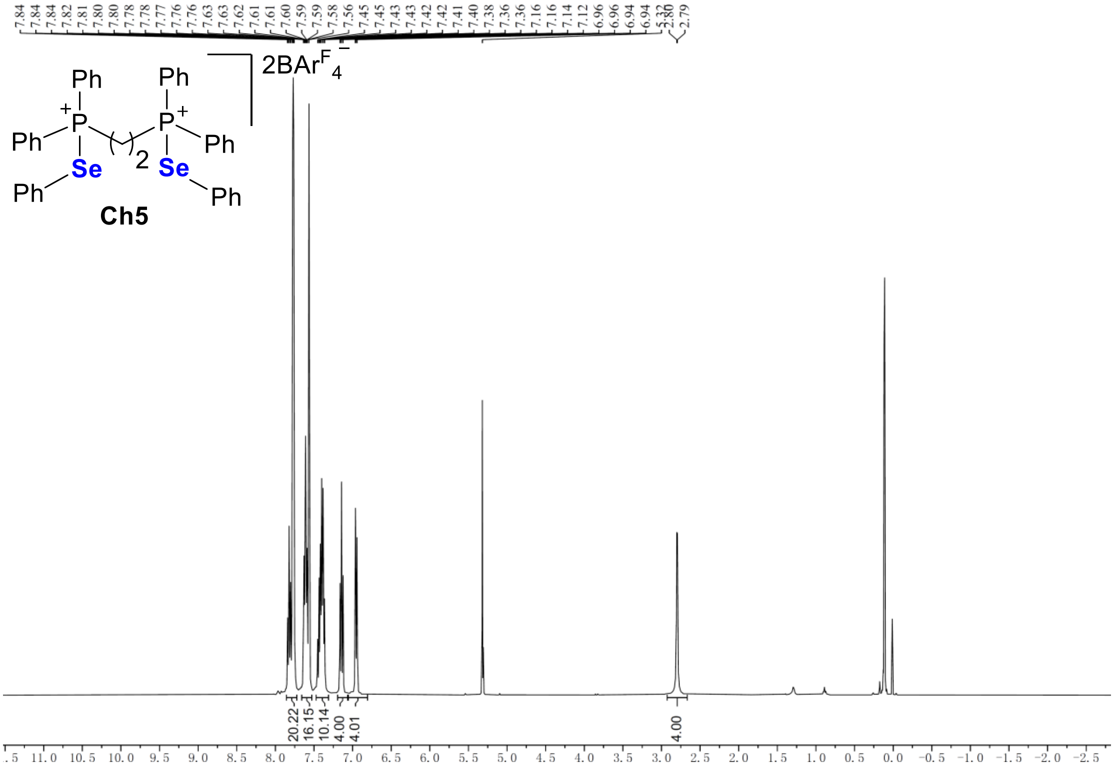

Supplementary Fig. 41 ^{1}H NMR spectrum of compound Ch5 (CD_2Cl_2, 400 MHz, 298K)

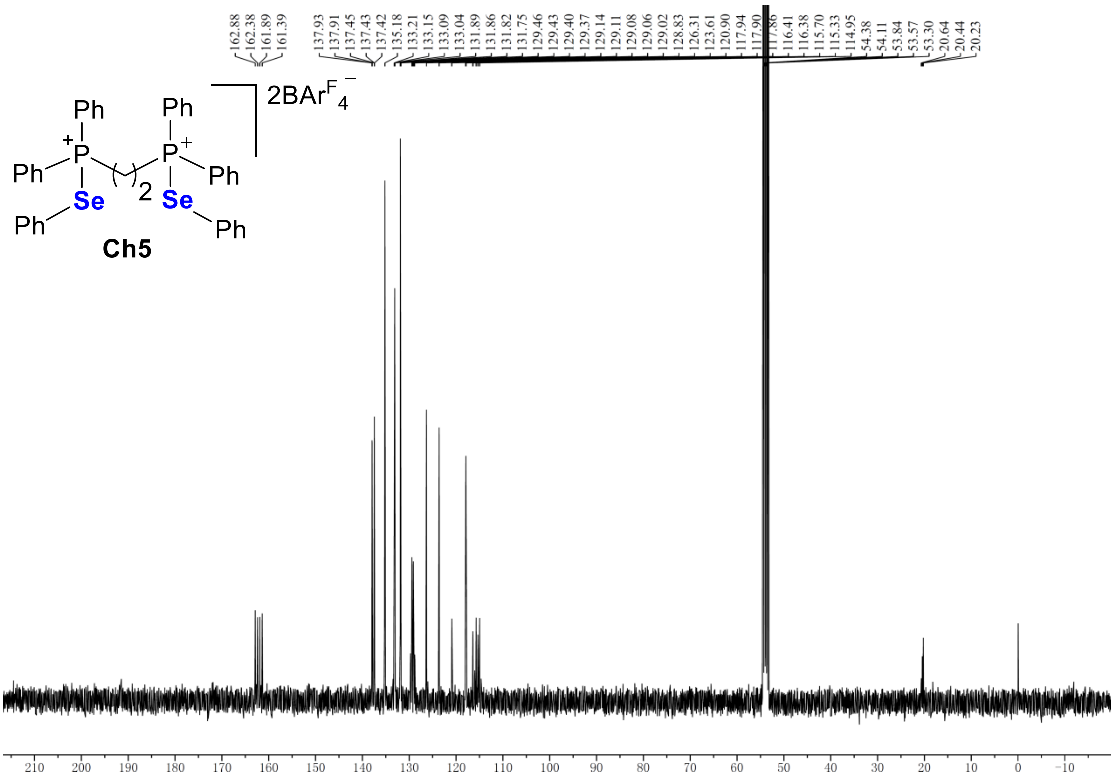

Supplementary Fig. 42 ^{13}C NMR spectrum of compound Ch5 (CD_2Cl_2, 100 MHz, 298K)

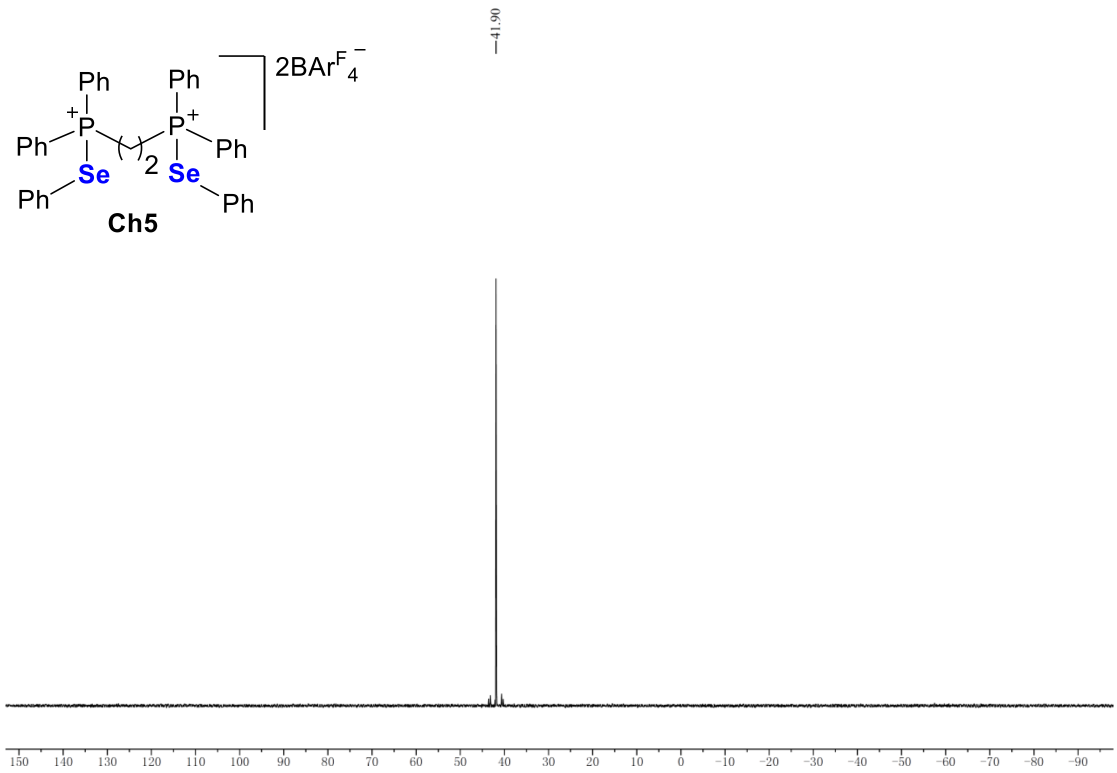

Supplementary Fig. 43 ^{31}P NMR spectrum of compound Ch5 (CD_2Cl_2, 162 MHz, 298K)

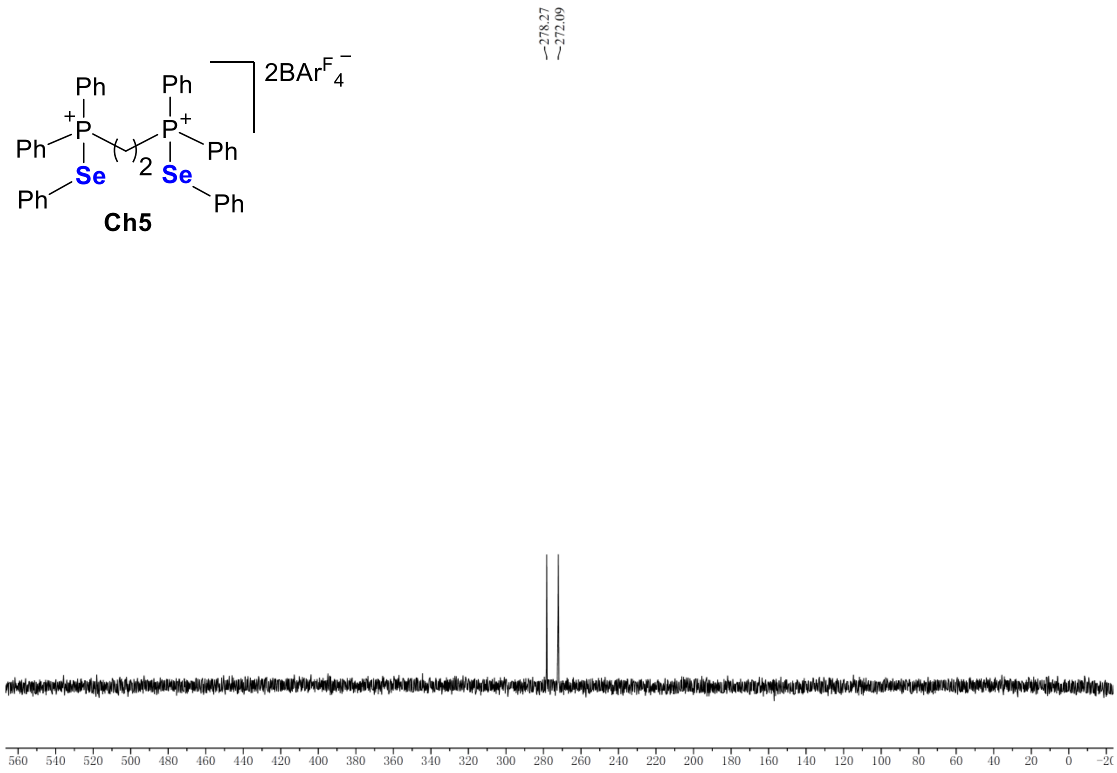

Supplementary Fig. 44 ^{77}Se NMR spectrum of compound Ch5 (CD_2Cl_2, 76 MHz, 298K)

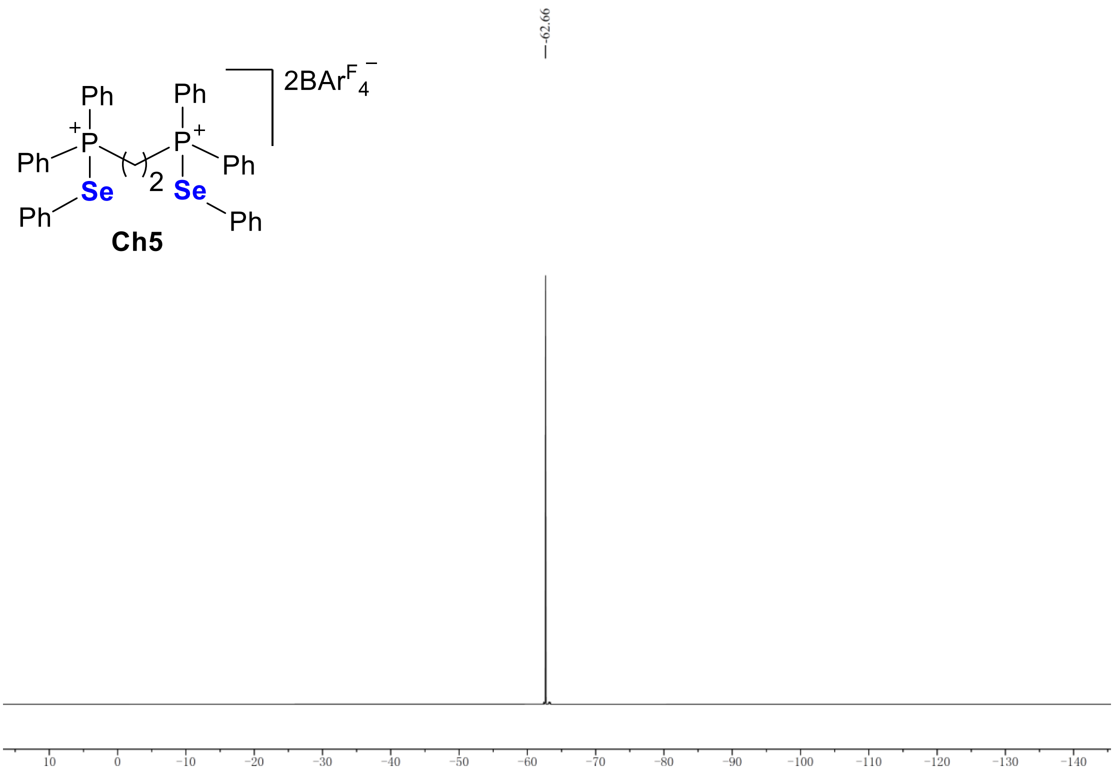

Supplementary Fig. 45 ^{19}F NMR spectrum of compound Ch5 (CD_2Cl_2, 376 MHz, 298K)

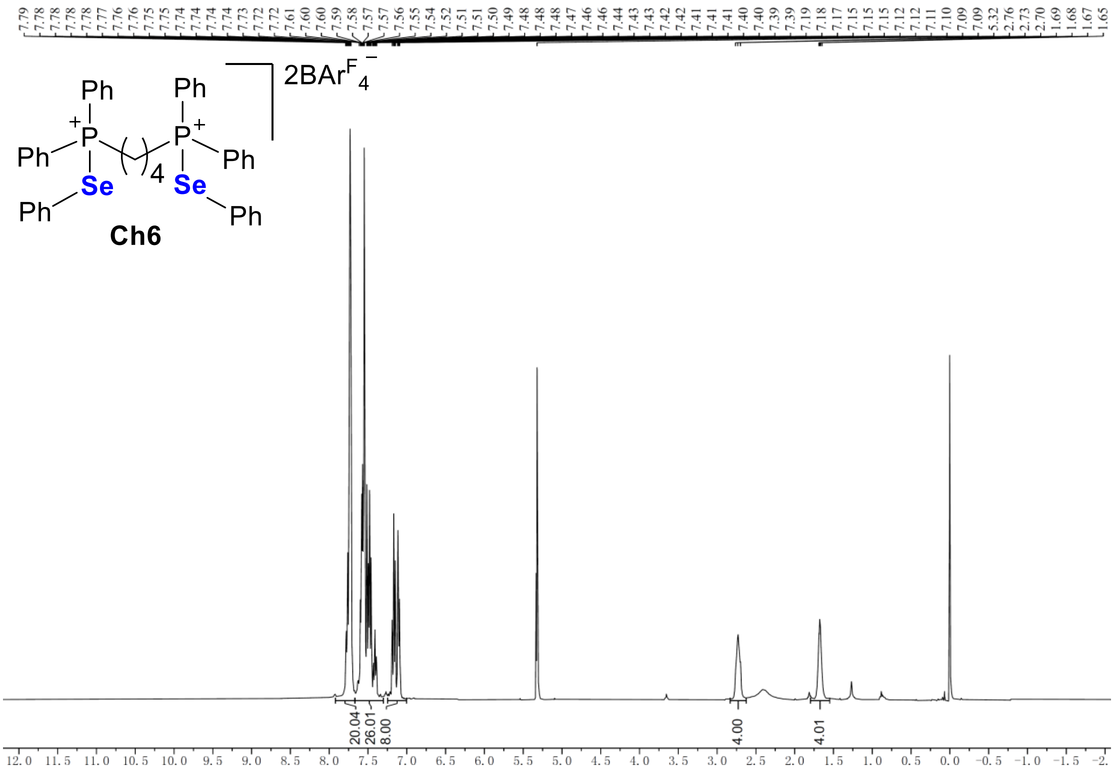

Supplementary Fig. 46 ^{1}H NMR spectrum of compound Ch6 (CD_2Cl_2, 400 MHz, 298K)

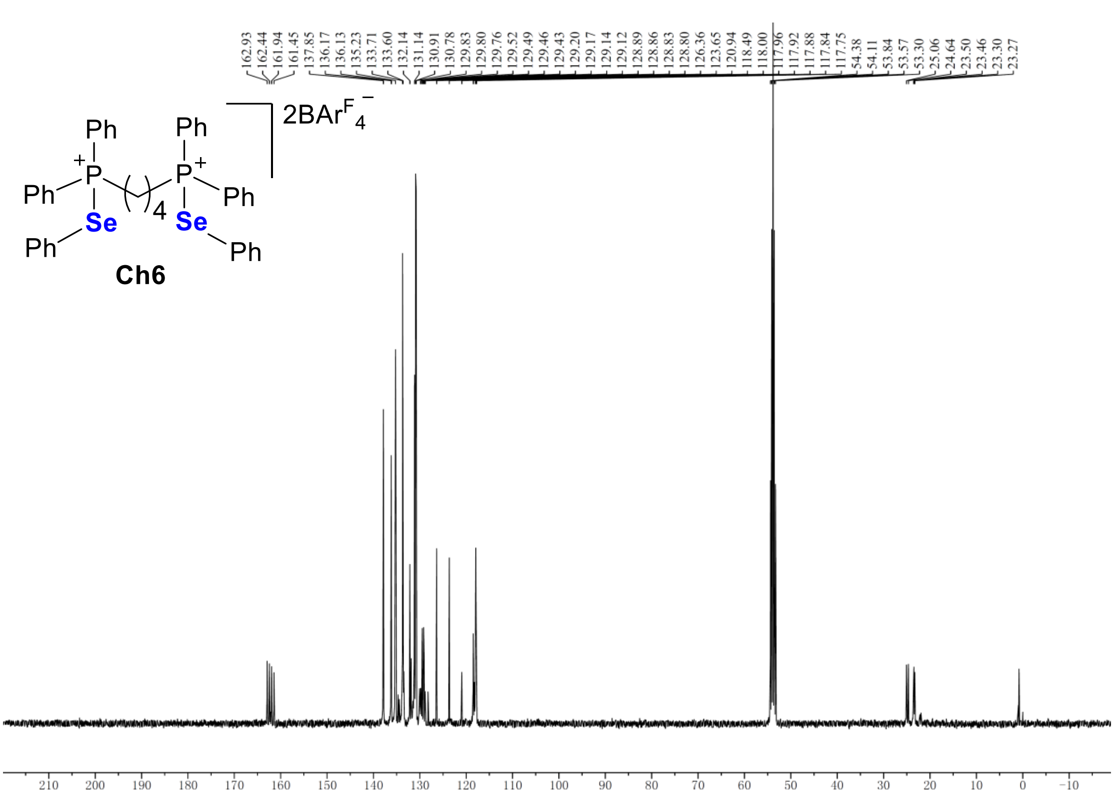

Supplementary Fig. 47 ^{13}C NMR spectrum of compound Ch6 (CD_2Cl_2, 100 MHz, 298K)

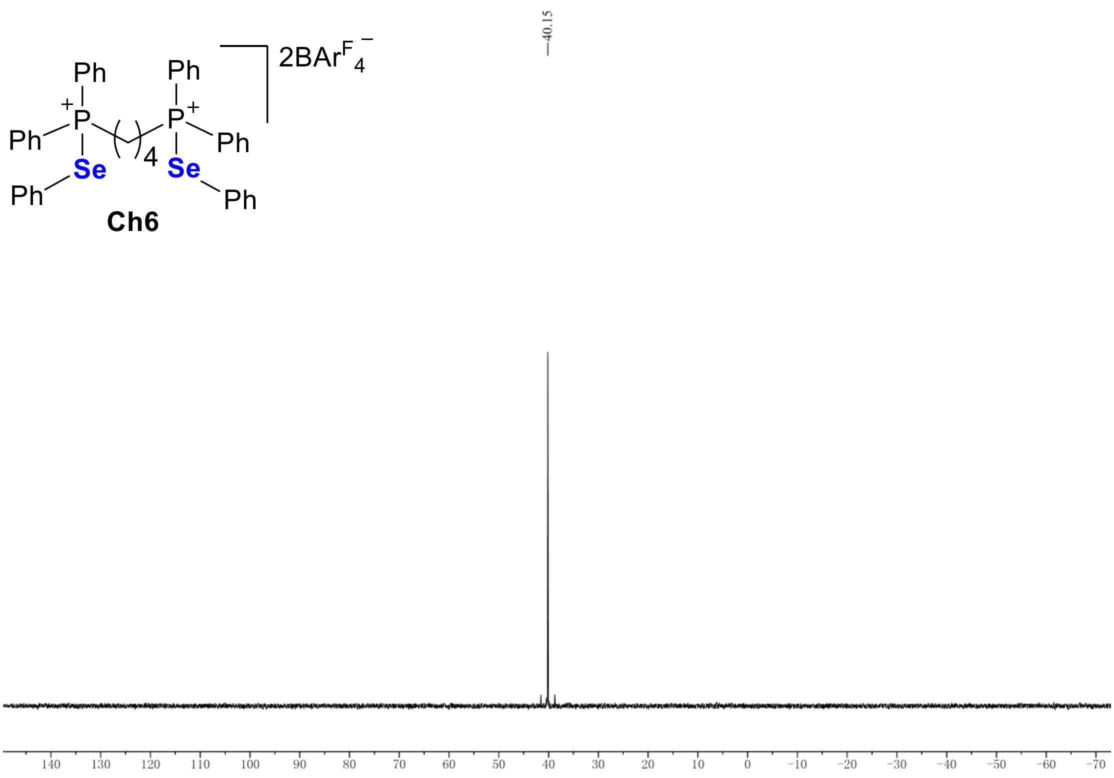

Supplementary Fig. 48 ^{31}P NMR spectrum of compound Ch6 (CD_2Cl_2, 162 MHz, 298K)

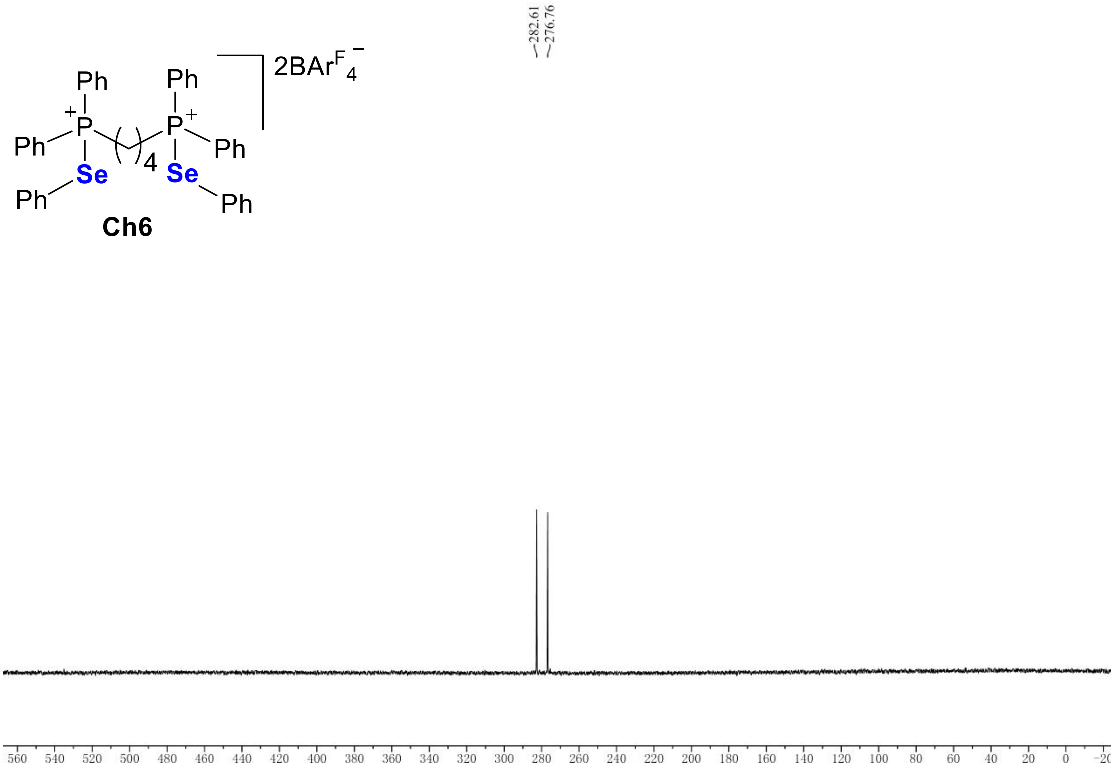

Supplementary Fig. 49 ^{77}Se NMR spectrum of compound Ch6 (CD_2Cl_2, 76 MHz, 298K)

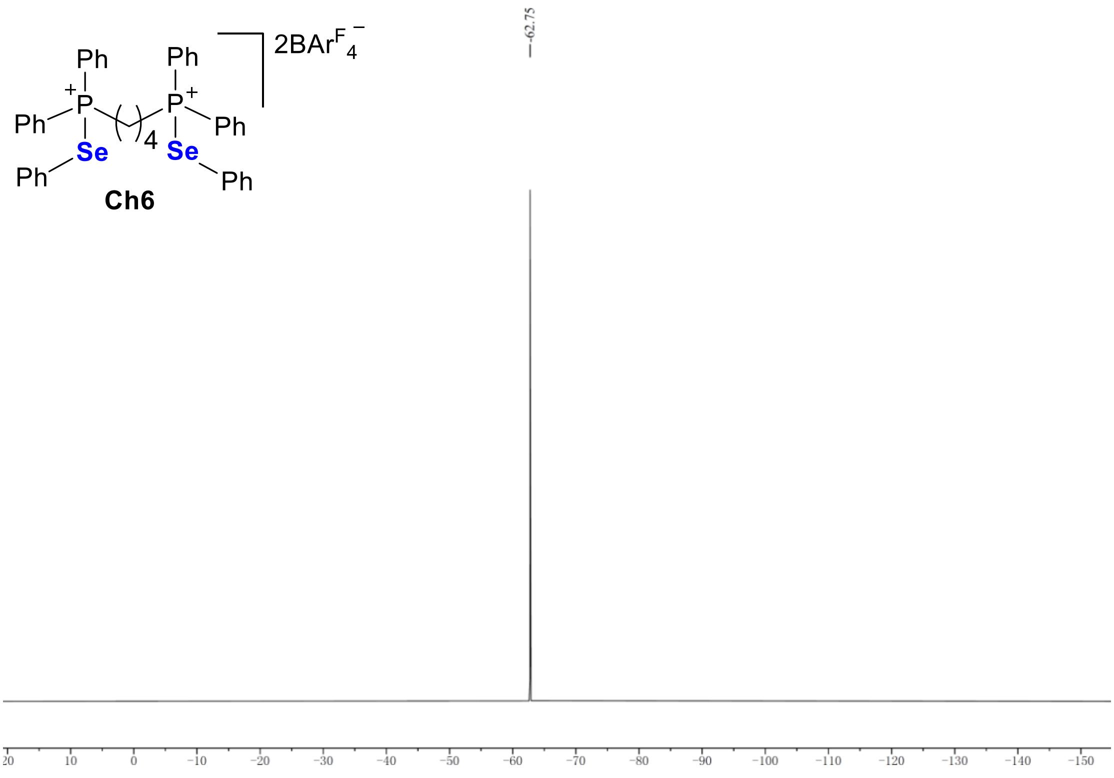

Supplementary Fig. 50 ^{19}F NMR spectrum of compound Ch6 (CD_2Cl_2, 376 MHz, 298K)

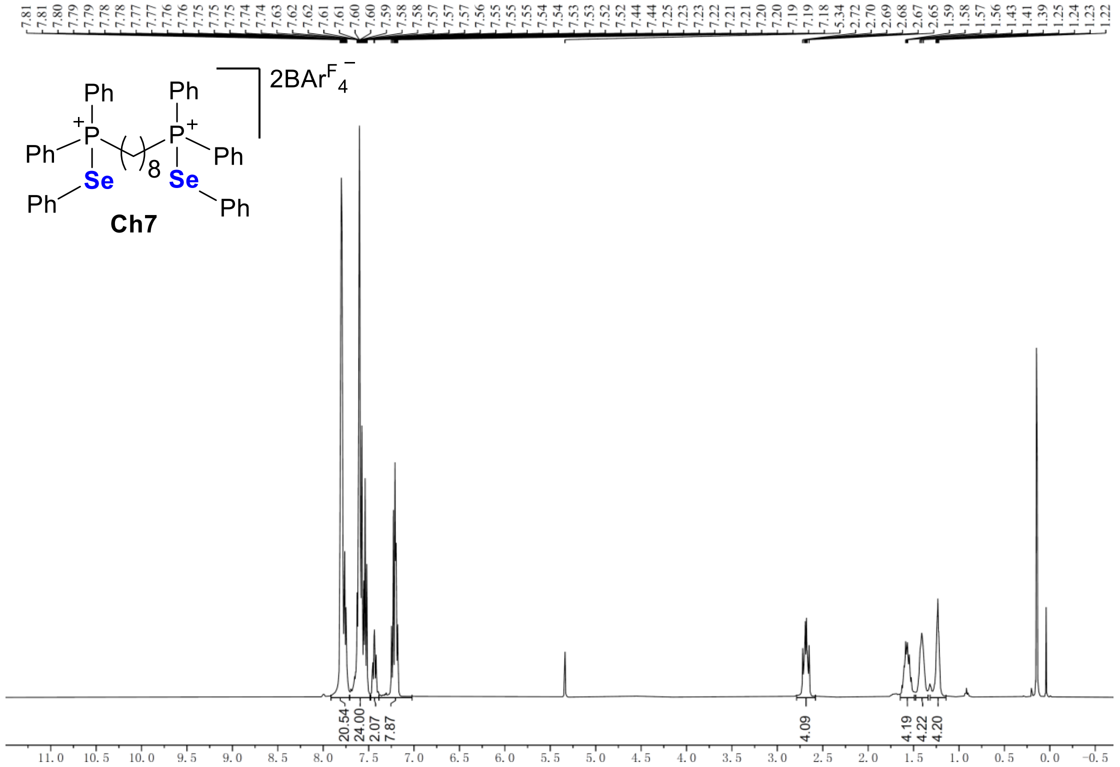

Supplementary Fig. 51 ^{1}H NMR spectrum of compound Ch7 (CD_2Cl_2, 400 MHz, 298K)

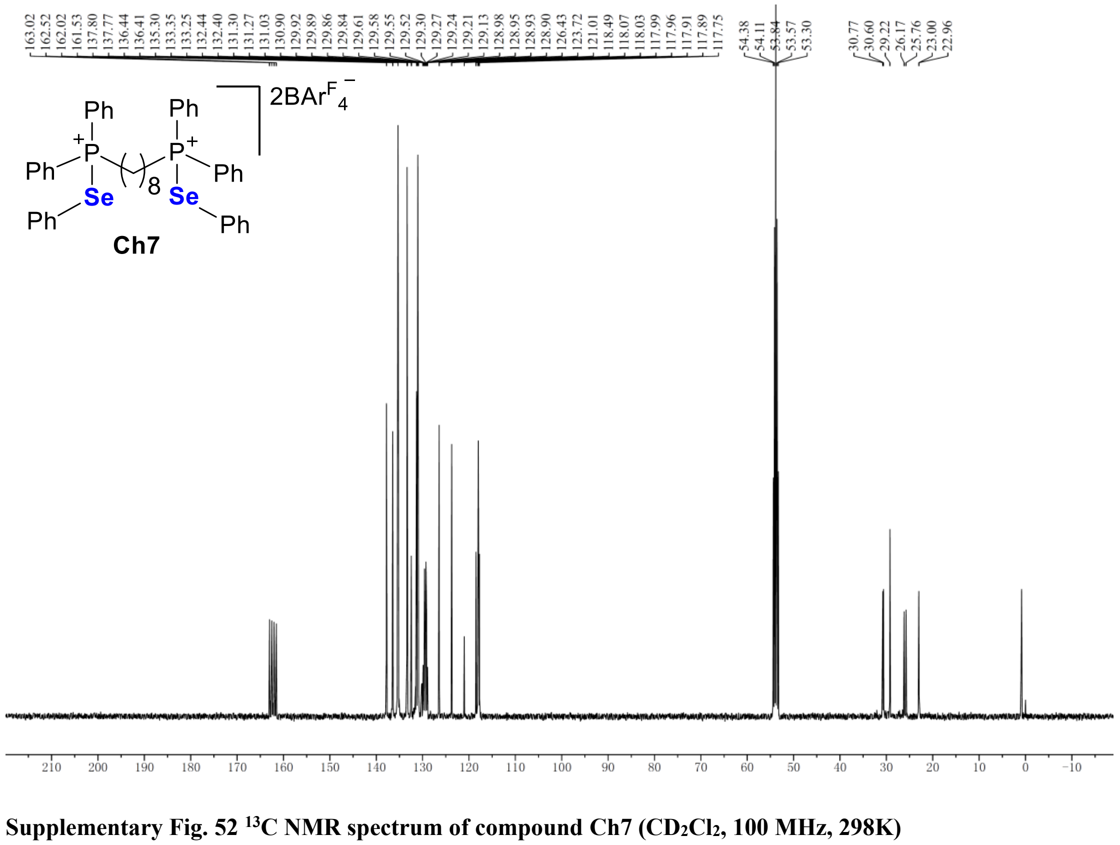

Supplementary Fig. 52 ^{13}C NMR spectrum of compound Ch7 (CD_2Cl_2, 100 MHz, 298K)

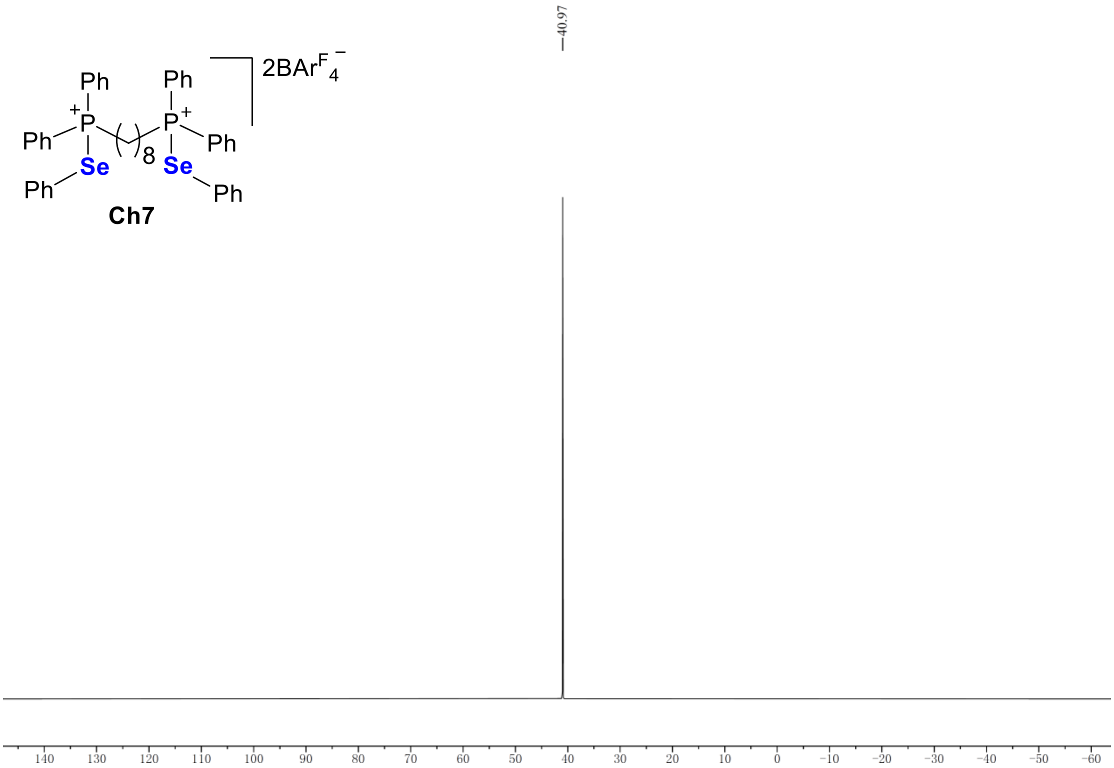

Supplementary Fig. 53 ^{31}P NMR spectrum of compound Ch7 (CD_2Cl_2, 162 MHz, 298K)

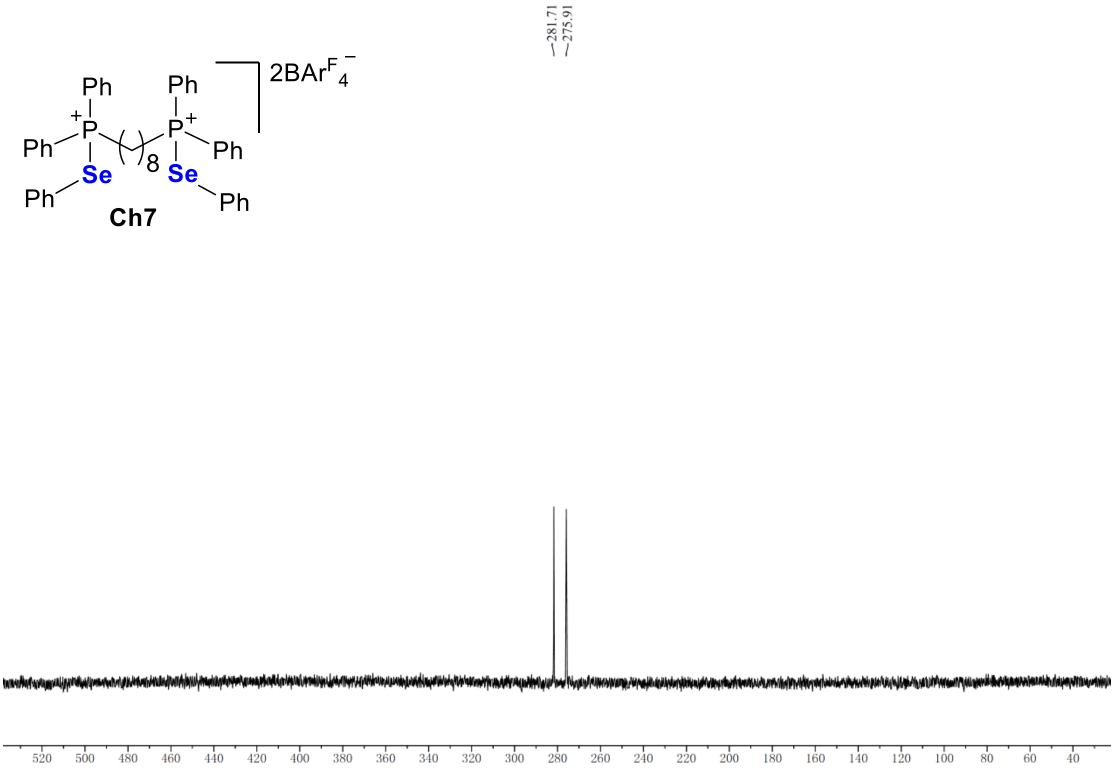

Supplementary Fig. 54 ^{77}Se NMR spectrum of compound Ch7 (CD_2Cl_2, 76 MHz, 298K)

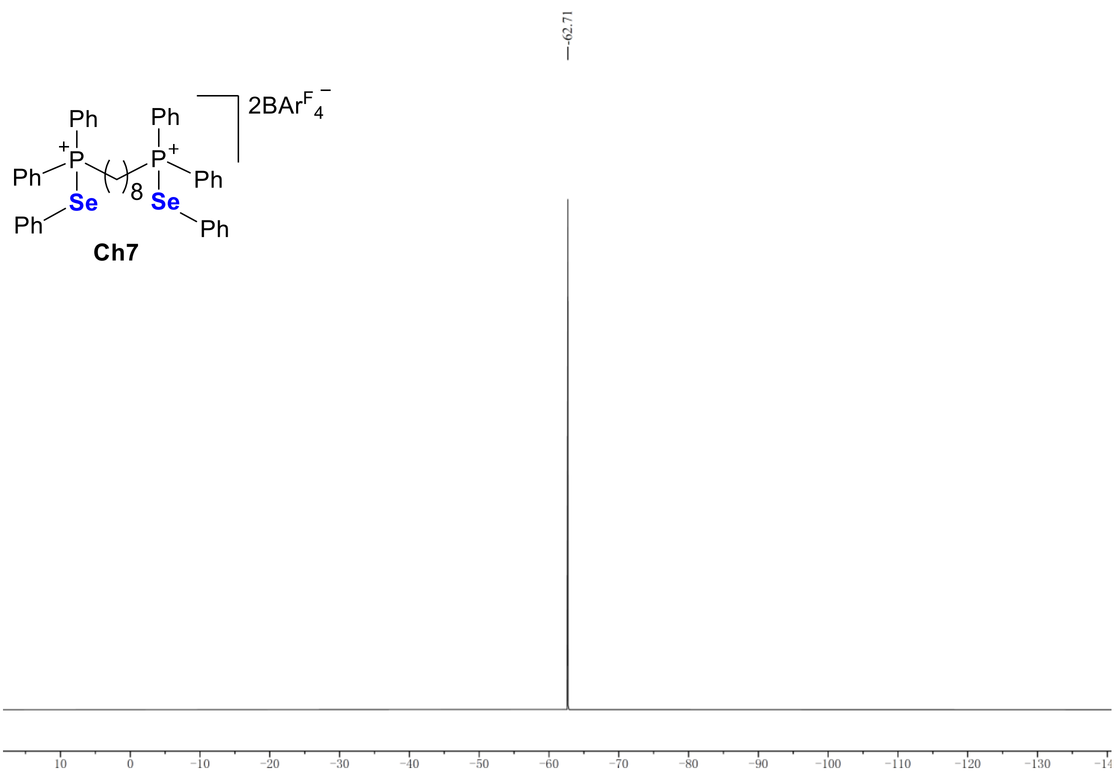

Supplementary Fig. 55 ^{19}F NMR spectrum of compound Ch7 (CD_2Cl_2, 376 MHz, 298K)

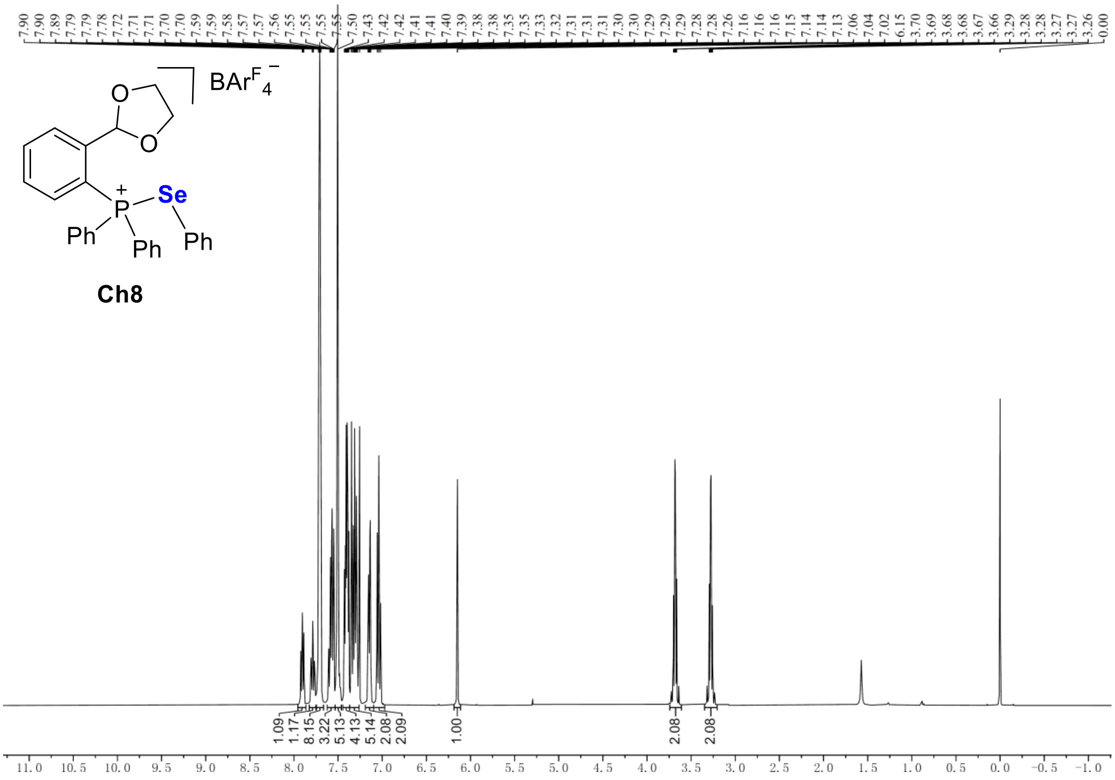

Supplementary Fig. 56 ^{1}H NMR spectrum of compound Ch8 ($CDCl_3$, 400 MHz, 298K)

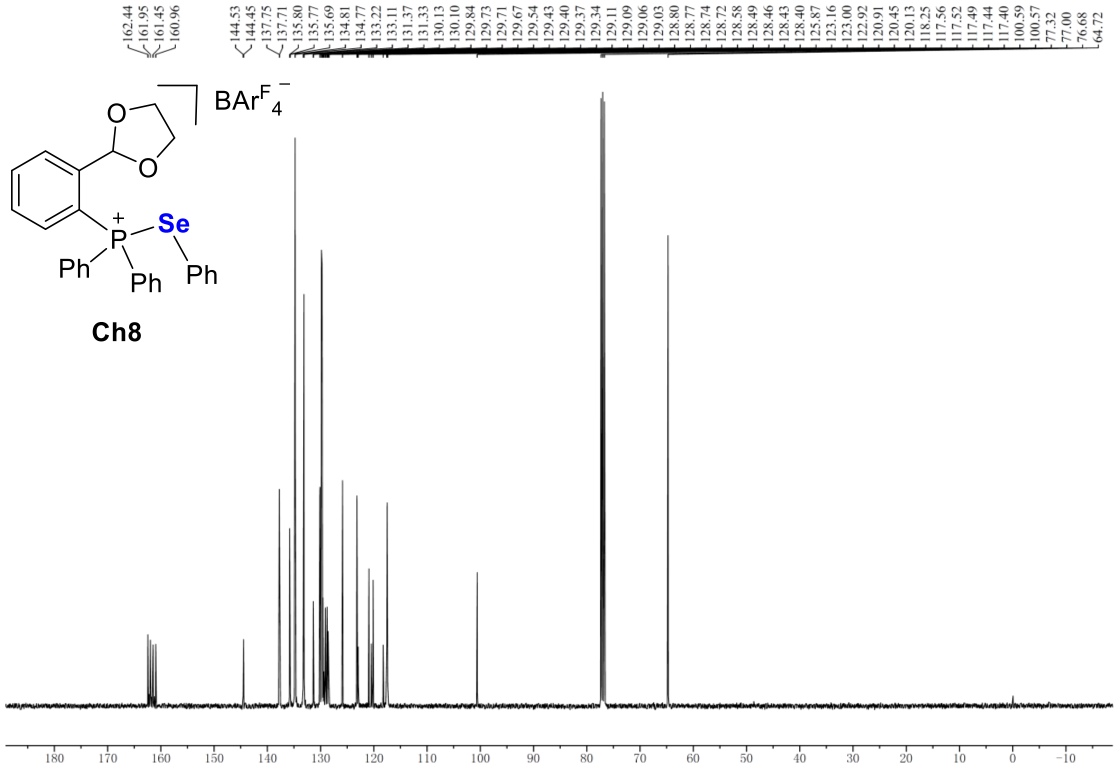

Supplementary Fig. 57 ^{13}C NMR spectrum of compound Ch8 ($CDCl_3$, 100 MHz, 298K)

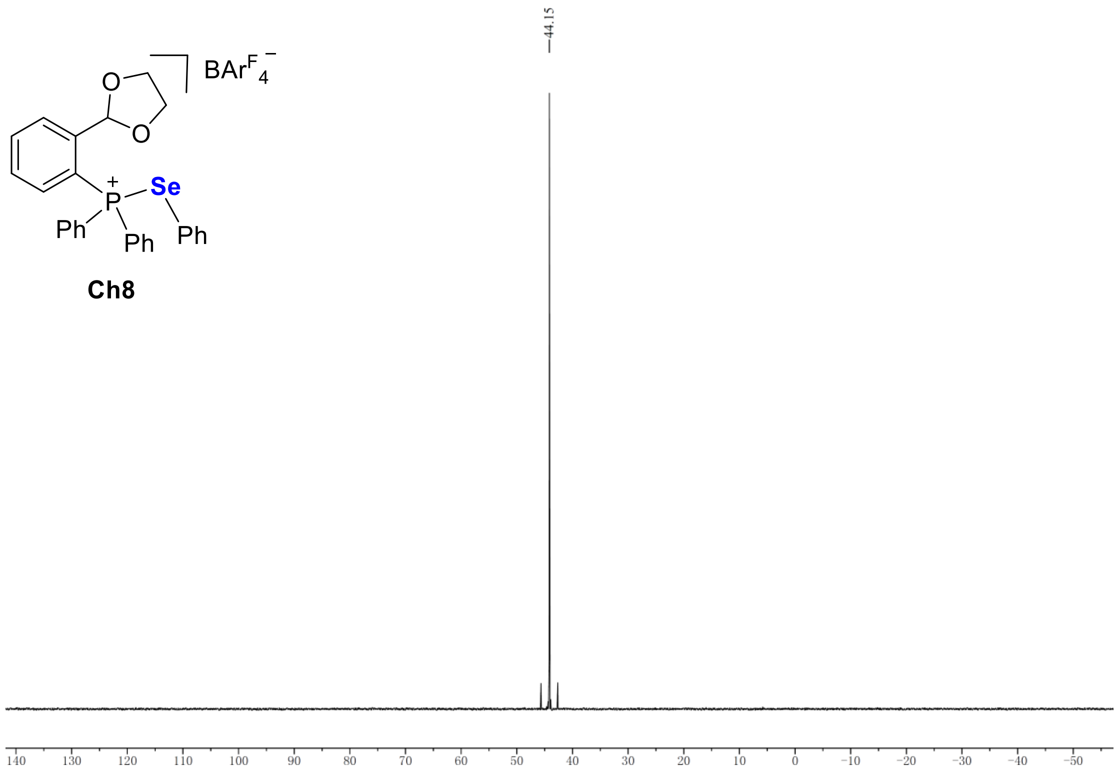

Supplementary Fig. 58 ^{31}P NMR spectrum of compound Ch8 ($CDCl_3$, 162 MHz, 298K)

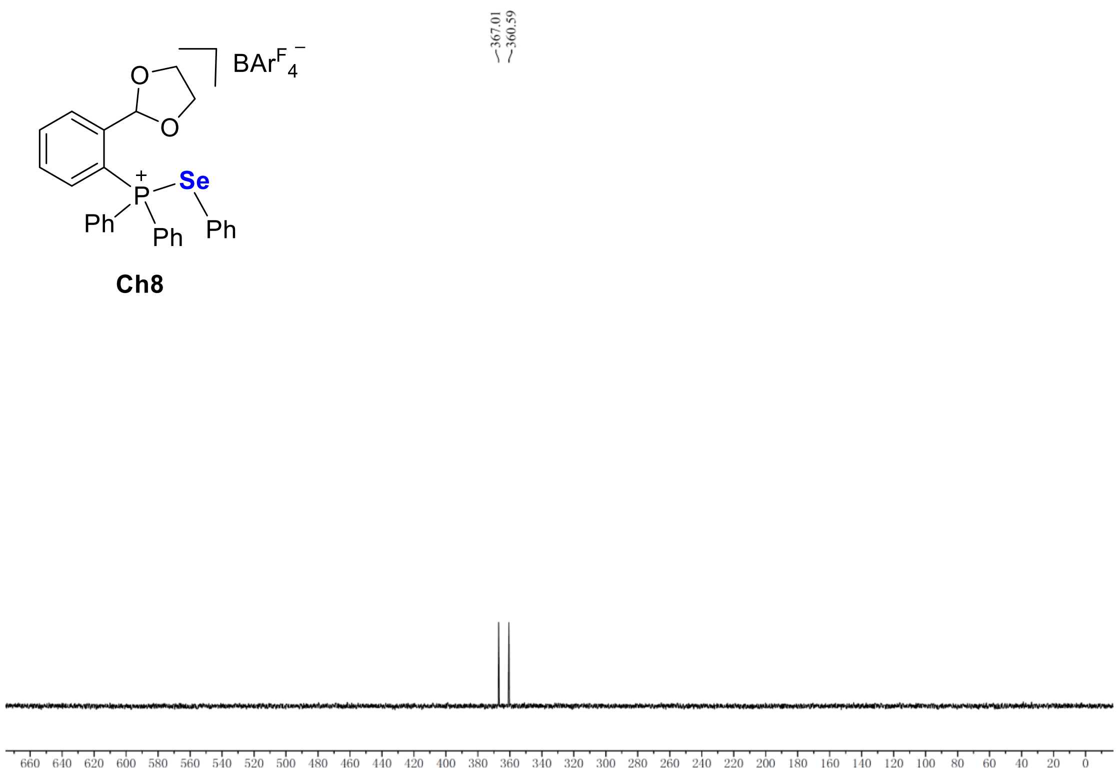

Supplementary Fig. 59 ^{77}Se NMR spectrum of compound Ch8 ($CDCl_3$, 76 MHz, 298K)

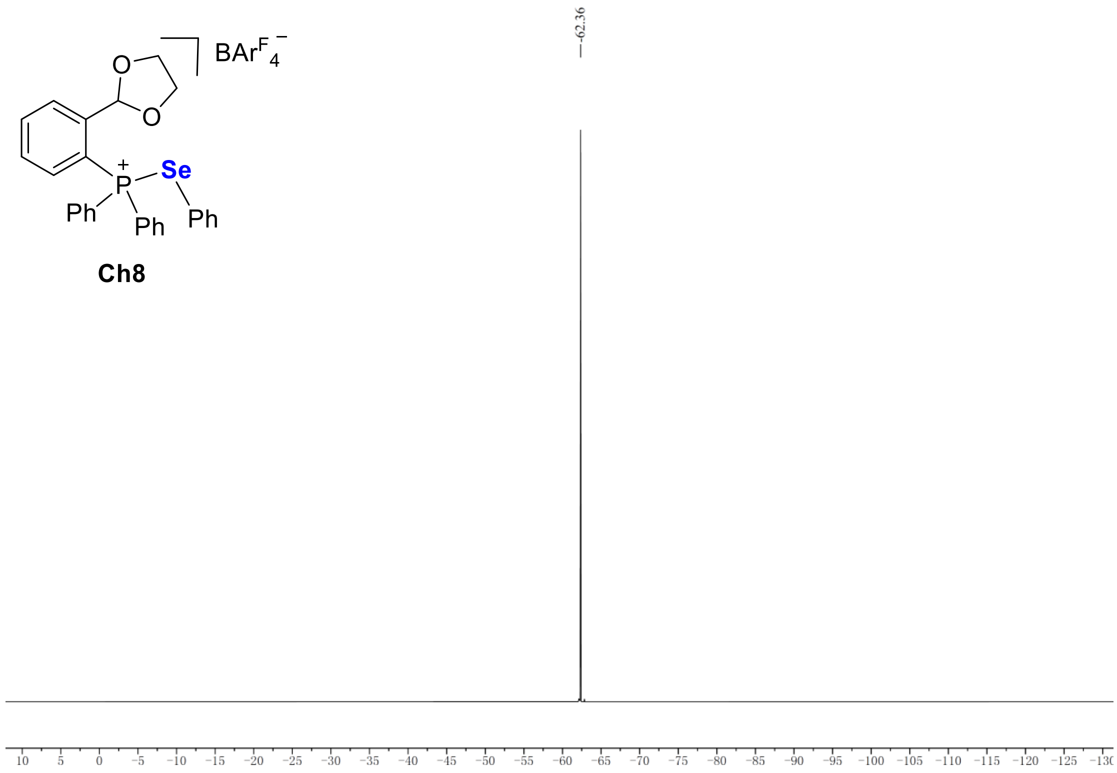

Supplementary Fig. 60 ^{19}F NMR spectrum of compound Ch8 ($CDCl_3$, 376 MHz, 298K)

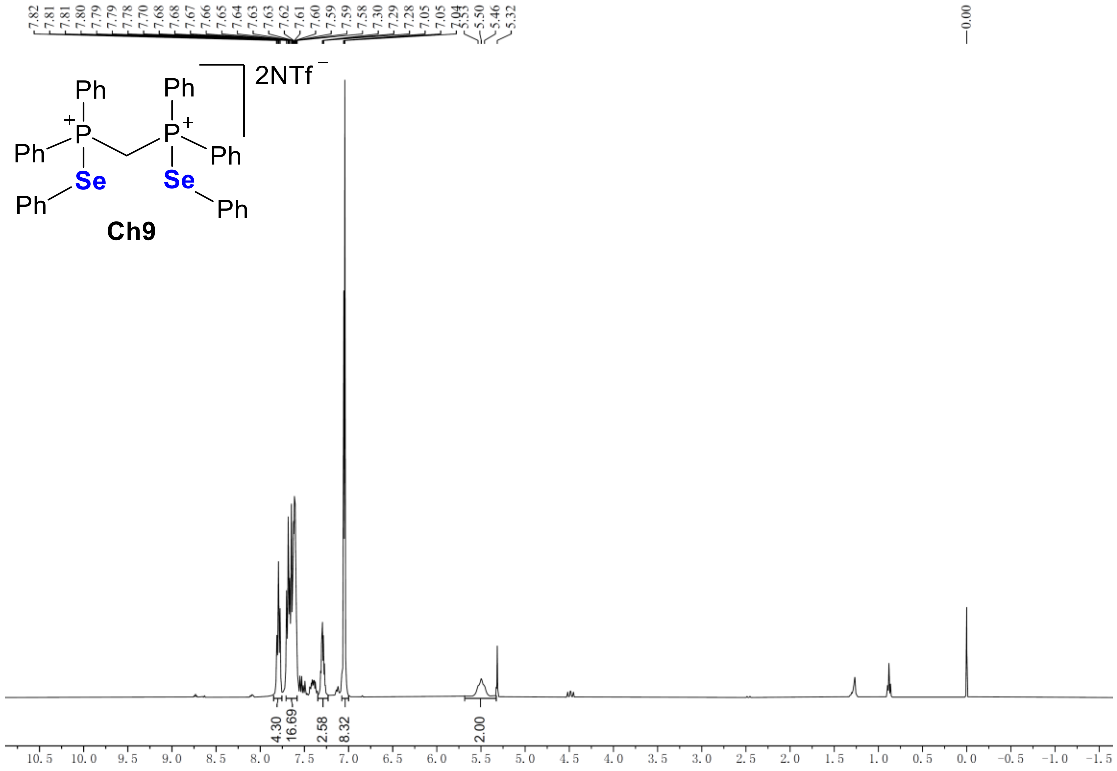

Supplementary Fig. 61 ^{1}H NMR spectrum of compound Ch9 (CD_2Cl_2, 400 MHz, 298K)

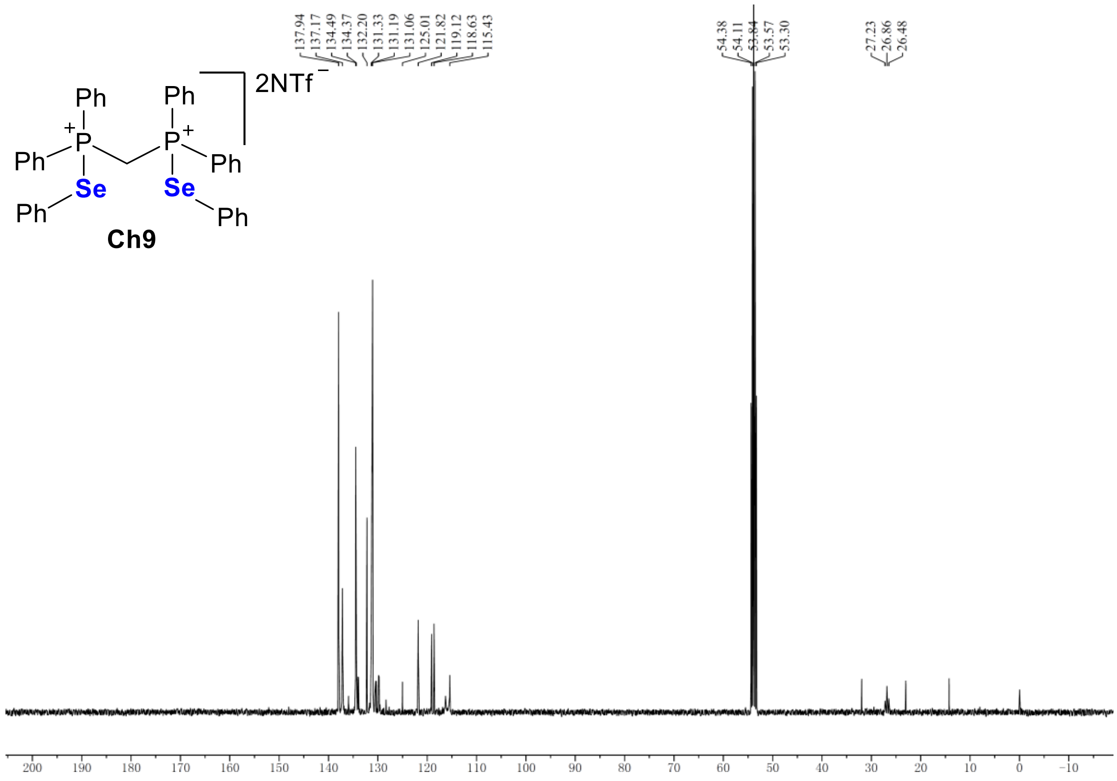

Supplementary Fig. 62 ^{13}C NMR spectrum of compound Ch9 (CD_2Cl_2, 100 MHz, 298K)

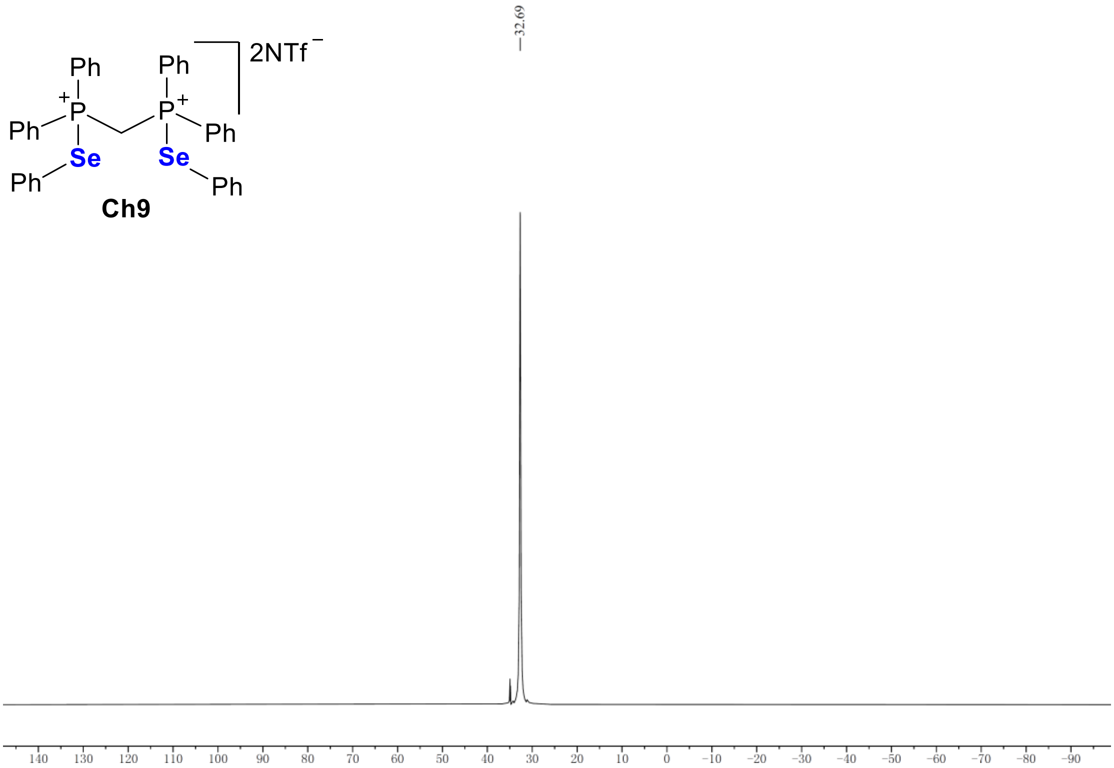

Supplementary Fig. 63 ^{31}P NMR spectrum of compound Ch9 (CD_2Cl_2, 162 MHz, 298K)

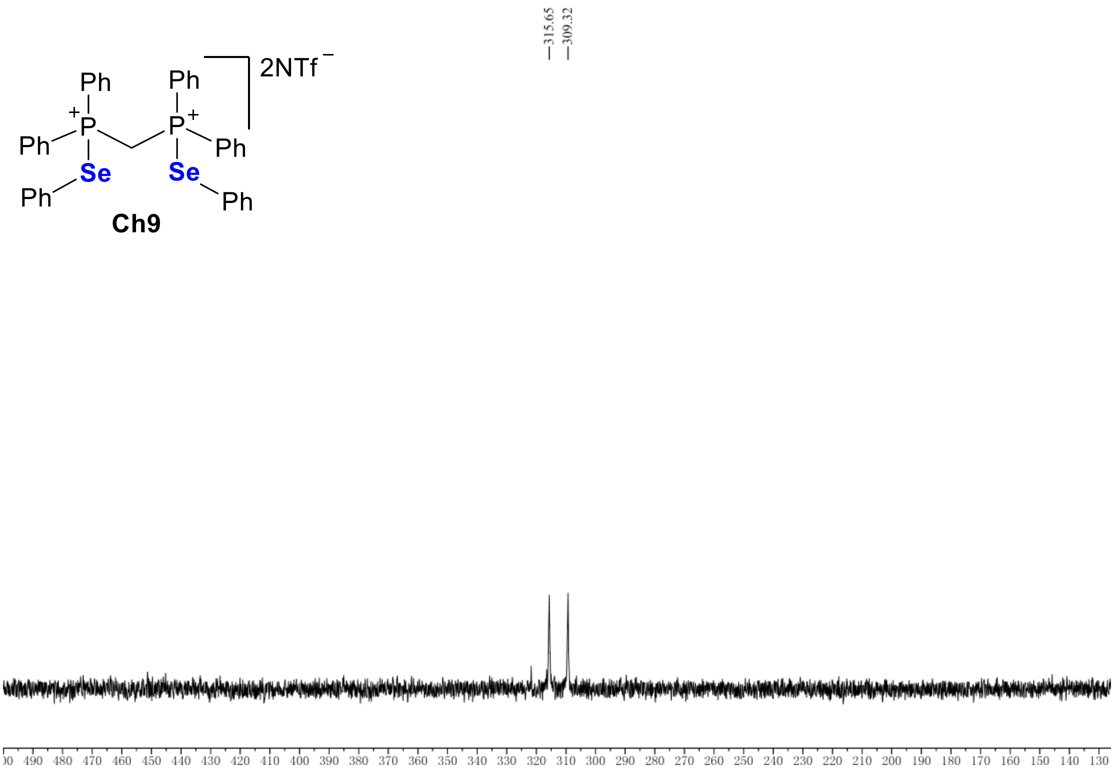

Supplementary Fig. 64 ^{77}Se NMR spectrum of compound Ch9 (CD_2Cl_2, 76 MHz, 298K)

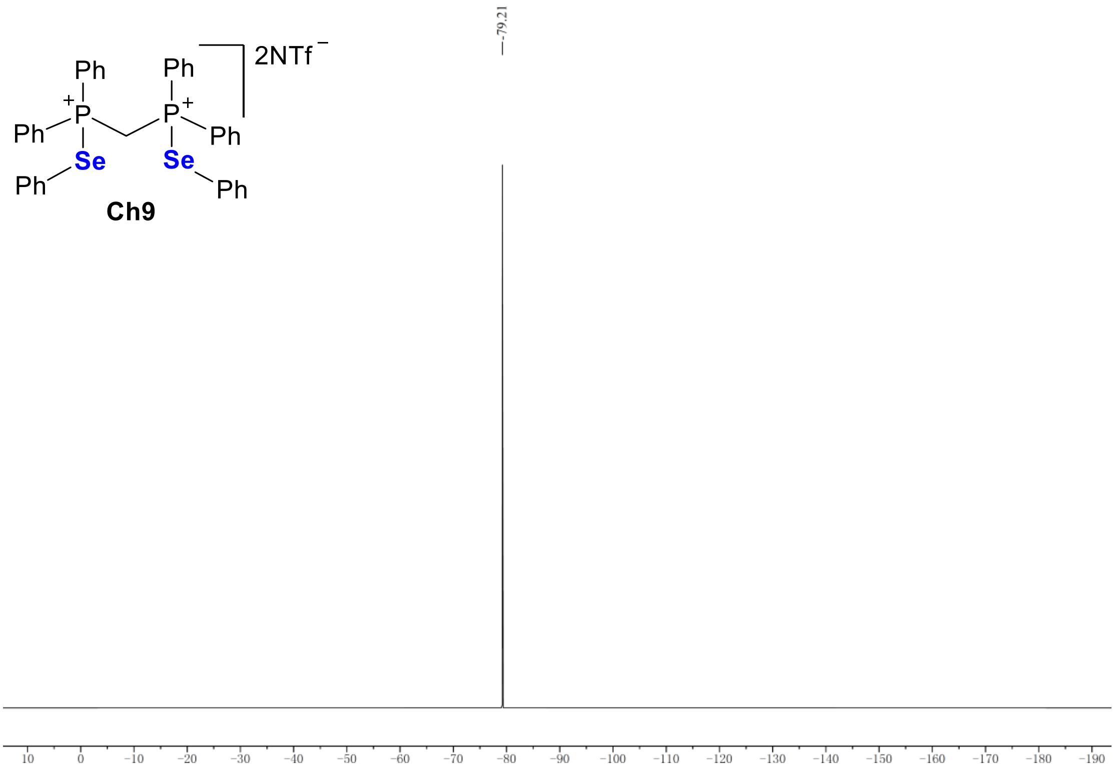

Supplementary Fig. 65 ^{19}F NMR spectrum of compound Ch9 (CD_2Cl_2, 376 MHz, 298K)

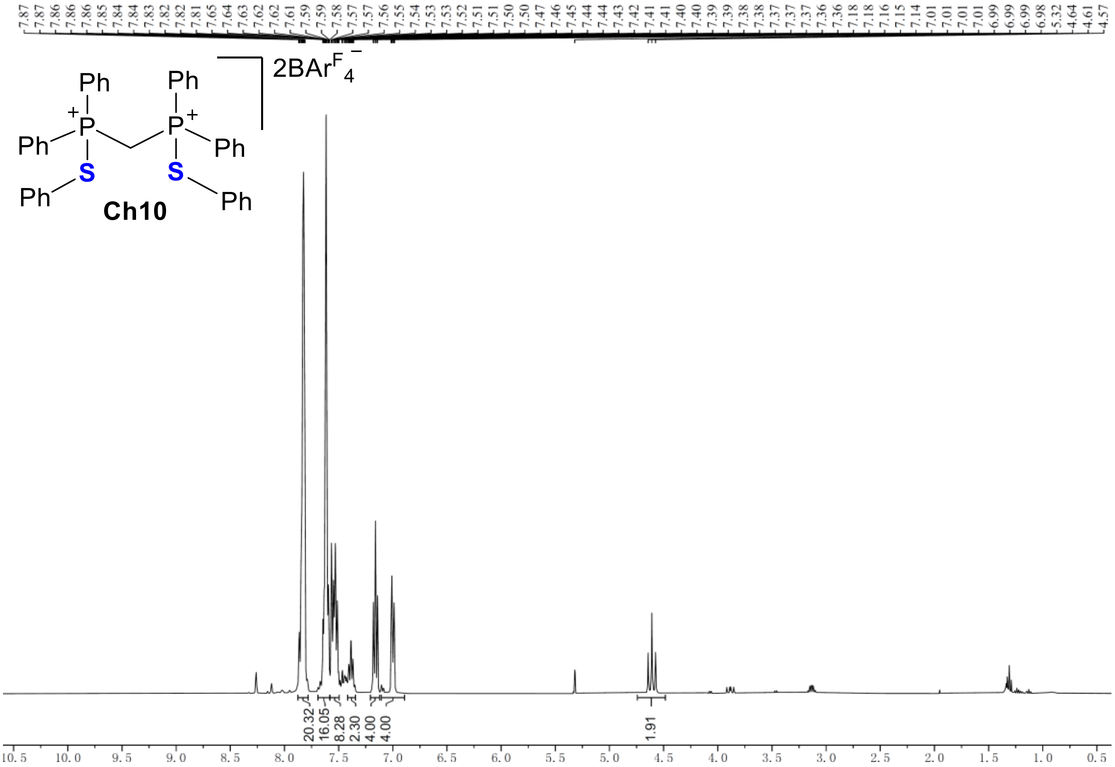

Supplementary Fig. 66 ^{1}H NMR spectrum of compound Ch10 (CD_2Cl_2, 400 MHz, 298K)

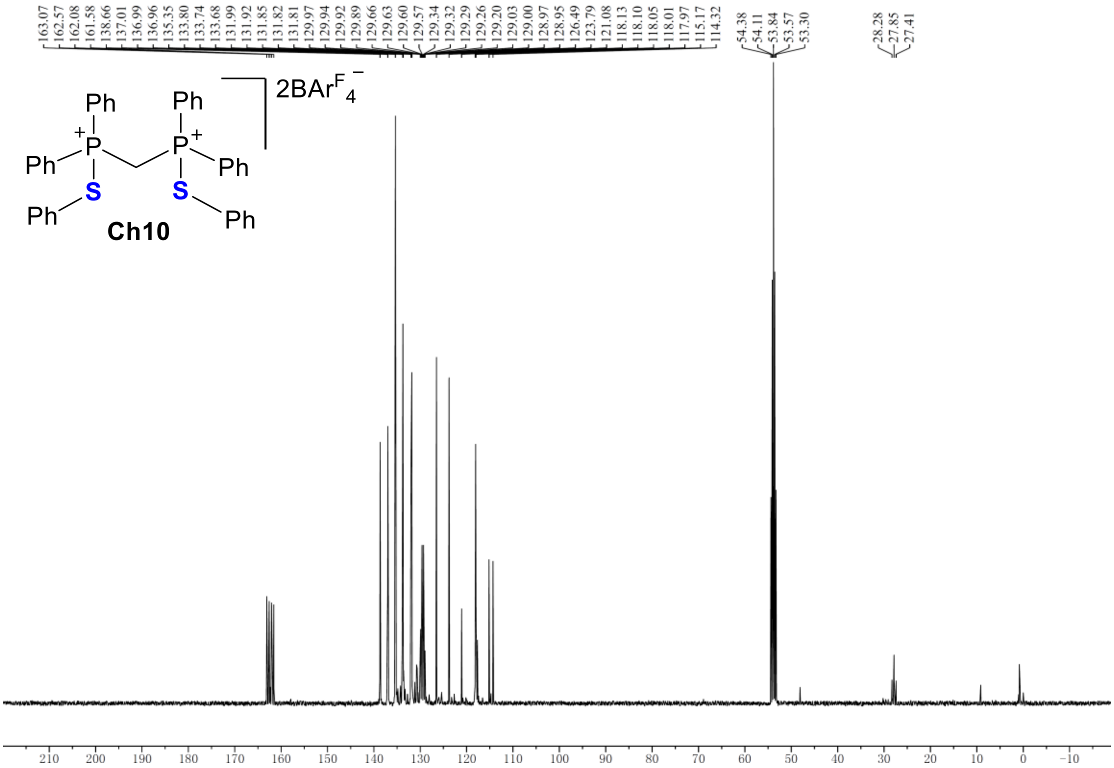

Supplementary Fig. 67 ^{13}C NMR spectrum of compound Ch10 (CD_2Cl_2, 100 MHz, 298K)

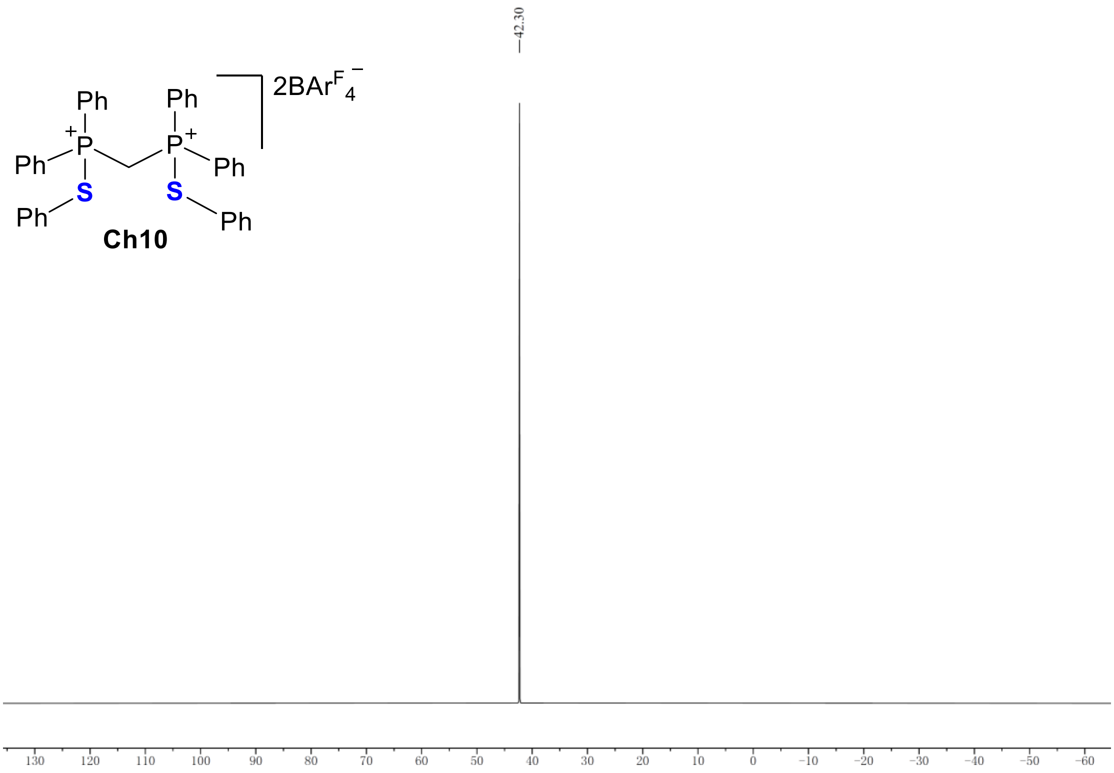

Supplementary Fig. 68 ^{31}P NMR spectrum of compound Ch10 (CD_2Cl_2, 162 MHz, 298K)

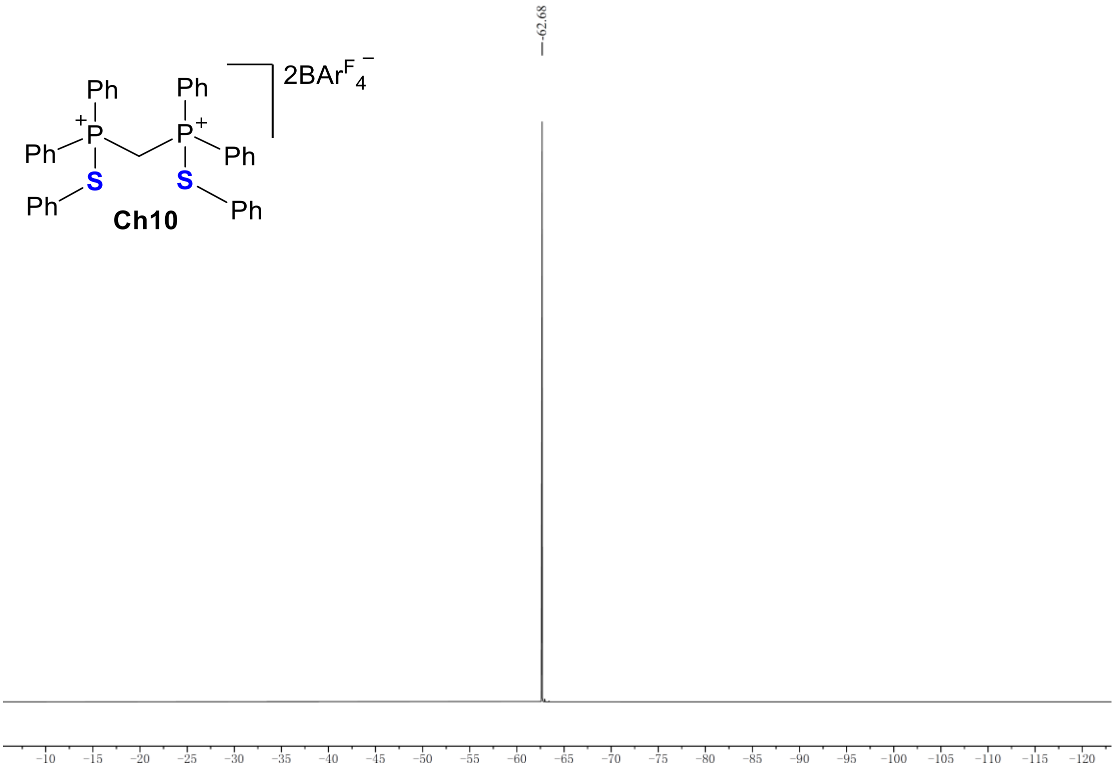

Supplementary Fig. 69 ^{19}F NMR spectrum of compound Ch10 (CD_2Cl_2, 376 MHz, 298K)

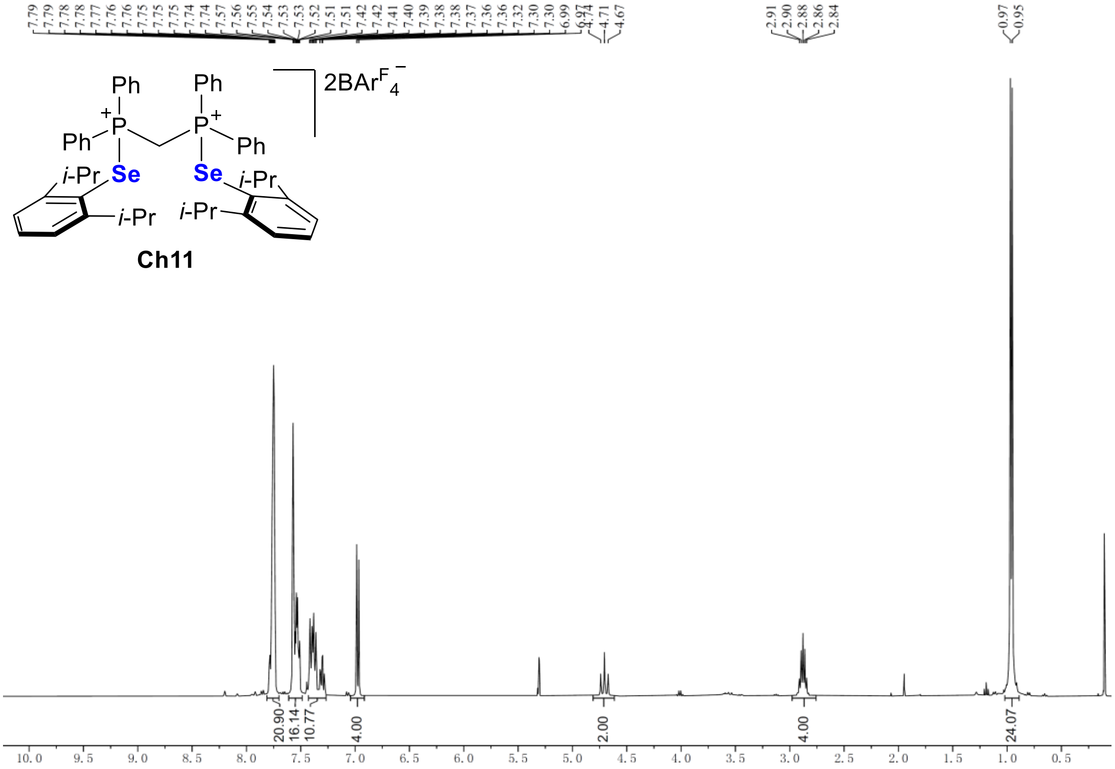

Supplementary Fig. 70 ^{1}H NMR spectrum of compound Ch11 (CD_2Cl_2, 400 MHz, 298K)

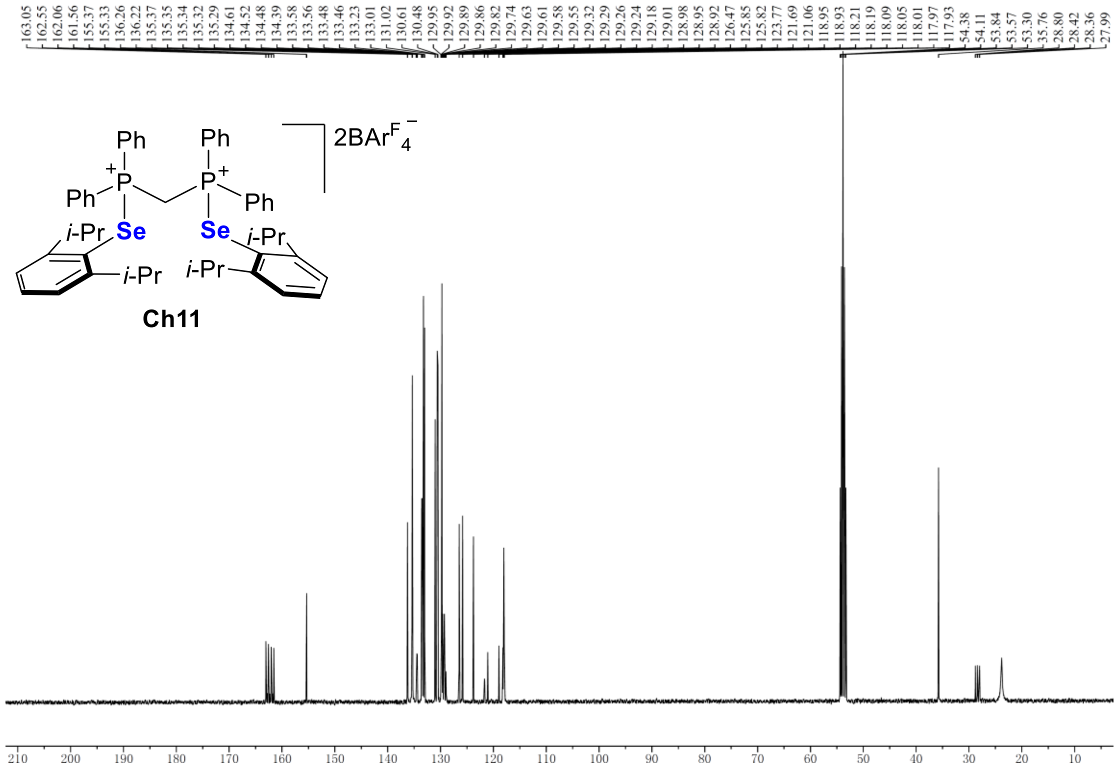

Supplementary Fig. 71 ^{13}C NMR spectrum of compound Ch11 (CD_2Cl_2, 100 MHz, 298K)

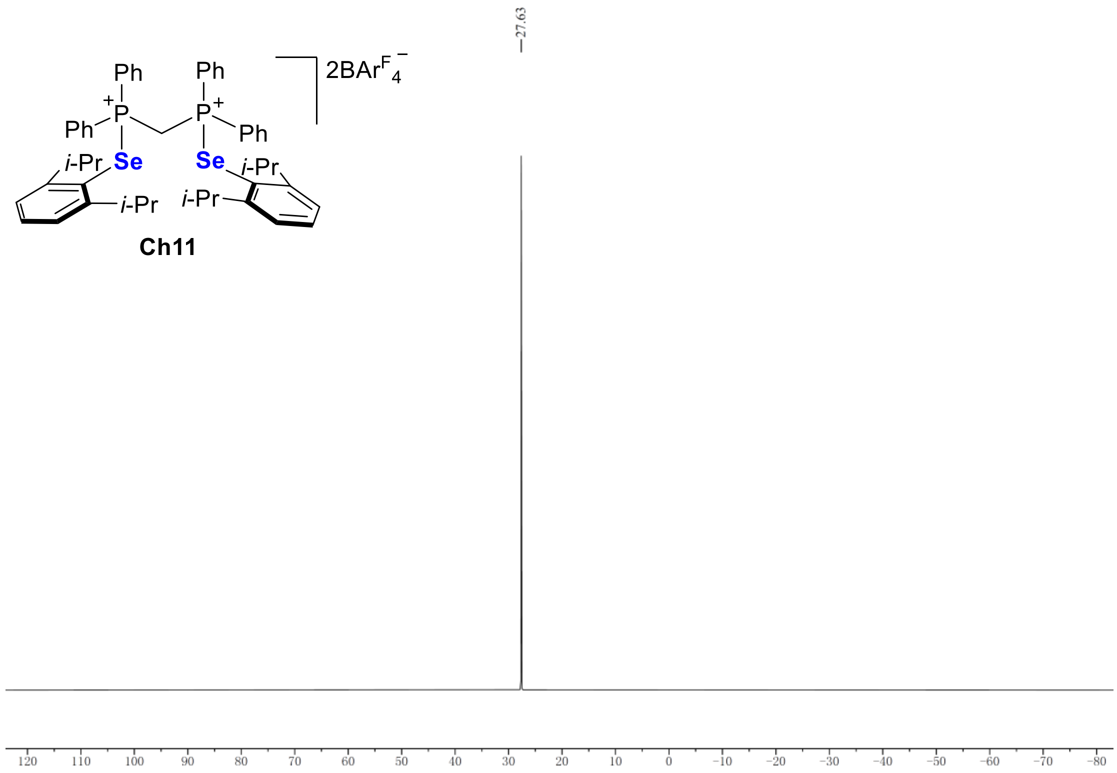

Supplementary Fig. 72 ^{31}P NMR spectrum of compound Ch11 (CD_2Cl_2, 162 MHz, 298K)

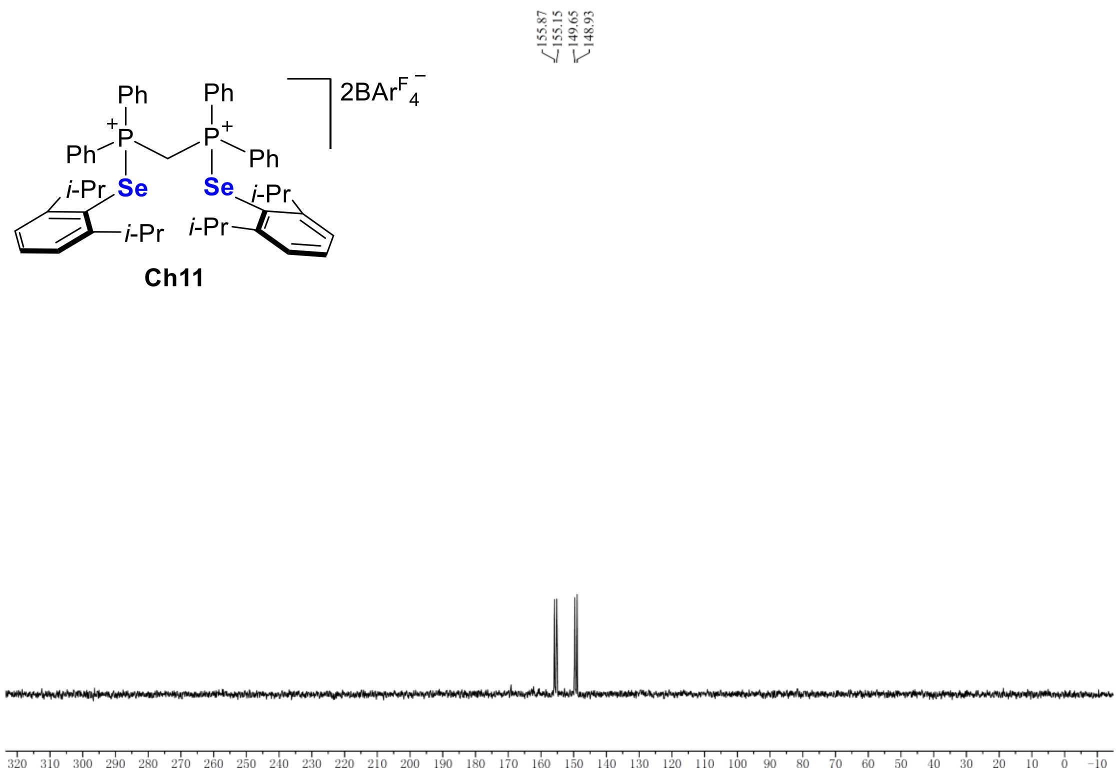

Supplementary Fig. 73 ^{77}**Se NMR spectrum of compound Ch11 (**CD_2Cl_2**, 76 MHz, 298K)**

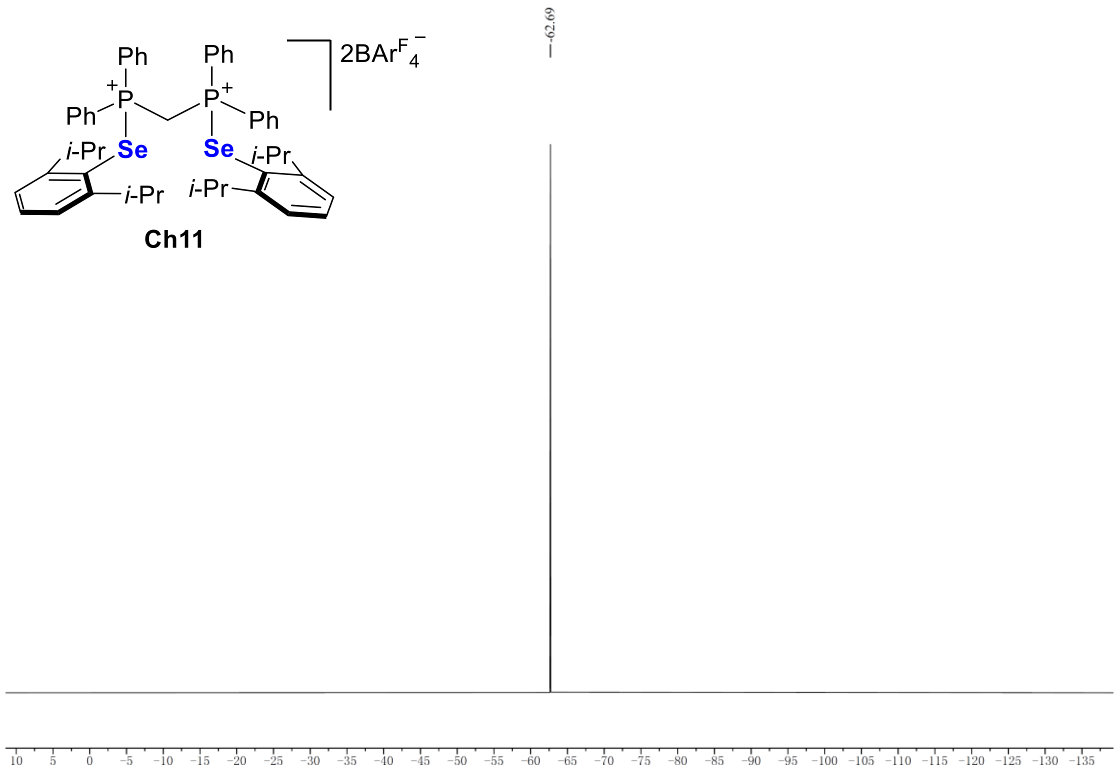

Supplementary Fig. 74 ^{19}**F NMR spectrum of compound Ch11 (**CD_2Cl_2**, 376 MHz, 298K)**

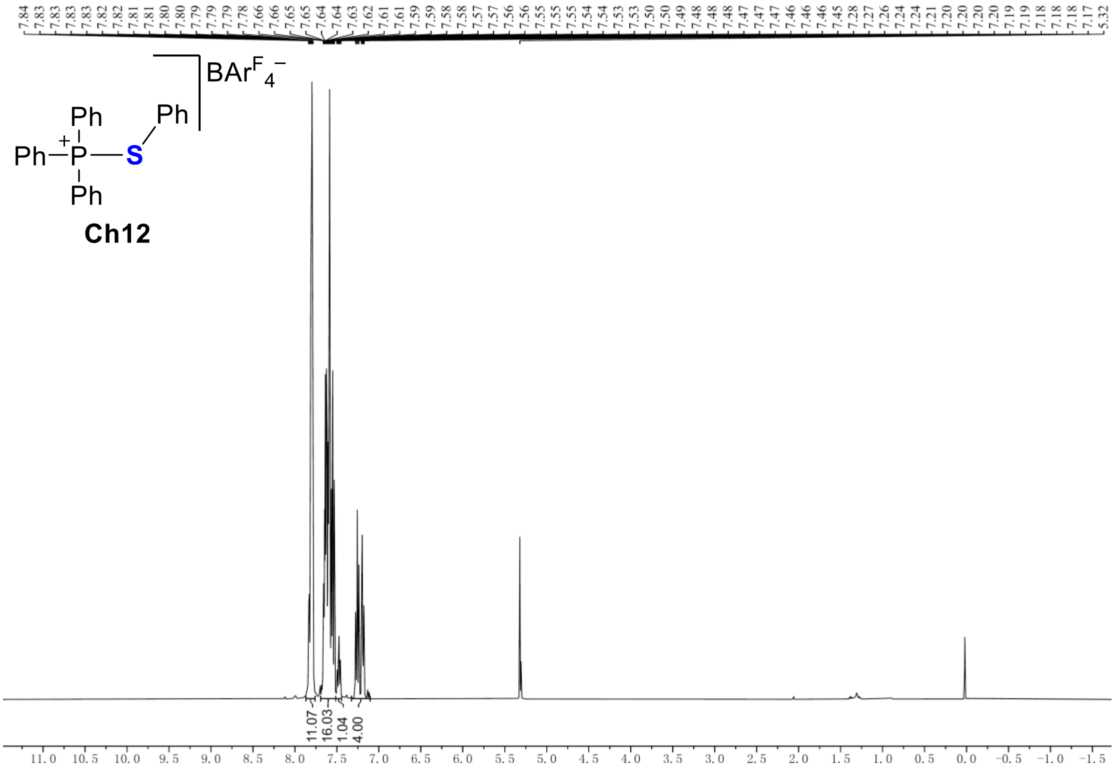

Supplementary Fig. 75 ^{1}H NMR spectrum of compound Ch12 (CD_2Cl_2, 400 MHz, 298K)

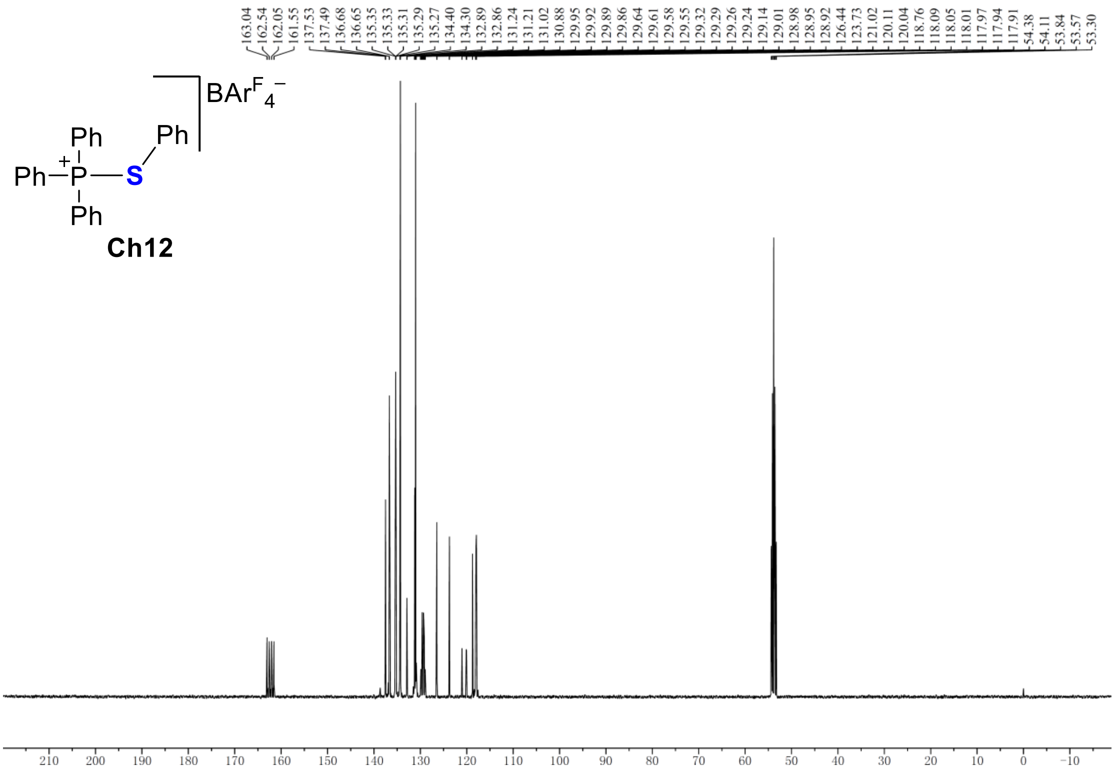

Supplementary Fig. 76 ^{13}C NMR spectrum of compound Ch12 (CD_2Cl_2, 100 MHz, 298K)

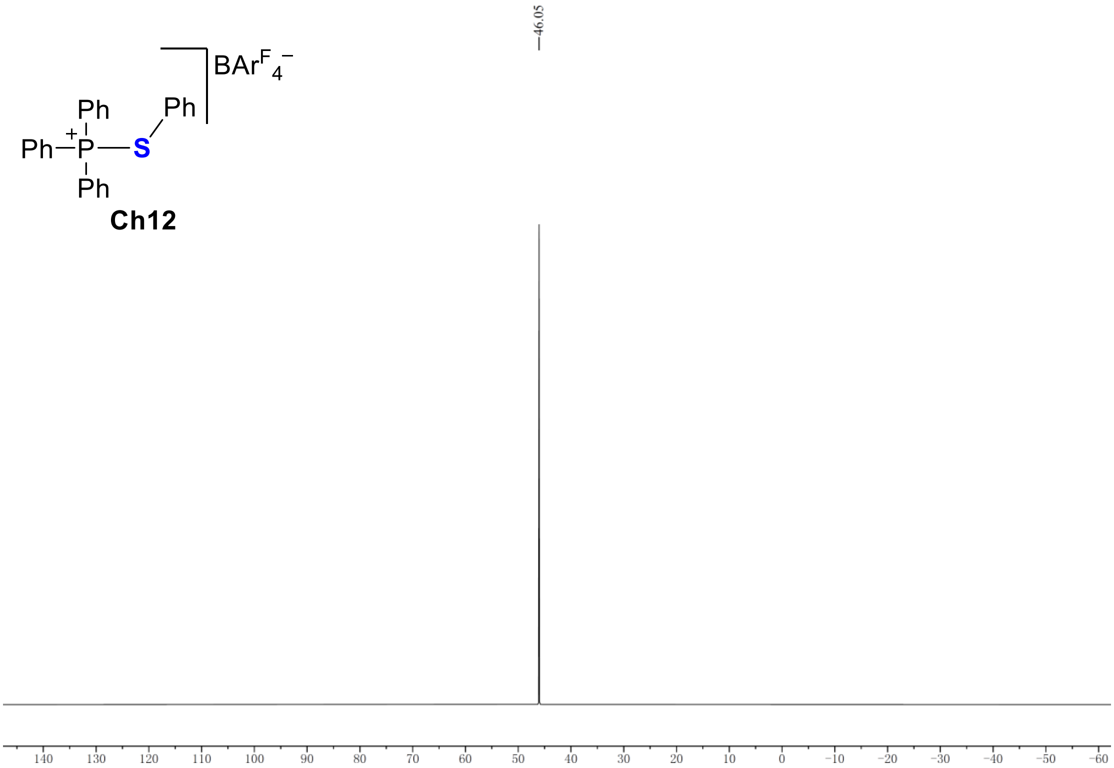

Supplementary Fig. 77 ^{31}P NMR spectrum of compound Ch12 (CD_2Cl_2, 162 MHz, 298K)

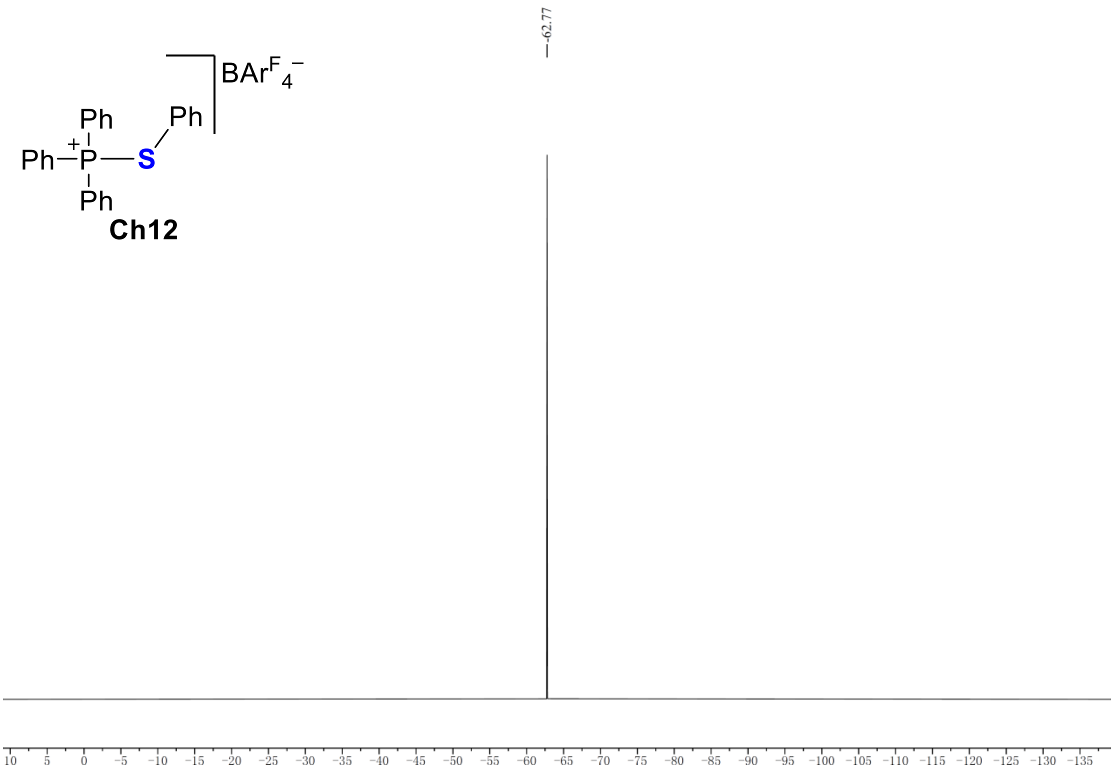

Supplementary Fig. 78 ^{19}F NMR spectrum of compound Ch12 (CD_2Cl_2, 376 MHz, 298K)

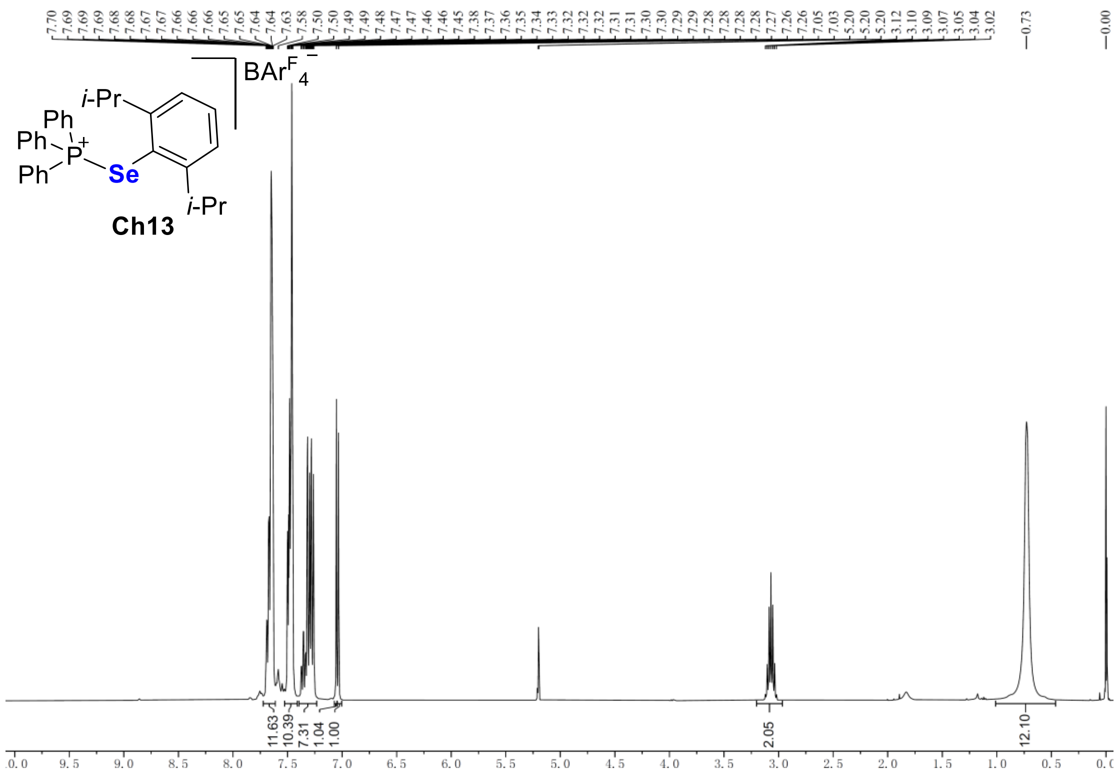

Supplementary Fig. 79 ^{1}**H NMR spectrum of compound Ch13 (**CD_2Cl_2**, 400 MHz, 298K)**

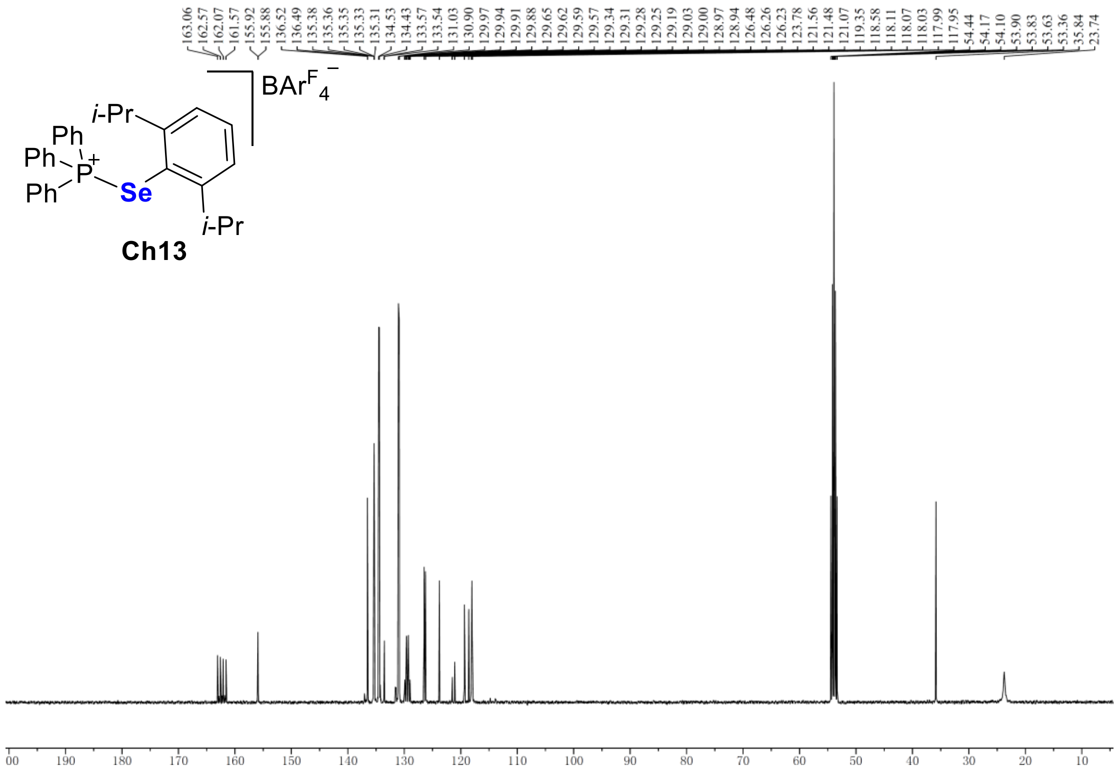

Supplementary Fig. 80 ^{13}**C NMR spectrum of compound Ch13 (**CD_2Cl_2**, 100 MHz, 298K)**

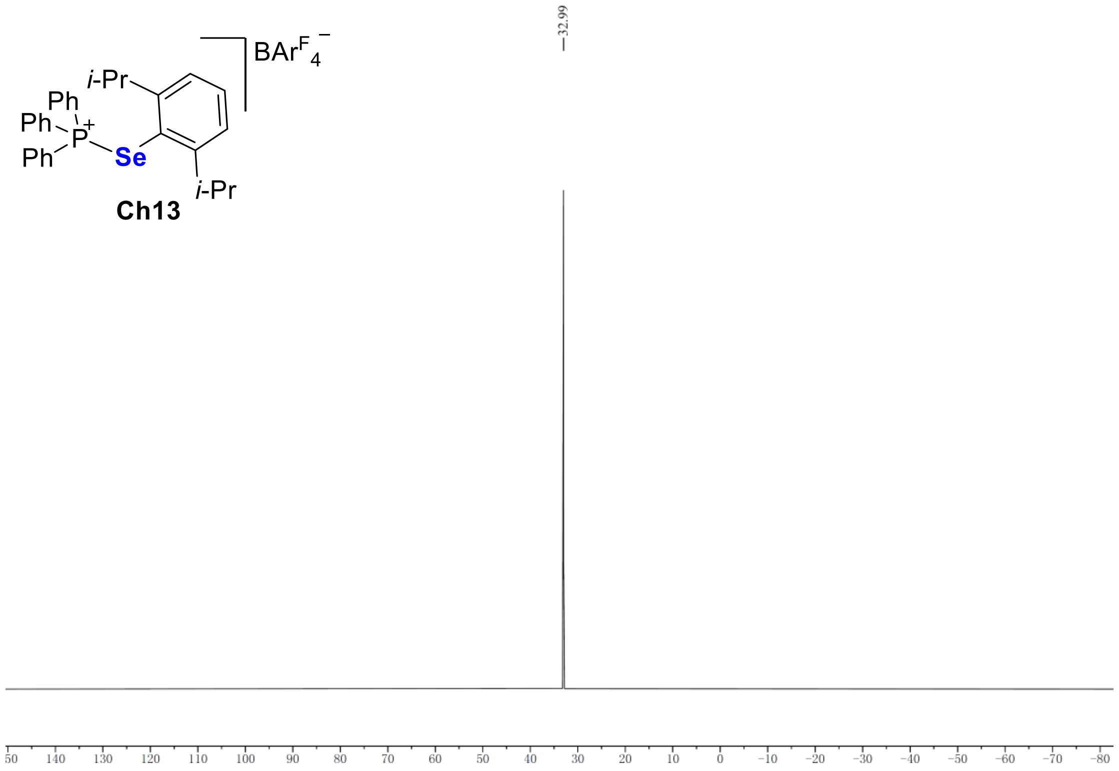

Supplementary Fig. 81 ^{31}P NMR spectrum of compound Ch13 (CD_2Cl_2, 162 MHz, 298K)

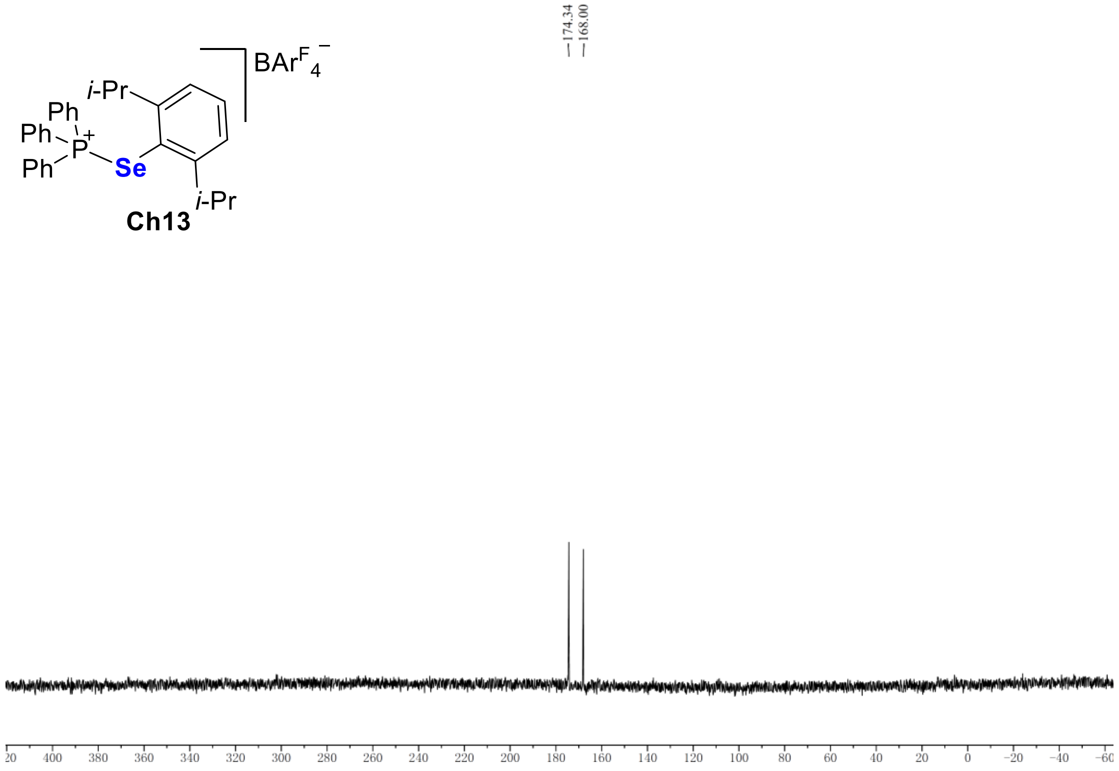

Supplementary Fig. 82 ^{77}Se NMR spectrum of compound Ch13 (CD_2Cl_2, 76 MHz, 298K)

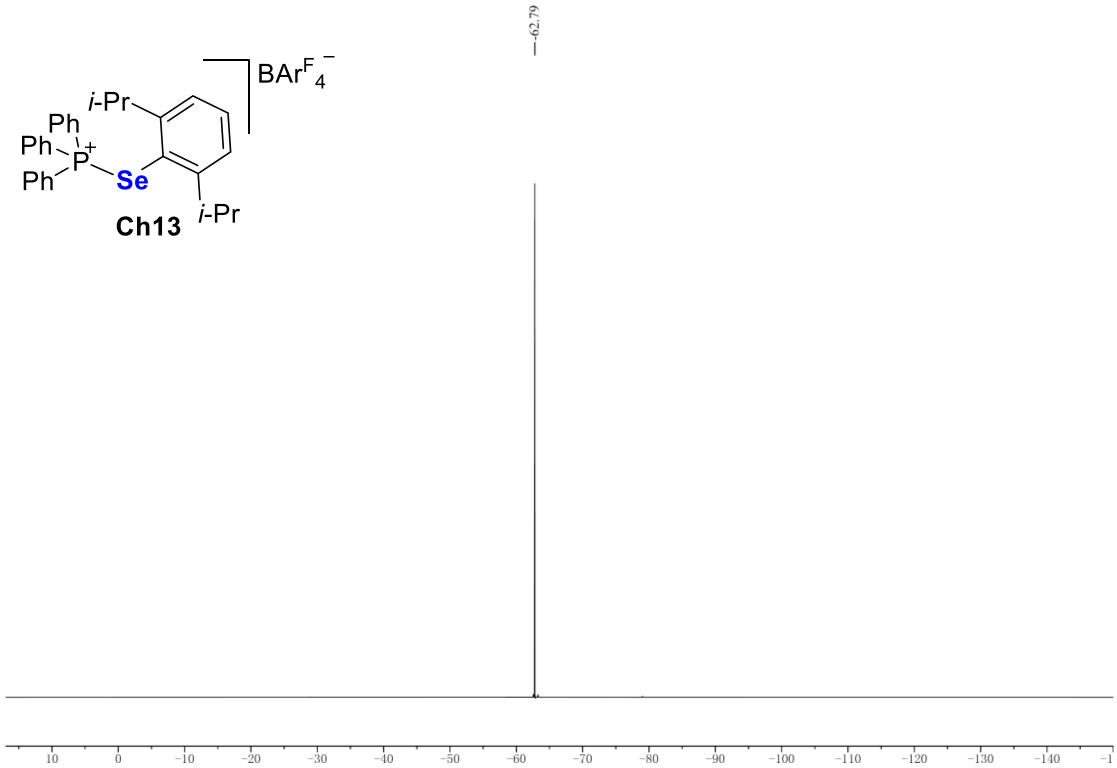

Supplementary Fig. 83 ^{19}F NMR spectrum of compound Ch13 (CD_2Cl_2, 376 MHz, 298K)

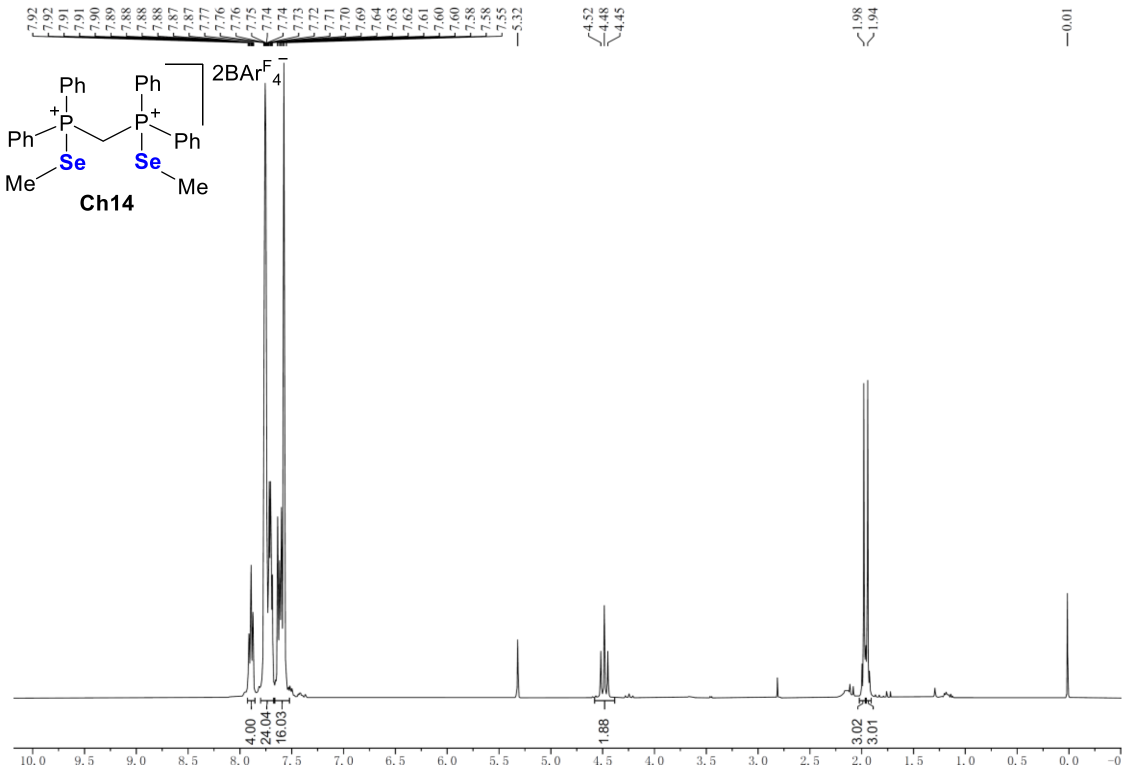

Supplementary Fig. 84 ^{1}H NMR spectrum of compound Ch14 (CD_2Cl_2, 400 MHz, 298K)

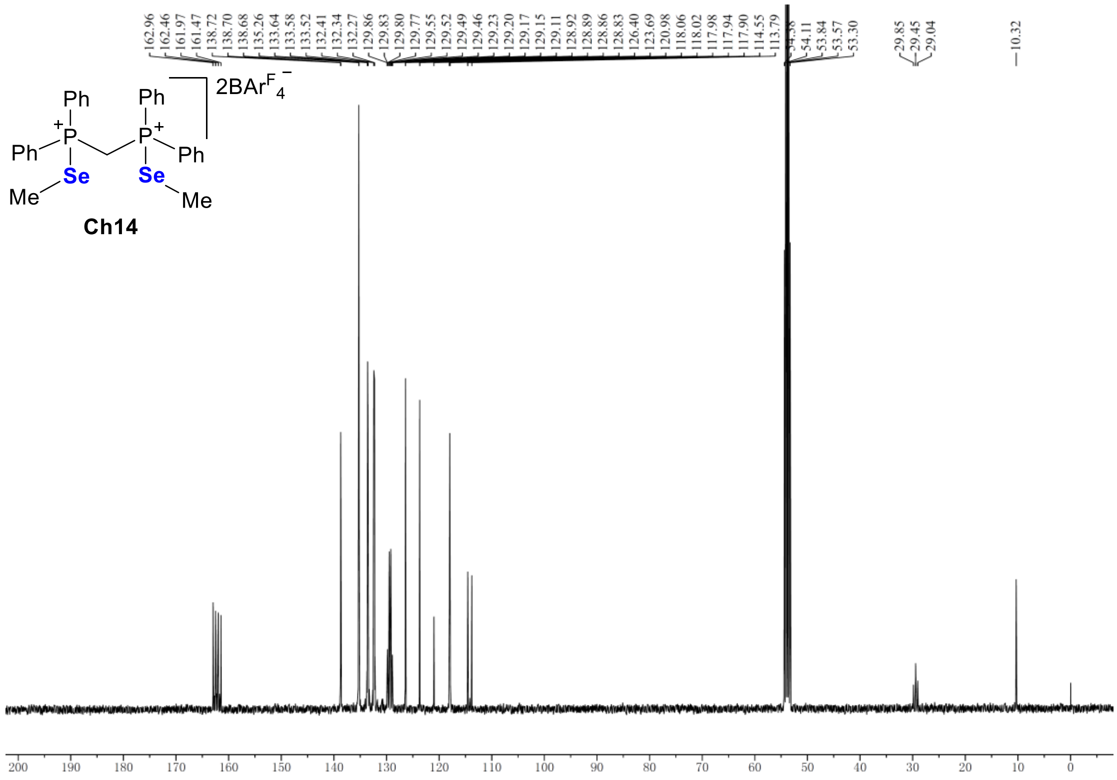

Supplementary Fig. 85 ^{13}C NMR spectrum of compound Ch14 (CD_2Cl_2, 100 MHz, 298K)

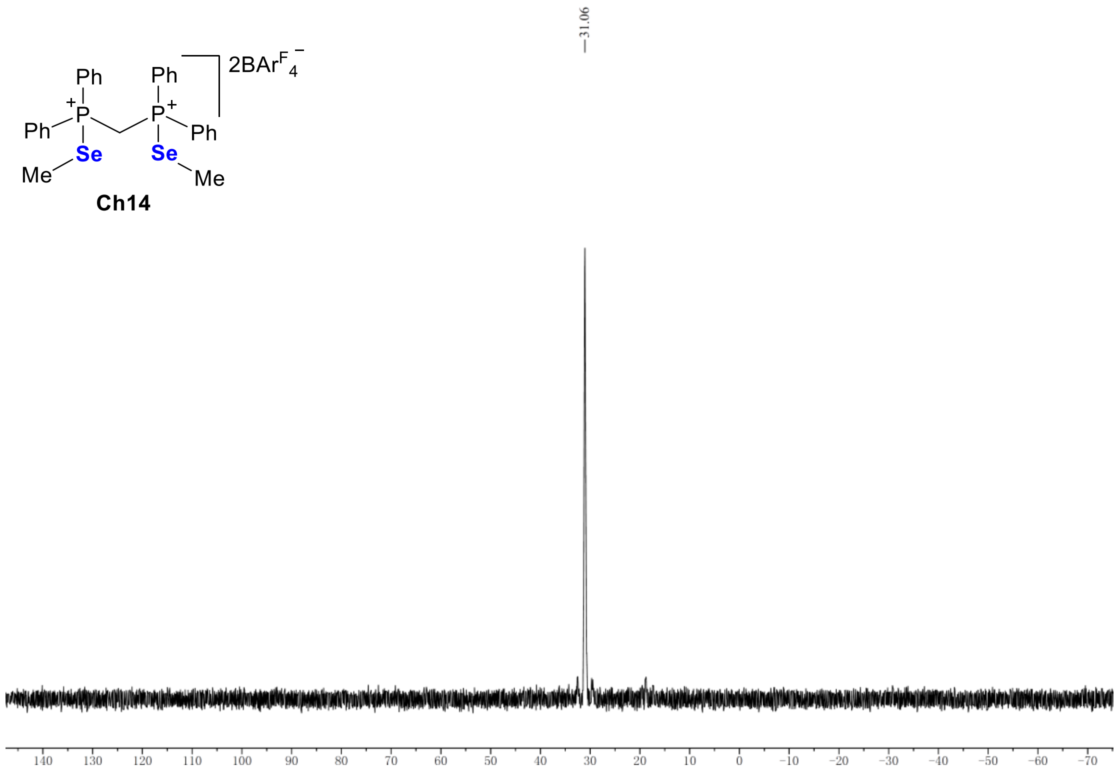

Supplementary Fig. 86 ^{31}P NMR spectrum of compound Ch14 (CD_2Cl_2, 162 MHz, 298K)

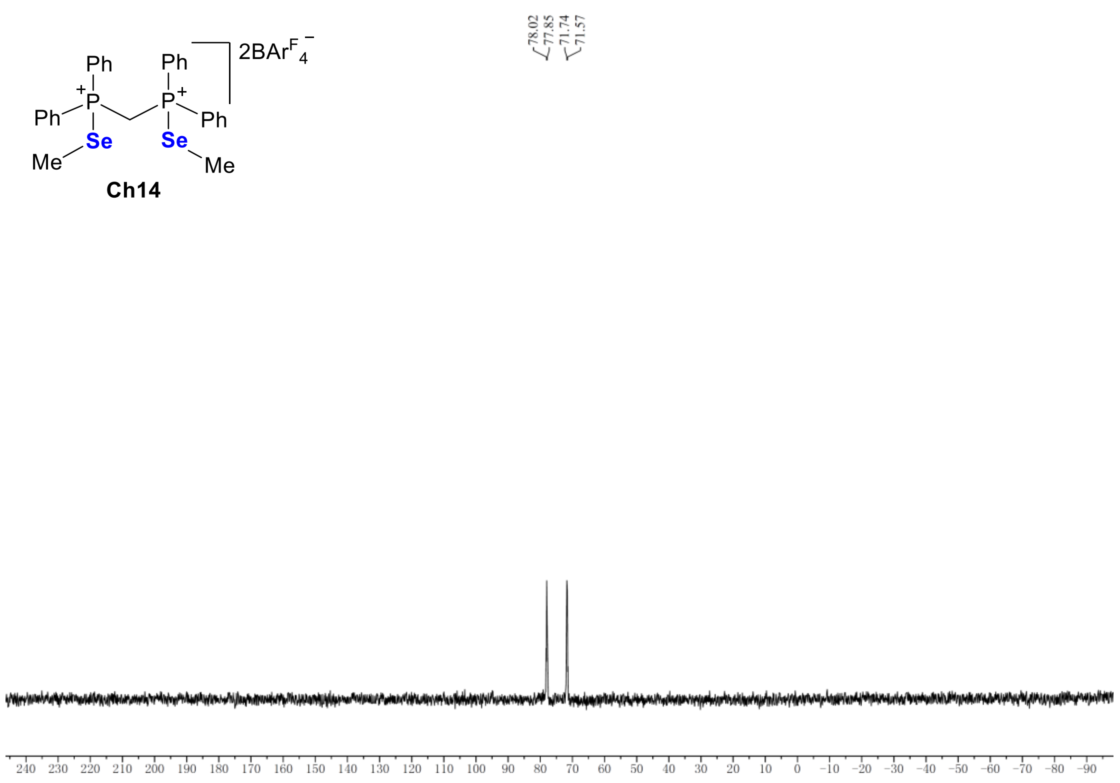

Supplementary Fig. 87 ^{77}Se NMR spectrum of compound Ch14 (CD_2Cl_2, 76 MHz, 298K)

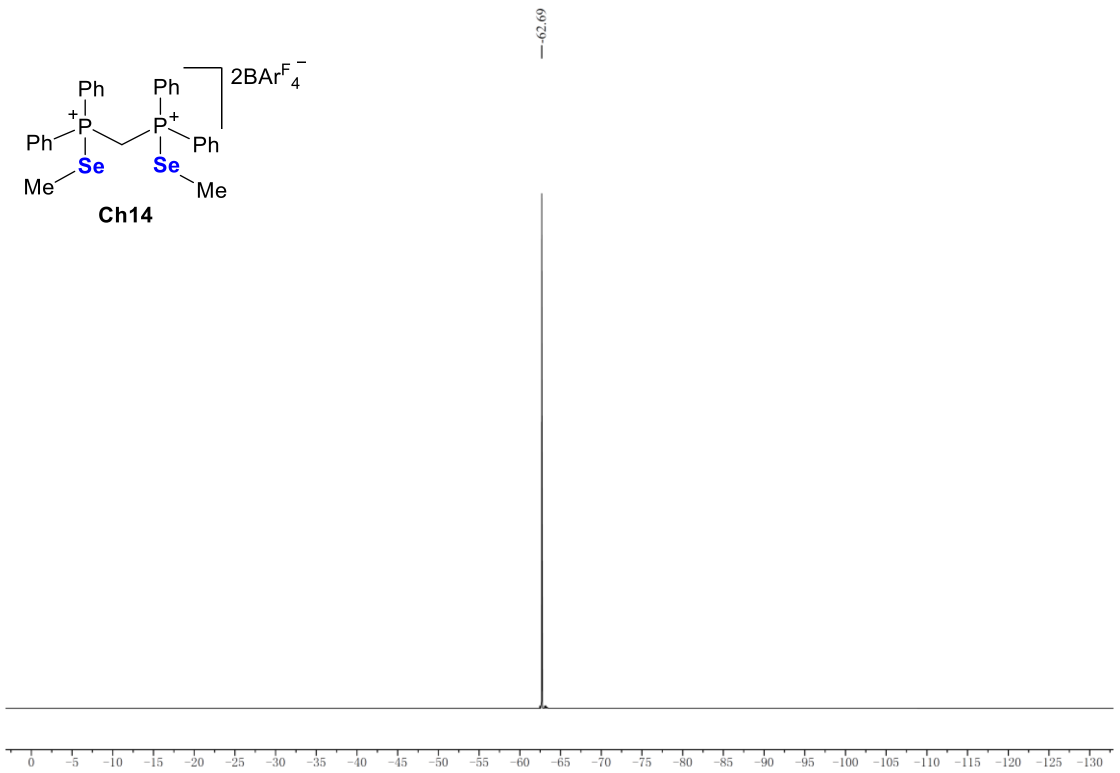

Supplementary Fig. 88 ^{19}F NMR spectrum of compound Ch14 (CD_2Cl_2, 376 MHz, 298K)

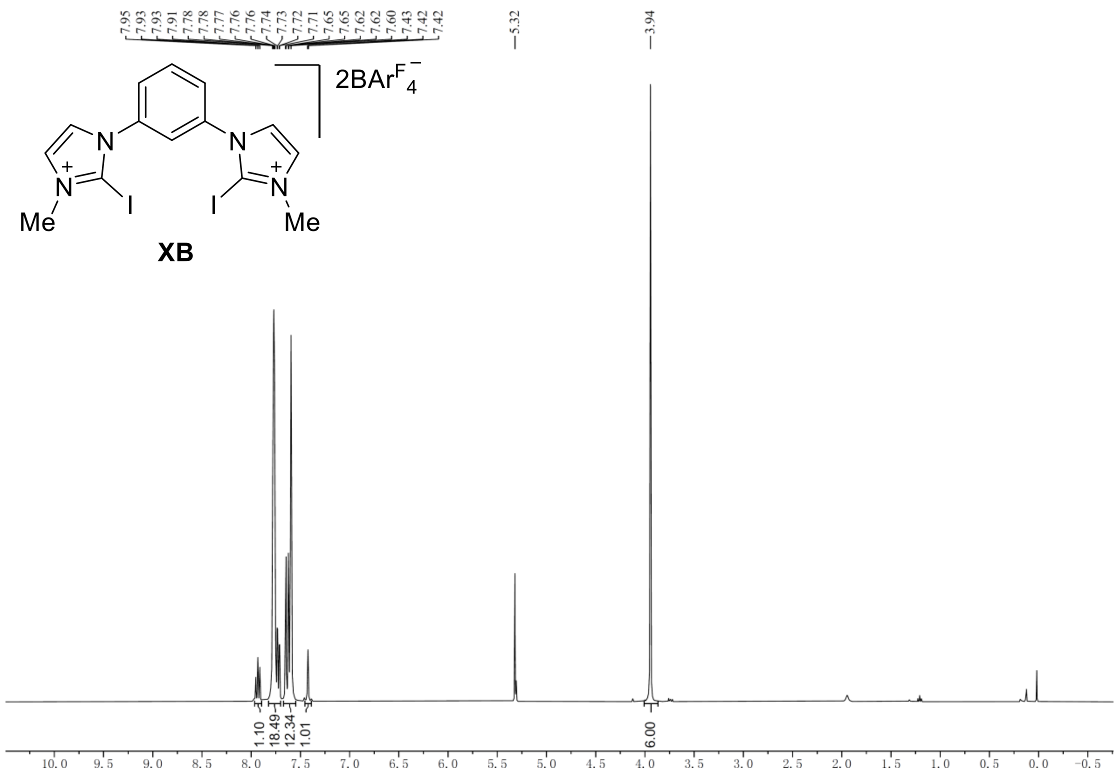

Supplementary Fig. 89 ^{1}H NMR spectrum of compound XB (CD_2Cl_2, 400 MHz, 298K)

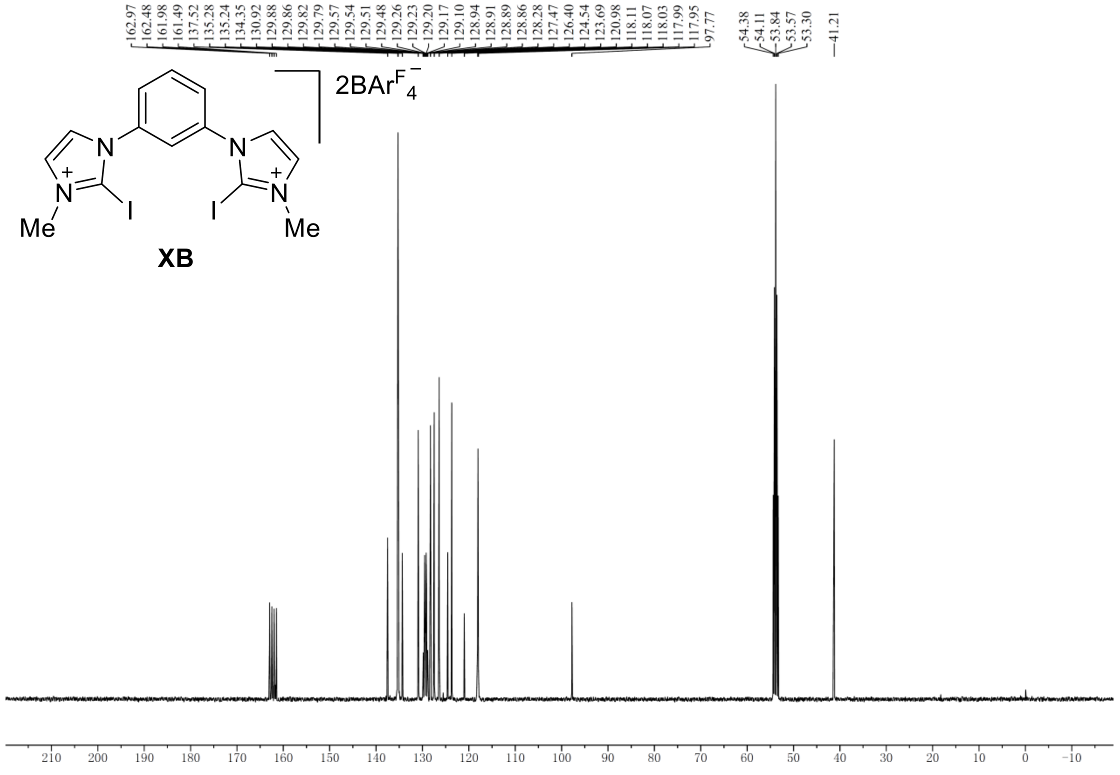

Supplementary Fig. 90 ^{13}C NMR spectrum of compound XB (CD_2Cl_2, 100 MHz, 298K)

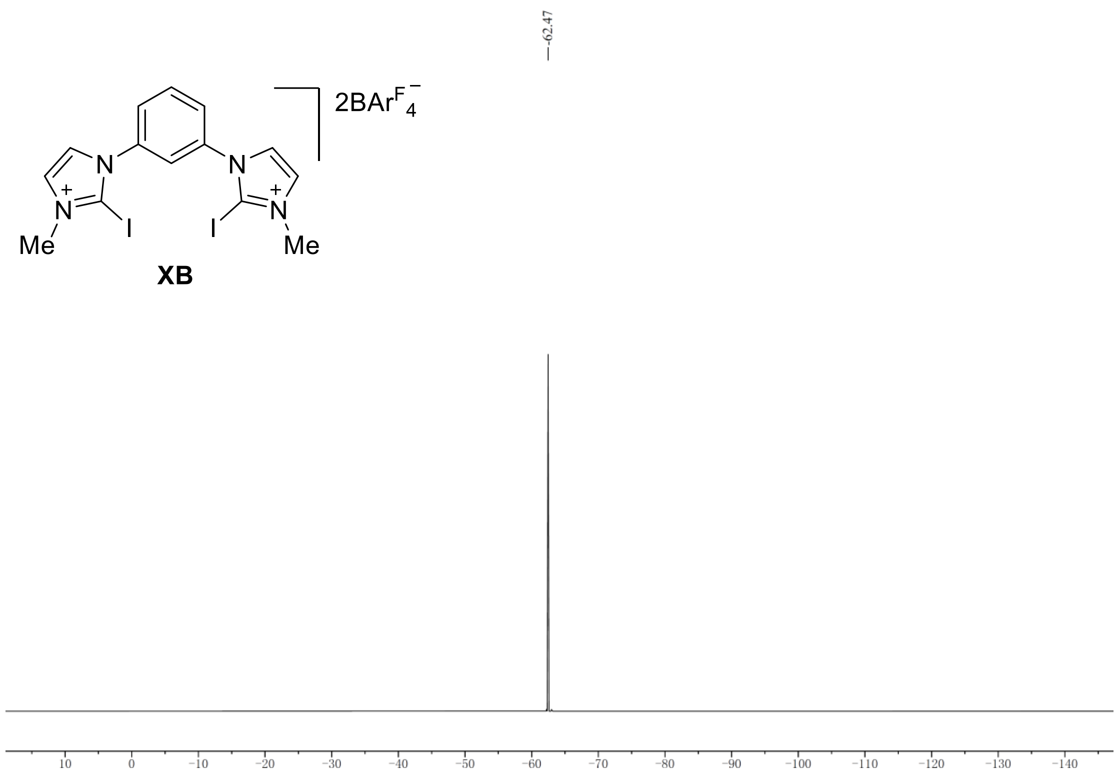

Supplementary Fig. 91 ^{19}F NMR spectrum of compound XB (CD_2Cl_2, 376 MHz, 298K)

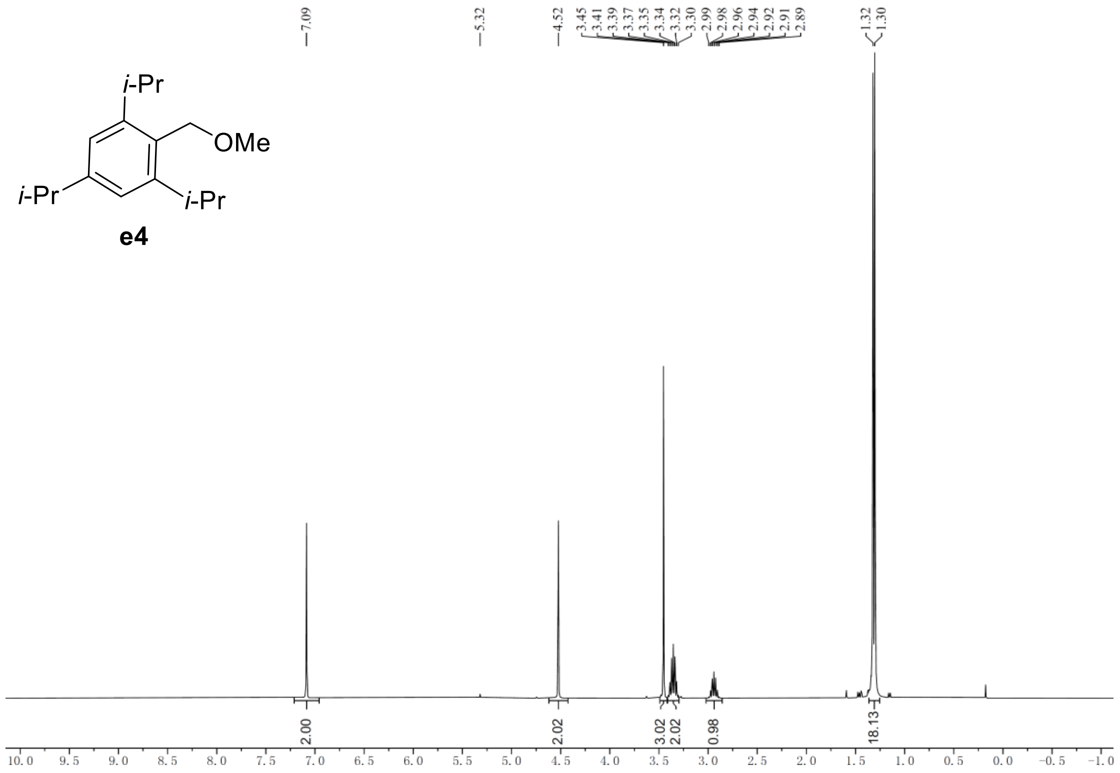

Supplementary Fig. 92 ^{1}H NMR spectrum of compound e4 (CD_2Cl_2, 400 MHz, 298K)

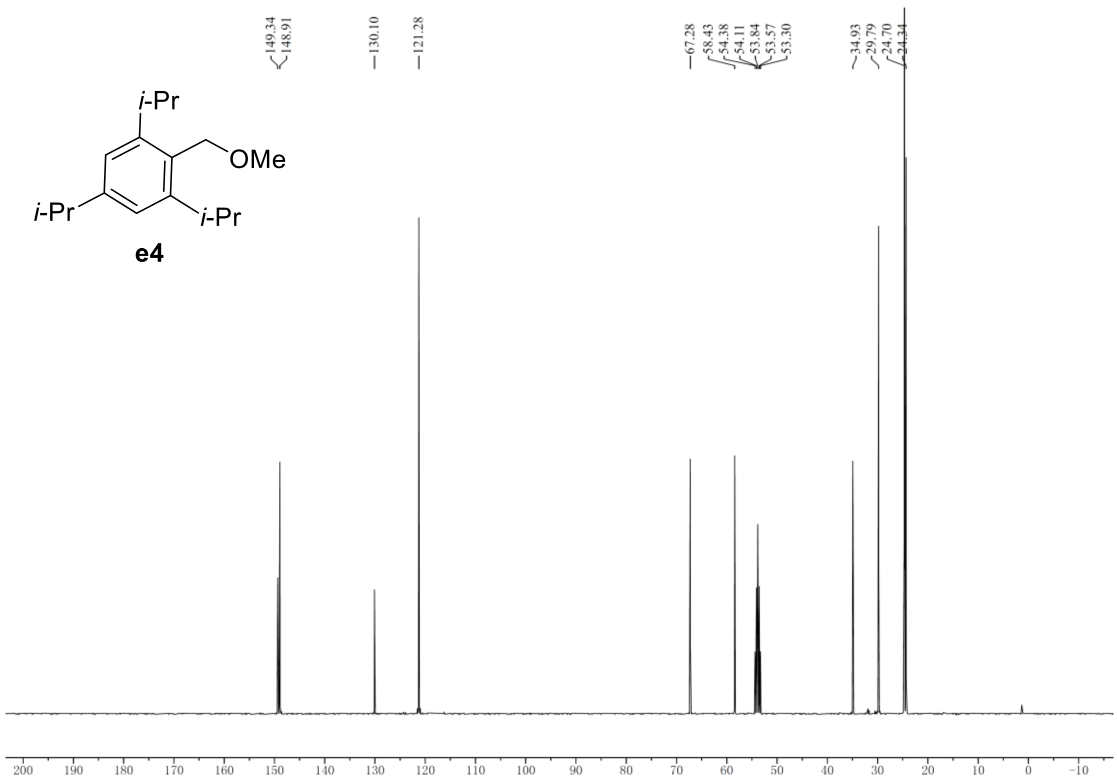

Supplementary Fig. 93 ^{13}C NMR spectrum of compound e4 (CD_2Cl_2, 100 MHz, 298K)

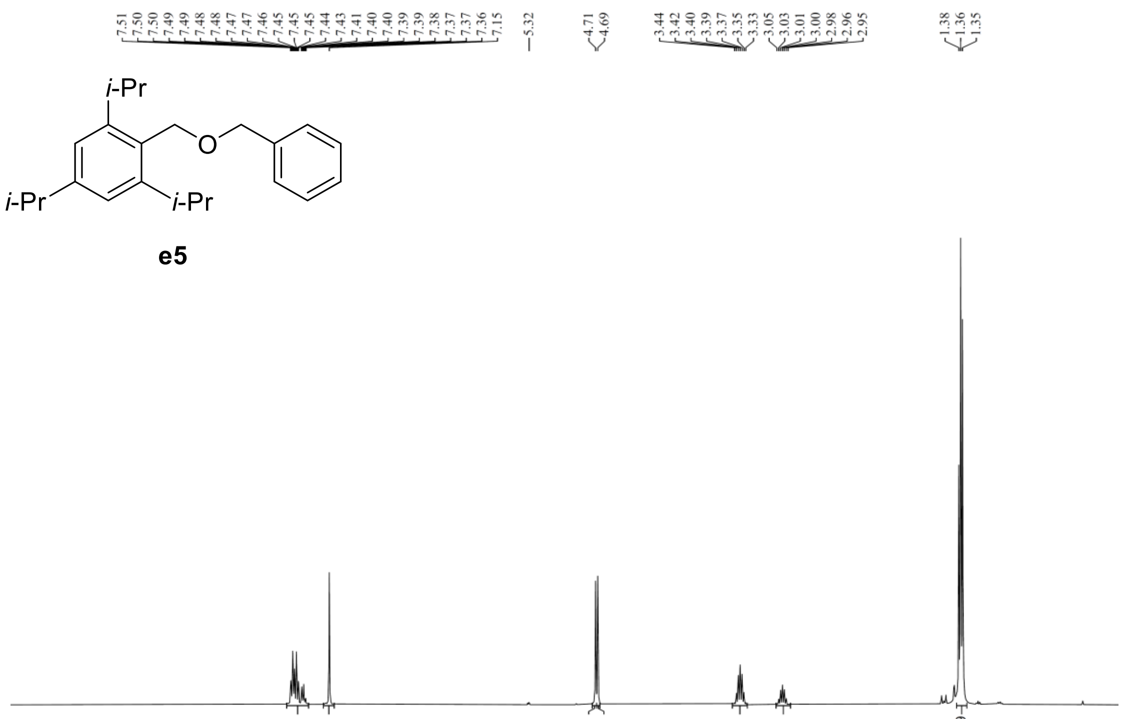

Supplementary Fig. 94 ^{1}H NMR spectrum of compound e5 (CD_2Cl_2, 400 MHz, 298K)

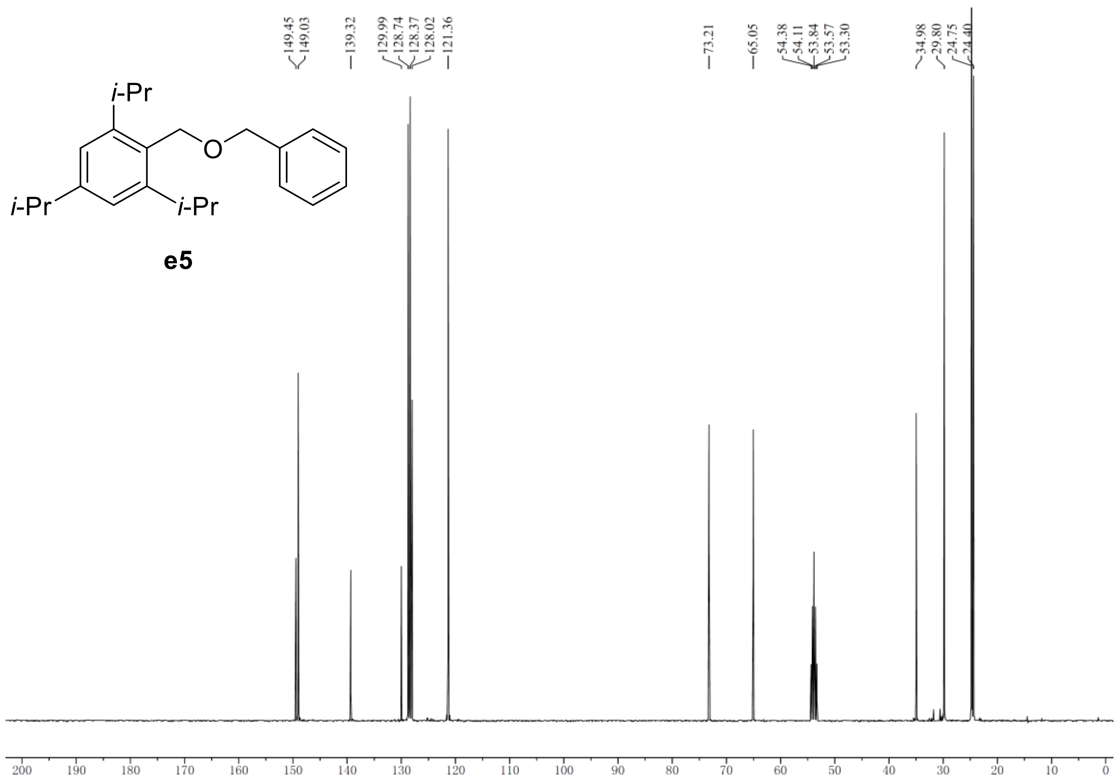

Supplementary Fig. 95 ^{13}C NMR spectrum of compound e5 (CD_2Cl_2, 100 MHz, 298K)

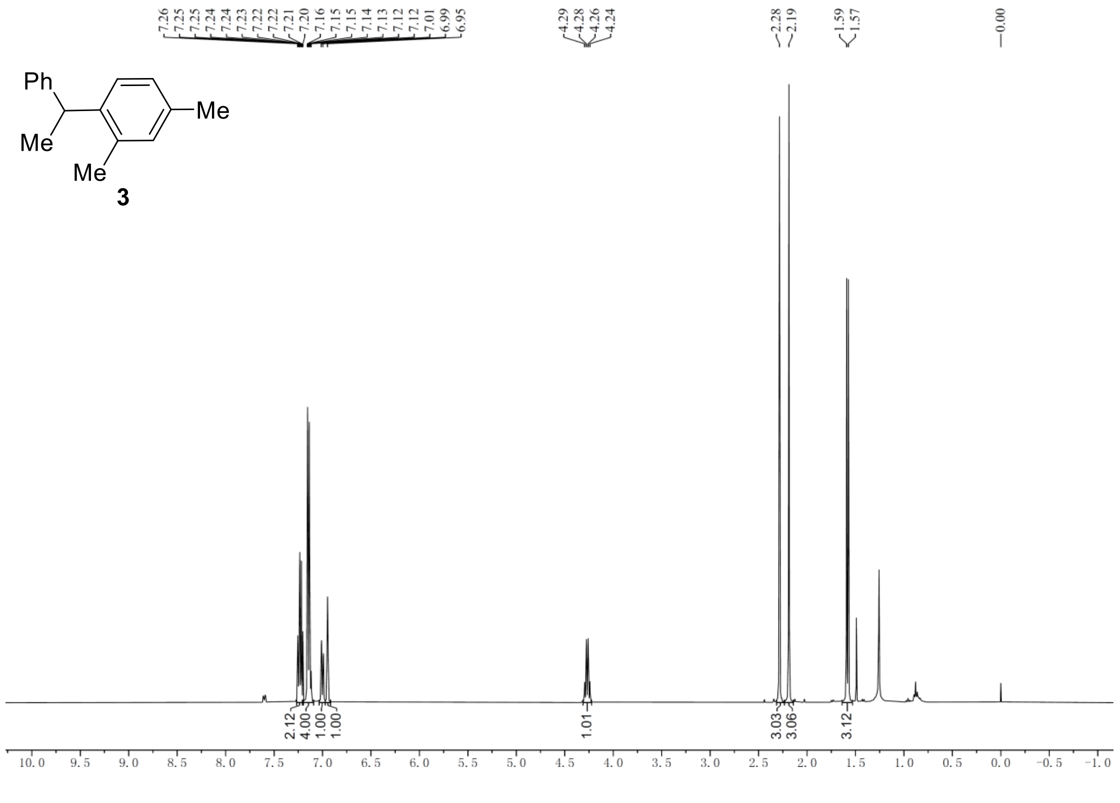

Supplementary Fig. 96 ^{1}H NMR spectrum of compound 3 ($CDCl_3$, 400 MHz, 298K)

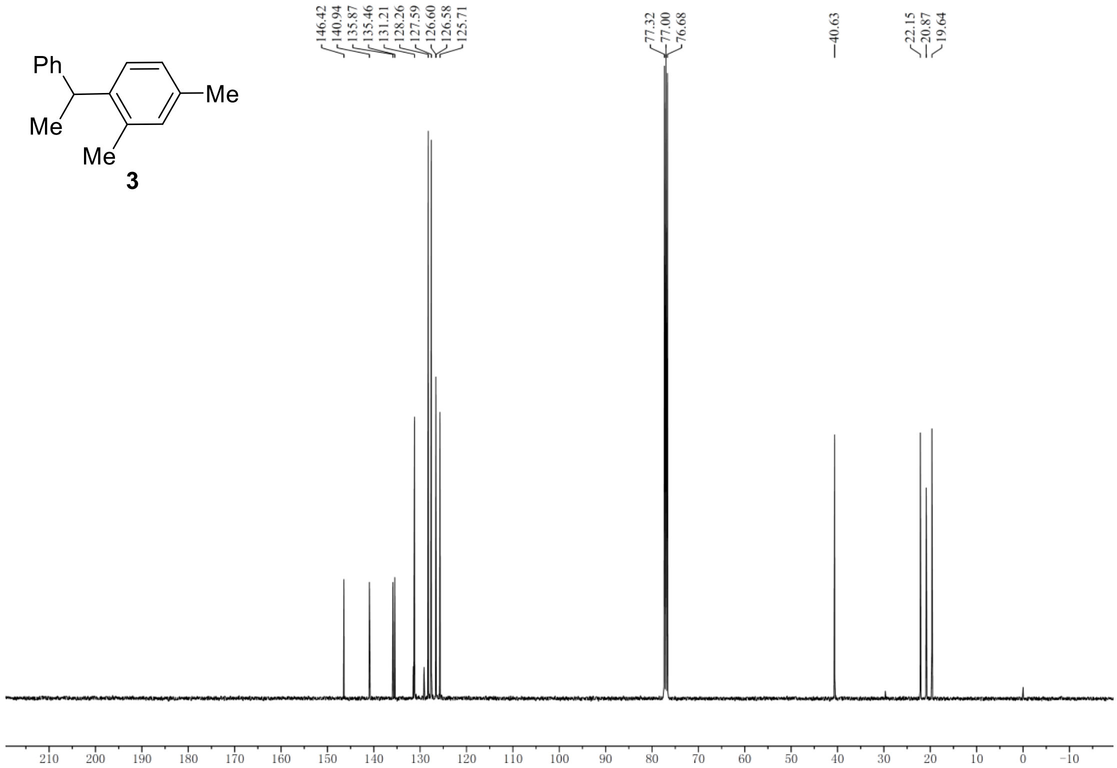

Supplementary Fig. 97 ^{13}C NMR spectrum of compound 3 ($CDCl_3$, 100 MHz, 298K)

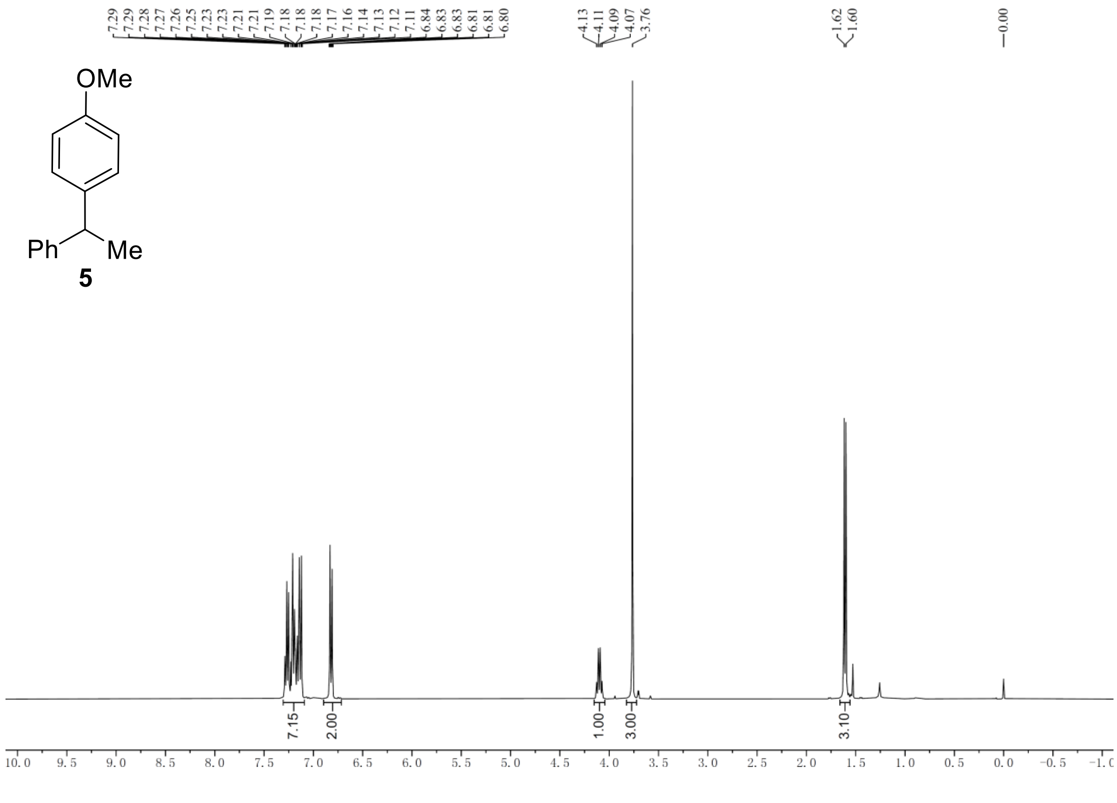

Supplementary Fig. 98 ^{1}H NMR spectrum of compound 5 ($CDCl_3$, 400 MHz, 298K)

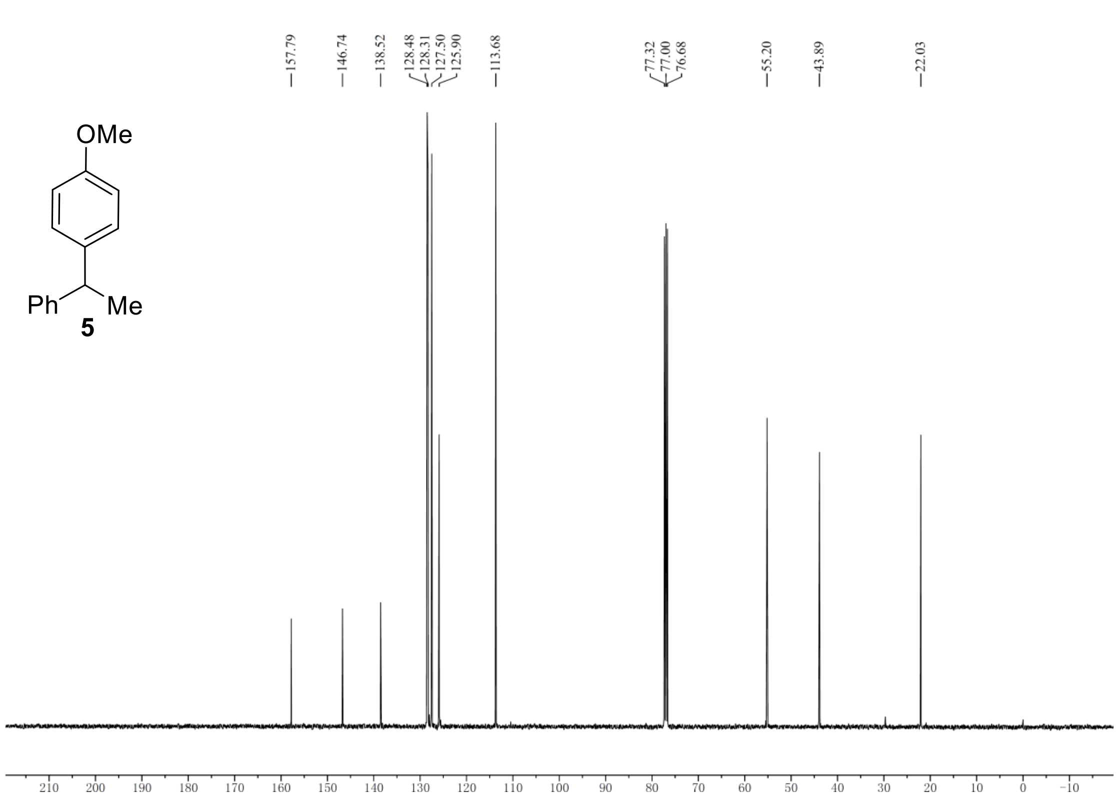

Supplementary Fig. 99 ^{13}C NMR spectrum of compound 5 ($CDCl_3$, 100 MHz, 298K)

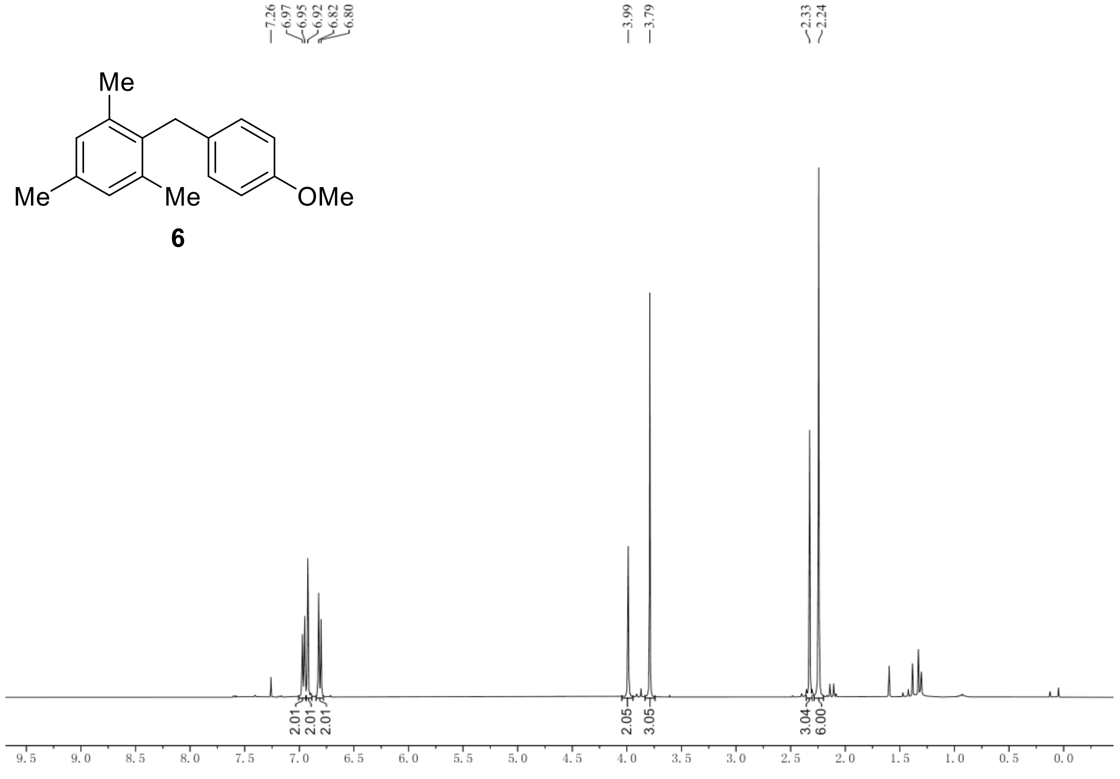

Supplementary Fig. 100 ^{1}H NMR spectrum of compound 6 ($CDCl_3$, 400 MHz, 298K)

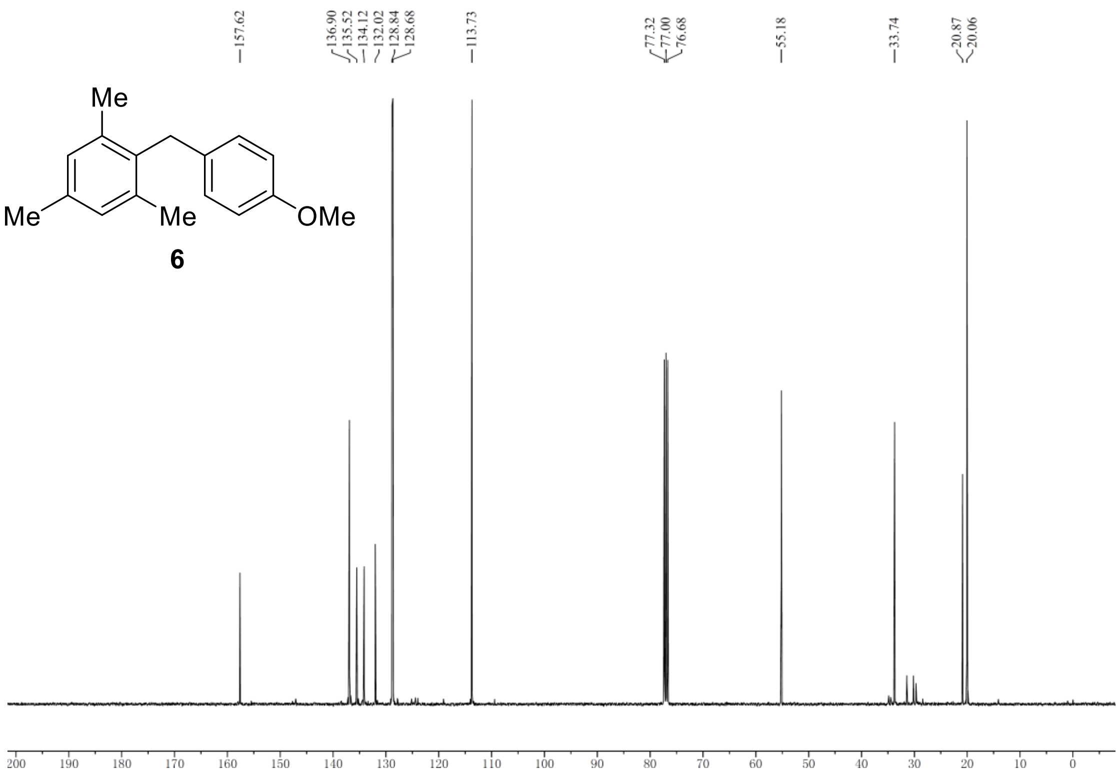

Supplementary Fig. 101 ^{13}C NMR spectrum of compound 6 ($CDCl_3$, 100 MHz, 298K)

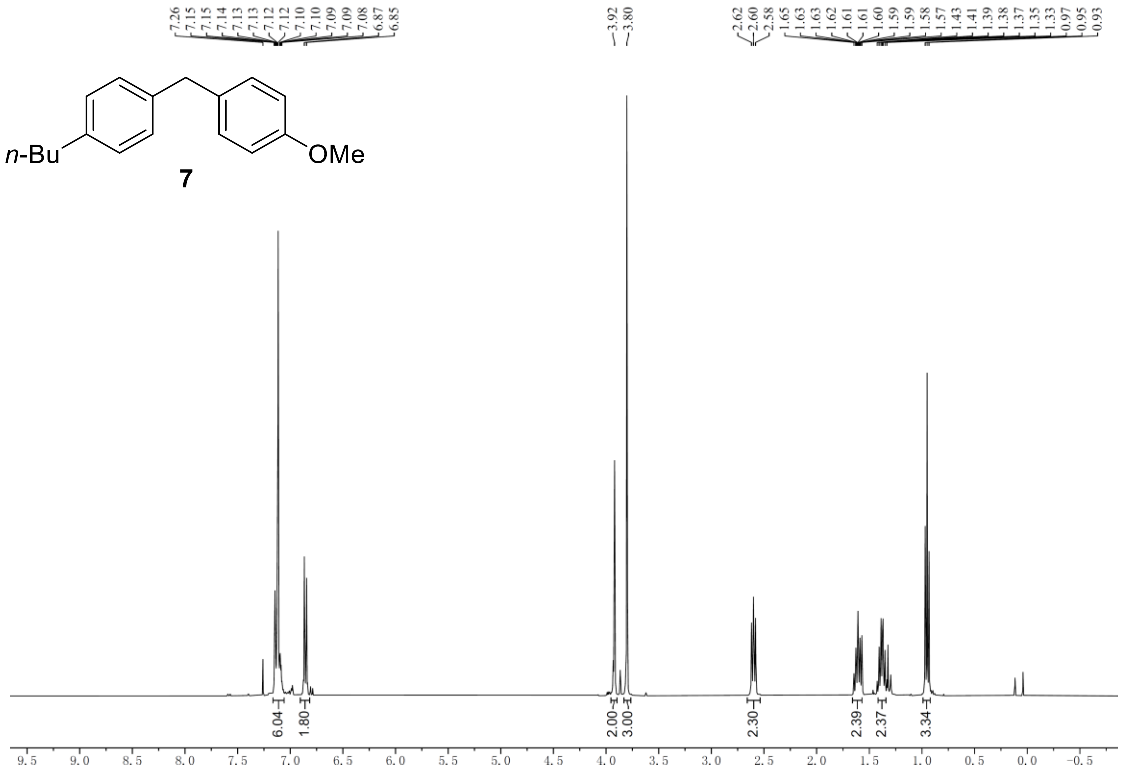

Supplementary Fig. 102 ^{1}H NMR spectrum of compound 7 ($CDCl_3$, 400 MHz, 298K)

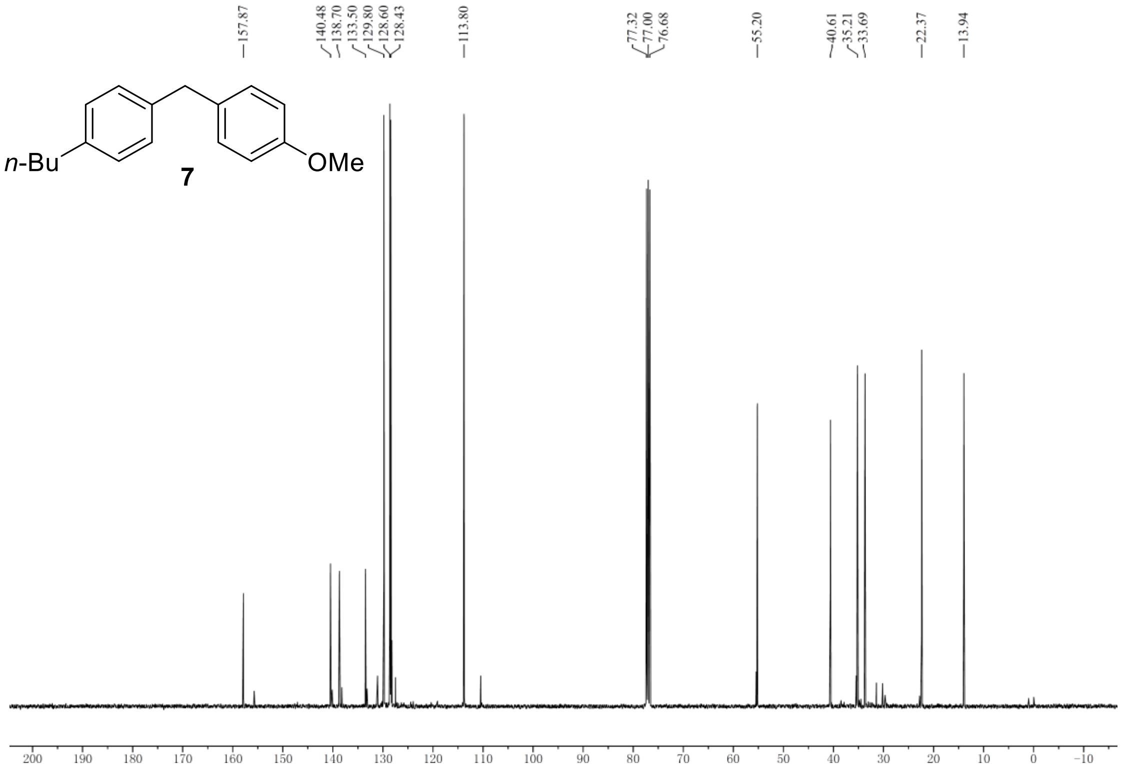

Supplementary Fig. 103 ^{13}C NMR spectrum of compound 7 ($CDCl_3$, 100 MHz, 298K)

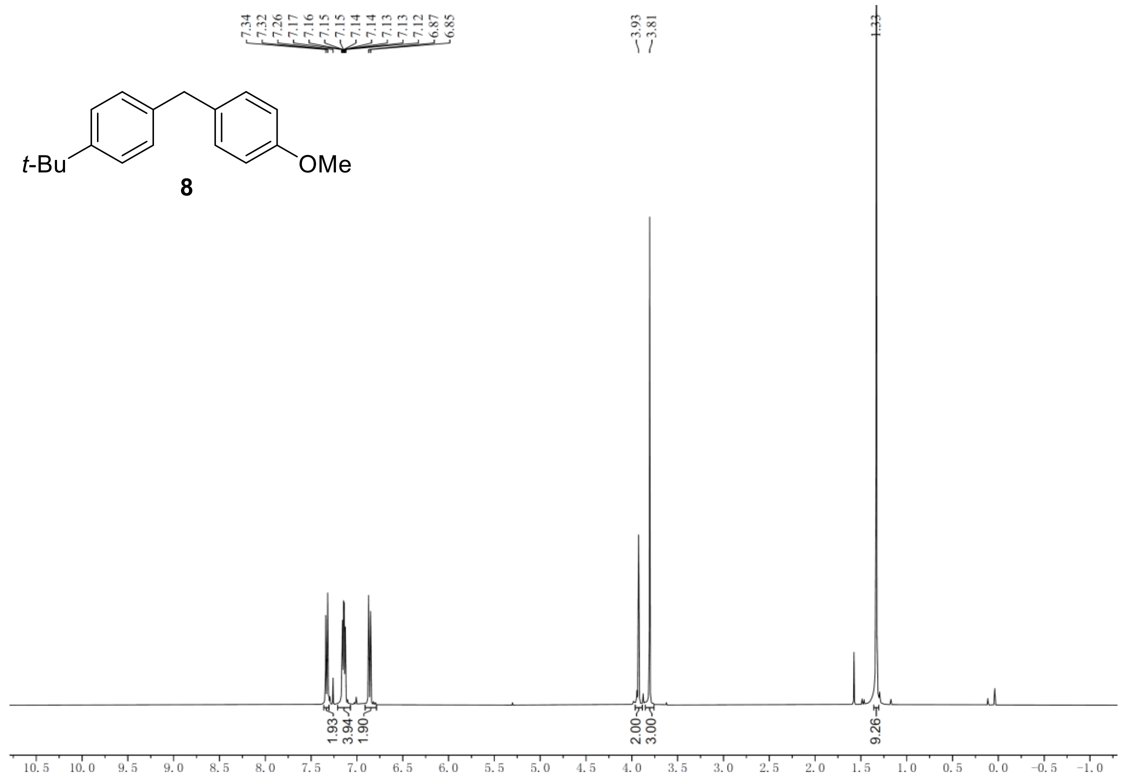

Supplementary Fig. 104 ^{1}H NMR spectrum of compound 8 ($CDCl_3$, 400 MHz, 298K)

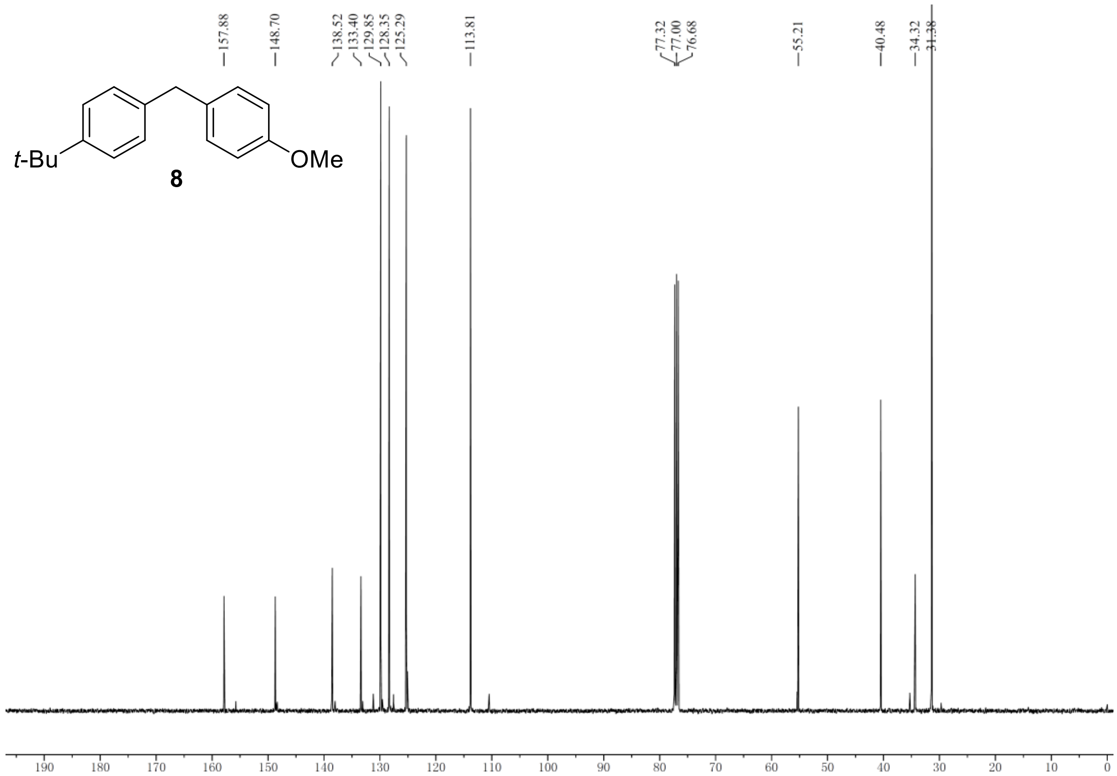

Supplementary Fig. 105 ^{13}C NMR spectrum of compound 8 ($CDCl_3$, 100 MHz, 298K)

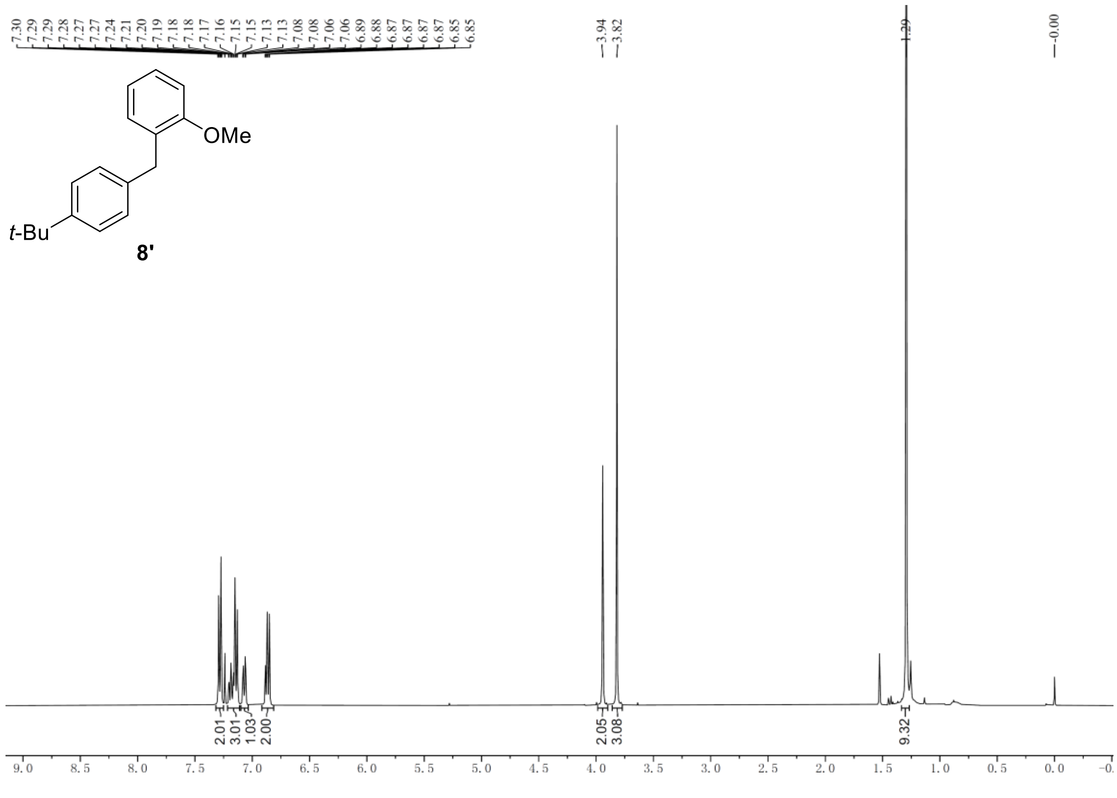

Supplementary Fig. 106 ^{1}H NMR spectrum of compound 8' ($CDCl_3$, 400 MHz, 298K)

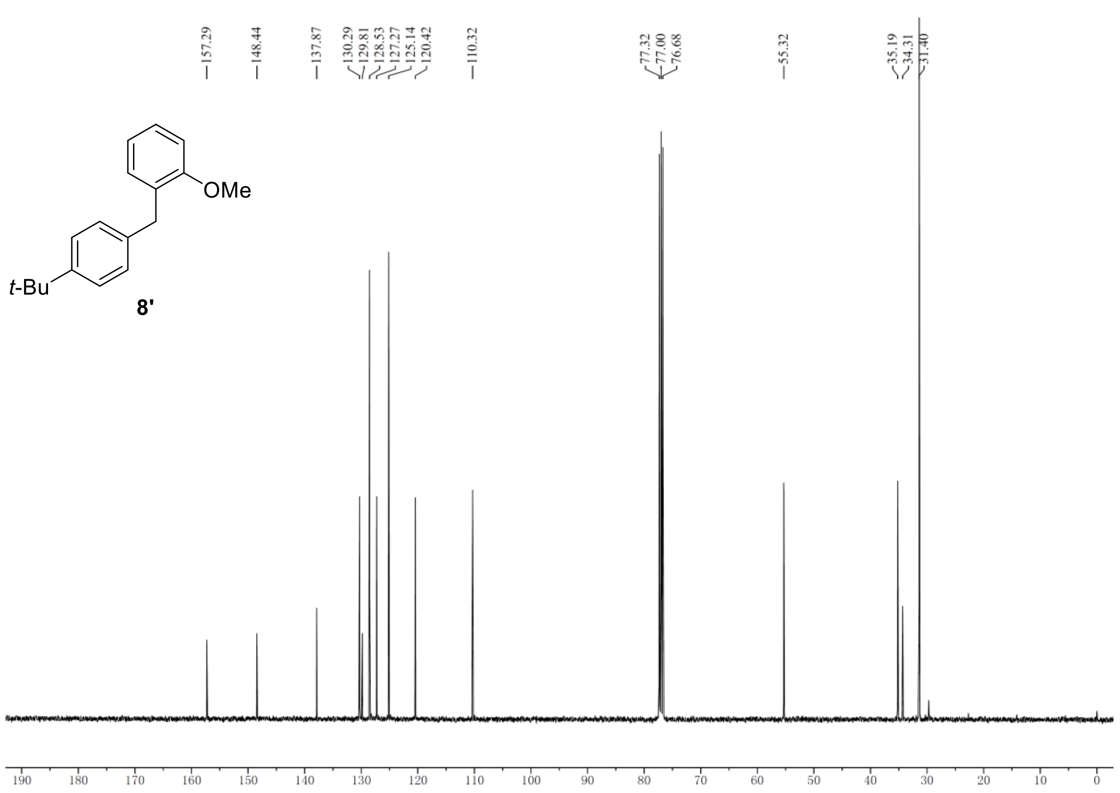

Supplementary Fig. 107 ^{13}C NMR spectrum of compound 8' (CDCl$_3$, 100 MHz, 298K)

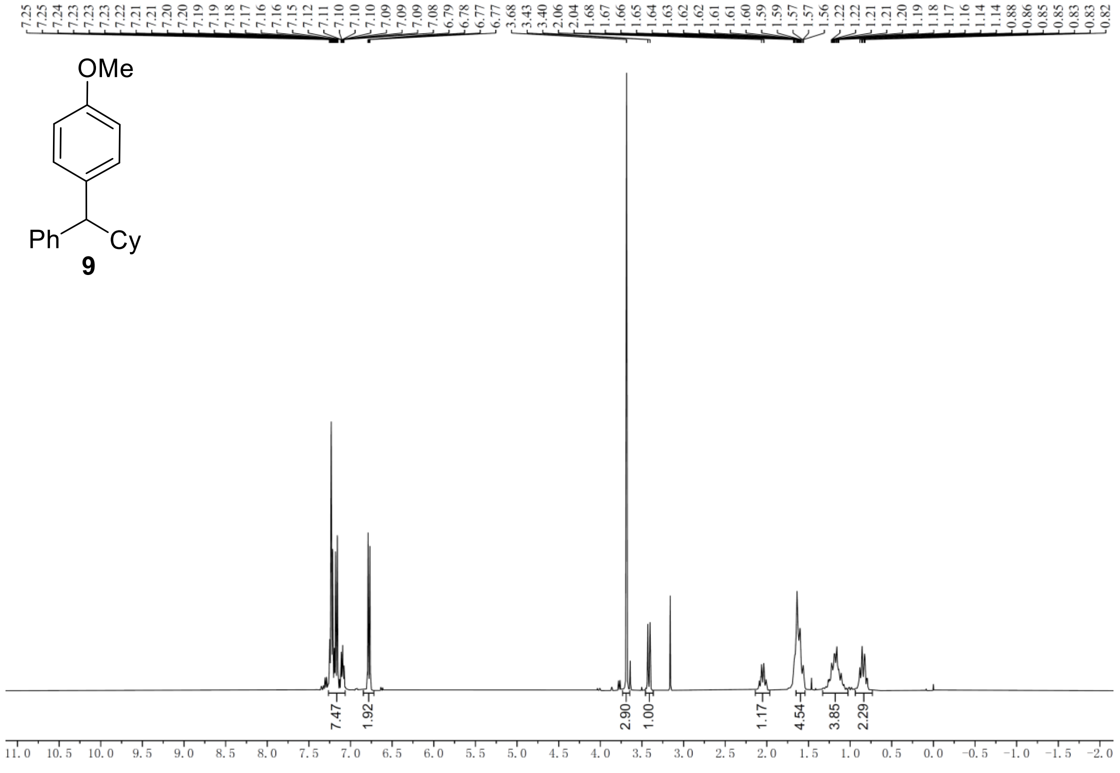

Supplementary Fig. 108 ^{1}H NMR spectrum of compound 9 (CDCl$_3$, 400 MHz, 298K)

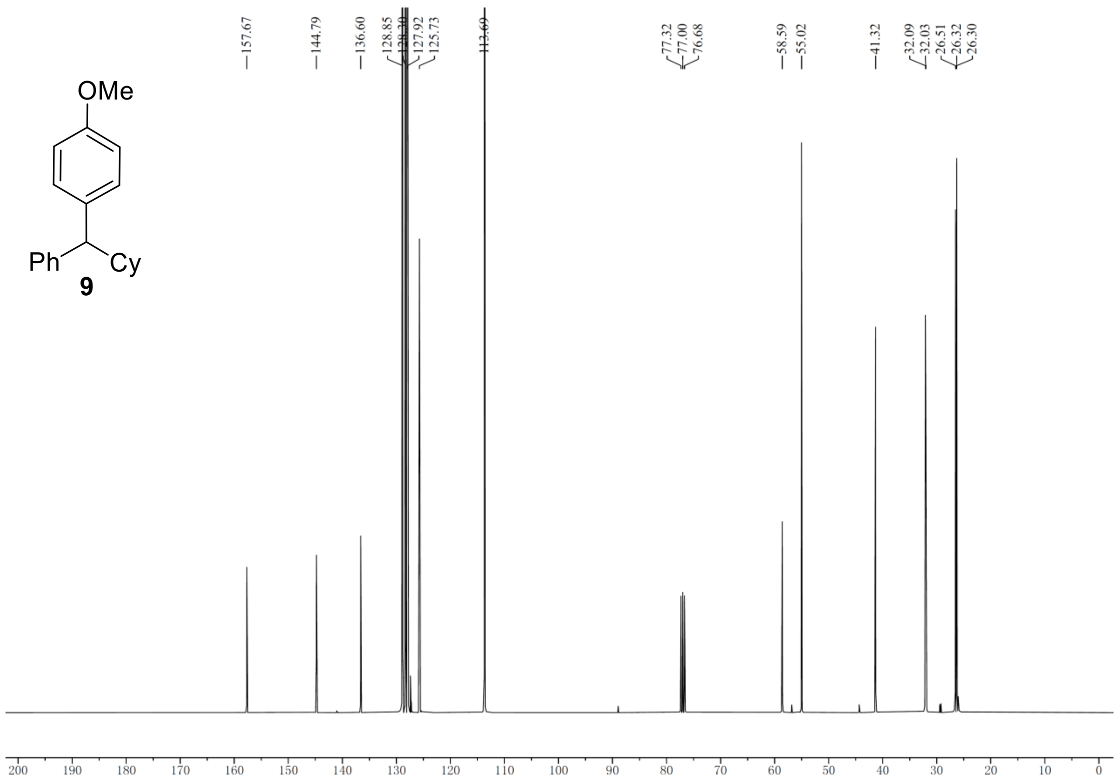

Supplementary Fig. 109 ^{13}C NMR spectrum of compound 9 (CDCl$_3$, 100 MHz, 298K)

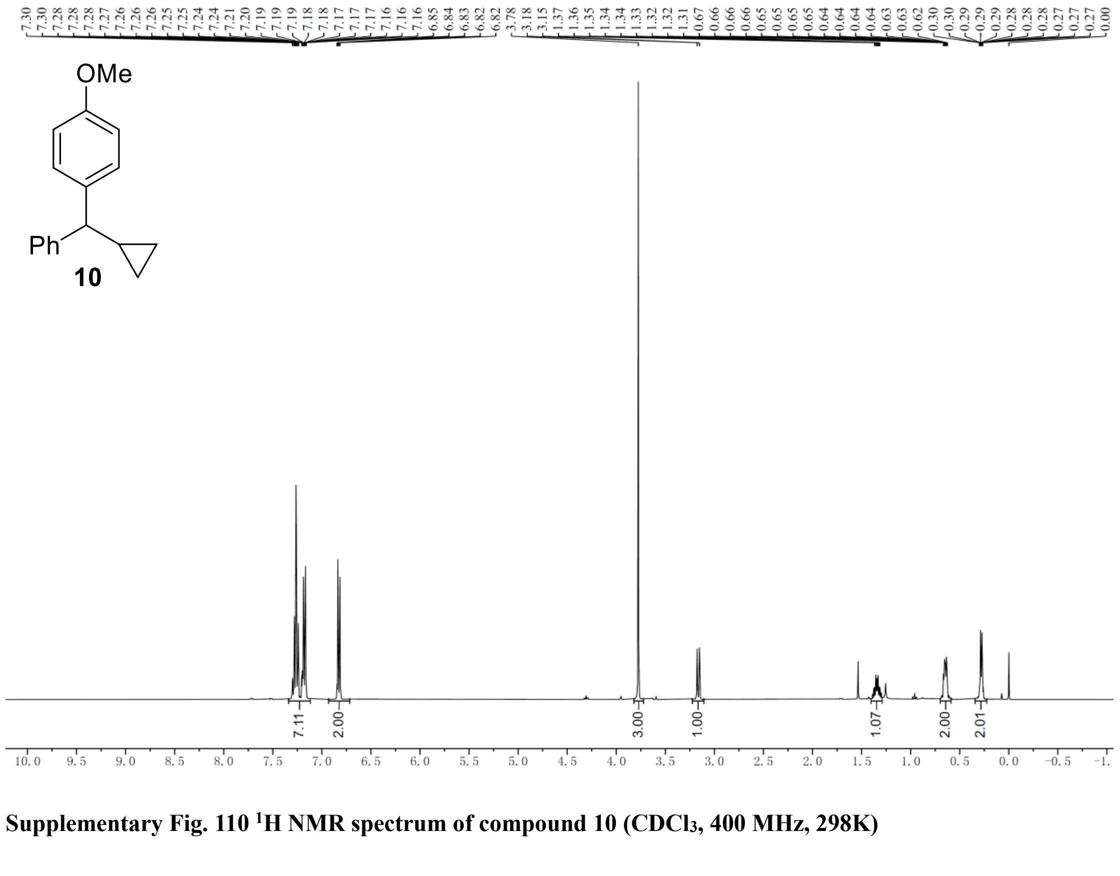

Supplementary Fig. 110 ^{1}H NMR spectrum of compound 10 (CDCl$_3$, 400 MHz, 298K)

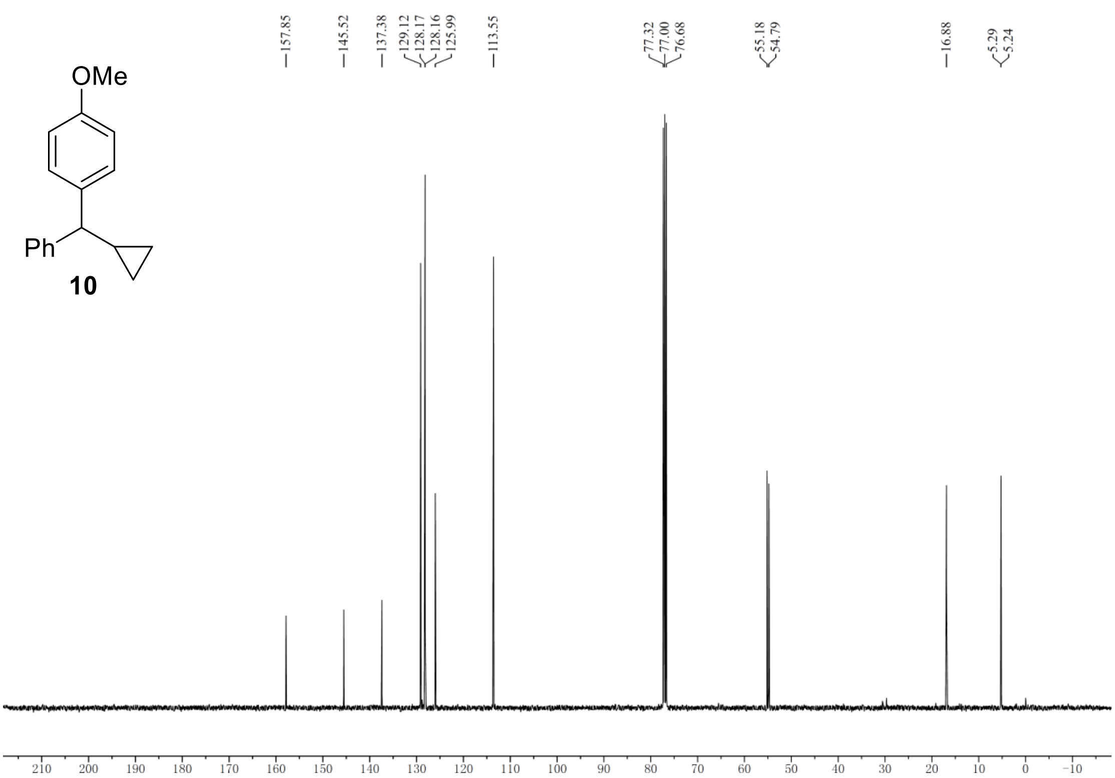

Supplementary Fig. 111 ^{13}C NMR spectrum of compound 10 ($CDCl_3$, 100 MHz, 298K)

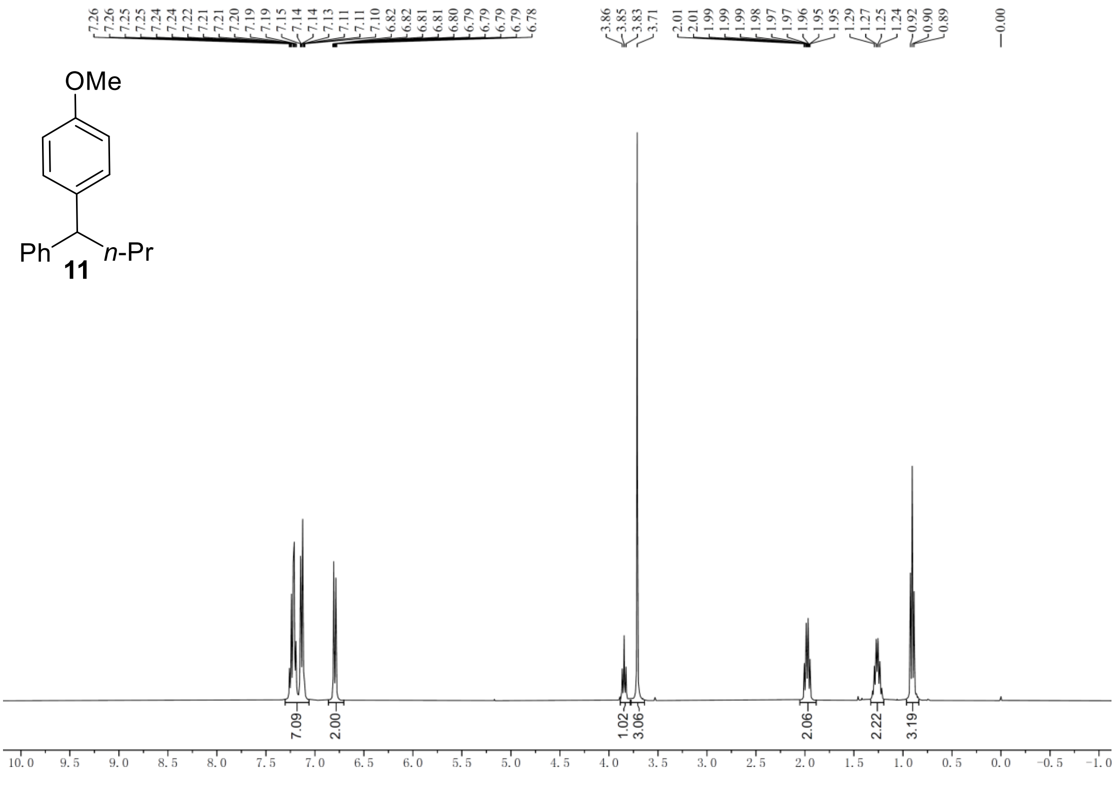

Supplementary Fig. 112 ^{1}H NMR spectrum of compound 11 ($CDCl_3$, 400 MHz, 298K)

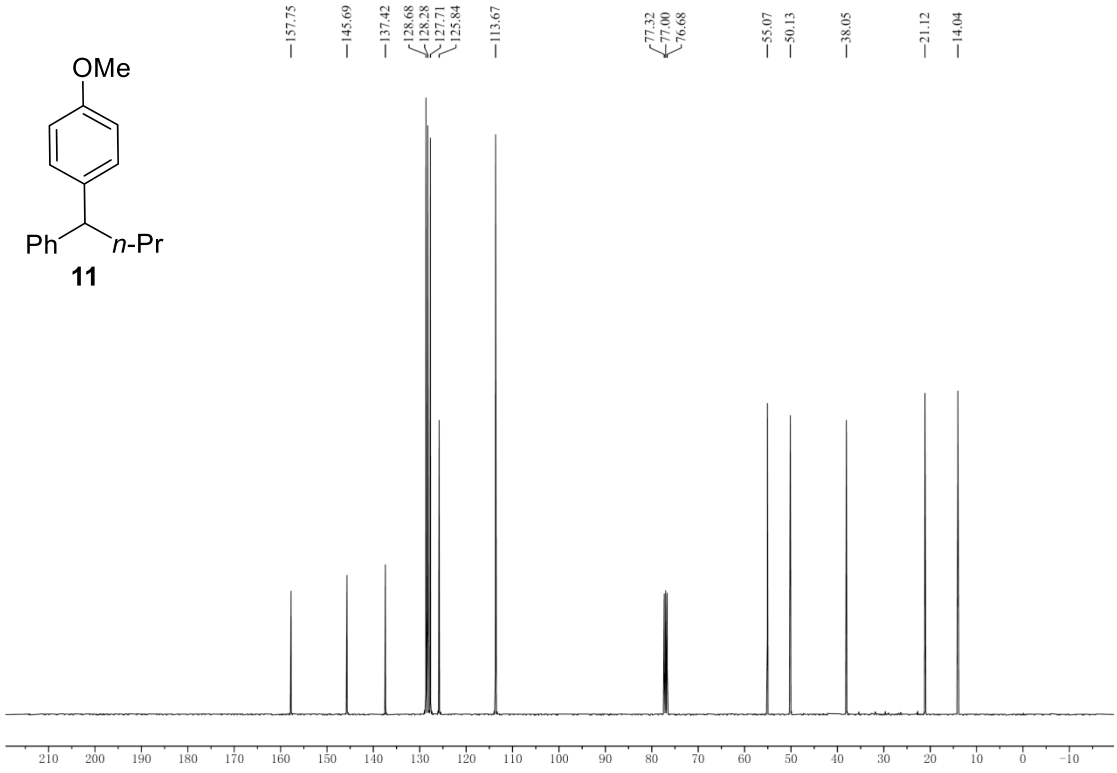

Supplementary Fig. 113 ^{13}C NMR spectrum of compound 11 ($CDCl_3$, 100 MHz, 298K)

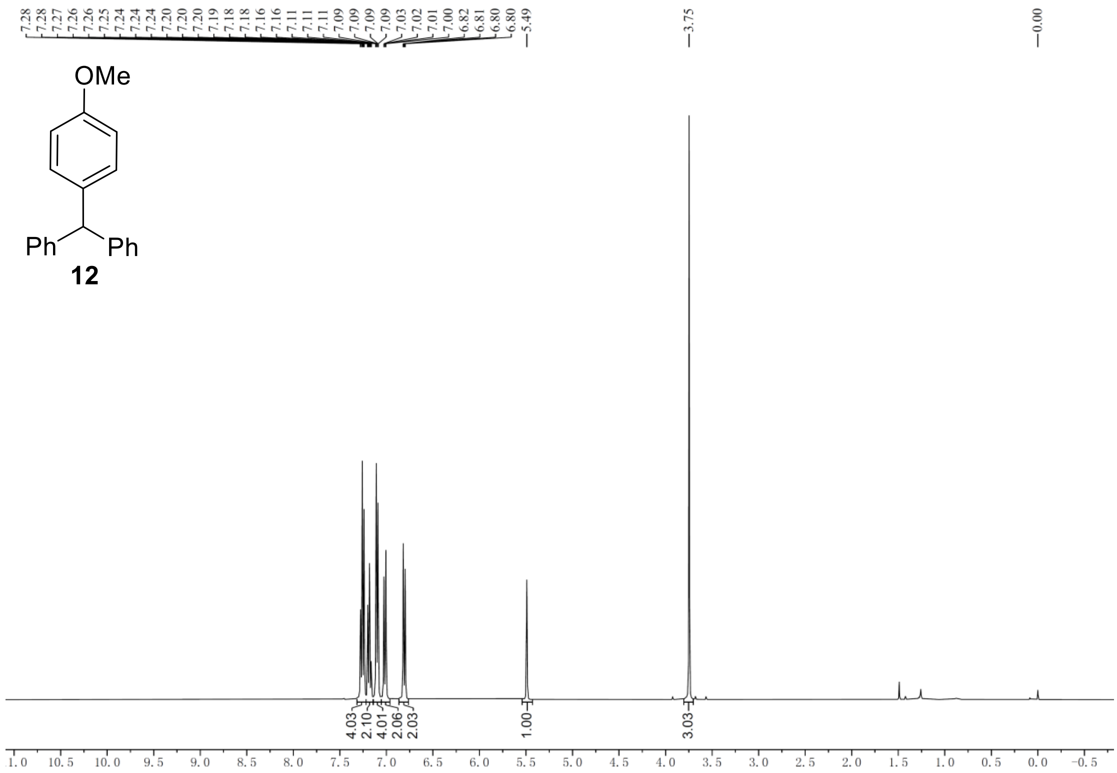

Supplementary Fig. 114 ^{1}H NMR spectrum of compound 12 ($CDCl_3$, 400 MHz, 298K)

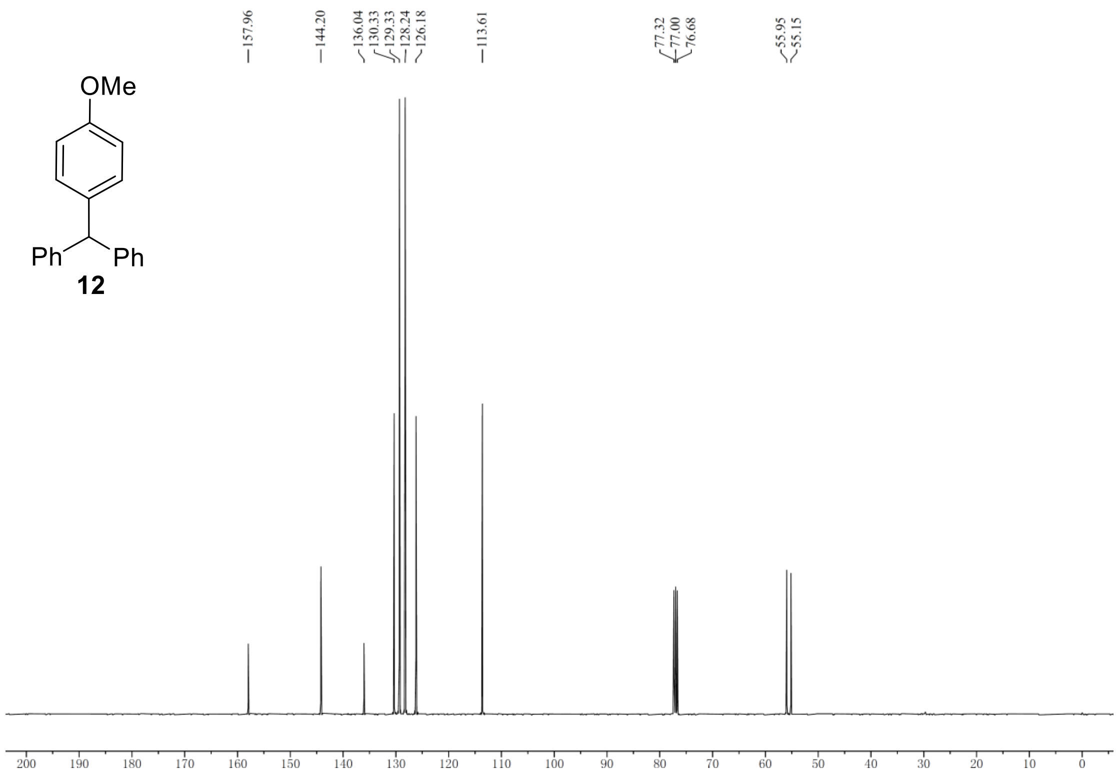

Supplementary Fig. 115 ^{13}C NMR spectrum of compound 12 ($CDCl_3$, 100 MHz, 298K)

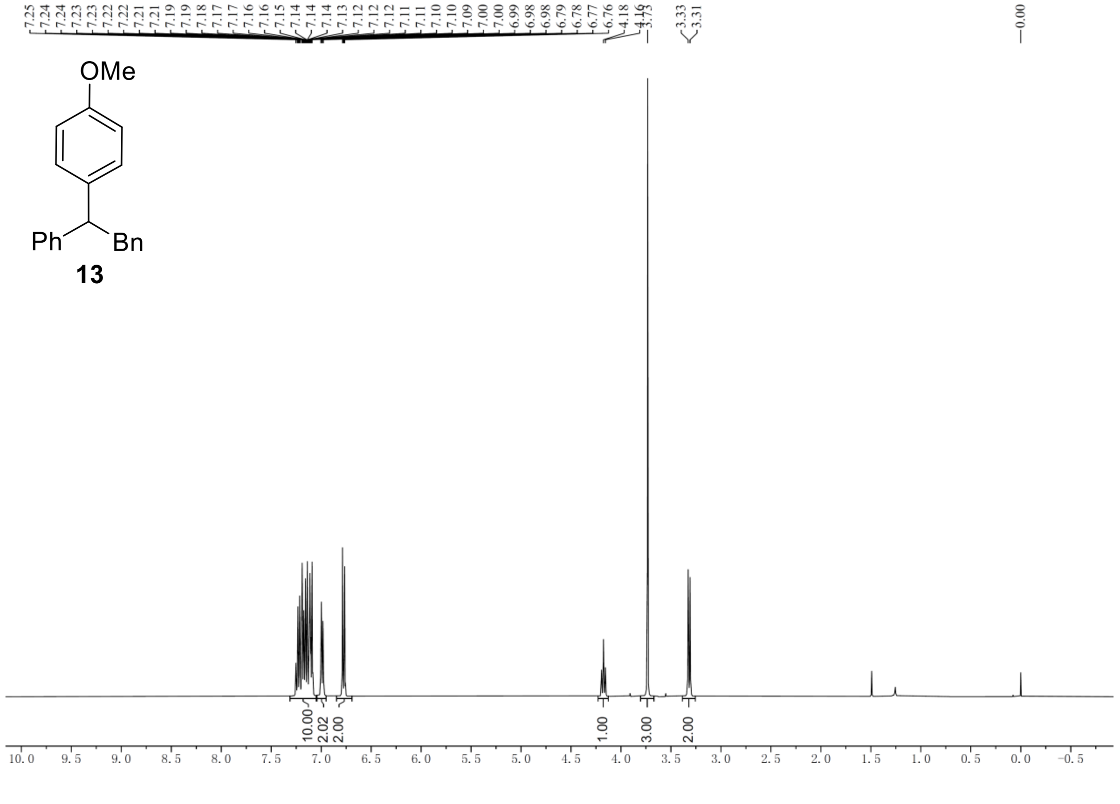

Supplementary Fig. 116 ^{1}H NMR spectrum of compound 13 ($CDCl_3$, 400 MHz, 298K)

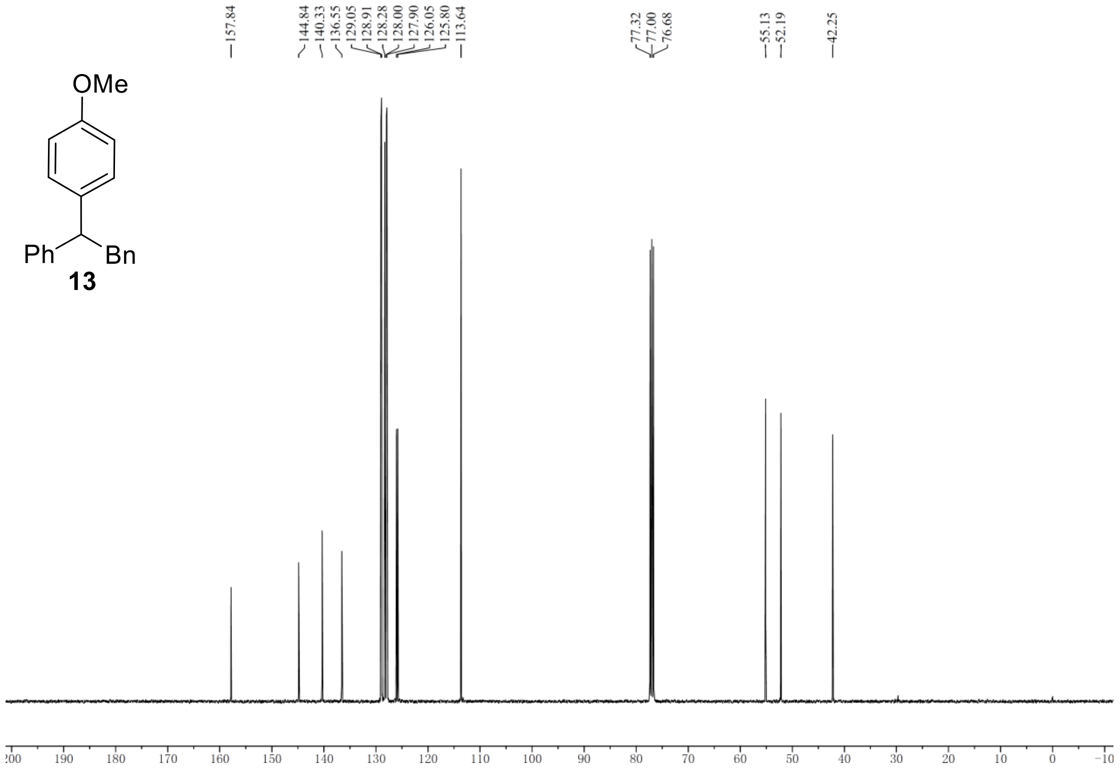

Supplementary Fig. 117 ^{13}C NMR spectrum of compound 13 ($CDCl_3$, 100 MHz, 298K)

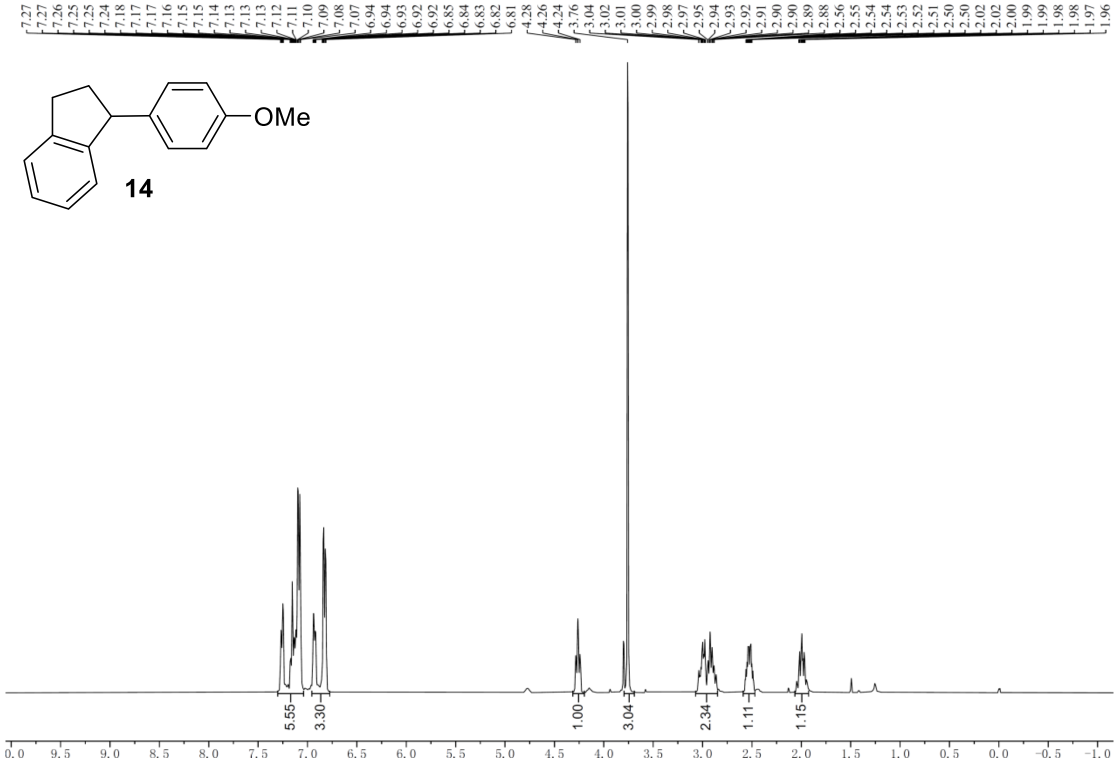

Supplementary Fig. 118 ^{1}H NMR spectrum of compound 14 ($CDCl_3$, 400 MHz, 298K)

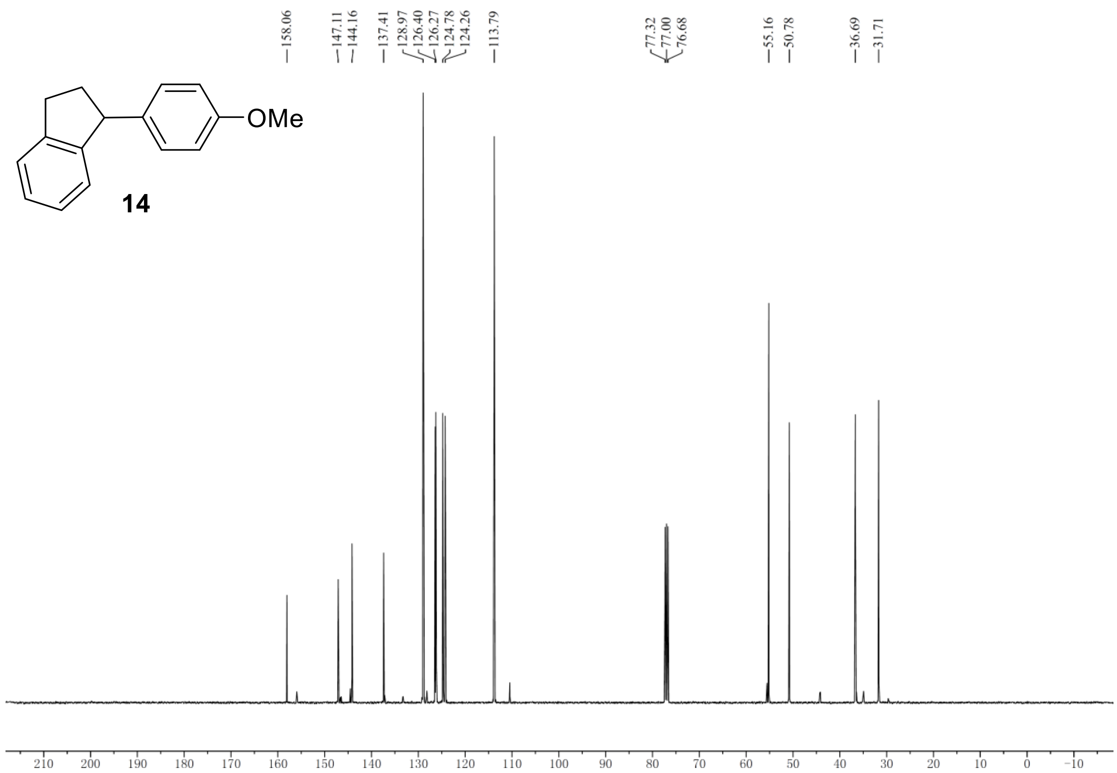

Supplementary Fig. 119 ^{13}C NMR spectrum of compound 14 ($CDCl_3$, 100 MHz, 298K)

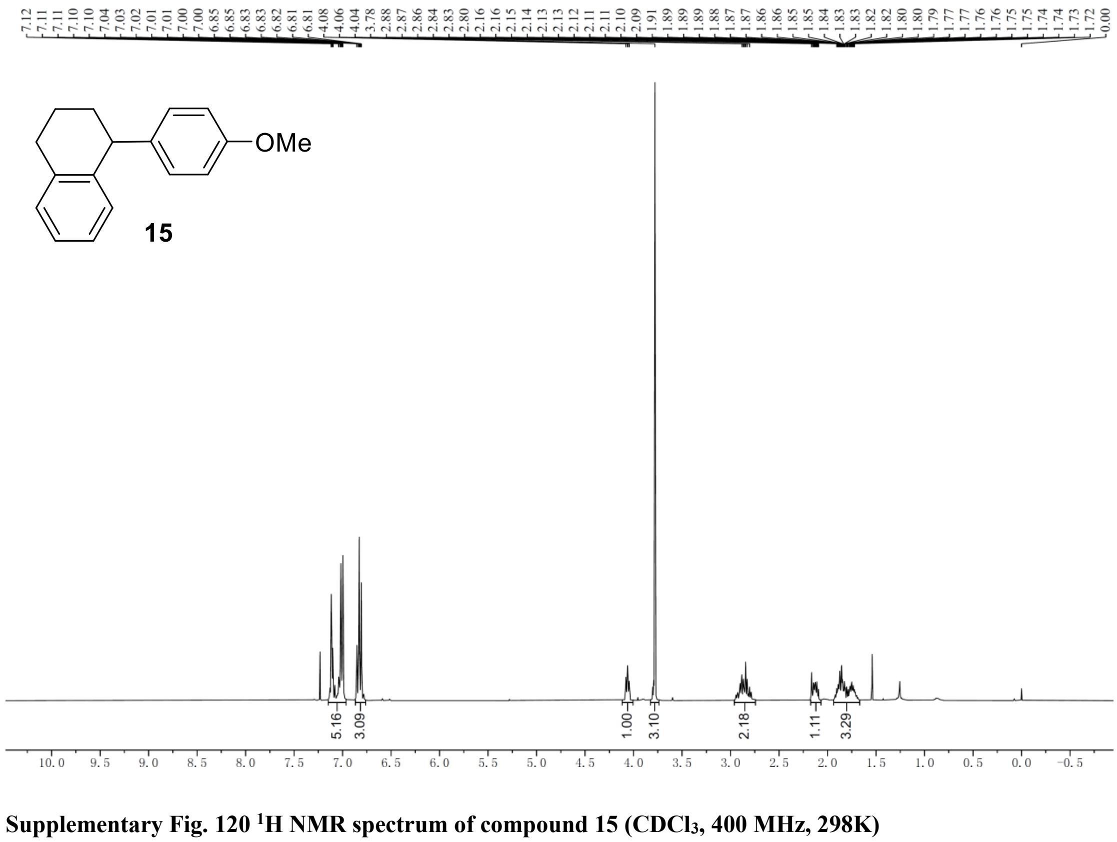

Supplementary Fig. 120 ^{1}H NMR spectrum of compound 15 ($CDCl_3$, 400 MHz, 298K)

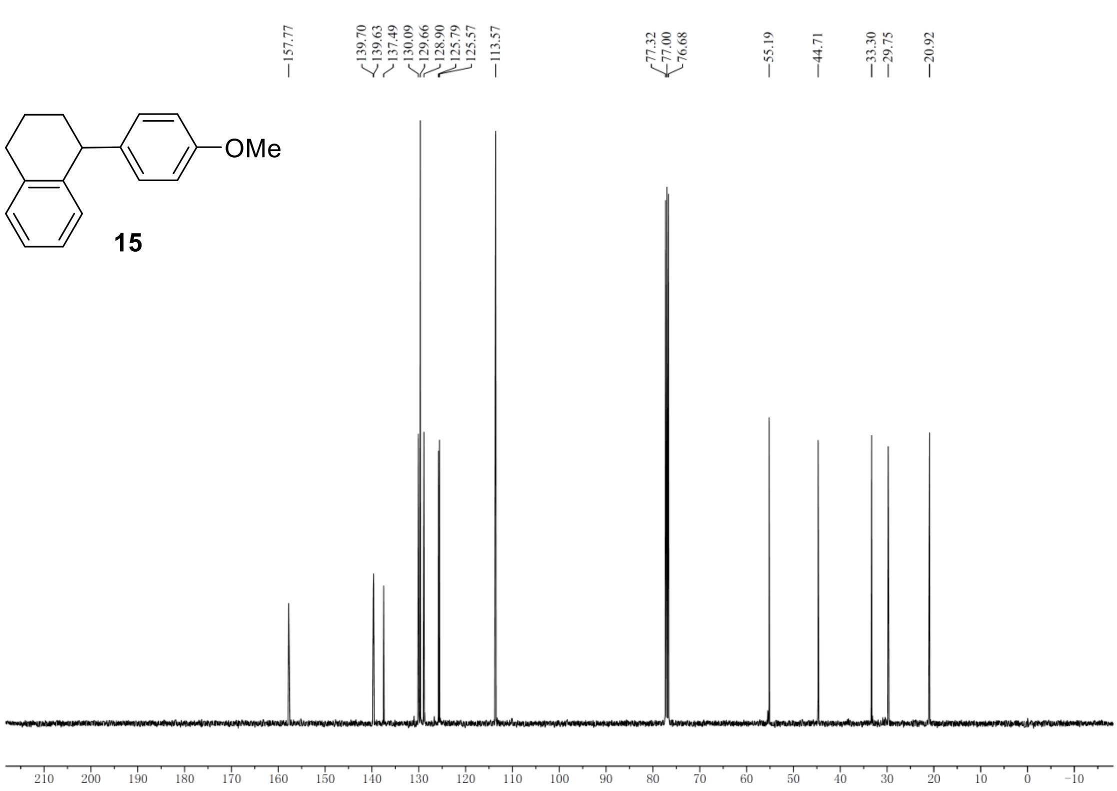

Supplementary Fig. 121 ^{13}C NMR spectrum of compound 15 ($CDCl_3$, 100 MHz, 298K)

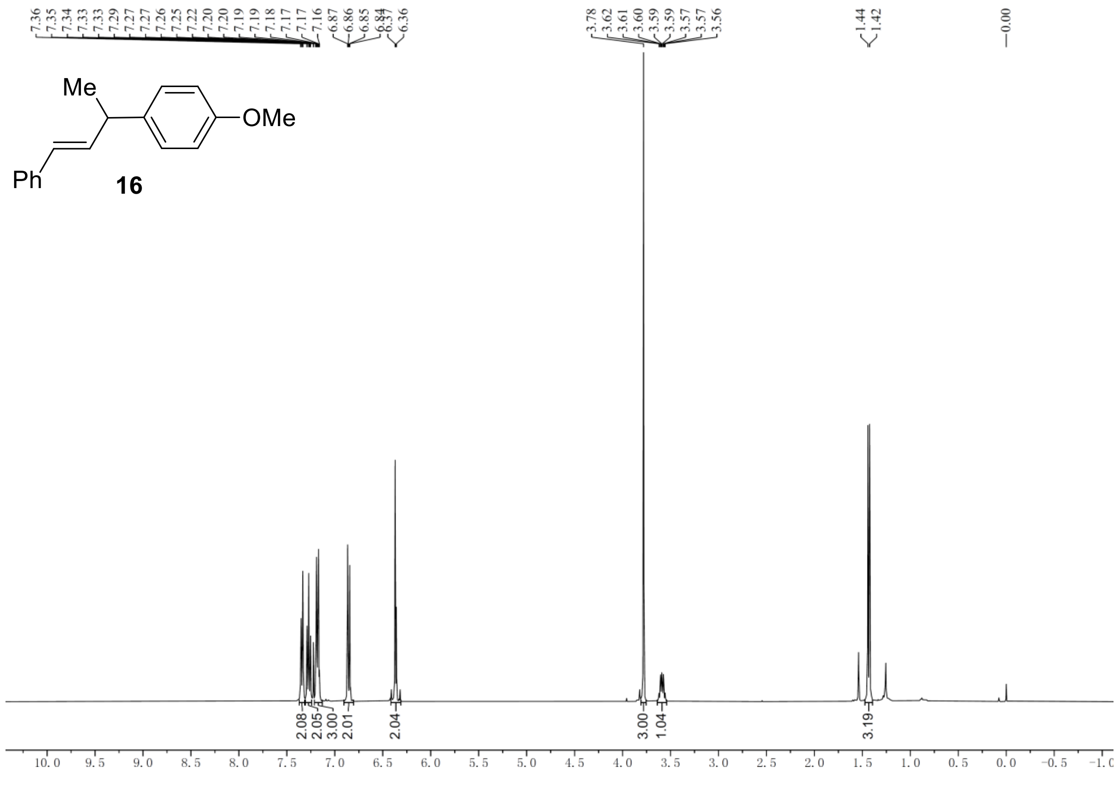

Supplementary Fig. 122 ^{1}H NMR spectrum of compound 16 ($CDCl_3$, 400 MHz, 298K)

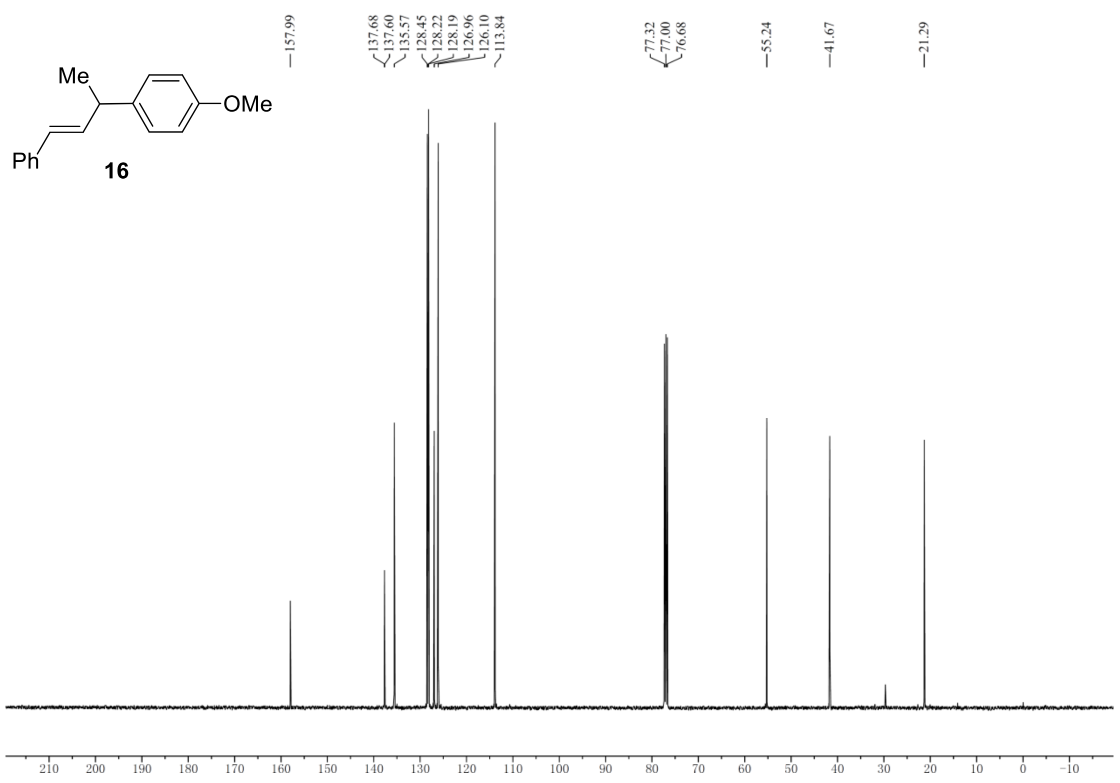

Supplementary Fig. 123 ^{13}C NMR spectrum of compound 16 ($CDCl_3$, 100 MHz, 298K)

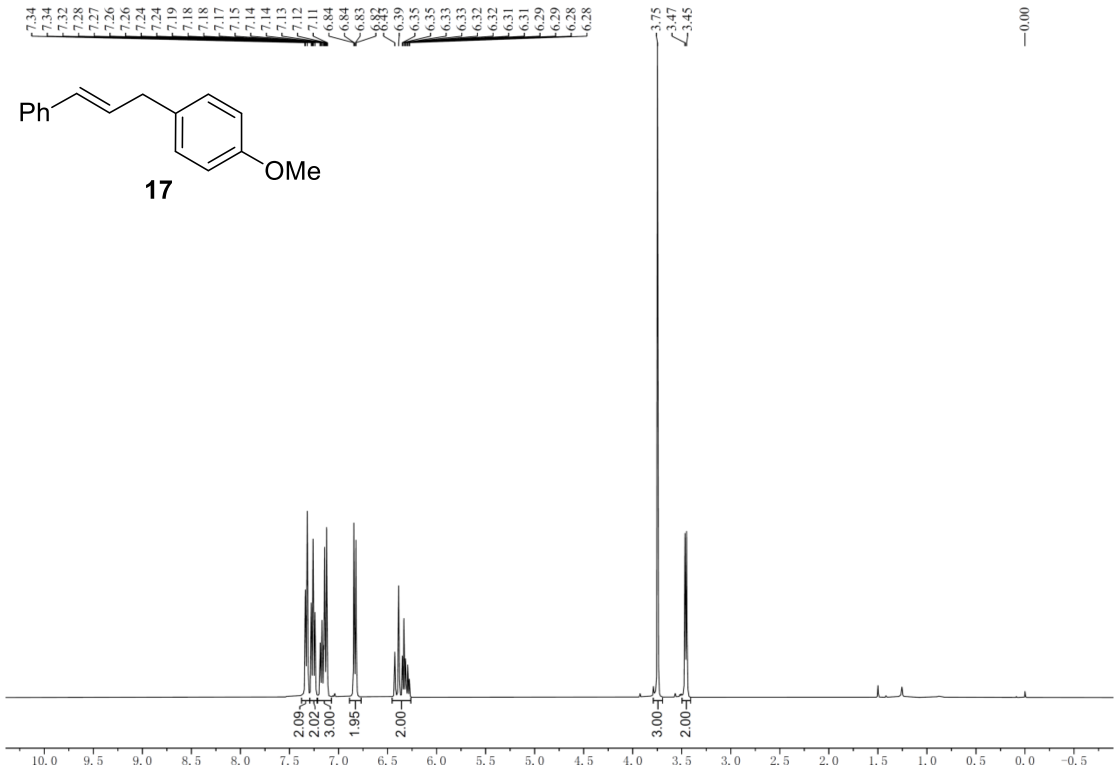

Supplementary Fig. 124 ^{1}H NMR spectrum of compound 17 ($CDCl_3$, 400 MHz, 298K)

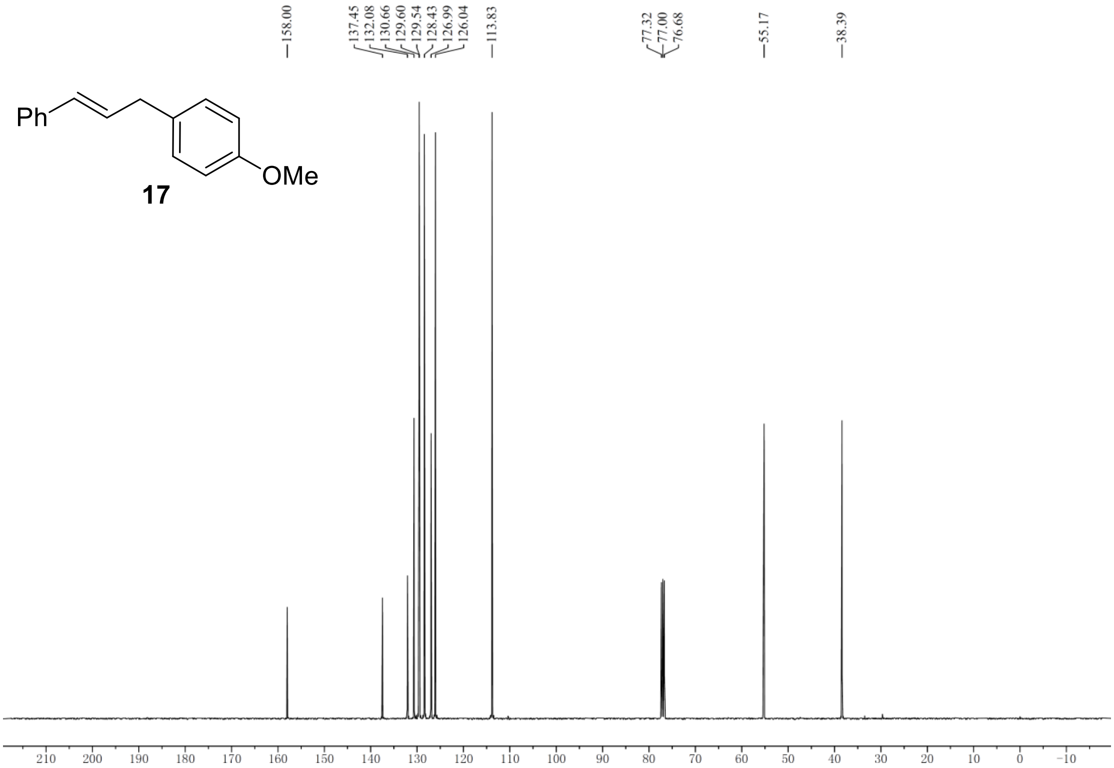

Supplementary Fig. 125 ^{13}C NMR spectrum of compound 17 ($CDCl_3$, 100 MHz, 298K)

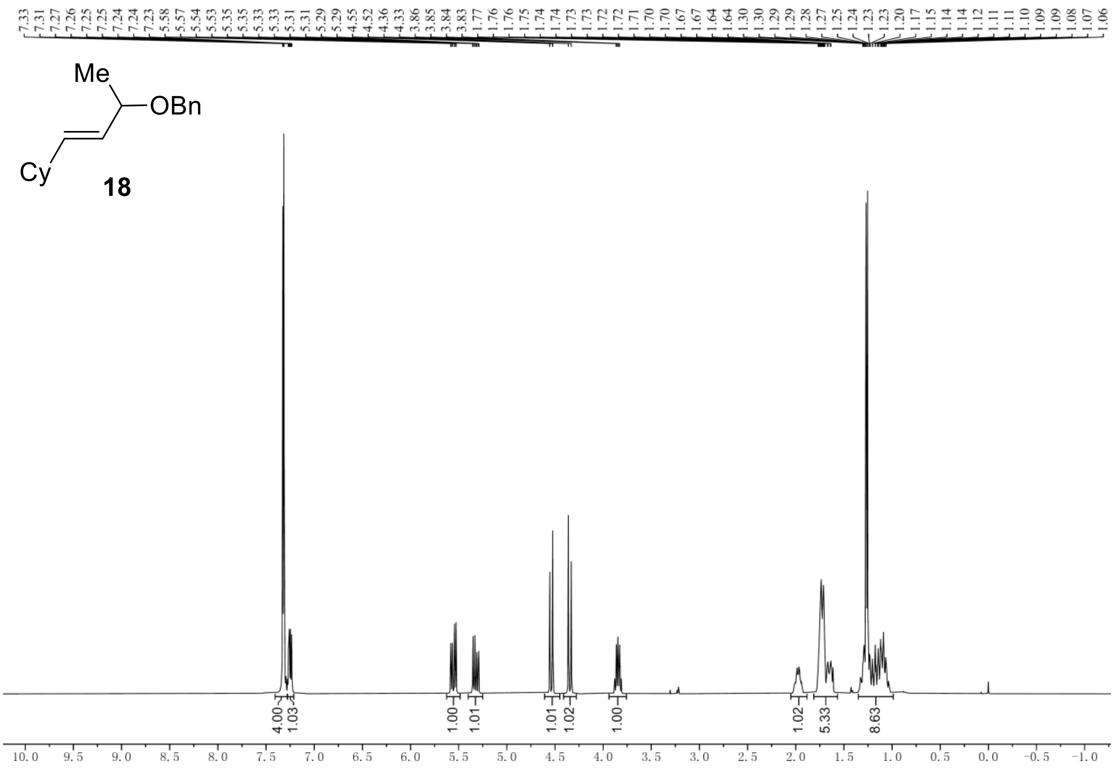

Supplementary Fig. 126 ^{1}H NMR spectrum of compound 18 ($CDCl_3$, 400 MHz, 298K)

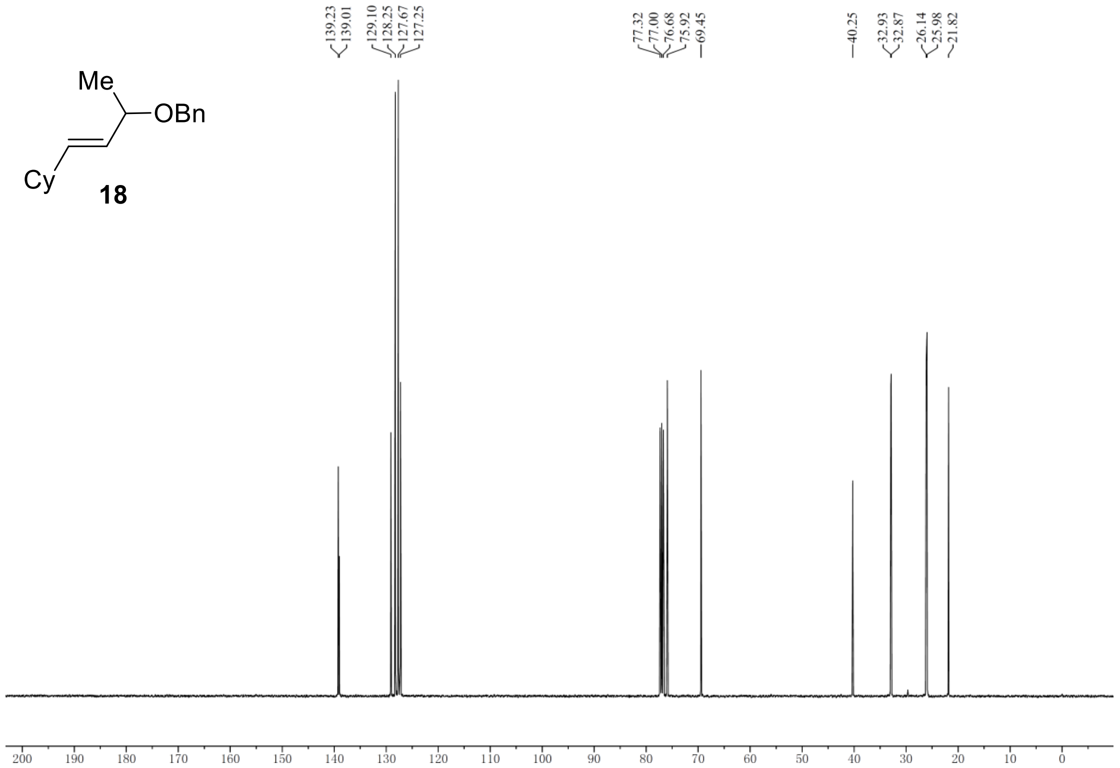

Supplementary Fig. 127 ^{13}C NMR spectrum of compound 18 ($CDCl_3$, 100 MHz, 298K)

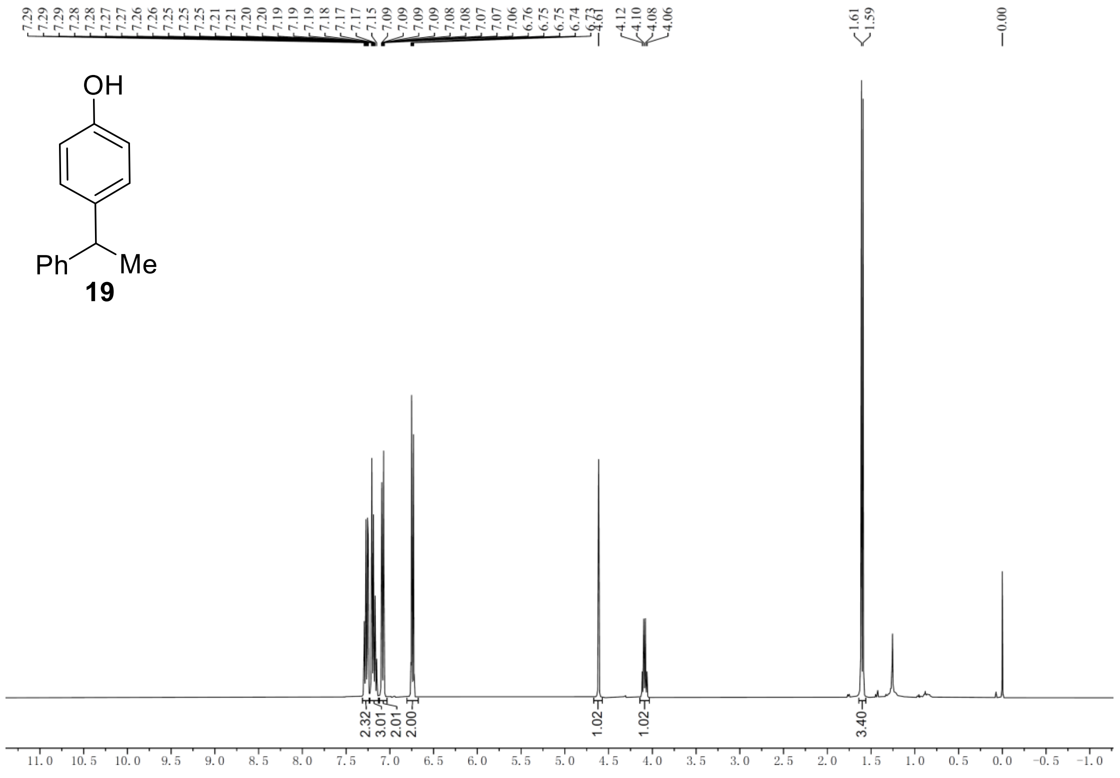

Supplementary Fig. 128 ^{1}H NMR spectrum of compound 19 ($CDCl_3$, 400 MHz, 298K)

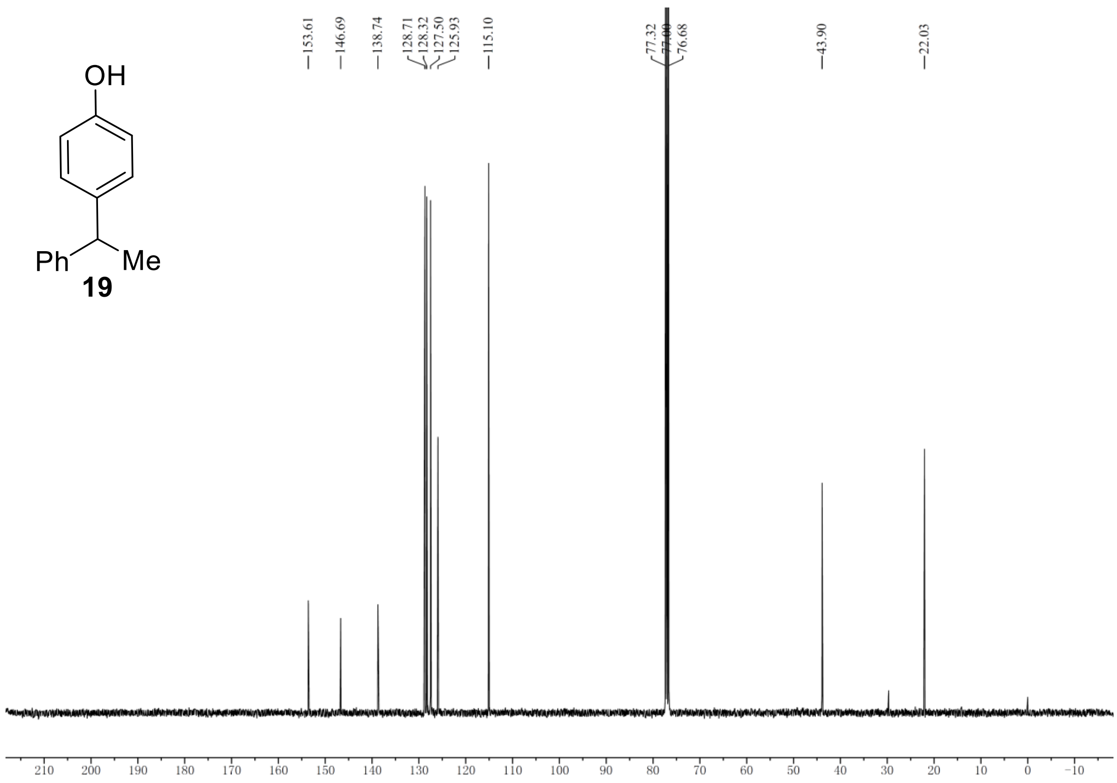

Supplementary Fig. 129 ^{13}C NMR spectrum of compound 19 ($CDCl_3$, 100 MHz, 298K)

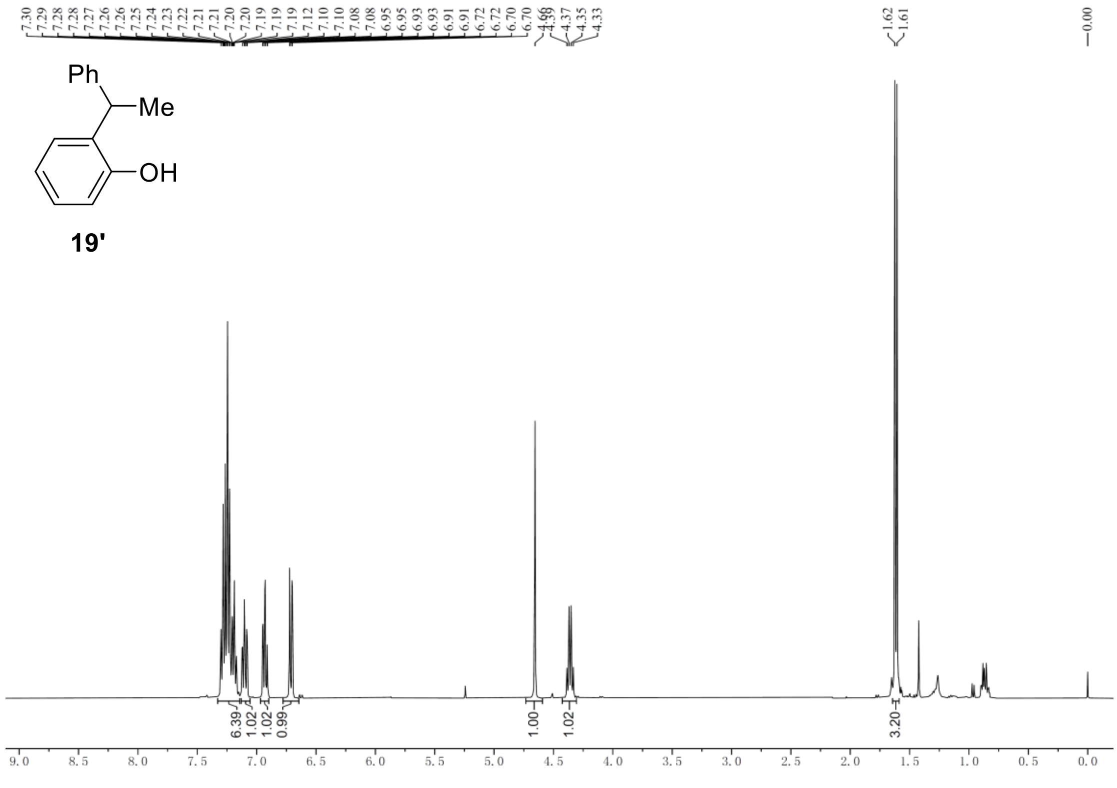

Supplementary Fig. 130 ^{1}H NMR spectrum of compound 19' ($CDCl_3$, 400 MHz, 298K)

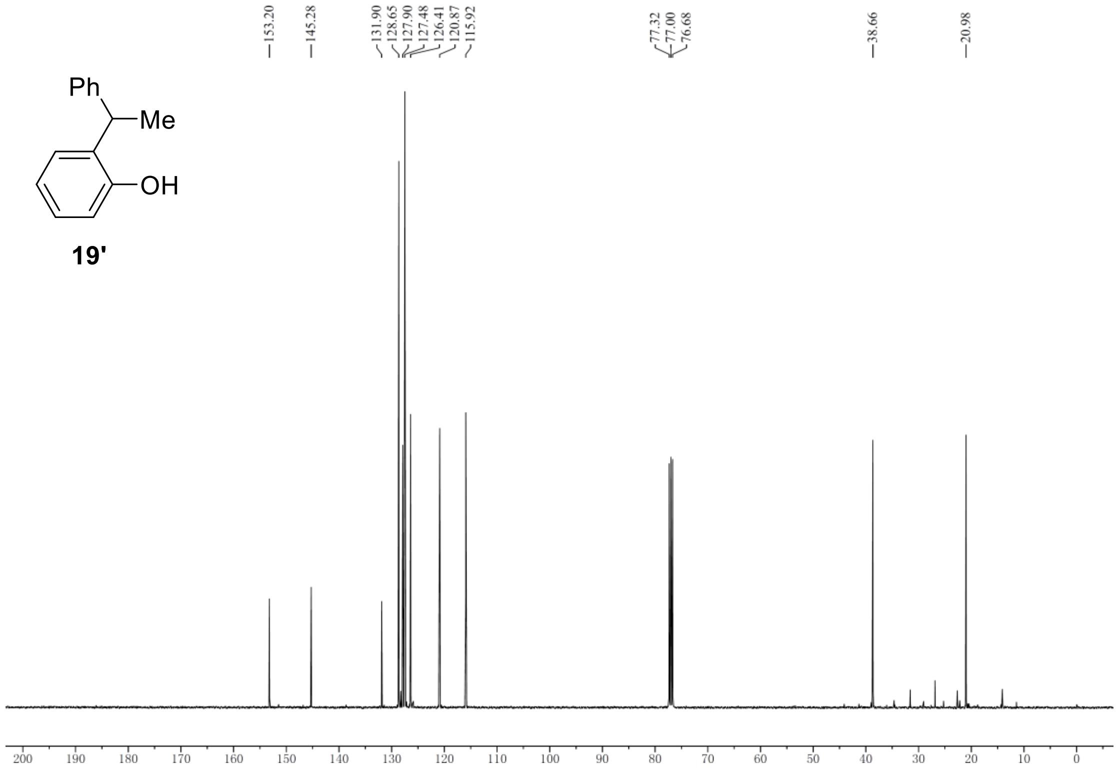

Supplementary Fig. 131 ^{13}C NMR spectrum of compound 19' ($CDCl_3$, 100 MHz, 298K)

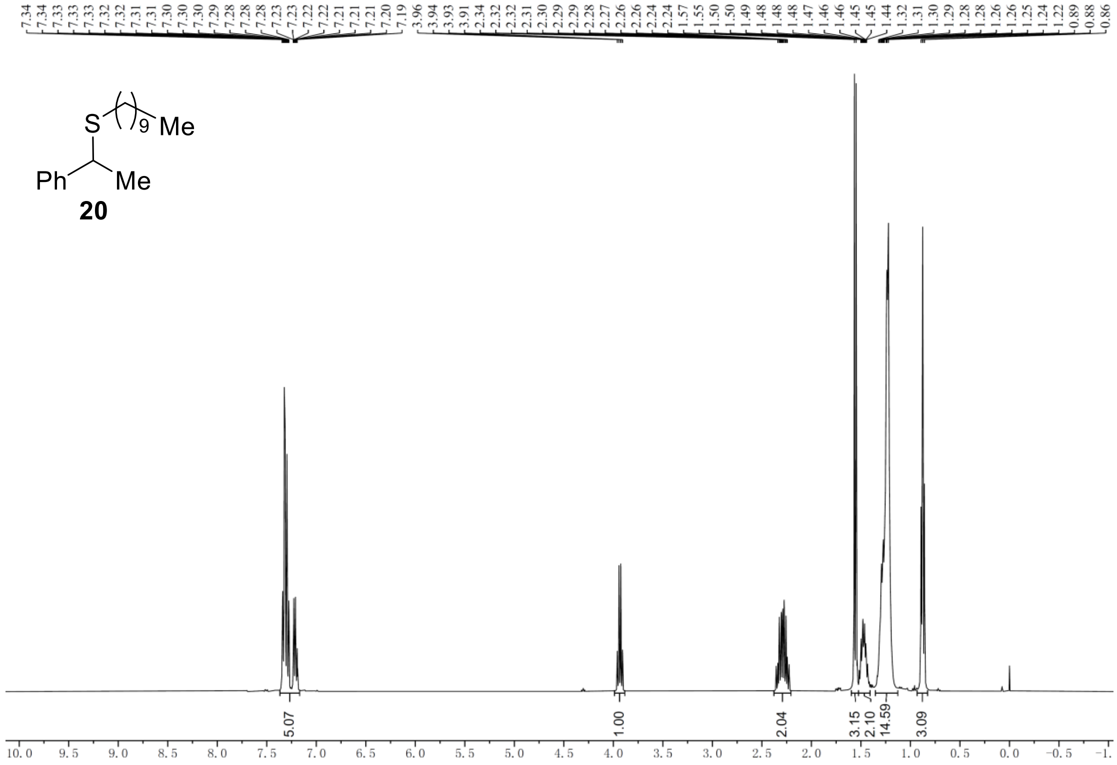

Supplementary Fig. 132 ^{1}H NMR spectrum of compound 20 ($CDCl_3$, 400 MHz, 298K)

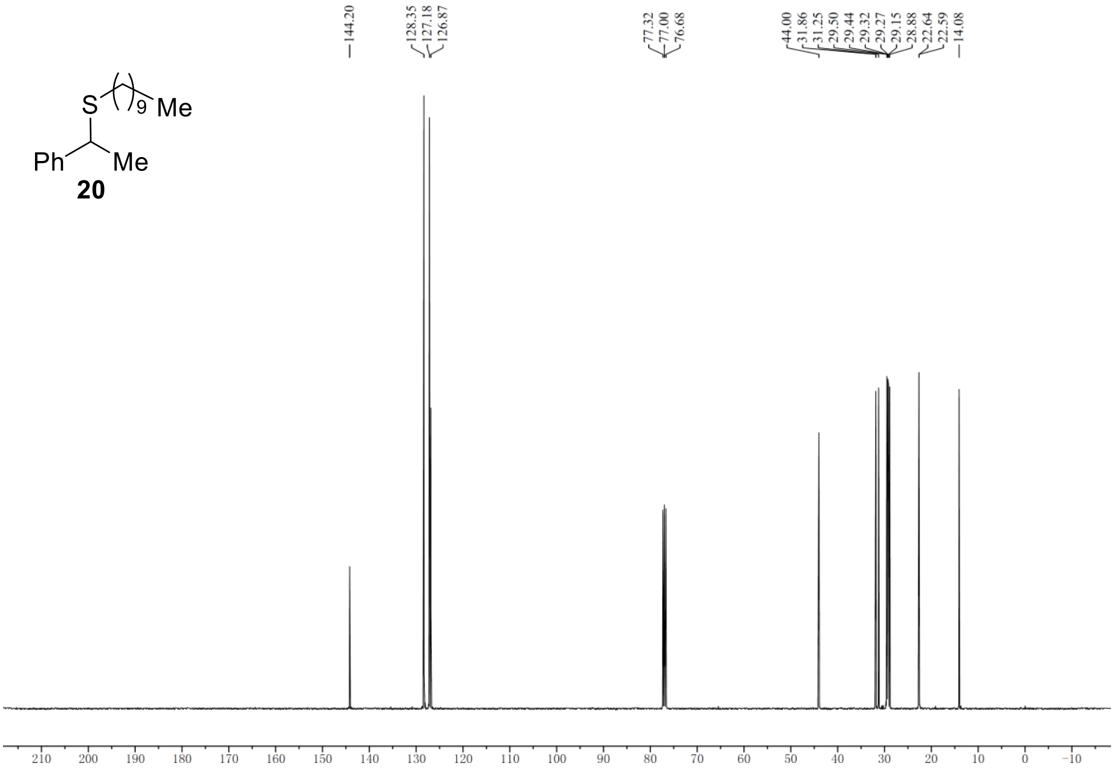

Supplementary Fig. 133 ^{13}C NMR spectrum of compound 20 ($CDCl_3$, 100 MHz, 298K)

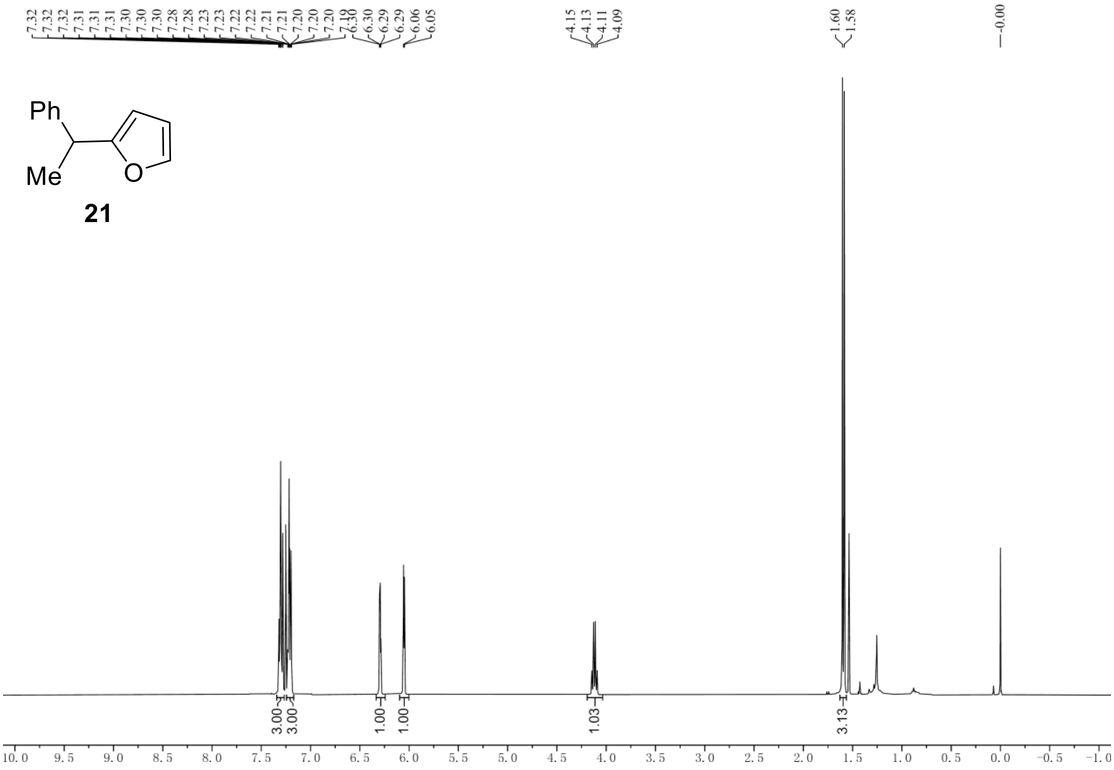

Supplementary Fig. 134 ^{1}H NMR spectrum of compound 21 ($CDCl_3$, 400 MHz, 298K)

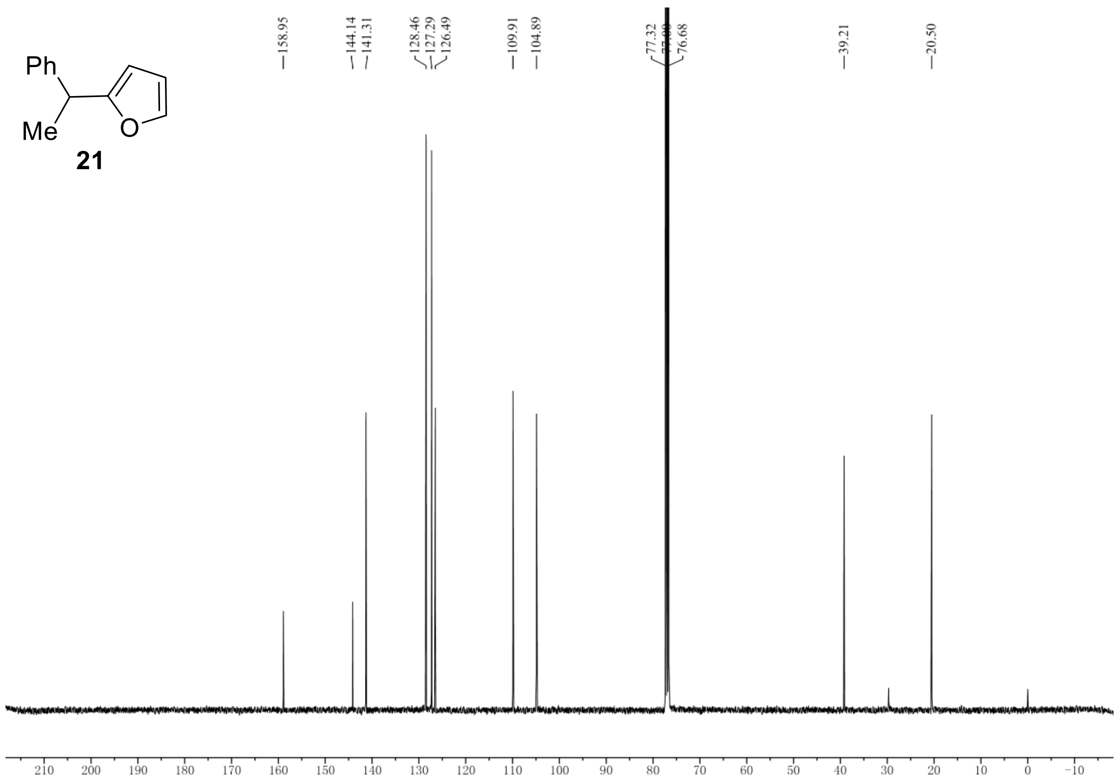

Supplementary Fig. 135 ^{13}C NMR spectrum of compound 21 ($CDCl_3$, 100 MHz, 298K)

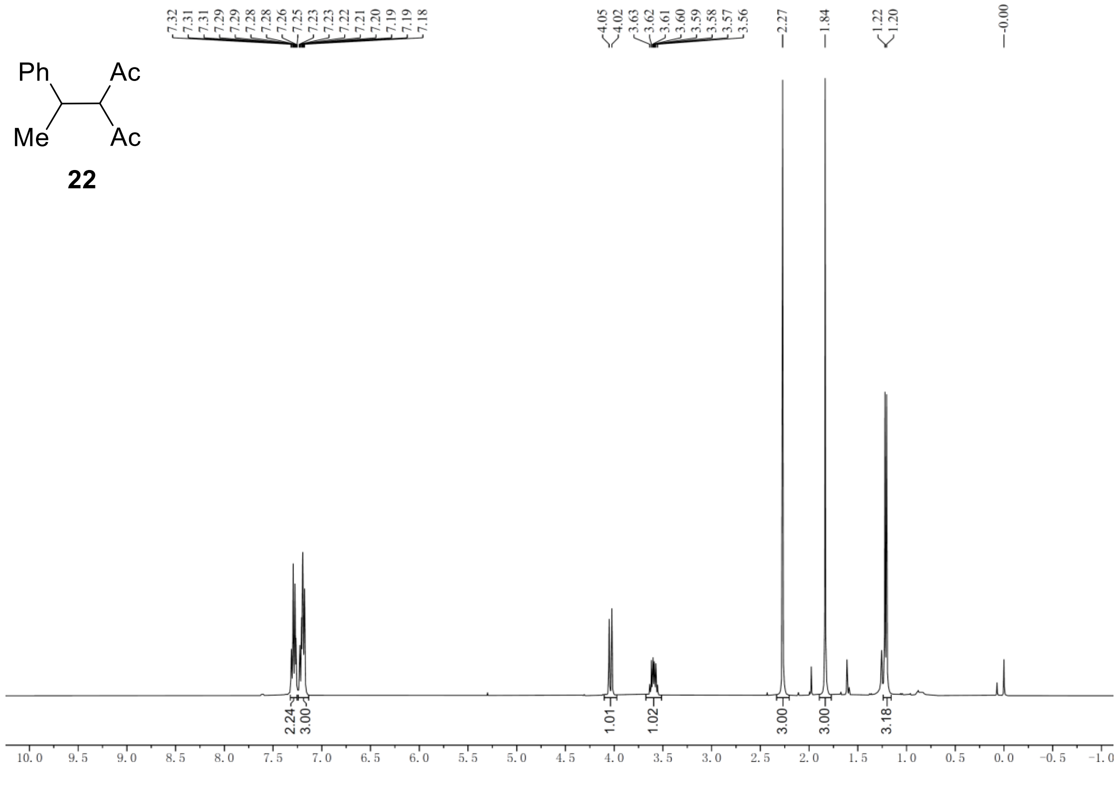

Supplementary Fig. 136 ^{1}H NMR spectrum of compound 22 ($CDCl_3$, 400 MHz, 298K)

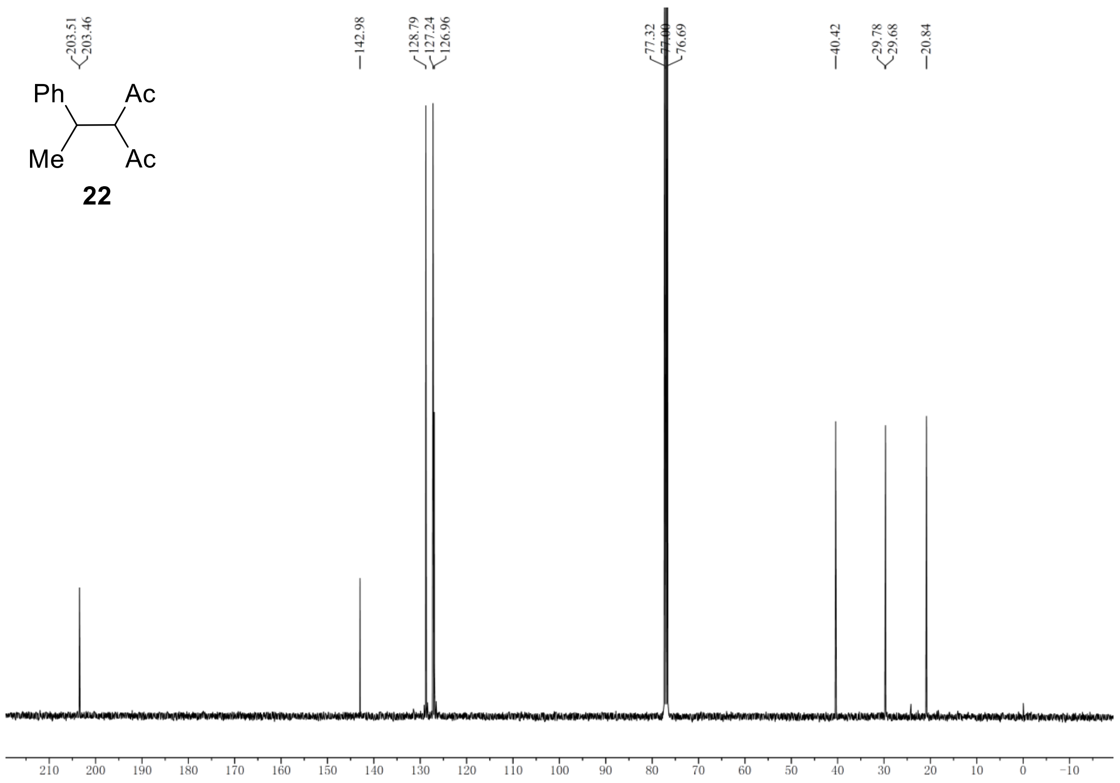

Supplementary Fig. 137 ^{13}C NMR spectrum of compound 22 (CDCl$_3$, 100 MHz, 298K)

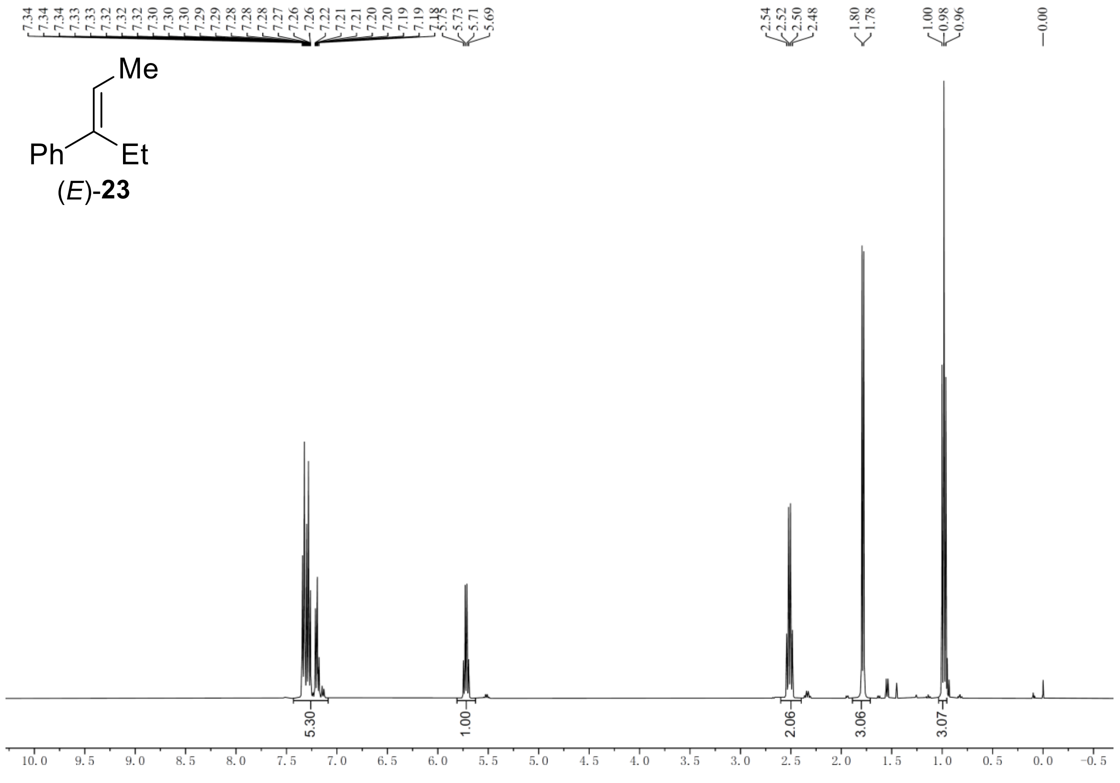

Supplementary Fig. 138 ^{1}H NMR spectrum of compound (*E*)-23 (CDCl$_3$, 400 MHz, 298K)

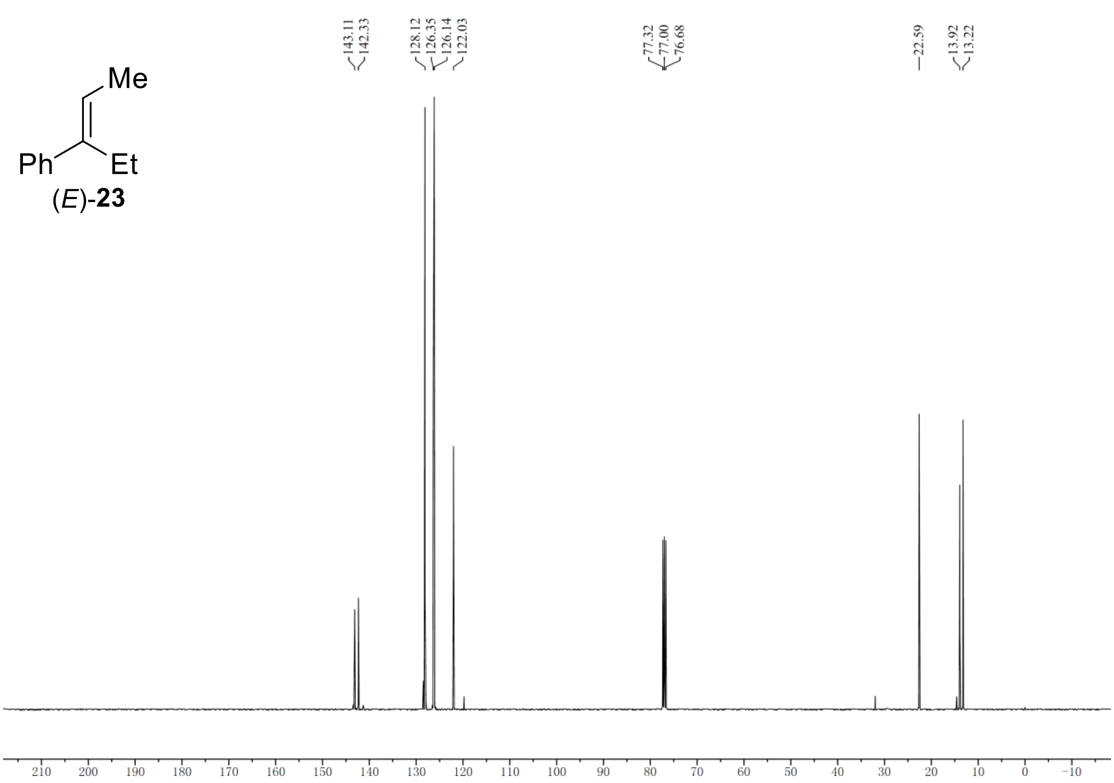

Supplementary Fig. 139 ^{13}C NMR spectrum of compound (*E*)-23 ($CDCl_3$, 100 MHz, 298K)

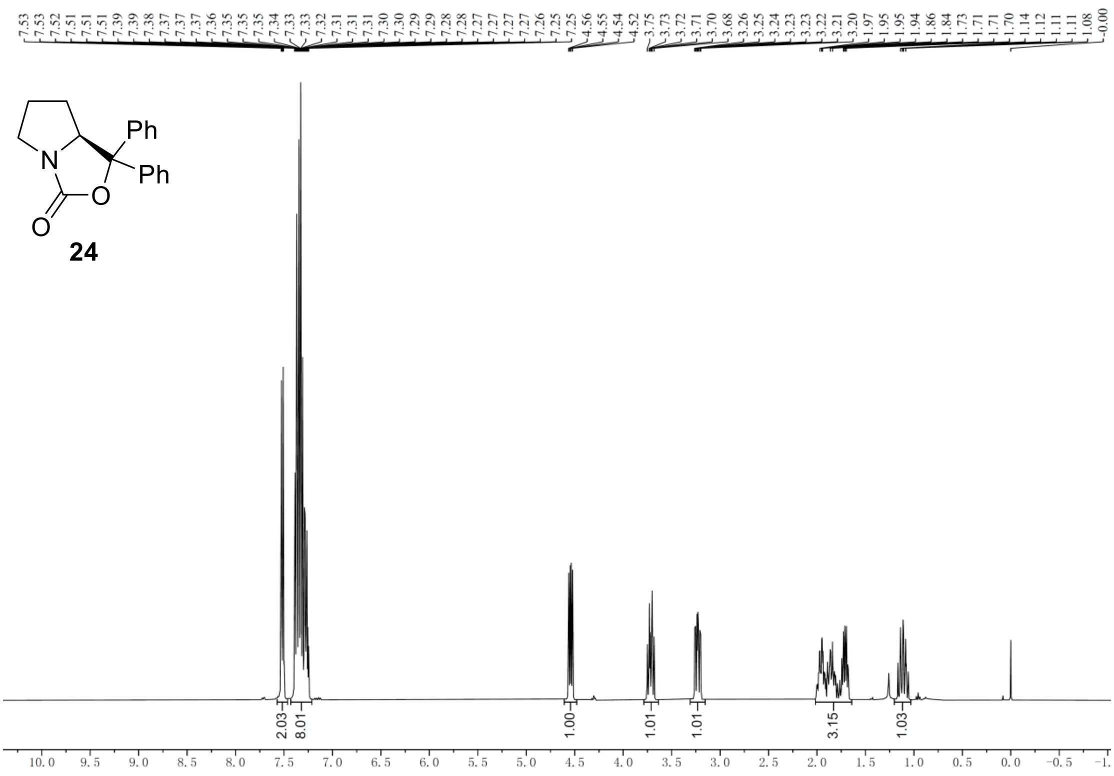

Supplementary Fig. 140 ^{1}H NMR spectrum of compound 24 ($CDCl_3$, 400 MHz, 298K)

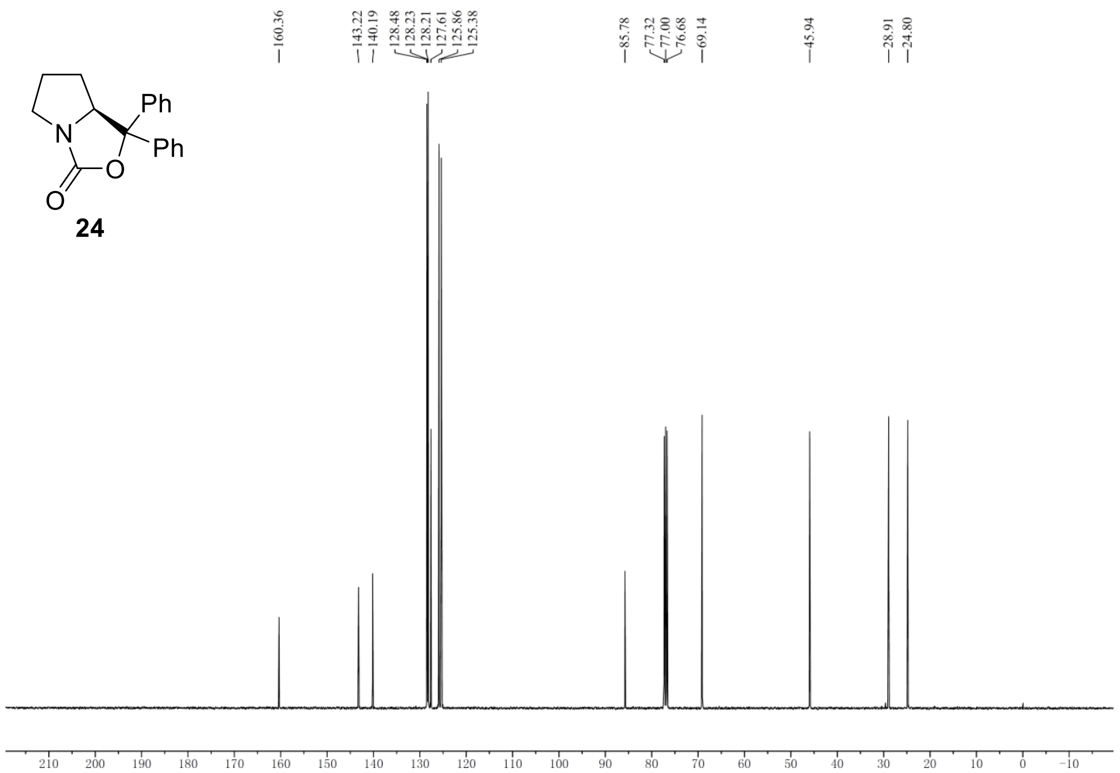

Supplementary Fig. 141 ^{13}C NMR spectrum of compound 24 ($CDCl_3$, 100 MHz, 298K)

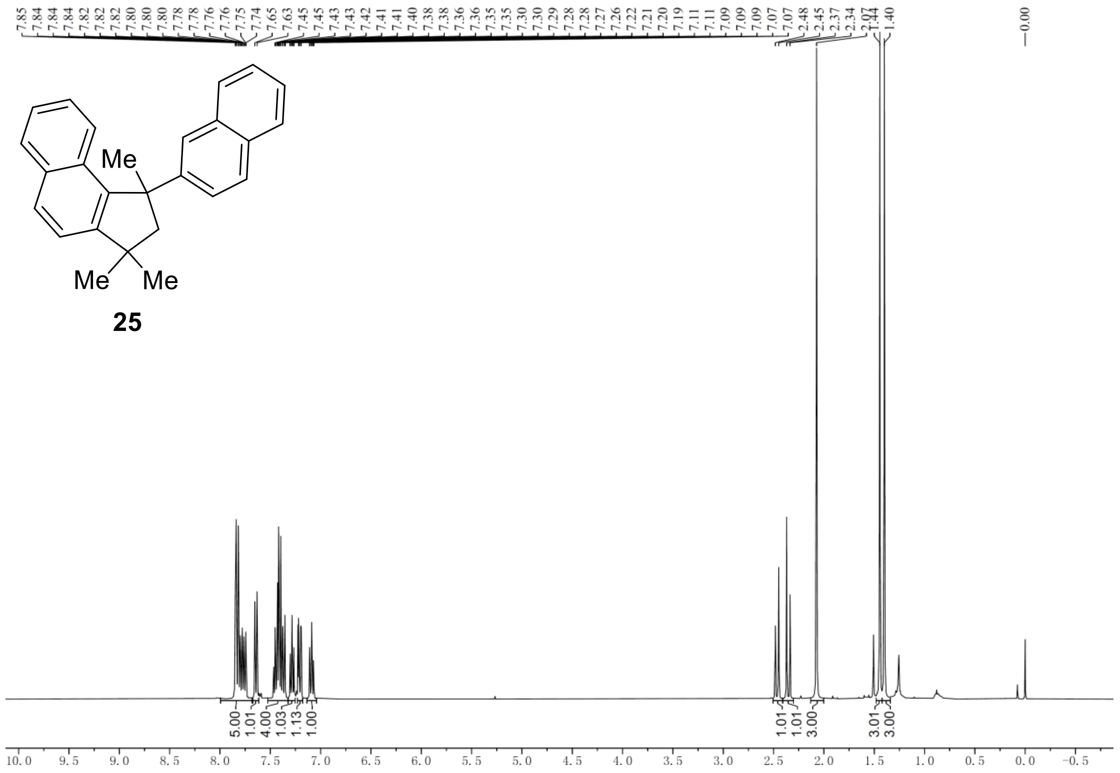

Supplementary Fig. 142 ^{1}H NMR spectrum of compound 25 ($CDCl_3$, 400 MHz, 298K)

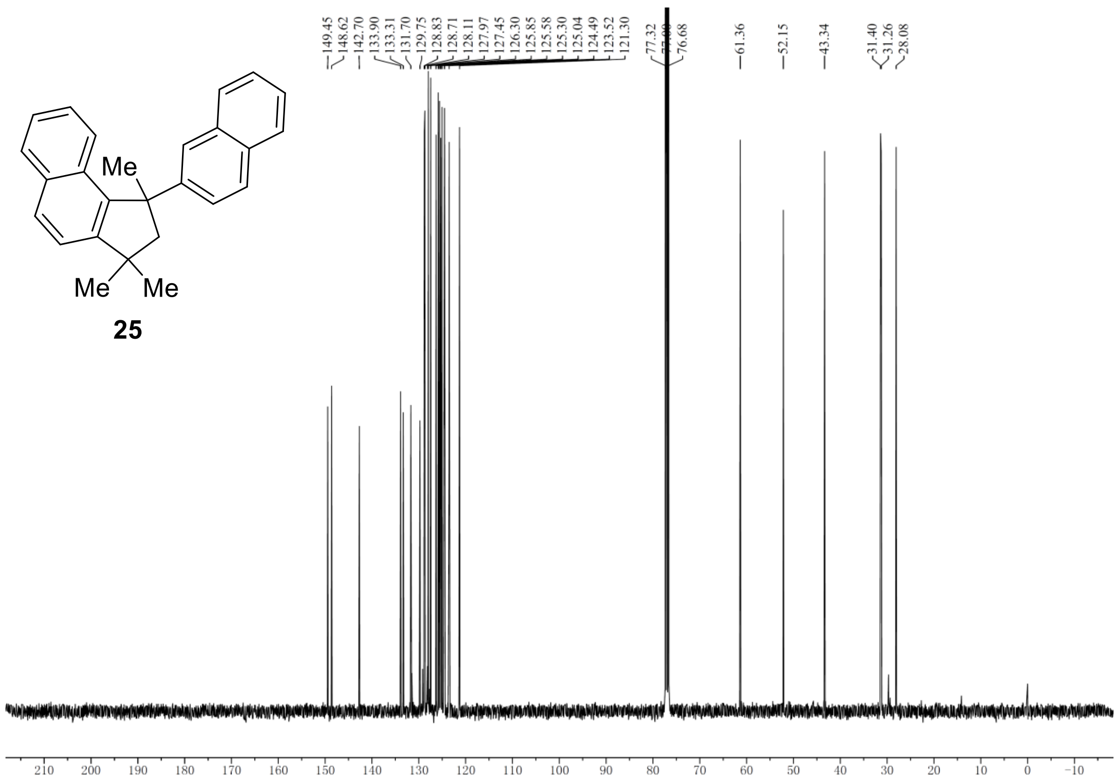

Supplementary Fig. 143 ^{13}C NMR spectrum of compound 25 ($CDCl_3$, 100 MHz, 298K)

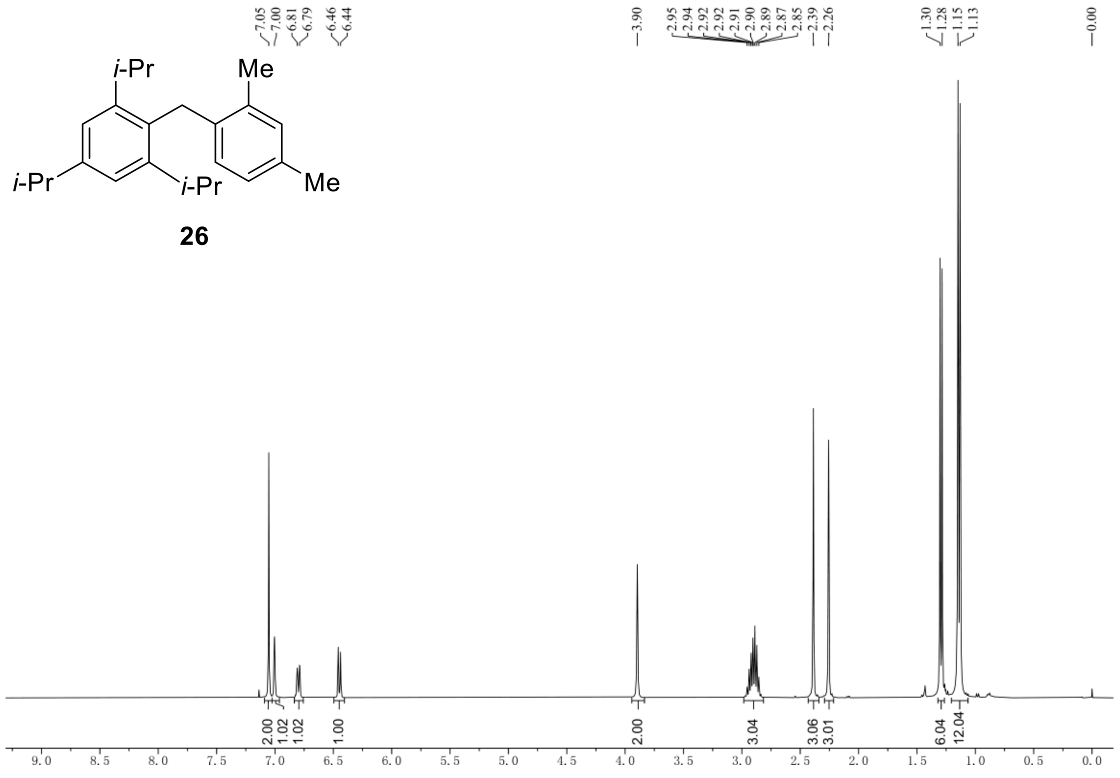

Supplementary Fig. 144 ^{1}H NMR spectrum of compound 26 ($CDCl_3$, 400 MHz, 298K)

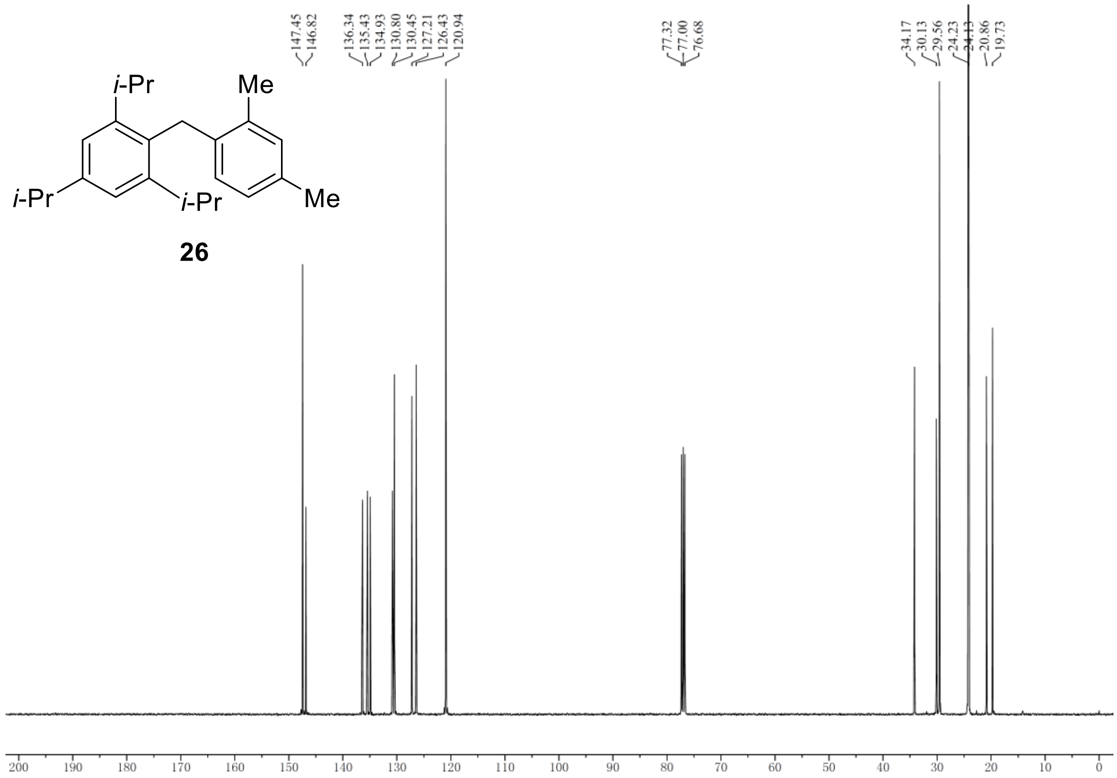

Supplementary Fig. 145 ^{13}C NMR spectrum of compound 26 ($CDCl_3$, 100 MHz, 298K)

10. References

1. Wang, W., Zhu, H., Liu, S., Zhao, Z., Zhang, L., Hao, J. & Wang, Y. Chalcogen-chalcogen bonding catalysis enables assembly of discrete molecules. *J. Am. Chem. Soc.* **141**, 9175-9179 (2019).

2. Wang, W., Zhu, H., Feng, L., Yu, Q., Hao, J., Zhu, R. & Wang, Y. Dual chalcogen-chalcogen bonding catalysis. *J. Am. Chem. Soc.* **142**, 3117-3124 (2020).

3. Kong, X., Zhou, P. & Wang, Y. Chalcogen•••π bonding catalysis. *Angew. Chem. Int. Ed.* **60**, 9395–9400 (2021).

4. Yuan, X. & Wang, Y. A selenide catalyst for the activation of alkenes through Se•••π bonding. *Angew. Chem. Int. Ed.* **61**, e202203671 (2022).

5. Zhu, H., Zhou, P. & Wang, Y. Cooperative chalcogen bonding interactions in confined sites activate aziridines. *Nat. Commun.* **13**, 3563 (2022).

6. Omelanczuk, J. & Mikolajczyk, M. Optically Active Trivalent Phosphorus Compounds. 2. Reactivity of Alkylthio- and Alkylselenophosphonium Salts. The First Stereospecific Synthesis of a Chiral Phosphinite. *J. Am. Chem. Soc.* **101**, 7292–7295 (1979).

7. Walter, S. M., Kniep, F., Herdtweck, E. & Huber, S. M. Halogen-Bond-Induced Activation of a Carbon–Heteroatom Bond. *Angew. Chem. Int. Ed.* **50**, 7187–7191 (2011).

8. Zhao, Y. & Truhlar, D. G. The M06 suite of density functionals for main group thermochemistry, thermochemical kinetics, noncovalent interactions, excited states, and transition elements: two new functionals and systematic testing of four M06-class functionals and 12 other functionals. *Theor. Chem. Acc.* **120**, 215-241 (2008).

9. Grimme, S., Antony, J., Ehrlich, S. & Krieg. H. A consistent and accurate *ab initio* parametrization of density functional dispersion correction (DFT-D) for the 94 elements H-Pu. *J. Chem. Phys.* **132**, 154104 (2010).

10. Frisch, M. J. et al. Gaussian 09, Revision D.01; Gaussian, Inc.: Wallingford, CT, (2013).

11. Tomasi, J., Mennucci, B. & Cammi, R. Quantum Mechanical Continuum Solvation Models. *Chem. Rev.* **105**, 2999-3094 (2005).

12. Politzer, P., Murray, J. S. & Concha, M. C. σ-hole bonding between like atoms; a fallacy of atomic charges. *J. Mol. Model.* **13**, 643-650 (2007).

13. Kenny, P. W. Hydrogen Bonding, Electrostatic Potential, and Molecular Design. *J. Chem. Inf. Model.* **49**, 1234-1244 (2009).

14. Contreras-García, J., Johnson, E. R., Keinan, S., Chaudret, R., Piquemal, J.-P., Beratan, D. N. & Yang, W. NCIPLOT: A Program for Plotting Noncovalent Interaction Regions. *J. Chem. Theory Comput.* **7**, 625-632 (2011).

15. Johnson, E. R., Keinan,S., Mori-Sánchez, P., Contreras-García, J., Cohen, A. J. & Yang, W. Revealing Noncovalent Interactions. *J. Am. Chem. Soc.* **132**, 6498-6506 (2010).

16. Lu, T. & Chen, F.-W. Multiwfn: A multifunctional wavefunction analyzer, *J. Comput. Chem.* **33**, 580-592 (2012).

17. Humphrey, W., Dalke, A. & Schulten, K. VMD: Visual molecular dynamics. *J. Mol. Graph.* **14**, 33-38 (1996).
